# Technology mediation in child sexual exploitation and abuse in Africa and Asia

**DOI:** 10.1038/s41586-026-10525-4

**Published:** 2026-05-27

**Authors:** Sakshi Ghai, Matti Vuorre, Daniel Kardefelt-Winther, Amanda M. Ferguson, Sebastian Kurten, Sonia Livingstone, Andrew K. Przybylski, Amy Orben

**Affiliations:** 1 Department of Psychological and Behavioural Science, London School of Economics and Political Science, London, UK; 2 Department of Social Psychology, Tilburg University, Tilburg, The Netherlands; 3 UNICEF Office of Strategy and Evidence – Innocenti, Florence, Italy; 4 MRC Cognition and Brain Sciences Unit, University of Cambridge, Cambridge, UK; 5 Department of Interdisciplinary Social Science, Utrecht University, Utrecht, The Netherlands; 6 Department of Media and Communications, London School of Economics and Political Science, London, UK; 7 Oxford Internet Institute, University of Oxford, Oxford, UK

**Keywords:** Human behaviour, Developing world, Law

## Abstract

As digital access expands rapidly among children worldwide, technology-facilitated child sexual exploitation and abuse (CSEA), including online grooming, sexual solicitation, non-consensual image sharing and sexual extortion, has emerged as urgent yet underexamined category of digital harms^1^. Despite growing policy attention to online safety, evidence remains limited, particularly in low- and middle-income countries, where most of the world’s children live^2^. We analysed nationally representative survey data from 11,912 children aged 12–17 years across 12 countries in eastern and southern Africa and Southeast Asia, collected through the Disrupting Harm project in 2020–2021. We found that one in six internet-using children experienced at least one form of technology-facilitated CSEA, equivalent to over 10 million children. Despite this scale, many experiences went undisclosed, pointing to disclosure as a critical pathway for protection in the digital age. When children did disclose, they relied primarily on informal channels, especially friends, rather than formal reporting mechanisms such as police or helplines. Using Bayesian hierarchical models accounting for cross-country heterogeneity, we find that older children were less likely to disclose, whereas enabling parental mediation of online activities and children’s knowledge of where to seek help after sexual harassment or assault were associated with higher rates of disclosure. These findings provide population-level evidence to inform prevention and response across low- and middle-income countries, where coordinated action by policymakers, law enforcement and technology companies is urgently needed to protect all children.

## Abstract (web summary)

Enabling parental mediation of online activities and children’s knowledge of where to seek help after sexual harassment or assault is associated with higher rates of disclosure of technology-facilitated CSEA.

## Main

*Content* *warning:* *this Article contains information about child sexual exploitation and abuse, which some readers may find upsetting*.

Rapid digitization is driving increasing research on the online safety of children worldwide^1^, yet the impact of increasing digital connectivity in low- and middle-income countries (LMICs) has been routinely overlooked^2,3^. This gap is particularly important for Africa, where over 60% of citizens are aged under 25 years and the population is projected to double by 2050^4^, and for Asia, home to the majority of the world’s children and social media users^2,3^. While many initiatives across both regions have focused on digital inclusion to benefit children’s education^5^, understanding how widening internet access affects children’s exposure to online harms has become increasingly urgent.

Among the most concerning of these harms is the use of digital technologies to perpetrate sexual violence against children^6–8^, encompassing solicitation and dissemination of child sexual abuse material (images and videos), online grooming, live-streamed sexual abuse and sexual extortion^8^. These issues were highlighted in the 2024 US Senate Judiciary Committee^9^ hearing, which sought to hold popular social media companies to account for child safety issues on their platforms. Despite the efforts of multiple law enforcement authorities, UN organizations^10^, civil society organizations^11^ and national governments^12^, the true extent of technology-facilitated CSEA remains unclear for most LMICs, where children may face heightened risks of sexual violence^13^.

Effective prevention and response strategies require data-driven insights into the prevalence, root causes and effectiveness of protection systems. However, obtaining reliable data on child sexual abuse is extremely difficult. Ethical, sociocultural and safeguarding constraints limit what can be asked, how it should be asked and of whom it can be asked^14^. The varied international legal landscape further complicates research; in some countries legal thresholds vary and harms are not comprehensively recorded^15^. These challenges make research time consuming, costly and technically difficult, while limited funding and safeguarding infrastructure further constrain research capacity in LMICs^16^.

Here we address this evidence gap by analysing a multinational representative dataset investigating the nature and disclosure of technology-facilitated sexual exploitation and abuse experienced by children living in eastern and southern Africa and Southeast Asia. The Disrupting Harm project is a collaboration between UNICEF Office of Strategy and Evidence – Innocenti, ECPAT International and INTERPOL, funded by the Safe Online initiative, which collected survey data from nationally representative samples of 11,912 internet-using children aged 12 to 17 years across 12 countries (Ethiopia, Kenya, Mozambique, Namibia, Tanzania, Uganda, Cambodia, Indonesia, Malaysia, the Philippines, Thailand and Vietnam)^17^ between 2020 and 2021. These data therefore provide insights into technology-facilitated CSEA experienced by children in the African and Asian contexts.

To situate these data within current terminology, we adopt the Terminology Guidelines for the Protection of Children from Sexual Exploitation and Sexual Abuse (known as the Luxembourg Guidelines)^18^, developed through youth and expert stakeholder consultations. Importantly, this classification moves away from technological determinism while recognizing the increasingly pervasive role of technology in sexual crimes against children, across online and offline environments^19^. We use technology-facilitated CSEA to refer to children’s exposure to nine types of sexual harms measured in the Disrupting Harm survey (Methods). These include unwanted sexual attention through comments or images to clear instances of exploitation, including coercion into sexual conversations or activities, non-consensual sharing of children’s sexual images, offers of money or gifts in exchange for sexual content or contact and blackmail for sexual acts^10^. While the severity varies across these experiences, any such harms can lead to serious psychological, socioemotional and even physical consequences^20^. Referring to such experiences merely as risks can inadvertently contribute to the normalization of abuse^21^, discourage children from disclosing their experiences and even enable perpetrators to continue offending. Although not every online risk leads to harm^22^, adopting a harm-focused framing is crucial to fully recognizing the severity of children’s experiences, and safeguarding their rights^23,24^.

Research on technology-facilitated CSEA has expanded substantially in high-income countries^1^ yet has focused predominantly on prevalence^25–27^. Nationally representative surveys reveal wide variation in prevalence estimates due to definitional^18^ and methodological choices such as whether peer or adult solicitation is counted^28^, the recall period used (past year versus lifetime)^1^ and sampling denominators (all children versus internet users only)^29^. For example, 17.7% of Australian 16–24-year-olds reported adult online sexual solicitation before age 18^30^, while US estimates indicate that adding online abuse items can increase overall CSA prevalence estimates from 13.5% to 21.7% depending on how online harms are operationalized^25^. A recent systematic review and meta-analysis estimates that roughly 1 in 12 children globally has experienced online CSEA (pooled past-year prevalence, 8.1%)^1^. While such studies provide critical epidemiological functions such as mapping population burden, guiding resources, and enabling surveillance^31^, prevalence data alone cannot reveal whether children seek help after harm. Understanding disclosure pathways is therefore equally critical for effective prevention and response^32^.

To date, research on disclosure of technology-facilitated CSEA remains limited^33,34^. Where it exists, it often draws on forensic or criminal-justice settings^35–37^, which include victims who have already navigated formal reporting systems. Recent qualitative studies have identified barriers to disclosure such as self-blame, shame and lack of trust^33^, yet empirical investigations into facilitators or enablers of disclosure in online environments has been limited^32,38–40^. Evidence from broader child sexual abuse research indicates that disclosure marks a pivotal step for identifying and stopping offline sexual abuse^32,41^, facilitating recovery^42^ and reducing the long-term mental health impacts^32,43,44^. Children tend to first disclose to peers rather than formal reporting channels such as police, helplines or teachers^32,45,46^. Yet little is known about disclosure of technology-facilitated CSEA in LMICs^34^, where formal and informal support systems may differ substantially from those in high-income countries. Cross-cultural research on prevalence and disclosure is therefore needed to inform timely prevention, support and protection.

Guided by child-rights^47^ and public-health^48–50^ frameworks, we examine disclosure patterns and associated factors through a socio-ecological lens^51–53^. The UN Committee on the Rights of the Child’s General Comment No. 25 affirms that protection in digital environments depends on accessible, child-friendly pathways to seek help^24,47^. From a public-health perspective, primary prevention (preventing abuse before it occurs) is the foundational priority, supported by secondary (early detection and intervention) and tertiary (treatment and rehabilitation) responses^50^. These tiers are interdependent^48^ as disclosure operates primarily within secondary and tertiary prevention, yet patterns of disclosure and non-disclosure can highlight how systems prevent, detect and respond to harm. When children cannot disclose, whether due to a lack of awareness, inaccessible systems or a lack of trusted adults, these barriers may signal gaps in primary prevention infrastructure^50^. Conversely, when disclosure does occur, timely intervention and trauma-informed care become possible. Understanding how, to whom and under what conditions children disclose technology-facilitated CSEA can therefore guide more targeted interventions, strengthen support pathways and ultimately reduce subsequent harms to children^32^.

In this cross-sectional study, we address two questions (Methods): (1) what are the prevalence and disclosure rates of technology-facilitated CSEA in these contexts; and (2) which demographic, family, cultural and protective factors are associated with disclosure? This study provides researchers, policymakers, child-protection and education systems and law enforcement with population-level data on children living in LMICs who are both highly vulnerable and systematically under-represented on the global scientific stage.

## Technology-facilitated CSEA

To address the first research question, we identify the prevalence of each of nine categories of technology-facilitated CSEA, reporting the rates for internet-using 12–17-year-old children and population-adjusted estimates for all children. Pooled across 12 nationally representative samples and surveys of 11,912 internet-using children in eastern and southern Africa and Southeast Asia, we found that one in six (17%; 95% confidence interval (CI) = 16.2–17.8; weighted *n* = 2,025) had experienced at least one instance of technology-facilitated CSEA in 2020–2021. Across all instances of CSEA, one in three (31%, 95% CI = 30.1–32.1; weighted *n* = 3,477; see Supplementary Information 2.4) internet-using children experienced some form of sexual exploitation or abuse (Supplementary Tables 3, 49 and 51), including incidents occurring online (social media, online games), in person, some other way or where the setting was not specified (do not know/prefer not to say).

Specifically, on social media or in an online game, about 10% of children received unwanted sexual images (95% CI = 9.0–10.2; *n* = 1,143) and 8% received sexual comments that made them feel uncomfortable (95% CI = 6.9–8.0; *n* = 889). Moreover, 5% (95% CI = 4.4–5.2; *n* = 571) were asked to discuss sex or sexual acts, while 4% (95% CI = 3.5–4.3; *n* = 462) were asked online to explicitly do something sexual. Furthermore, 4% (95% CI = 3.8–4.6; *n* = 498) of children were asked for a photo or video showing their private parts, and about 3% (95% CI = 2.4–3.1; *n* = 328) of children were offered money or gifts online to meet the perpetrator in person to perform sexual acts. Finally, 3% of children reported that their sexual images were shared without their consent (95% CI = 2.5–3.1; *n* = 332), or they were offered money or gifts to share sexual images (95% CI = 2.4–3.1; *n* = 324) and even blackmailed online to engage in sexual activity (95% CI = 2.1–2.8; *n* = 294) (Supplementary Table 50). Further details on the digital platforms and perpetrator identity associated with technology-facilitated CSEA are provided in Supplementary Figs. 17–19 and Supplementary Tables 42, 43, 47 and 48.

**Fig. 1:**
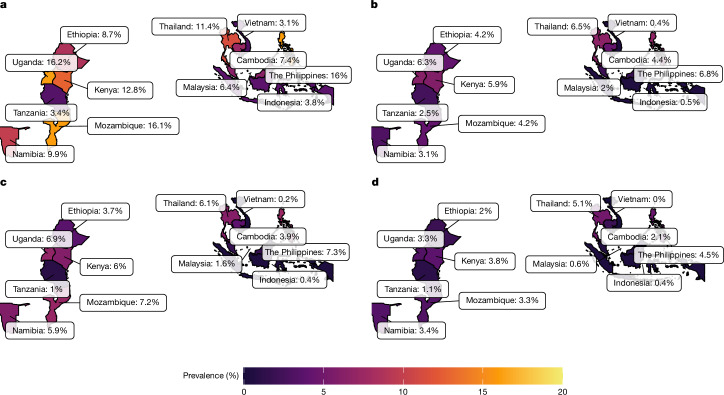
Estimated prevalence of four forms of technology-facilitated CSEA across 12 countries in Africa and Asia in 2020–2021. The estimated proportion of 12–17-year-old internet-using children who reported their experience of technology-facilitated CSEA in 2020–2021 across the 12 surveyed countries in eastern and southern Africa and Southeast Asia (*n* = 11,912; per country* n* values are reported in the Methods). Sampling weights were applied to render our estimates representative of the digitally connected children of each country. **a**–**d**, Four of the nine technology-facilitated CSEA categories occurring through social media or online game platforms. **a**, Someone sent me sexual images I did not want. **b**, Been asked by someone to do something sexual when I did not want to. **c**, Someone asked for a photo or video showing my private parts. **d**, Someone threatened or blackmailed me to engage in sexual activities. The full set of nine technology-facilitated CSEA types is presented in Supplementary Figs. 2–10. Basemap boundaries were created using Natural Earth (https://naturalearthdata.com).

**Extended Data Fig. 1:**
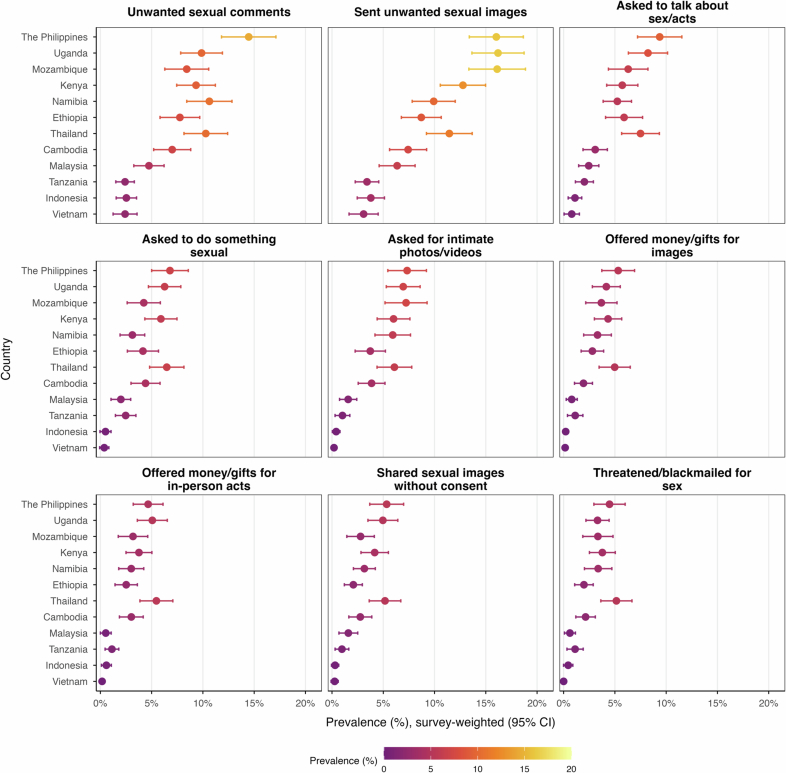
Survey-weighted prevalence of nine types of technology-facilitated CSEA by country. Survey-weighted prevalence estimates with 95% design-based confidence intervals for nine types of technology-facilitated CSEA experienced by internet-using children aged 12–17 years across 12 countries (n = 11,912, 2020-2021). Points represent the survey-weighted prevalence estimate (measure of centre). Error bars show 95% design-based confidence intervals and are suppressed where country-specific cell sizes were fewer than three observations. Colour indicates prevalence (lighter = higher). No statistical hypothesis tests were performed. Countries are ordered by overall technology-facilitated CSEA prevalence: Philippines, Uganda, Mozambique, Kenya, Ethiopia, Namibia, Thailand, Cambodia, Malaysia, Tanzania, Indonesia, and Vietnam. Full country-specific estimates are reported in Supplementary Table 52.

Country-level prevalence estimates of technology-facilitated CSEA ranged from an estimated 5.5% (95% CI = 3.7–7.3; *n* = 54) of internet-using children in Vietnam to 29% (95% CI = 25.3–31.9; *n* = 271) in the Philippines. Extended Data Fig. 1 presents survey-weighted prevalence for each specific type of technology-facilitated CSEA across 12 countries, with 95% confidence intervals. For example, less than 1% of children in Vietnam (95% CI = 0.0–1.5; *n* = 8) were asked to talk about sex or sexual acts online; in the Philippines, this figure was almost 9% (95% CI = 7.2–11.5; *n* = 89) (Fig. 1, Supplementary Figs. 2–10 and Supplementary Table 52).

**Extended Data Fig. 2:**
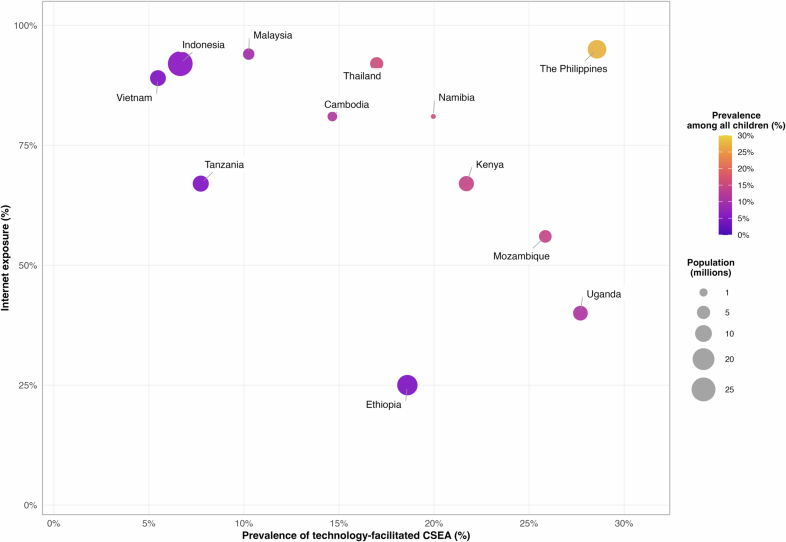
Relationship between technology-facilitated CSEA prevalence among internet users, internet exposure, and estimated population-level prevalence among all children. Scatter plot showing prevalence of technology-facilitated CSEA among internet-using children aged 12–17 years (x-axis) against internet exposure among all children aged 12–17 years (y-axis), across 12 countries. Bubble size is proportional to the national population of children aged 12–17 years (in millions). Colour denotes estimated overall prevalence among all children aged 12–17 years, calculated as the product of CSEA prevalence among internet users and internet exposure. All prevalence estimates are survey-weighted. Data sources: Disrupting Harm child and household surveys (n = 11,912; 2020–2021) and UN World Population Prospects 2022 (2020 population estimates).

To calculate the proportion of all children aged 12–17 years experiencing technology-facilitated CSEA, we multiplied each country’s internet penetration rate (collected through Disrupting Harm household surveys) by prevalence among internet users (Table 1 and Extended Data Fig. 2). Across 12 countries, this corresponds to approximately 10.7 million children (95% CI = 9.9 million–11.5 million; Supplementary Information 2.1–2.2 and Supplementary Fig. 15). In countries with widespread internet penetration, such as the Philippines (95%), Malaysia (94%) and Thailand (92%), the prevalence for the child population closely mirrors that among children with internet access. In countries with more limited access, such as Ethiopia (25%) and Uganda (40%), the overall prevalence among all children was 5% and 11%, respectively, reflecting differences in both internet exposure and prevalence among internet-using children. Countries with large youth populations such as Indonesia (6% among all children) and the Philippines (27% among all children) also face particular challenges as these rates translate into substantial absolute numbers of affected children. As internet connectivity continues to expand across LMICs, the population-level burden of technology-facilitated CSEA may grow substantially over the next decade.

**Table 1:** Country-level prevalence and population impact of technology-facilitated CSEA among children aged 12–17 years (2020–2021)

Country	Prevalence of technology-facilitated CSEA among internet using children aged 12–17 (%)	Proportion of child internet users, aged 12–17 (%)	Prevalence of technology-facilitated CSEA among all children (%) (uncertainty, %)	Estimated number of children affected	Child population aged 12–17
Cambodia	14.7	81.0	11.9 (9.9–14.1)	215,962 (179,843–256,539)	1,819,679
Ethiopia	18.6	25.0	4.6 (3.5–6.1)	775,526 (581,437–1,018,510)	16,680,781
Indonesia	6.6	92.0	6.1 (4.6–7.9)	1,665,769 (1,250,027–2,150,872)	27,231,704
Kenya	21.7	67.0	14.5 (12.4–16.8)	1,096,163 (932,413–1,269,843)	7,539,254
Malaysia	10.2	94.0	9.6 (7.6–12.0)	306,234 (241,236–380,807)	3,178,610
Mozambique	25.9	56.0	14.5 (12.3–17.0)	634,331 (536,934–744,858)	4,381,428
Namibia	20.0	81.0	16.2 (13.6–18.9)	47,686 (40,172–55,612)	294,899
The Philippines	28.6	95.0	27.1 (23.1–30.6)	3,580,485 (3,043,351–4,035,759)	13,190,360
Tanzania	7.7	67.0	5.2 (4.0–6.7)	456,274 (353,053–589,653)	8,813,838
Thailand	17.0	92.0	15.6 (13.0–18.2)	769,133 (642,336–896,437)	4,922,710
Uganda	27.7	40.0	11.1 (9.1–13.3)	755,632 (623,496–907,413)	6,819,466
Vietnam	5.5	89.0	4.9 (3.6–6.5)	410,438 (300,307–548,994)	8,421,996

The first column lists countries included in the Disrupting Harm data. The second column shows the prevalence of technology-facilitated CSEA among internet-using 12–17-year-old children who experienced any form of technology-facilitated CSEA in 2020–2021, estimated from the Disrupting Harm child survey using nationally representative probability samples with design weights. The third column shows the proportion of child internet users: percentage of all 12–17-year-old children in each country who use the internet (any use, any device), estimated from the Disrupting Harm household survey module administered at every sampled household. The fourth column shows the prevalence of technology-facilitated CSEA among all children as the percentage of all 12–17-year-old children who experienced technology-facilitated CSEA, calculated by multiplying prevalence among internet users (second column) by the proportion of child internet users (third column) in each country; uncertainty was propagated using Monte Carlo simulation (5,000 iterations per country). The fifth column shows the estimated number of affected children, representing the absolute number of 12–17-year-old children estimated to have experienced technology-facilitated CSEA in each country, calculated by applying the prevalence among all children (column 4) to the total population aged 12–17 (column 6). 95% CIs reflect uncertainty in prevalence only (population treated as fixed). The sixth column shows the child population aged 12–17, representing the total number of children aged 12–17 in each country, derived from United Nations World Population Prospects 2022 (reference year, 2020).

## Demographic differences

Among internet-using children aged 12–17 years, the overall prevalence of technology-facilitated CSEA through social media or gaming platforms was nearly identical for boys and girls: 16.9% (95% CI = 15.9–18.0; *n* = 1,026) of boys and 17.0% (95% CI = 15.9–18.1; *n* = 999) of girls experienced at least one form of technology-facilitated CSEA. Prevalence increased with age from 11% (95% CI = 9.5–13.3; *n* = 161) among 12-year-old children to 22% (95% CI = 20.4–23.7; *n* = 558) among 17-year-old children (Supplementary Table 39). A Bayesian multilevel model confirmed that there is no meaningful gender difference in the probability of experiencing technology-facilitated CSEA (posterior mean = −0.01, s.d. = 0.11, 95% Bayesian credible interval (CrI) = −0.22–0.20, posterior probability of direction (PD) = 54%), with 99.7% of the posterior distribution falling within the region of practical equivalence (ROPE; odds ratios, 0.67–1.50). Older age was associated with higher probability of experiencing technology-facilitated CSEA (posterior mean = 0.18, s.d. = 0.04, 95% CrI = 0.10–0.26, PD > 99.9%), equivalent to approximately 2.7 percentage points higher probability per additional year of age (average marginal effect (AME), 95% CI = 2.2–3.1). Although the age gradient was slightly stronger for girls compared with boys (posterior mean = 0.09, s.d. = 0.04, 95% CrI = 0.01–0.17, PD = 98.5%), the overall predicted difference between girls and boys was small and the CrI included zero (AME = 0.8 percentage points; 95% CI = −0.6–2.0).

**Fig. 2:**
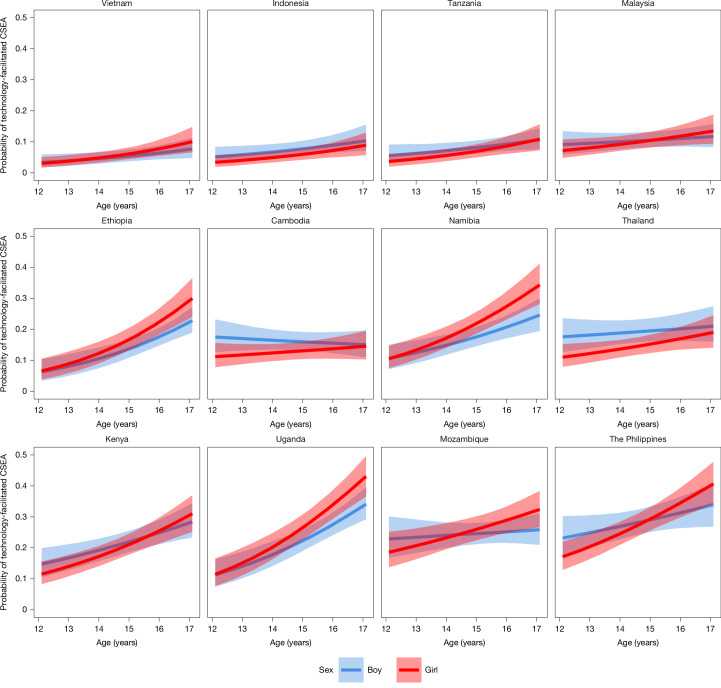
Predicted probability of experiencing one or more forms of technology-facilitated CSEA by age and gender across 12 countries. Predictions were derived from a survey-weighted Bayesian multilevel logistic regression (Bernoulli family), with gender, age and their interaction as fixed effects, and country-varying intercepts and slopes for gender, age and their interaction (*n* = 11,912 internet-using children). The *x* axis shows age in years (12–17 years) and the *y* axis shows the predicted probability (0–0.50).  The lines show the posterior mean predicted probabilities for girls (red) and boys (blue); the shaded bands indicate the 95% CrI. Panels are ordered by country-level mean predicted probability.

The predicted probabilities of technology-facilitated CSEA varied substantially across countries (Fig. 2, Supplementary Table 7 and Supplementary Fig. 21). Among girls aged 17 years, predicted probabilities were highest in Uganda (42%, 95% Crl = 0.36–0.49) and the Philippines (40%, 95% CrI = 0.34–0.47). Among boys aged 17 years, predicted probabilities were highest in Uganda (34%, 95% CrI = 0.29–0.39) and the Philippines (34%, 95% CrI = 0.27–0.40). In Vietnam, predicted probabilities were among the lowest, ranging from 3% for boys aged 12 years (95% CrI = 0.02–0.06) to 8% (95% CI = 0.05–0.11) for boys aged 17 years and 3% for girls aged 12 years (95% CrI = 0.02–0.05) to 10% (95% CrI = 0.06–0.14) for girls aged 17 years. These differences could reflect variability in the likelihood of reporting across countries rather than true prevalence differences alone.

The prevalence of technology-facilitated CSEA also varied by degree of urbanization after adjusting for gender and age (Supplementary Fig. 23 and Supplementary Table 39). Children in peri-urban settings reported the highest prevalence. Rural children had lower prevalence than peri-urban children (AME = −4.4 percentage points, 95% CrI = −7.1 to −1.9), and urban children had higher prevalence than rural children (AME = 2.4 percentage points, 95% CrI = 1.0–3.9). The difference between urban and peri-urban settings was small and uncertain (AME = −2.0 percentage points, 95% CrI = −4.8–0.7). Full model results are reported in Supplementary Tables 8 and 9.

## Disclosure of technology-facilitated CSEA

We next identified to whom children disclosed CSEA, distinguishing between formal and informal channels. Formal channels are typically embedded within statutory child protection systems (such as police, teachers or social workers), whereas informal channels offer more personal and familial support (such as friends, family or peers). This distinction serves as an analytic heuristic to organize disclosure pathways based on the role of the recipient rather than the outcome of the disclosure (that is, whether it triggers an investigation), consistent with past research^54,55^ (Supplementary Table 34).

Of an estimated 2,025 children who experienced one or more forms of technology-facilitated CSEA (weighted), 51% (95% CI = 48.6–53.6; *n* = 1,034) did not disclose it to anyone (responses by countries and CSEA types are shown in Supplementary Fig. 24 and Supplementary Tables 40 and 41). Among those who did disclose, most did so through informal channels, including close family members, friends or other trusted adults. Specifically, 46% (95% CI = 43.1–48.1; *n* = 923) disclosed to friends, while many also shared their experience with family members, with 26% (95% CI = 23.6–28.0; *n* = 523) disclosing to siblings, 21% (95% CI = 19.3–23.4; *n* = 432) to mothers and 20% (95% CI = 17.9–21.9; *n* = 403) to fathers. Rarely did children disclose to any formal channels, such as the police (3%, 95% CI = 2.3–4.2; *n* = 66), helplines (3%, 95% CI = 2.4–4.2; *n* = 67), social workers (3%, 95% CI = 2.0–3.8; *n* = 59) or teachers (9%, 95% CI = 7.3–10.1; *n* = 177). About 6% (95% CI = 5.1–7.5; *n* = 127) told other trusted adults and 1% (95% CI = 0.6–1.6; *n* = 22) disclosed through other channels. As children could select more than one disclosure type, these percentages are non-additive (mutually exclusive categories are shown in Supplementary Fig. 25).

**Fig. 3:**
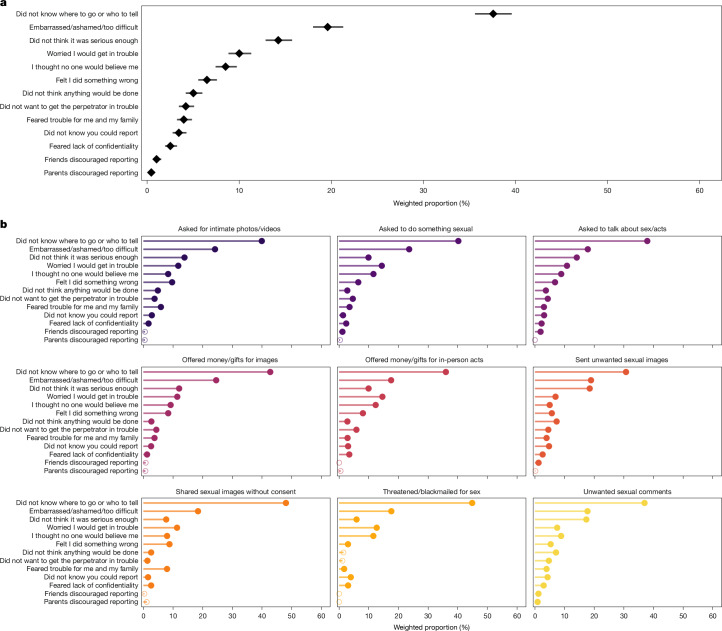
Barriers to disclosure of technology-facilitated CSEA in children aged 12–17 years. **a**, The overall barrier prevalence across all CSEA types among children who did not disclose their experiences among *n* = 2,921 non-disclosed incidents (unweighted sample). Data are presented as survey-weighted proportions (diamond markers) with 95% Wilson score CIs (horizontal lines). **b**, Barriers stratified by each of the nine technology-facilitated CSEA types. The points show survey-weighted proportions. The *x* axis shows the survey-weighted proportion (%) of non-disclosed instances in which each barrier was reported; the* y* axis lists barrier types. Estimates are weighted proportions with 95% CIs using Wilson score methods incorporating survey weights. In **b**, estimates with fewer than ten observations or fewer than three positive responses are shown without CIs (hollow points) due to insufficient precision; estimates with CI width exceeding 20 percentage points are marked with semi-transparent points. As barrier items were multi-select, within-type proportions do not sum to 100%. Full numeric results are provided in Supplementary Table 46.

For non-disclosed incidents of technology-facilitated CSEA, children were asked to select all reasons why they had not told anyone. Responses were recorded per CSEA type (Supplementary Tables 45 and 46 and Supplementary Fig. 26). As respondents could select multiple barriers for each non-disclosed incident, the percentages do not sum to 100% within any CSEA type. Pooled across CSEA types (Fig. 3a), the most common barrier to disclosure was “Did not know where to go or who to tell” (37.6%, 95% CI = 35.6–39.6), followed by “feeling embarrassed, ashamed or that it would be too emotionally difficult to tell” (19.6%, 95% CI = 18.0–21.3) and “did not think it was serious enough to report” (14.2%, 95% CI = 12.9–15.7). Figure 3b shows stratified barriers by each CSEA type. For example, among non-disclosed instances of sharing sexual images without consent, 48.1% (95% CI = 39.8–56.5) cited “did not know who to tell” as a barrier, while 18.4% (95% CI = 12.8–25.9) reported feeling embarrassed or ashamed.

## Factors associated with disclosure

To address the second research question and understand the factors associated with disclosure, our analysis focused on a set of interconnected predictors across four conceptual levels: individual, family, cultural and protective. First, we examined demographic factors, such as a child’s age and gender, based on extensive evidence linking these factors to a higher likelihood of both offline and online disclosure^56,57^. Second, enabling parental mediation or supportive engagement by parents around a child’s use of the internet was identified as a key factor. Previous research shows that supportive parental involvement is associated with a child’s vulnerability to online risks^58,59^, highlighting the importance of parental scaffolding within the family unit. Third, we examine cultural factors, as well as gender norms and attitudes towards sex, including patriarchal views on violence, honour and control of girls. These factors are known to shape both the perceived severity of risks and the likelihood of disclosure in LMICs^60^.

Finally, we assessed a set of protective factors that may equip children with the tools and knowledge needed to report harmful experiences^61^, including sex education^62^, digital skills to report^63^ and children’s knowledge of where to seek help after sexual assault or harassment (help-seeking)^64^. Operationally, we analyse these as child-level indicators of protective factors that span the three levels of our socioecological model: acquired and enacted by children (individual), reinforced by caregivers, peers and schools (family/community), and enabled or constrained by curricula, safeguarding policies and legal frameworks (cultural).

**Fig. 4:**
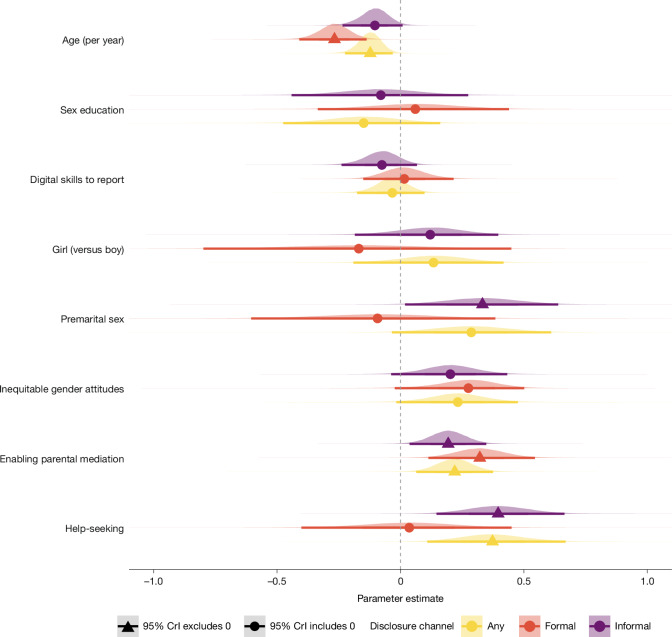
Predictors of disclosure of technology-facilitated CSEA to any, formal and informal channels. Parameter estimates from three Bayesian multilevel logistic regression models of disclosure (any channel, formal and informal) among *n* = 2,067 children (unweighted sample) who experienced at least one incident of technology-facilitated CSEA. The *x* axis shows log-odds coefficients, the *y* axis shows different demographic and sociocultural predictors. The points represent the posterior median values and the horizontal lines denote the 95% CrI. The posterior density curves visualize uncertainty. The triangles indicate estimates of which the 95% CrI excludes zero; the circles indicate intervals that include zero. The vertical dotted line serves as a reference point, marking 0 as no association; positive values indicate a higher probability of disclosure. Models are survey-weighted; estimates are pooled across 30 imputations.

An exploratory Bayesian multilevel model examined factors associated with children’s disclosure of technology-facilitated CSEA through any channel (that is, formal or informal). Older children were less likely to disclose these experiences (posterior mean = −0.12, s.d. = 0.05, 95% CrI = −0.22 to −0.03, PD = 99.4%) but disclosure did not differ by gender (posterior mean = 0.13, s.d. = 0.15, 95% CrI = −0.19–0.42, PD = 81.9%). By contrast, more parental involvement in children’s digital lives (that is, enabling parental mediation) was associated with higher overall disclosure (posterior mean = 0.22, s.d. = 0.08, 95% CrI = 0.06–0.38, PD = 99.4%). Similarly, children who knew where to seek help after an assault or harassment (that is, help-seeking knowledge) were more likely to disclose (posterior mean = 0.38, s.d. = 0.14, 95% CrI = 0.11–0.67, PD = 99.5%). No associations with disclosure were found for sex education, attitudes towards premarital sex, inequitable gender attitudes and digital skills to report (95% CrI crossed zero) (Fig. 4 and Supplementary Table 23).

Two supplementary exploratory analyses compared children’s disclosure through informal or formal channels, revealing distinct factors associated with each. Children who knew where to seek help after sexual assault or harassment (posterior mean = 0.40, s.d. = 0.13, 95% CrI = 0.15–0.66, PD = 99.8%) and who received enabling parental mediation (posterior mean = 0.19, s.d. = 0.08, 95% CrI = 0.04–0.35, PD = 98.9%) were more likely to disclose through informal channels, potentially reflecting that the former addresses a key barrier to disclosure (Fig. 3a). Children who believed in the acceptability of premarital sex (posterior mean = 0.33, s.d. = 0.16, 95% CrI = 0.02–0.64, PD = 98%) were also more likely to disclose to friends, family and other adults. Only two predictors were related to disclosure through formal channels: older children were less likely to disclose (posterior mean = −0.27, s.d. = 0.07, 95% CrI = −0.41 to −0.14, PD = 99.9%), while children who received enabling parental mediation were more likely to do so (posterior mean = 0.32, s.d. = 0.11, 95% CrI = 0.11–0.54, PD = 99.7%).

**Fig. 5:**
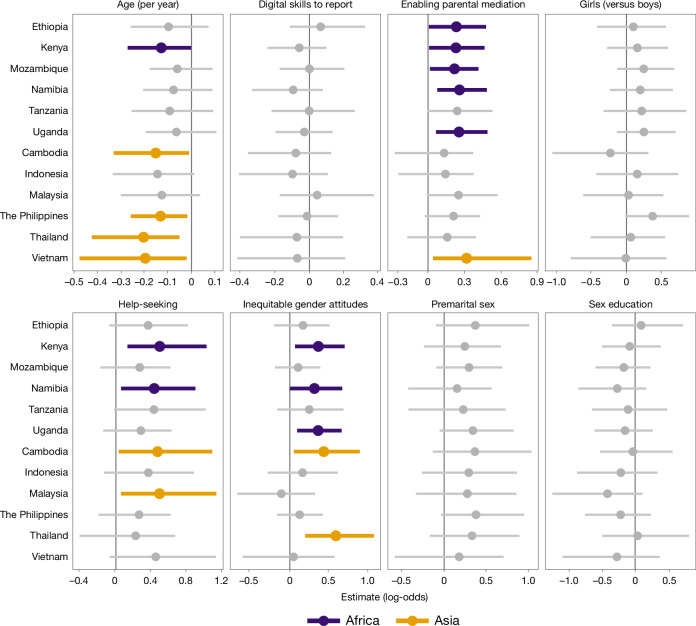
Country-specific predictors of any disclosure of technology-facilitated CSEA. Parameter estimates are derived from a Bayesian multilevel logistic regression of any disclosure among *n* = 2,067 children (unweighted sample) who experienced at least one technology-facilitated CSEA incident. The points show the posterior means (log-odds of disclosure) from a model with random intercepts and random slopes; the horizontal bars show the 95% CrIs. Purple indicates African countries (Ethiopia, Kenya, Mozambique, Namibia, Tanzania, Uganda); orange indicates Asian countries (Cambodia, Indonesia, Malaysia, the Philippines, Thailand, Vietnam). The solid marks denote effects with 95% CrIs excluding zero; the faded marks denote estimates with intervals including zero. The model includes all predictors simultaneously and accounts for country-level heterogeneity in baseline disclosure and predictors. Models are survey-weighted; estimates are pooled across 30 imputations.

**Extended Data Fig. 3:**
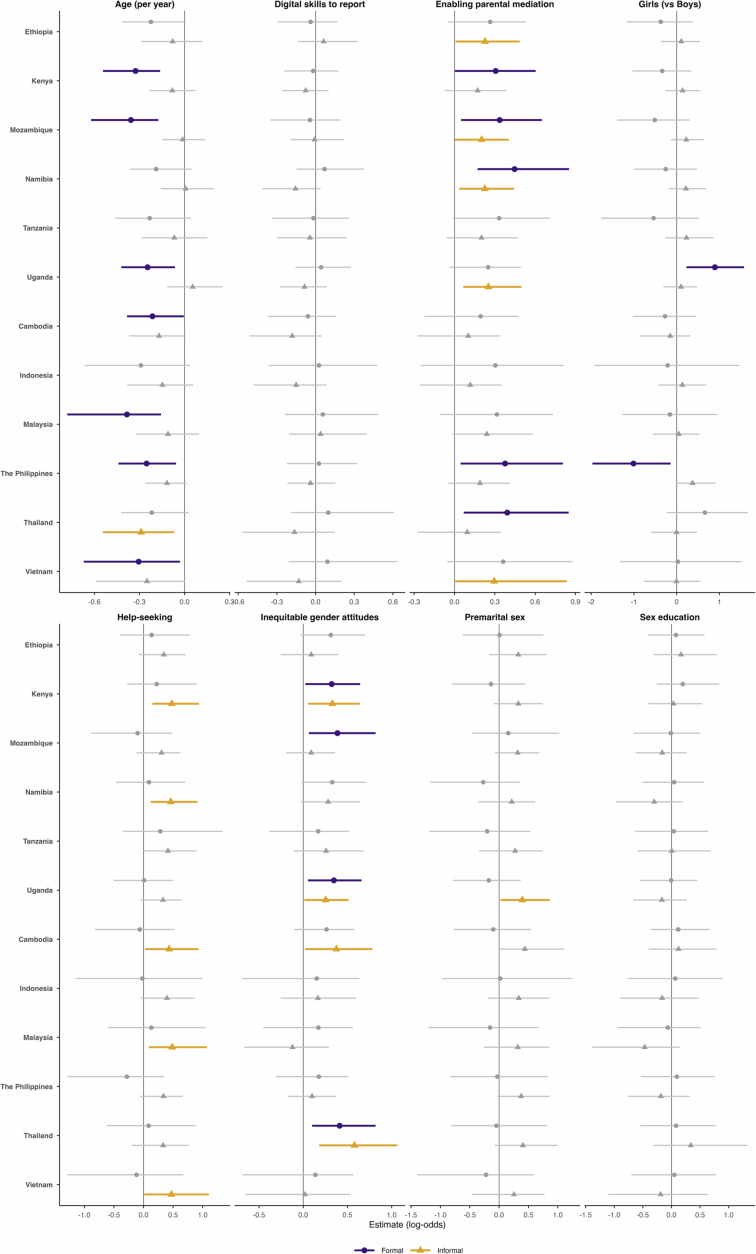
Country-specific associations between predictors and disclosure by channel type. Points show posterior means (log-odds scale) from Bayesian multilevel logistic regression models with random intercepts and slopes; horizontal bars represent 95% credible intervals. Formal channels include police, helpline, social worker, and teacher; informal channels include family members (siblings, father, mother), friends, and other adults. Purple denotes formal channels and orange denotes informal channels (see panel legend for shape and colour keys). Faded grey points and intervals indicate estimates whose 95% credible intervals include zero. Countries are ordered by region (African countries above; Asian countries below). Each model adjusts for all predictors simultaneously and accounts for clustering at the country level. Models are survey-weighted; estimates are pooled across 30 imputations. Sample: n = 2,067 children (unweighted sample) who experienced at least one technology-facilitated CSEA incident. Full country-specific estimates are reported in Supplementary Table 30.

The above results reflected average trends across 12 countries, examining country-specific estimates reveals substantial between-country heterogeneity in factors associated with disclosure of technology-facilitated CSEA (Fig. 5 and Supplementary Table 30). For disclosure to any channel, enabling parental mediation was associated with higher disclosure in 6 out of 12 countries (95% CrI excludes 0). Knowing where to seek help was positively associated with disclosure in 4 out of 12 countries; older age was associated with lower disclosure in 5 out of 12 countries; and more inequitable gender attitudes were associated with higher disclosure in 5 out of 12 countries. Country-level estimates are further disaggregated by formal versus informal channels in Extended Data Fig. 3, underscoring the complexity of cross-cultural comparisons.

## Model comparisons and robustness checks

Consistent with our preregistered analysis plan, we conducted multiple model comparisons, including: (1) a logistic regression model (generalized linear model, GLM) with fixed effects; (2) a multilevel logistic regression model with random intercepts to account for differences by country; (3) an interaction model incorporating age and gender; and (4) regularized regression models to evaluate the stability of predictors (full summaries for all specifications are provided in the Supplementary Tables 10–30).

**Extended Data Fig. 4:**
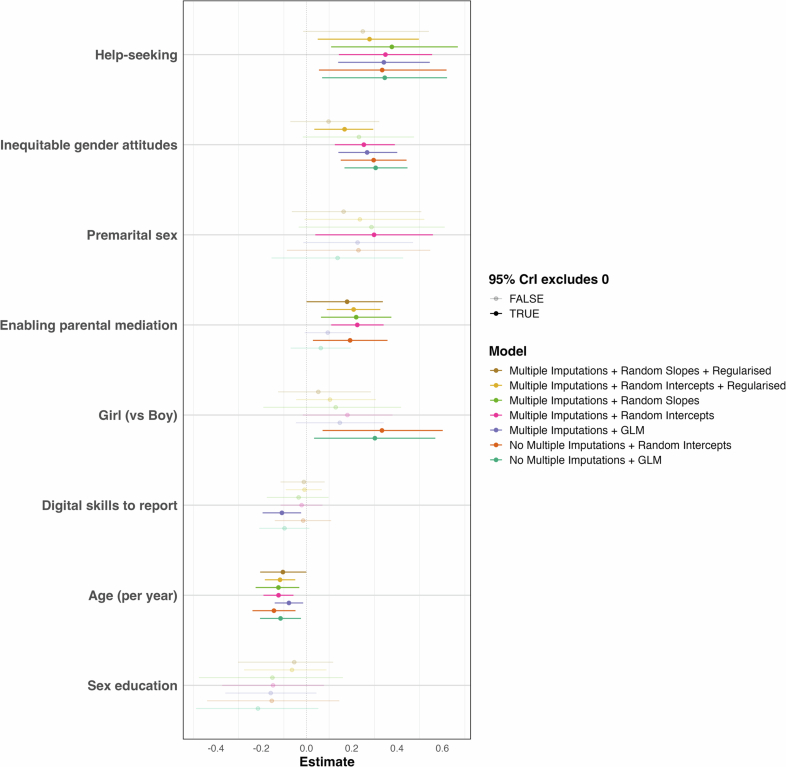
Sensitivity of disclosure associations across model specifications. Parameter estimates from seven model variants predicting disclosure via any channel. Points represent posterior means and horizontal line segments show corresponding 95% credible intervals. The x-axis shows posterior estimates (log-odds scale) and the y-axis shows predictors. Models differ in their handling of missing data and model structure: models without multiple imputation include a standard Bayesian logistic regression and a random-intercepts model; models incorporating multipl imputations include a logistic regression (GLM), a random-intercepts model, a random-intercepts-and-slopes model, a horseshoe-penalised random-intercepts model, and a random-intercepts-and-slopes model with horseshoe penalisation.Sample: n = 2,067 children (unweighted sample) who experienced at least one technology-facilitated CSEA incident; estimates are survey-weighted.

While the GLM represented our confirmatory model (Supplementary Table 12), we do not draw inferential conclusions from this model, owing to limitations in its ability to account for country-level heterogeneity (see the ‘Deviations from preregistration’ section). Instead, we relied on a Bayesian multilevel model as our primary inference model, including both random intercepts and slopes to account for the nested data structure. This analytical approach aligned with the theoretical expectation that associations between predictors and disclosure may differ meaningfully across countries. Across all models predicting disclosure, key predictors consistently showed directional associations, although the certainty of these estimates varied across specifications (all model specifications are shown in Extended Data Fig. 4).

To ensure the robustness of our findings, we specifically estimated two regularized regression models that apply shrinkage to coefficient estimates to reduce the risk of overfitting and by offering more-conservative specifications (Supplementary Table 27). First, we fit a model with random intercepts only, constraining predictors to be constant across countries. This enabled us to test whether the key associations were sensitive to the exclusion of varying slopes. All three key predictors were retained, consistent with the patterns observed in our primary Bayesian multilevel model, knowing where to seek help (posterior mean = 0.28, s.d. = 0.11, 95% CrI = 0.05–0.50, PD = 99.4%), enabling parental mediation (posterior mean = 0.21, s.d. = 0.06, 95% CrI = 0.09–0.33, PD = 99.9%) and age (posterior mean = −0.12, s.d. = 0.03, 95% CrI = −0.18 to −0.05, PD = 99.9%). We also found that inequitable gender attitudes were positively correlated with disclosure (posterior mean = 0.17, s.d. = 0.3, 95% CrI = 0.03–0.29, PD = 99.4%), suggesting that having more traditional beliefs regarding gender roles was associated with a higher likelihood of disclosing.

Second, we estimated a more conservative model that included both random intercepts and slopes, allowing associations to vary across countries. Age (posterior mean = −0.10, s.d. = 0.05, 95% Crl = −0.20–0.00, PD = 97.7%) and enabling parental mediation (posterior mean = 0.18, s.d. = 0.09, 95% CrI = 0.00–0.34, PD = 97.6%) continued to be associated with disclosure, although their credible intervals approached zero, reflecting more uncertainty. Children’s knowledge of where to seek help remained positively associated (posterior mean = 0.25, s.d. = 0.15, 95% CrI = −0.01–0.54, PD = 95.4%) but showed reduced certainty.

## Discussion

In this Article, we provide nationally representative evidence from 12 LMICs showing substantial exposure to technology-facilitated CSEA and critically low disclosure through formal channels. Approximately 1 in 6 of the 11,912 internet-using children reported experiencing at least one instance of technology-facilitated CSEA during 2020–2021. It is important to note that this figure cannot be directly extrapolated to the general population of children. Scaled by the share of child internet users in each country, we estimate that at least 10 million children were exposed to technology-facilitated CSEA over a single year alone. While population-based surveys capture prevalence more accurately than administrative data or case reports, they probably still underestimate the true scale of sexual violence due to social desirability bias, stigma, shame, fear of social repercussions and ongoing victimization^65^. Even under these conservative assumptions, this is a growing problem affecting millions of children globally.

By analysing data from children living in eastern and southern Africa and Southeast Asia, including peri-urban and rural populations, often under-represented in behavioural research, this study provides population-level insights from regions where such data have been largely absent. We find that although older children were more likely to experience technology-facilitated CSEA, they were less likely to disclose such experiences. As the survey captured only past year experiences, cumulative exposure (particularly among older adolescents) is probably underestimated. These findings highlight the need for prevention efforts that begin in early childhood and are sustained through late adolescence, adapting to children’s evolving developmental contexts. Both qualitative and longitudinal data are needed to understand how developmental trajectories influence vulnerabilities, the likelihood of disclosure over time and how decisions around disclosures are shaped by children’s broader life circumstances.

We found no gender differences in exposure rates, although this may mask important differences in context and consequences. Emerging evidence from the Disrupting Harm project^17^ indicates that societal expectations shape how perpetrators target children, how children interpret their experiences and the responses that they receive. Prevention strategies must therefore account for gendered dimensions of risk and response, even when prevalence rates appear similar^66^. If internet access differs by gender (for example, in many LMICs), absolute exposure numbers may vary substantially, therefore warranting gender-disaggregated monitoring and support.

Disclosure most often occurred through informal networks such as friends and family. By contrast, very few children reported their experiences to formal systems such as police, teachers, helplines or social workers, even in serious cases involving threats or blackmail. This gap in formal disclosure presents a critical challenge for law enforcement authorities, potentially limiting their ability to detect and respond to technology-facilitated CSEA^67^ and restricting children’s access to professional support and justice.

“Not knowing where to go or who to tell” was the most common barrier to disclosure, suggesting gaps in both awareness of formal channels and access to trusted adults. Although basic information on where to report sexual abuse should be provided to all children from an early age (for example, schools, health systems), formal reporting channels must be made more accessible and responsive to encourage disclosure^68^. Addressing other barriers identified in this study, including worries about not being believed, self-blame, fear of repercussions for the child or perpetrator, concerns about confidentiality and limited awareness of reporting options, will also be crucial for ensuring an effective child protection response.

Friends were frequently the first port of call, highlighting young people’s roles in supporting their peers. While children should not bear the responsibility of child protection professionals, they may benefit from guidance that helps them support peers by connecting them to appropriate services or taking actions that minimize subsequent short-term harm. However, the burden of detection and response should not rest on children, families or civil society alone^69^.

Exploratory analyses identified sociocultural factors associated with children’s likelihood of disclosing experiences of technology-facilitated CSEA. Two key findings aligned with our preregistered theoretical predictions. First, enabling parental mediation was positively associated with disclosure. Safe family environments and open conversations about online safety may facilitate children’s willingness to report incidents without fear of judgement or shame^70^. Such family climates may foster trust that enables disclosure after harm occurs, or regular parent-child engagement may create opportunities for proactive communication that make children feel comfortable seeking help. However, our data cannot disentangle these pathways or establish directionality. Second, consistent with not knowing where to seek help being the most common barrier to disclosure, knowing where to seek help was associated with higher disclosure, particularly through informal channels. Developmentally appropriate help-seeking education may be a low-risk, scalable prevention target; however, this association was sensitive to model regularization, suggesting that relationships may vary across countries.

Finally, we observed more complex patterns related to normative attitudes toward gender and disclosure of technology-facilitated CSEA. Children who perceived premarital sex as more socially acceptable were more likely to disclose experiences of online sexual harms, although this association was limited to disclosure through informal channels such as friends or family. Although reduced stigma around sex and sexuality is theoretically associated with lower barriers to disclosure of sexual abuse, this predictor was not robust across all modelling specifications. Furthermore, inequitable gender attitudes were associated with higher disclosure rates in a subset of models. The relationship between beliefs about gender roles (for example, preservation of male honour and acceptance of violence against women) and children’s willingness to disclose remains complex and difficult to disentangle^60,71^. Addressing harmful gender norms is widely recognized as critical for violence prevention and may be relevant for technology-facilitated harms, particularly as shame or embarrassment around these conversations can prevent children from disclosing experiences of abuse altogether^60,72^. When these norms persist, perpetrators may take advantage of them to reduce the likelihood of being reported.

Our cross-sectional findings therefore identify plausible prevention and response levers^40^ but remain hypothesis-generating and warrant prospective evaluation. They highlight intervention priorities across multiple prevention levels: providing age-appropriate information on where to seek help from early childhood; supporting caregivers to sustain open, enabling communication that strengthens protective relationships; addressing stigma around sexual topics; and improving accessibility and responsiveness of formal services to meet children’s needs when harm occurs. As factors related to disclosure span interconnected domains (individual, family, cultural and systemic), effective prevention and response will probably require coordinated, multi-level approaches, rather than isolated, child- or technology-level interventions^73^.

These findings point to several important directions for future research. First, substantial heterogeneity observed between the 12 countries, combined with sensitivity to modelling techniques, highlights the need for culturally specific investigations into the mechanisms shaping disclosure. Participatory approaches that directly engage children with lived experience of technology-facilitated CSEA across rural, peri-urban and urban settings can generate region-specific insights that complement the population-level patterns identified^74^. Second, given the identification and retention challenges of traditional longitudinal cohorts in LMICs^75^, prospective longitudinal or diary-based designs that follow children over time may be more feasible for establishing temporal precedence and determining whether associated factors prevent harm, facilitate disclosure or both. Third, understanding platform-based disclosure mechanisms represents a critical priority^76^. Our analyses focus only on interpersonal disclosure pathways, but digital platforms can both facilitate and detect CSEA. Thus, understanding how children use platform-specific reporting tools and how technology companies respond will provide a more complete account of help-seeking in digital environments.

## Limitations

Our study has several important limitations. Our samples comprise only internet-using children; inferences are therefore limited to this subgroup. In high-connectivity countries (such as Malaysia and Thailand) this covers the majority of children, but substantial populations remain excluded in low-connectivity settings (such as Ethiopia). Cross-sectional data preclude causal inference; we cannot determine whether knowing where to seek help facilitated disclosure, or whether disclosed experiences increased awareness of support channels. Binary measurement (for example, help seeking, premarital sex) of key constructs provides only preliminary estimates. As such, all findings should be interpreted as identifying plausible associations that warrant future testing^77^ through ethically appropriate and feasible methodological approaches.

The eight demographic and sociocultural factors we investigated are not exhaustive^40^ and other important influences may exist. It is also likely that quantitative measurements alone cannot capture sociocultural influences at a sufficiently granular level to fully shed light on disclosure. Furthermore, our theoretical operationalization of technology-facilitated CSEA may blur the distinction between potential versus actual forms of CSEA (such as, sending and receiving sexual images)^18^. Data were collected in 2020–2021 before the widespread emergence of generative artificial intelligence tools. Our measures therefore do not capture artificial-intelligence-facilitated forms of CSEA. Disclosure is also a multifaceted and continuous process, which our measurement may risk oversimplifying by treating as a singular event^32^. As questions about barriers were administered only to non-disclosers, findings may under-represent barriers overcome by those who eventually disclosed. Finally, we acknowledge the positionality of our authorship team. None of the authors are originally from eastern and southern Africa or Southeast Asia. While the Disrupting Harm project was designed by teams with local representation and implemented in partnership with local stakeholders, future work would be strengthened by increasing equitable representation and diversifying authorship^78^.

## Conclusion

Looking ahead, as social media continues to reach remote parts of LMICs, large-scale, nationally representative mixed-methods studies will be key for building a global understanding of how digitally facilitated sexual crimes against children evolve. As boundaries between online and offline abuse become increasingly blurred^51^, the research agenda on digital harms must move beyond its predominant emphasis on screen time towards a more substantial focus on technology-facilitated CSEA.

Future research should build on this foundation by examining how perpetrators exploit children’s identities and life circumstances, including not only gender and age, but also socioeconomic status, poverty, disability, ethnicity, sexual orientation, migration and displacement, conflict exposure and other dimensions of marginalization. Closer collaboration and rapid data sharing among governments, law enforcement, technology companies, civil society and researchers are vital to mitigate these harms effectively. This is especially urgent as generative artificial intelligence and deepfakes are already being used to produce synthetic child sexual abuse material, expanding the scale of potential harm, and outpacing existing legal and safeguarding frameworks^79^.

While the Disrupting Harm project is finalizing research in 12 new countries across Latin America, Eastern Europe, Central Asia and the Middle East, continued scientific investment, funding and cross-national collaboration remain urgent to strengthen prevention and response systems to better protect children for an increasingly digital childhood. All children deserve dignity, equal rights and protection, regardless of where they live.

## Methods

### Ethical approval

The Disrupting Harm Survey was reviewed and approved by a global institutional review board (HML IRB Research and Ethics). Moreover, ethics approval was obtained from national or institutional ethics review bodies in each of the 12 participating countries. These included the National Commission for Science, Technology and Innovation (Kenya); Makerere University School of Public Health and the Uganda National Council of Science and Technology (Uganda); the Cambodia National Council for Children and the Ministry of Interior (Cambodia); the Ministry of Health, National Committee on Bioethics for Health (Mozambique); the Medical Research and Ethics Committee (Malaysia); the Health Research Ethics Committee, National Institute of Health Research and Development (Indonesia); the Ministry of Health and Social Services Ethical Review Board (Namibia); the Ministry of Labour, Invalids and Social Affairs (Vietnam); the Philippine Social Science Council Ethical Review Board (the Philippines); the Ethiopian Society of Sociologists, Social Workers and Anthropologists (Ethiopia); and multiple bodies in Tanzania: the National Institute of Medical Research, National Bureau of Statistics and the President’s Office–Regional Administration and Local Government, with permits from the Tanzania Commission for Science and Technology and additional approvals from the Zanzibar Health Research Institute. In Thailand, the study was reviewed by a special panel at Mahidol University’s Institute of Human Rights and Peace Studies, as no formal government ethics review process exists for social research.

### Dataset and participants

#### Disrupting Harm child survey

This survey was conducted between 2020 and 2021 across 12 countries in Eastern and Southern Africa and Southeast Asia and was designed to be representative of each country’s digitally connected population. A random-probability clustered-sample design was used to ensure all households had an equal chance of being sampled, while accounting for some countrywide variation. Ipsos Mori collected the data using a combination of face-to-face household interviews and online interviews (possibly due to the COVID-19 pandemic restrictions) to ensure maximum national coverage of the total population that had the probability of being included in the survey. The total sample size was approximately 1,000 children and caregivers in each country (Cambodia, *n* = 992; Ethiopia, *n* = 1,000; Indonesia, *n* = 995; Kenya, *n* = 1,014; Malaysia, *n* = 995; Mozambique, *n* = 999; Namibia, *n* = 994; the Philippines, *n* = 950; Tanzania, *n* = 996; Thailand, *n* = 967; Uganda, *n* = 1,016; Vietnam, *n* = 994), including some of the regions with the highest rates of violence against children in the world^13^ (Supplementary Table 1 for data collection timelines). The sampling strategy was further implemented in three stages: (1) 100 primary sampling units (PSUs) with a probability proportional to population size, region and urbanity were selected; (2) random samples of household units within each PSU were undertaken; and (3) children (and caregivers) within each eligible household unit were identified by a local enumerator. Field coverage varied slightly between countries owing to inaccessibility because of conflict-affected or remote areas. Kenya, Namibia, Cambodia and Mozambique had 100% coverage, while Indonesia had the lowest national coverage at 76% given its dispersed geography (Supplementary Fig. 1). Once PSUs were identified, the selection of household units was done using random walk methods, which included a random starting point, usually a landmark area (for example, church, mosque, bridge). To be included, children needed to have used the internet at least once in the past 3 months. Gender matching between interviewer and interviewee was encouraged and informed consent was provided by children and their caregivers.

We analysed data from the 11,912 children aged 12–17 years (full sample, 23,824 including parents), including 6,044 (51%) boys and 5,868 (49%) girls, 3,112 (26%) 12–13-year-old children, 3,954 (33%) 14–15-year-old children and 4,846 (40%) 16–17-year-old children (survey-weighted; unweighted counts in Supplementary Table 39). Gender-specific estimates were calculated among internet-using adolescents from the Disrupting Harm child survey. We could not construct comparable gender-specific population denominators because harmonised data on internet use by age (12–17 years) and gender are not consistently available across all study countries, including from the International Telecommunication Union (ITU) statistics (Supplementary Fig. 12).

The inclusion of rural and peri-urban children in LMICs is a particular strength of this dataset. 6,586 (55%) of the children were living in rural areas, 1,177 (10%) were living in peri-urban areas and 4,149 (35%) were living in urban areas (see Supplementary Table 32 for the full-weighted sample demographics, Supplementary Table 38 for children who experienced one or more forms of technology-facilitated CSEA, and Supplementary Table 39 for survey-weighted prevalence by demographic subgroup with unweighted counts and 95% CIs). A detailed pre-analysis plan was time-stamped and archived on the Open Science Framework (OSF) before data analysis (15 April 2022; https://osf.io/3tpqa/files/osfstorage?view_only=5f02ad5dcbbe4d2ab2b161ce9a00ccfb).

#### Disrupting Harm household survey (internet exposure)

In each country, the Disrupting Harm household survey (2020–2021) visited a nationally representative sample of approximately 1,500–10,000 households depending on connectivity. At each household, enumerators established whether any children aged 12–17 years lived in the household and, if so, whether those children used the internet (through any device and at any location). These exposure data were collected regardless of whether a child from the household completed the separate CSEA interview, thereby avoiding selection on child interview participation. Aggregating responses across sampled households yielded the national proportion of 12–17-year-old children who are internet users for each country. For validation, we compared these estimates with ITU youth internet-use indicators (typically ages 15–24 years, nearest available year; Supplementary Fig. 13). As ITU data differ in age band and reference period, we retained the Disrupting Harm household data as our primary exposure source (Supplementary Fig. 11).

### Measures

#### Overall instance of technology-facilitated CSEA

##### Measuring technology-facilitated CSEA

The prevalence of CSEA among internet-using children was measured using a composite variable capturing whether a child had experienced any form of technology facilitated CSEA within the past year, as reported during the 2020–2021 survey period (Supplementary Information 2.4 and Supplementary Tables 3 and 54). Rather than computing an average frequency, this variable provides a binary classification, flagging children who have been exposed to one or more types of sexual harms in the digital environment. The construction of this variable was implemented in two stages and combined responses to a battery of questions about (1) whether they had experienced one or more forms of CSEA; and (2) whether this occurred online (Supplementary Table 4).

First, to determine whether they had been exposed to technology-facilitated CSEA, children were asked nine screening items: “In the past year, how often have these things happened to you” (country-wise and Likert scale responses are shown in Supplementary Table 37). Their responses on a Likert scale were converted into binary indicators: a response of “never” was recoded as 0 (indicating did not experience), while responses ranging from ‘rarely’ to ‘very often’ were recoded as 1 (indicating did experience).

1. Someone made sexual comments about me (such as jokes, stories or comments about my body appearance or sexual activities) that made me feel uncomfortable.
2. Someone sent me sexual images I did not want.
3. I have been asked to talk about sex or sexual acts with someone when I did not want to.
4. I have been asked by someone to do something sexual when I did not want to.
5. I have been asked for a photo or video showing my private parts when I did not want to.
6. Someone offered me money or gifts in return for sexual images or videos.
7. Someone offered me money or gifts to meet them in person to do something sexual.
8. Someone shared sexual images of me without my consent.
9. Someone threatened or blackmailed me to engage in sexual activities.

Second, to estimate whether this happened online or offline, children were asked: “Thinking about the last time this happened, did it happen in any of these ways?”. The response items included multi-select options; that is, the children could select any of the following options: “in person”, “on social media” (for example, Facebook, YouTube, Snapchat, WhatsApp, Twitter)”, “in an online game”, “some other way”, “prefer not to say” or “don’t know” for each category of CSEA (Supplementary Tables 4, 47, 48 and 53). We coded a binary indicator as 1 if, for any of the nine harms, the child reported experiencing the harm and indicated that it occurred on social media or in an online game. This approach ensured that the final variable counted all those children who experienced sexual harms in the digital world.

##### Dual reporting approach

We report technology-facilitated CSEA prevalence in two ways. First, we estimate prevalence among internet-using children directly from the Disrupting Harm child survey. Second, we estimate prevalence in the total population aged 12–17 years by multiplying individual-level prevalence by internet penetration rates from the Disrupting Harm household survey. This approach assumes that only children with internet access can experience technology-facilitated sexual harms.

##### Uncertainty propagation

We propagated uncertainty from both survey components using Monte Carlo simulation to estimate 95% CIs for population-level prevalence and we drew 5,000 samples from each component’s sampling distribution on the logit scale, calculated the product for each iteration, and extracted the 2.5th and 97.5th percentiles of the resulting distribution (Supplementary Figs. 14 and 15). This approach treats the child and household surveys as statistically independent, justified by their separate sampling frames, child respondents and field operations. We calculated standard errors using effective sample sizes (Kish’s method; Supplementary Information 2.2) that account for survey weights. For the internet exposure component in countries where sample sizes were unavailable, we applied conservative default standard errors (3 percentage points for internet exposure and 5 percentage points for prevalence among internet users). These defaults affect only the width of the confidence intervals, not the point estimates.

Several considerations warrant caution when interpreting population-level estimates. First, internet exposure data were collected in 2020–2021. In rapidly digitalizing countries, exposure rates have probably increased substantially since then, meaning that our estimates do not represent current population burden. Second, the household measure captures regular internet use but may not capture occasional use outside the home (for example, at schools or community centres), leaving our prevalence estimates conservative. Third, our framework assumes children without internet access cannot experience technology-facilitated harms, an assumption that cannot be empirically validated with our data. Given these constraints and propagated uncertainty, population-level estimates should be interpreted as illustrative indicators of relative burden across countries rather than precise national estimates. A full list of caveats is provided in Supplementary Information 2.2.

#### Disclosure

To determine whether children disclosed online sexual incidents, they were asked “Who did you tell about what happened?” and presented with a list of multiple response options such as potential individuals, educators, and law enforcement organizations. They could also respond that they “hadn’t disclosed to anyone”, “didn’t know whether they had” or “preferred not to say” (multiple responses possible). As each disclosure target is a distinct behavioural decision with potentially different determinants and our exploratory research question is channel-specific, we derived three child-level binary indicators:

1. Any disclosure (told all): coded 1 if the child disclosed at least one CSEA type to any channel 0 otherwise.
2. Informal disclosure (told informal): coded 1 if the child disclosed any CSEA type to informal sources (parents/caregivers, siblings, friends, other adults, or other), 0 otherwise.
3. Formal disclosure (told formal): coded 1 if the child disclosed any CSEA type to formal authorities (teachers, police, helplines, social workers), 0 otherwise.

These categories are non-mutually exclusive (Supplementary Tables 40 and 41). Children who disclosed to both formal and informal channels are counted in both categories because each disclosure represents a distinct behavioural decision potentially influenced by different factors. Children selecting only “didn’t tell anyone” were counted as non-disclosures. Children could also select “prefer not to say,” or “don’t know”, which were counted as non-responses. As this question used this multi-select format, channel proportions can sum to more than 100% by design, as children who disclosed to multiple sources are counted in each applicable category. See Supplementary Fig. 25 for mutually exclusive categories of disclosure.

#### Barriers to disclosure

After the reporting item, adolescents who indicated “I did not tell anyone about it” for a given technology-facilitated CSEA harm received a multi-select barrier question (“Were any of the following reasons why you did not tell anyone about what happened?”). The unit of analysis is the non-disclosed incident at the CSEA-type level (that is, for each CSEA type where the child did not disclose).

For each barrier, we estimated the survey-weighted percentage of all non-disclosed incidents that cited that barrier. As the item is multi-select, an instance can contribute to multiple barrier categories, and percentages within a harm type need not sum to 100%. To assess whether barriers cluster, we constructed a co-occurrence matrix of weighted joint percentages among all non-disclosed incidents (Supplementary Fig. 26 and Supplementary Tables 55 and 56). Children could select any of the following multi-select barriers:

1. I did not know where to go or who to tell.
2. I felt embarrassed, ashamed or that it would be too emotionally difficult to tell.
3. I did not think anyone would believe me or understand my situation.
4. I was worried I would get in trouble if I told someone.
5. I felt that I did something wrong and did not want to tell.
6. I did not think it was serious enough to report.
7. I did not want the person who did this to get into trouble.
8. I feared it would cause trouble for me or my family.
9. I did not think anything would be done.
10. I did not know you could report these things.
11. My friends discouraged me from reporting.
12. My parents discouraged me from reporting.
13. I feared it would not be kept confidential.

#### Predictors of disclosure

Informed by the integrated child-centred model for violence prevention^51^, and Bronfenbrenner’s socioecological framework^52^, we situate disclosure within the broader context. These frameworks recognize that online violence intersects with a range of risk and protective factors, including individual, interpersonal, community, structural and institutional factors^51,80^. We preregistered a logistic regression with predictors across four conceptual levels: demographic (age and gender), family (enabling parental mediation of online activities), cultural (children’s attitudes towards sex) and protective factors (whether the child received sex education, had the knowledge of where to seek help, or had digital skills to report) (response options are shown in Supplementary Table 33, and country-wise responses are shown in Supplementary Tables 35 and 36).

##### Demographics

We included a continuous measure of age (centred at the mean for models) and binary measure for gender (treated as 1 = girl and 0 = boy in models). For regression models, gender was entered as a two-level factor with +0.5 contrast coding (boy = −0.5, girl = +0.5). We had no directional prediction regarding how individual factors such as age and gender may be related to disclosure. Previous research has found that girls are more frequently subjected to sexual abuse^57^, which might make them less likely to disclose given the increased prevalence. At the same time, boys may be less likely to report abuse owing to feelings of shame, potentially reducing their likelihood of disclosure^40^.

##### Family

To measure enabling parental mediation of online interactions, children answered the question “When you use the internet, how often does your parent/carer/guardian do any of these things?”, rating the frequency (from 1 = never to 5 = very often) of four specific behaviours that capture active support, help and supervision in navigating the online space. These behaviours included (1) encouraging the child to explore and learn things on the internet; (2) suggesting ways to use the internet safely; (3) helping the child when something bothers them on the internet; and (4) doing shared activities together on the internet. We took the item mean to obtain the enabling parental mediation measure (Cronbach’s Alpha across all parental mediation items was *α* = 0.78).

We hypothesized that enabling parental mediation would be associated with a  higher likelihood of disclosure. Research indicates there are different tiers of parental mediation^81^, including restrictive, active, supportive and co-playing mediation strategies, that buffer children’s exposure to online risks^59,81^. However, digital parenting styles may differ cross-culturally and diverse research on parental strategies remains lacking^82^. Yet some empirical evidence from Pakistan suggests that parents who are engaged in highly active mediation of internet safety are likely to mitigate adverse experiences online^58^.

##### Culture

Attitudes towards sex were measured with responses to the following statements: (1) “Having sex before marriage is acceptable”; (2) “Only men, not women, should decide when to have sex”; (3) “If someone insults a boy or man, he should defend his reputation with force if he needs to”; (4) “A woman should tolerate violence to keep her family together” (recoded: 1 = yes, 0 = no). These were then bifurcated into two separate variables; the first statement measured attitudes towards premarital sex, a proxy for measuring positive gender norms, while the latter three statements were conceptually distinct and combined to measure inequitable gender attitudes. We analysed two predictors: premarital sex (item 1) as a separate binary variable (0.5 centred for regression) to account for the positive gender norm, and an inequitable gender-attitudes index equal to the sum of items (items 2 to 4) (range 0–3; higher = more inequitable).

We hypothesized that the combined gender norm measure (1–4 items) may be related to lower likelihood of disclosure. Decades of research on violence against women have shown that gender inequality is often linked to the acceptance of intimate partner violence and gender-based violence^71,72,83,84^. In some countries, patriarchal views normalize violence against women^85^, for example, a study using high-quality data from the Gender and Adolescence: Global Evidence^86^ in Ethiopia found that community norms, more than household attitudes, predicted violence against children^71^.

##### Protective level

We included three factors that could theoretically be related to the likelihood of disclosure: sex education, digital skills to report and the child’s knowledge of how to seek help. Sex education was measured by children answering if they had “received any sex education” (the term ‘sex education’ was changed to ‘reproductive health’ in Cambodia) (binary variable: 1 = yes, 0 = no). A child’s knowledge of how to seek help (help-seeking knowledge) was measured by children answering whether they knew where to get help if “you or a friend experience sexual assault or sexual harassment” (binary variable: 1 = yes, 0 = no). Digital skills to report was measured by children answering if they knew how to “report harmful content”, which was measured on a scale of 1 to 4 (1 = not at all true for me, 4 = very true for me). Note that the digital skills questions measured a total of eight skills and competencies related to internet use, including information, operational, social and creative skills. However, for this analysis, we selected only the core social skill related to disclosure.

We hypothesized that sex education, digital skills and a child’s knowledge of how to seek help when they or a friend are assaulted would be positively associated with disclosure. Previous research has identified specific protective factors, such as sex education and digital skills that are linked to children’s attitudes toward online safety^21,62^. Research has also underscored the importance of online safety resources in directly addressing the complexities of child abuse^50,87^.

### Inclusion criteria

In the Disrupting Harm survey, we first adjusted the country’s sample size to reflect the weights, dropping 707 responses and then omitting observations that we had planned to exclude, such as non-responders (don’t know or prefer not to say) and variables with high missingness (Supplementary Table 35).

### Analyses

Using Bayesian multilevel logistic regression models, we modelled our binary outcomes (1) whether a child experienced technology-facilitated CSEA (Supplementary Tables 5 and 6 and Supplementary Fig. 20); and (2) whether they disclosed the experience, where individual children formed the lower level and countries the upper level. First, we analysed whether age and gender predicted the likelihood of experiencing one or more types of technology-facilitated CSEA (1 = did experience, 0 = did not experience). We modelled this outcome as Bernoulli distributed with a logit link function:

$$\begin{array}{c}\mathrm{logit}(\Pr ({\mathrm{CSEA}}_{{ij}}=1))={\beta }_{0}+{u}_{0j}+({\beta }_{1}+{u}_{1j}){\mathrm{Gender}}_{{ij}}\\ \,+\,({\beta }_{2}+{u}_{2j}){\mathrm{Age}}_{{ij}}+({\beta }_{3}+{u}_{3j}){\mathrm{Gender}}_{{ij}}{\mathrm{Age}}_{{ij}}\end{array}$$

where *β*_0_, *β*_1_, *β*_2_, *β*_3_ represent the fixed effects for the intercept, gender, age and their interaction respectively, Gender_*ij*_ and Age_*ij*_ represent the gender and age of child *i* in country *j* and u_0*j*_ represents the random effect of belonging to country *j*, *u*_1*j*,_ *u*_2*j*_ and *u*_3*j*_ represent the random slopes of gender, age and their interaction in the model. We modelled the country-specific parameters as multivariate normally distributed.

As logit-scale interaction coefficients can be hard to interpret^88^, we report predicted probabilities and AMEs for gender, age and their interaction. Uncertainty reflects posterior draws and is summarized with 95% credible intervals. AMEs are averaged over the observed country-level random effects, reflecting the average effect across the 12 study countries in the sample. To evaluate evidence for the null, we conducted Bayesian equivalence tests by calculating the proportion of the posterior distribution falling within a ROPE defined as odds ratios between 0.67 and 1.50, a range conventionally considered negligible^89,90^. We also compared additive and gender and age interaction specifications using Pareto-smoothed importance-sampling leave-one-out cross-validation (PSIS-LOO)^91^ (Supplementary Fig. 22). The difference in expected log predictive density (ΔELPD) was −0.9 (s.e.m. = 1.9), indicating no meaningful improvement in out-of-sample predictive performance from including the interaction.

We further examined whether prevalence of technology-facilitated CSEA differed across urban, peri-urban and rural settings, while accounting for gender, age and cross-country heterogeneity (Supplementary Tables 8 and 9). We report results as AMEs on the probability scale, population-averaged over country random effects. This model preserves an additive fixed effect for degree of urbanization (that is, the urban, peri-urban and rural contrasts are adjusted for gender and age), while allowing gender and age effects (and their interaction) to vary by country as random slopes. We chose not to model degree of urbanization with random effects because each country contributes only three categories, making random-effects estimation unstable and difficult to interpret.

We next modelled disclosure (1 = did disclose, 0 = did not disclose) using the same Bayesian multilevel logistic regression framework, with the following predictors: gender, age, attitudes towards premarital sex, inequitable gender attitudes, enabling parental mediation, digital skills to report, sex education and knowing where to seek help if you or a friend were sexually assaulted. We assessed multicollinearity using variance inflation factors on the complete-case sample; all variance inflation factors values were below 3 (Supplementary Table 14). Weighted Pearson correlations among all predictors were examined as a further check for multicollinearity (Supplementary Fig. 27). This model was specified as:

$$\mathrm{logit}(\Pr ({\mathrm{Disclosure}}_{{ij}}=1))={\beta }_{0}+{u}_{0j}+\mathop{\sum }\limits_{p=1}^{P}({\beta }_{p}+{u}_{{pj}}){X}_{{pij}}$$

where *β*_0_ is the fixed intercept, *u*_0*j*_ is the random intercept for country* j*, *P* is the total number of predictors, *β*_*p*_ is the fixed effect for predictor *p*, *u*_*pj*_ is the country-specific random slope for predictor *p*, and *X*_*pij*_ is the value of predictor *p* for child* i* in country *j*.

To reduce the risk of overfitting, we applied shrinkage through a horseshoe prior on the fixed effect coefficients to regularize estimation, specifying three degrees of freedom and a global scale parameter of 0.5. The horseshoe prior induces adaptive shrinkage and is a recommended Bayesian alternative to LASSO for variable selection^92^. All other parameters retained brms default priors: a Student-*t*(3, 0, 2.5) prior for the intercept, half-Student-*t*(3, 0, 2.5) priors for random effect standard deviations and an LKJ(1) prior for the random effects correlation matrix^93,94^.

To assess whether predictor–disclosure associations varied by country, we compared a baseline additive model (all predictors fixed, random intercepts for country) to a series of models in which one predictor at a time had a random slope by country (for example, inequitable gender attitudes allowed to vary by country) (Supplementary Tables 16–19 and Supplementary Fig. 28). We used PSIS-LOO to compute ELPD and ΔELPD (s.e.) relative to the baseline model^91^. ΔELPD values ranged from −3.1 to 0.0 (s.e.: 3.4-4.3), indicating no supported improvement from adding any single random slope (Supplementary Table 20 and Supplementary Fig. 29). Bayesian *R*^2^ for the complete-case model is reported in Supplementary Table 15. Posterior correlations among fixed-effect parameter estimates for all disclosure models are reported in Supplementary Figs. 30–32.

We used Bayesian methods for all model estimation because they are well suited to estimating (co)variance parameters in multilevel models^93^. We estimated all models using Markov chain Monte Carlo (MCMC) sampling, specifying 4 chains of 2,000 iterations with 1,000 warm-up iterations (4,000 post warm-up draws per imputation). We set the target acceptance probability (delta) to 0.99 to facilitate convergence. We report all posterior mean or median summaries, posterior probabilities of direction and the credible intervals between the 2.5th and 97.5th percentiles. To evaluate convergence, we used a conservative approach tailored for multiply imputed models. We computed *R*-hat separately for each imputed dataset and report the highest value observed across imputations (Supplementary Table 31). We also inspected the number of divergent transitions and visually inspected trace plots, *R*-hat histograms and stratified posterior predictive checks (Supplementary Figs. 34–38).

All analyses were adjusted using weights for random probability samples and included three stages: (1) inverse probability weights to account for the variation in design; (2) non-response weights to reduce bias; and (3) post-stratification weights to account for differences between the sample and target internet-using population distributions. All reported estimates and counts are survey-weighted (unless mentioned otherwise). Further details about our sample weights and design are provided in Supplementary Table 2. Missing data ranged from 0.2% to 33.8% across variables (Supplementary Table 11). All missing data for the independent variables were imputed using the multiple imputation by chained equations (MICE) method and the mice package in R^95^. MICE was used as it accommodates both continuous and categorical variables flexibly and is well suited to the complexity of the dataset. We ran 30 imputations of the analysis and pooled results across all datasets to ensure robust estimation. All main models were estimated using the brms^93^ R package for Bayesian estimation. All graphs were created using ggplot2^96^ and ggdist^97^. All diagnostics were checked using the Bayesplot^98^ package.

### Deviation from the pre-registration

We deviated from the pre-registrated analysis plan in several ways. First, although we preregistered one confirmatory logistic regression model (our preregistered hypothesis is shown in the ‘Predictors of disclosure’ section) and four exploratory models (interaction, random intercepts, comparing formal versus informal channels and regularized regression), we did not interpret the GLM as the test of our confirmatory analysis. We extended the preregistered exploratory random-intercept model into a Bayesian random intercepts and slopes model. This allowed us to (1) factor in the heterogeneity in the relationship between the set of predictors and disclosure; (2) provide more nuanced uncertainty estimation; and (3) reflect the empirical expectation that effects will not be homogeneous across these diverse countries in Asia and Africa. Owing to this expected heterogeneity, we judged this extension to be theoretically and methodologically appropriate. All preregistered and exploratory models with missing data and imputed models are reported in Supplementary Tables 10, 12, 13, 21–23 and 25–30 and Supplementary Fig. 33. Second, given that conceptual frameworks for technology-facilitated CSEA are still emerging and considering the lack of standardization in how such harms are measured and categorized^19^, we analysed each reported type of sexual harm (that occurred on social media or in an online game) separately. We had initially divided the nine measures into three categories (the previous categorization is shown in Supplementary Fig. 16). However, it was challenging to clearly delineate the boundaries between possible and grave instances of forms of sexual exploitation and abuse, and we wanted to avoid conflating these categories or introducing measurement error. Our final terminology was informed by the updated guidelines by the Working Group for the Revision of the Terminology Guidelines for the Protection of Children from Sexual Exploitation and Sexual Abuse (the Luxembourg Guidelines)^18^.

Third, we extended the scope of our analysis to add a core model to estimate the probability of children experiencing technology-facilitated CSEA and unpack the associations with key demographic factors. Fourth, we excluded the perpetrator’s identity (for example, unknown, known romantic, known family, known other) from the main model due to substantial conceptual and reporting overlap between categories (that is, many children reported multiple perpetrators) (Supplementary Fig. 19 and Supplementary Tables 43 and 44). This was initially preregistered as an interpersonal predictor. Instead, we reclassified enabling parental mediation, which was also preregistered after revisiting the literature as an interpersonal (family-level) predictor. Fifth, we had preregistered to run an exploratory analysis using the LASSO regularized regression. However, the underlying brms package (v.2.19.2) no longer supports the LASSO prior, and we use a Horseshoe prior instead. Finally, we did not implement backward stepwise selection or bootstrapping; instead, model comparison was conducted by PSIS-LOO cross-validation^91^ and uncertainty was quantified through posterior distributions (Supplementary Tables 20 and 24 and Supplementary Figs. 22 and 29).

### Reporting summary

Further information on research design is available in the Nature Portfolio Reporting Summary linked to this article.

## Online content

Any methods, additional references, Nature Portfolio reporting summaries, source data, extended data, supplementary information, acknowledgements, peer review information; details of author contributions and competing interests; and statements of data and code availability are available at 10.1038/s41586-026-10525-4.

## Supplementary information

Supplementary Information Supplementary Information 1: fieldwork. Supplementary Information 2: technology-facilitated CSEA. Supplementary Information 3: disclosure analysis. Supplementary Information 4: disclosure inferential analysis: complete case. Supplementary Information 5: disclosure inferential analysis: multiple imputations. Supplementary Information 6: model checking. Supplementary Information 7: descriptives summaries. Supplementary Information 8: references.

Reporting Summary

Peer Review File

## Data Availability

The Disrupting Harm survey data used in this study were shared with the research team (S.G. and A.O.) under a data-sharing agreement with UNICEF Office of Strategy and Evidence – Innocenti. Owing to the sensitive nature of the data, involving child-level reports of sexual exploitation and abuse, they cannot be made publicly available.

## Code availability

All analyses were conducted in R (v.4.4.1)^99^. All pre-processing and modelling code, including rendered HTML outputs showing full code and results, is publicly available at GitHub (https://github.com/sghai9/technology-mediation-csea) and archived on Zenodo (10.5281/zenodo.20050190)^100^.
