# Supplementary material for: Technology mediation in child sexual exploitation and abuse in Africa and Asia
Source: Nature. 2026 May 27;654(8118):408–18. doi: 10.1038/s41586-026-10525-4 (PMC13253325; doi:10.1038/s41586-026-10525-4)
Supplement: Supplementary file 1 — Supplementary Information 1: fieldwork. Supplementary Information 2: technology-facilitated CSEA. Supplementary Information 3: disclosure analysis. Supplementary Information 4: disclosure inferential analysis: complete case. Supplementary Information 5: disclosure inferential analysis: multiple imputations. Supplementary Information 6: model checking. Supplementary Information 7: descriptives summaries. Supplementary Information 8: references. [file 41586_2026_10525_MOESM1_ESM.pdf]

nature portfolio

https://doi.org/10.1038/s41586-026-10525-4

Supplementary information

Technology mediation in child sexual exploitation and abuse in Africa and Asia

In the format provided by the authors and unedited

Supplementary Information for: Technology Mediation in Child Sexual Exploitation and Abuse in Africa and Asia

Table of contents

1 Fieldwork **3**
- 1.1 Data Collection . . . 3
- 1.2 Fieldwork Coverage . . . 3
- 1.3 Survey Weighting Procedures . . . 5

2 Technology-facilitated CSEA **7**
- 2.1 Prevalence of Technology-facilitated CSEA Among All Children . . . 12
- 2.2 Uncertainty Propagation . . . 17
- 2.3 Categorisation of Online Child Sexual Abuse and Exploitation (OCSEA) . . . 19
- 2.4 Measurement of Technology-Facilitated CSEA . . . 20
- 2.5 Location of Technology-Facilitated CSEA Incidents . . . 21
- 2.6 Heatplot (Location of Technology-Facilitated CSEA Incidents) . . . 23
- 2.7 Online Platforms Involved in Technology-Facilitated CSEA Incidents . . . 24
- 2.8 Perpetrator Identity in Technology-Facilitated CSEA Incidents . . . 25
- 2.9 Demographic Predictors of Technology-facilitated CSEA . . . 26
- 2.10 Leave-One-Out Cross-Validation (Demographic models) . . . 30
- 2.11 Degree of Urbanisation Differences . . . 31

3 Disclosure Analysis **35**
- 3.1 Disclosure by Types of Technology-facilitated CSEA . . . 35
- 3.2 Mutually Exclusive Categories of Disclosure . . . 36
- 3.3 Disclosure Barriers Full Co-occurrence Matrix . . . 37
- 3.4 Disclosure Inferential Analysis Overview . . . 39

4 Disclosure Inferential Analysis: Complete Case **40**
- 4.1 Investigate Missingness . . . 40
- 4.2 Pre-Registered Confirmatory Analysis: Logistic Regression . . . 40
- 4.3 Pre-Registered Exploratory Analysis: Random Intercepts Model . . . 41
- 4.4 Multicollinearity Diagnostics . . . 41
- 4.5 Channel Specific Regressions . . . 45
- 4.6 Forest Plot for Channel Specific Predictors . . . 49
- 4.7 Sensitivity Analysis: Predictors Varying by Random Slopes . . . 50

5 Disclosure Inferential Analysis: Multiple Imputations **52**
- 5.1 Pre-Registered Confirmatory Analysis: Logistic Regression Model . . . 52
- 5.2 Pre-registered Exploratory Analysis: Random Intercepts Model . . . 52

5.3 Exploratory Analysis (Not Pre-Registered): Random Intercepts and Slopes Model (Main Reported Analysis) . . . 53
5.4 Correlations Among Predictors in the Exploratory Model . . . 54
5.5 Pre-registered Exploratory Analysis: Random Intercepts, Slopes and Age-Gender Interactions . . . 58
5.6 Pre-registered Exploratory Analysis: Regularised Horseshoe Regression . . . 61
5.7 Posterior Probability of Direction . . . 62
5.8 Country-specific Coefficients Predicting Disclosure . . . 64

6 Model Checking **71**
6.1 R-hat Diagnostics . . . 71
6.2 Graphical Diagnostics . . . 72

7 Descriptives Summaries **77**
7.1 Demographic Characteristics . . . 78
7.2 Coding Scheme . . . 79
7.3 Role-based Disclosure . . . 80
7.4 Weighted Descriptive Statistics – Africa Subsample . . . 81
7.5 Weighted Descriptive Statistics – Asia Subsample . . . 84
7.6 Likert-Scale Responses for Technology-Facilitated CSEA . . . 89
7.7 Demographics of Technology-Facilitated CSEA . . . 92
7.8 Demographic-Specific Prevalence . . . 93
7.9 Disclosure Channel Rates by Country . . . 94
7.10 Disclosure by Type of Technology-Facilitated CSEA . . . 103
7.11 Perpetrator Identity . . . 104
7.12 Perpetrator Type Combinations . . . 106
7.13 Barriers to Disclosure . . . 107
7.14 Online vs Offline Context . . . 109
7.15 Social Media Platforms . . . 110
7.16 Overall Prevalence by Type . . . 112
7.17 Overall Prevalence by Type - Extended . . . 113
7.18 Prevalence by Country . . . 114
7.19 Technology-Facilitated CSEA Outcomes by Country . . . 115
7.20 Location of CSEA . . . 120
7.21 Operationalisations of Technology-Facilitated CSEA by Country . . . 121
7.22 Multiplicity of Disclosure Barriers . . . 122

8 References **123**

1 Fieldwork

1.1 Data Collection

Table 1: Fieldwork dates and sample sizes for the Disrupting Harm child survey across 12 countries in Africa and Asia (2020–2021). Columns report fieldwork start and end dates, target sample size (n = 1,000 per country), achieved unweighted sample size, and achieved sample size after application of survey weights.

Country	Fieldwork Start Date	Fieldwork End Date	Target Sample Size	Achieved Sample Size	Sample Size (with Survey Weights)
Ethiopia	21/12/2020	19/01/2021	1000	1021	1000
Kenya	21/12/2020	19/01/2021	1000	1016	1014
Mozambique	South: 17/02/2021 Central and North: 13/05/2021	30/07/2021	1000	1111	999
Namibia	21/12/2020	28/02/2021	1000	1000	994
Tanzania	21/12/2020	19/01/2021	1000	1000	996
Uganda	04/01/2020	19/01/2021	1000	1020	1016
Cambodia	10/11/2020	31/12/2020	1000	1007	992
Indonesia	26/11/2020	28/02/2021	1000	1012	995
Malaysia	12/04/2021	05/11/2021	1000	1009	955
Philippines	11/01/2021	15/04/2021	1000	975	950
Thailand	21/11/2020	23/02/2021	1000	1000	967
Vietnam	21/11/2020	22/02/2021	1000	998	994

1.2 Fieldwork Coverage

We describe the fieldwork coverage in each country conducted by **Ipsos Mori**. The total proportion of the population that had a chance of being included in the survey sample.

Reasons for exclusion are detailed and provided by Ipsos Mori and the UNICEF team.

In **Ethiopia**, entire region of Tigray had to be removed from the PSU list, along with selected conflict-affected zones within the following regions: Afar, Amhara, Dire Dawa, Oromia, and Somali. In **Indonesia** given the dispersed geography of the country the following provinces were excluded from frame: Aceh, Bali, Bengkulu, Daerah Istimewa Yogyakarta, Gorontalo, Jambi, Kalimantan Selatan, Kalimantan Tengah, Kalimantan Utara, Kepulauan Bangka Belitung, Kepulauan Riau, Maluku Utara, Nusa Tenggara Barat, Nusa Tenggara Timur, Papua Barat, Riau, Sulawesi Barat, Sulawesi Tengah, Sulawesi Tenggara, Sumatra Barat, Sumatra Utara. In **Uganda** conflict areas and areas occupied by the military were excluded, such as in southwestern, West Nile, Acholi and Karamoja sub-regions; and areas that are close to refugee settlements. In **Malaysia**, rural areas that are very remote/isolated were excluded, primarily in East Malaysia (e.g., Borneo). In **Philippines**, autonomous Region in Muslim Mindanao (ARMM) excluded for security reasons. In **Thailand**, three southern provinces (Narathiwat, Yala, Pattani) excluded for security reasons. In **Tanzania**, the regions of Tanga (Pangani), Pemba (Kisiwa panzi, Kojani, Makohongo, Fundo, Shamiani), Unguja (Tumbatu, Uzi), Kigoma (Mwamgongo, Kagunga), Kagera (goziba), Ngorongoro (Pinyinyi, Naiyobi, Endureni), Longido (Geraimeurogoi, Geraibomba) were not included due to security threats and inaccessibility.

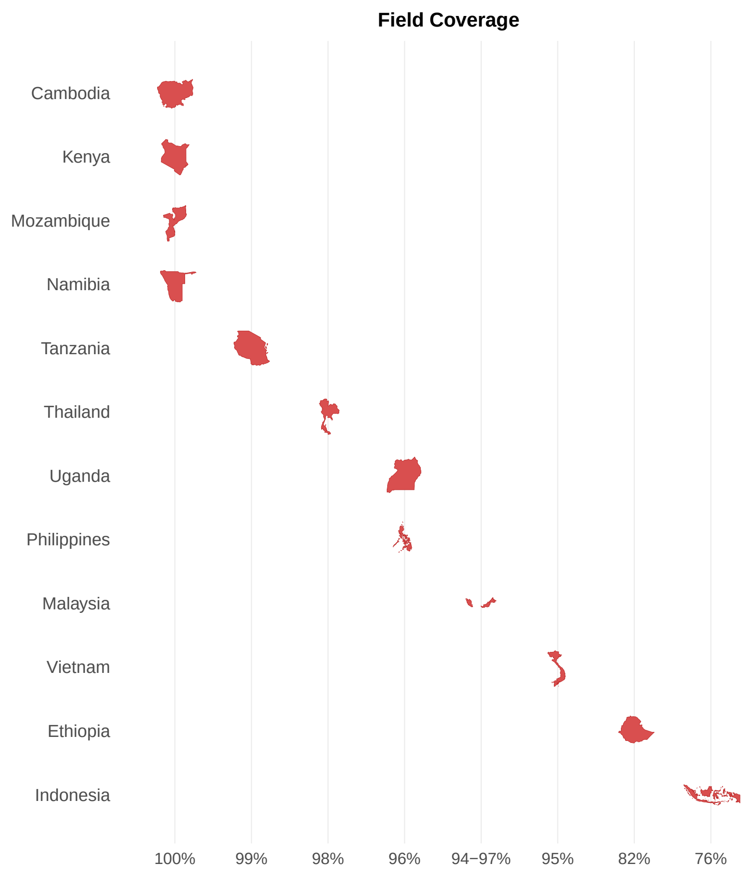

Figure 1: Percentages indicate the proportion of the national sampling frame covered in each country. Coverage was 100% in Kenya, Mozambique, Namibia, and Cambodia. Minor exclusions applied in Tanzania (99%), Uganda (96%), Thailand (98%), Vietnam (95%), Philippines (96%), and Malaysia (94–97%), primarily due to security concerns, military occupation, refugee settlement proximity, or remote/inaccessible terrain. Ethiopia's coverage was 82%, reflecting the removal of the Tigray region and selected conflict-affected zones in Afar, Amhara, Dire Dawa, Oromia, and Somali prior to PSU selection. Indonesia had the lowest coverage (76%), with 21 provinces excluded owing to the country's dispersed geography.Basemap boundaries: Natural Earth

1.3 Survey Weighting Procedures

Survey weights were developed and applied by **Ipsos Mori** to ensure that the sample of digitally connected children aged 12–17 was representative of the internet-using population in each country. We detail the weighting provided by Ipsos Mori, which involved three main steps:

1.3.1 A) Design Weights

First, design weights accounted for unequal selection probabilities across the three sampling stages: PSUs (selected via PPS), households within PSUs (random walk), and children within households (one randomly selected per household). The combined design weight was the product of these three stage-level weights.

- **Primary Sampling Units (PSUs):** Selected using probability proportional to size (PPS):
  dw_PSU = 1 / p_PSU
- **Households within PSUs:** Selected via a random walk method:
  dw_unit = 1 / (n_unit / Size_PSU)
- **Children within households:** One child was randomly selected:
  dw_child = 1 / p_child, where *p_child = 1 / number of eligible children*

The full design weight for each respondent was calculated as:
dw = dw_PSU × dw_unit × dw_child

1.3.2 B) Non-Response Adjustment

Second, design weights were adjusted for differential non-response among eligible children.

nr_weight = total number of eligible children / number of completed interviews

1.3.3 C) Post-Stratification Adjustment

Third, post-stratification weighting targets were constructed by combining UNDP (2020) population totals by age group with internet use rates estimated from the ECS household screening data. The resulting targets represent the estimated size of the internet-using child population aged 12–17 in each country. Weights were trimmed at each stage to reduce the influence of extreme values (PSU/household and non-response weights capped at the 97.5th percentile; individual and post-stratification weights capped at the 99th percentile), then rescaled to a mean of 1.0 (*wgt_scaled*). As all analyses reported here use proportions and prevalence rates rather than population totals, wgt_scaled was used throughout, including for cross-country comparisons. Design effects were calculated to assess the impact of weighting on the effective sample size, ranging from **1.16 (Kenya)** to **1.45 (Ethiopia)**.All descriptive and inferential statistics were conducted using these scaled weights.

Table 2: Design effects by country for the Disrupting Harm child survey (Ipsos MORI, 2020–2021). The ECS design weight (dw_PSU_unit) reflects the combined effect of unequal selection probabilities across primary sampling units and households. The interview analysis weight (wgt_scaled) reflects the full post-stratification weight after non-response adjustment, trimming, and rescaling to a mean of 1.0. Values greater than 1.0 indicate that weighting reduces the effective sample size relative to a simple random sample. All analyses reported in this manuscript use wgt_scaled

Country	ECS design weight (dw_PSU_unit)	Interview weight (wgt_scaled)
Ethiopia	1.2	1.4
Kenya	1.1	1.2
Mozambique	1.2	1.4

Country	ECS design weight (dw_PSU_unit)	Interview weight (wgt_scaled)
Namibia	1.2	1.4
Tanzania	1.1	1.3
Uganda	1.1	1.4
Cambodia	1.3	1.2
Indonesia	1.3	1.2
Malaysia	1.3	1.4
Philippines	1.2	1.3
Thailand	1.2	1.2
Vietnam	1.2	1.3

2 Technology-facilitated CSEA

Survey-weighted prevalence of nine forms of technology-facilitated child sexual exploitation and abuse (CSEA) among internet-using children aged 12–17 across 12 countries in Africa and Asia (2020–2021). Each map displays the percentage of children who reported experiencing a specific form of CSEA via social media or an online gaming platform. Colour reflects prevalence on a shared scale across all nine maps, ranging from dark purple (low) to yellow (high), enabling direct visual comparison across harm types.

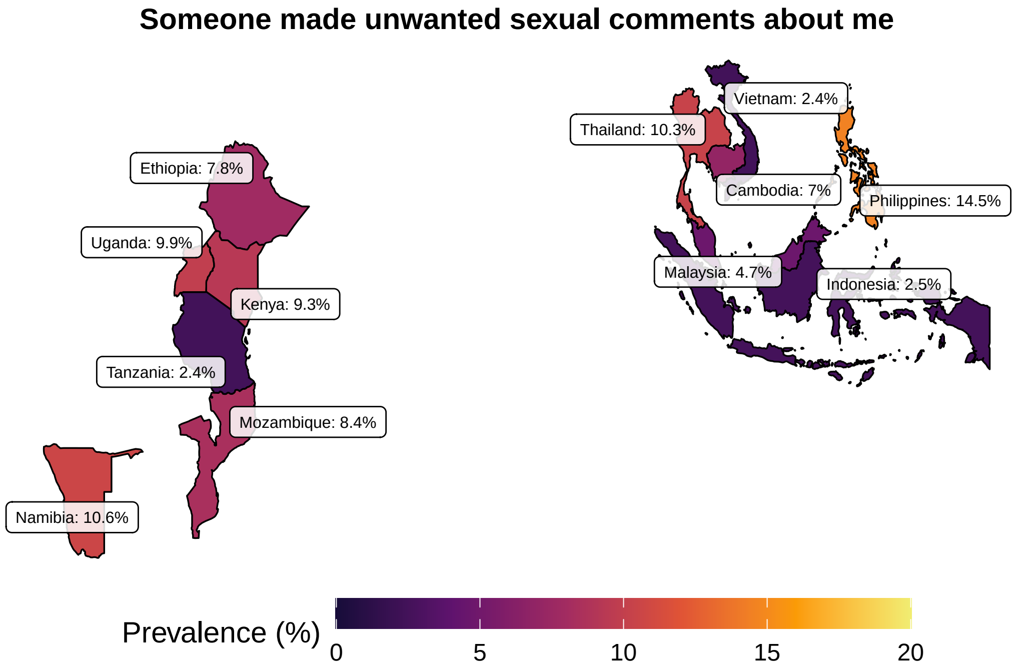

Figure 2: Survey-weighted prevalence of nine forms of technology-facilitated CSEA among internet-using children aged 12–17 across 12 countries in Africa and Asia (2020–2021). Basemap boundaries: Natural Earth (naturalearthdata.com; public domain).

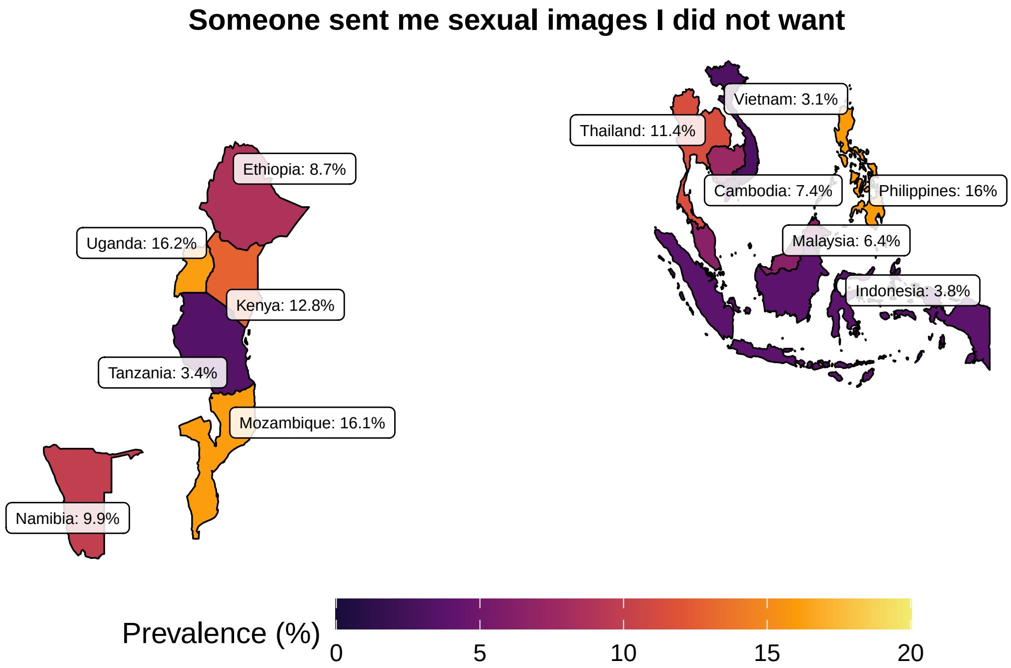

Figure 3: Survey-weighted prevalence of nine forms of technology-facilitated CSEA among internet-using children aged 12–17 across 12 countries in Africa and Asia (2020–2021). Basemap boundaries: Natural Earth (naturalearthdata.com; public domain).

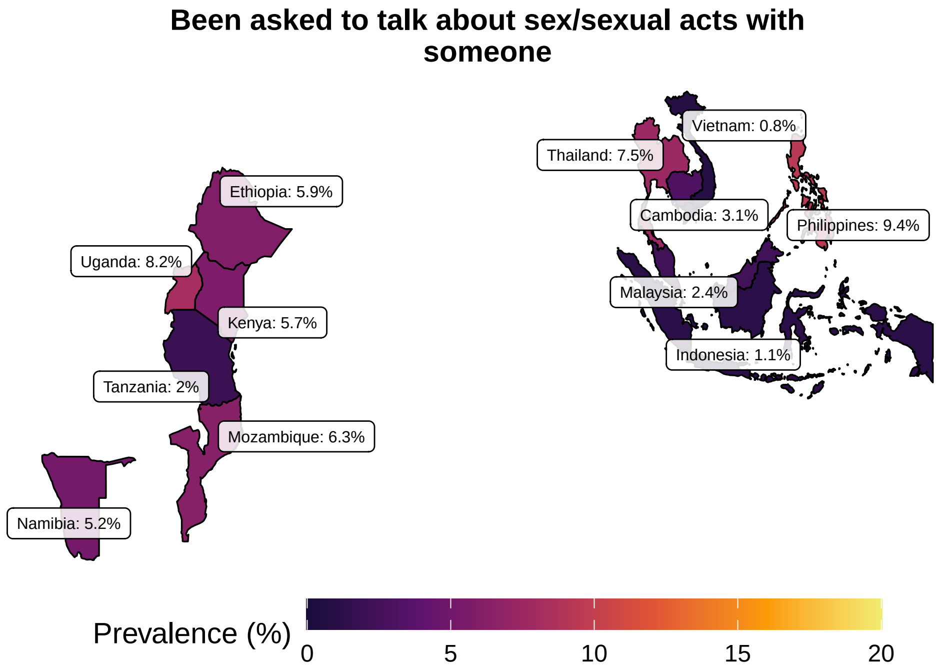

Figure 4: Survey-weighted prevalence of nine forms of technology-facilitated CSEA among internet-using children aged 12–17 across 12 countries in Africa and Asia (2020–2021). Basemap boundaries: Natural Earth (naturalearthdata.com; public domain).

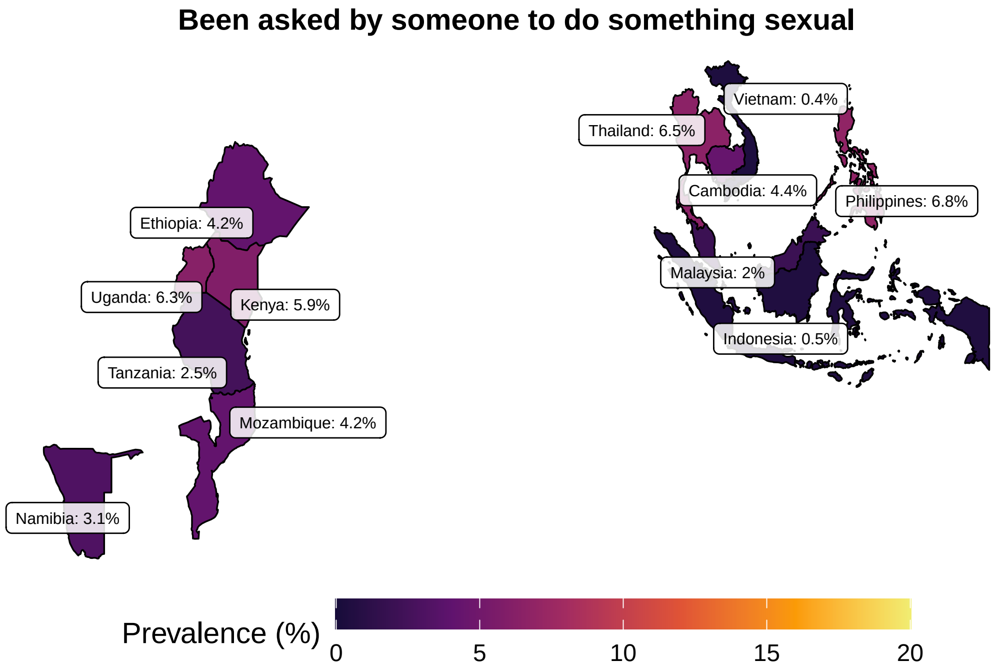

Figure 5: Survey-weighted prevalence of nine forms of technology-facilitated CSEA among internet-using children aged 12–17 across 12 countries in Africa and Asia (2020–2021). Basemap boundaries: Natural Earth (naturalearthdata.com; public domain).

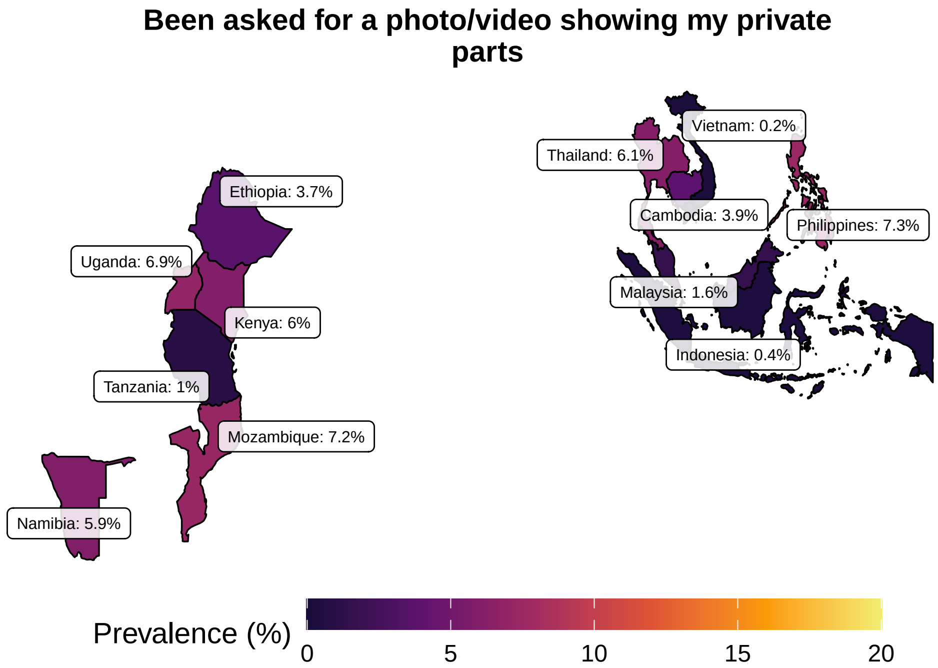

Figure 6: Survey-weighted prevalence of nine forms of technology-facilitated CSEA among internet-using children aged 12–17 across 12 countries in Africa and Asia (2020–2021). Basemap boundaries: Natural Earth (naturalearthdata.com; public domain).

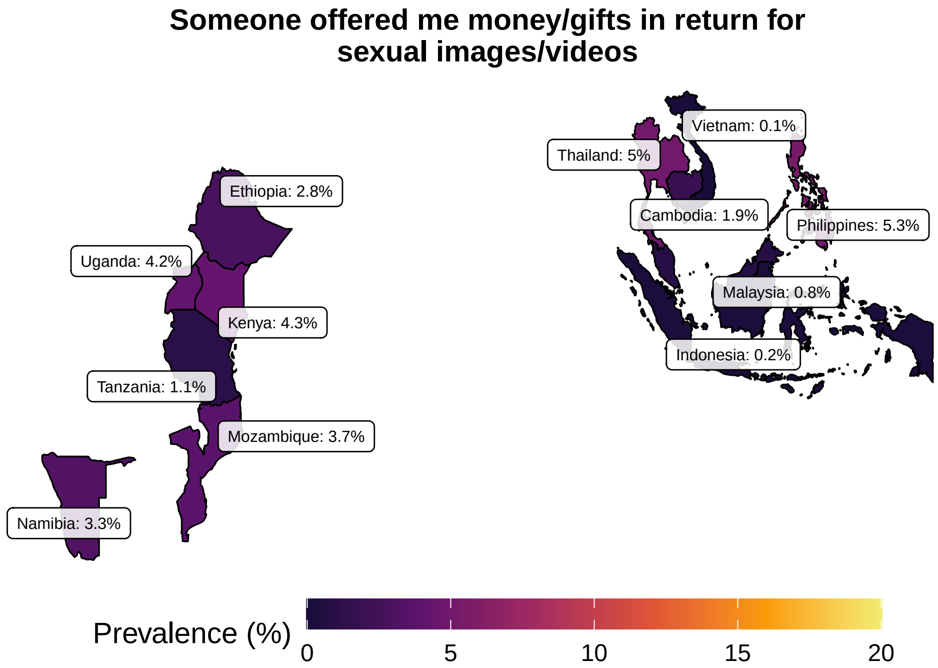

Figure 7: Survey-weighted prevalence of nine forms of technology-facilitated CSEA among internet-using children aged 12–17 across 12 countries in Africa and Asia (2020–2021). Basemap boundaries: Natural Earth (naturalearthdata.com; public domain).

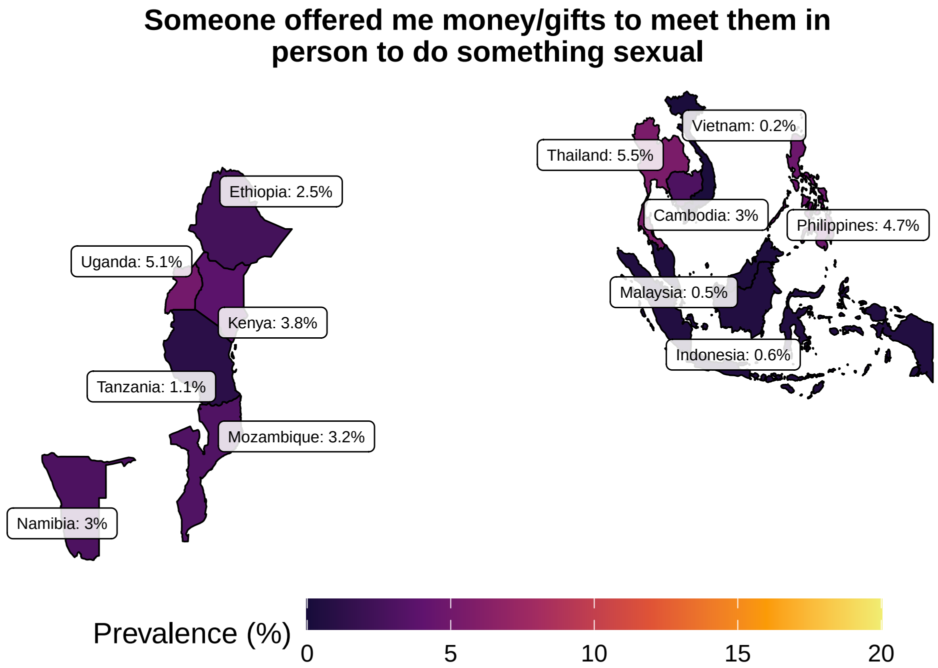

Figure 8: Survey-weighted prevalence of nine forms of technology-facilitated CSEA among internet-using children aged 12–17 across 12 countries in Africa and Asia (2020–2021). Basemap boundaries: Natural Earth (naturalearthdata.com; public domain).

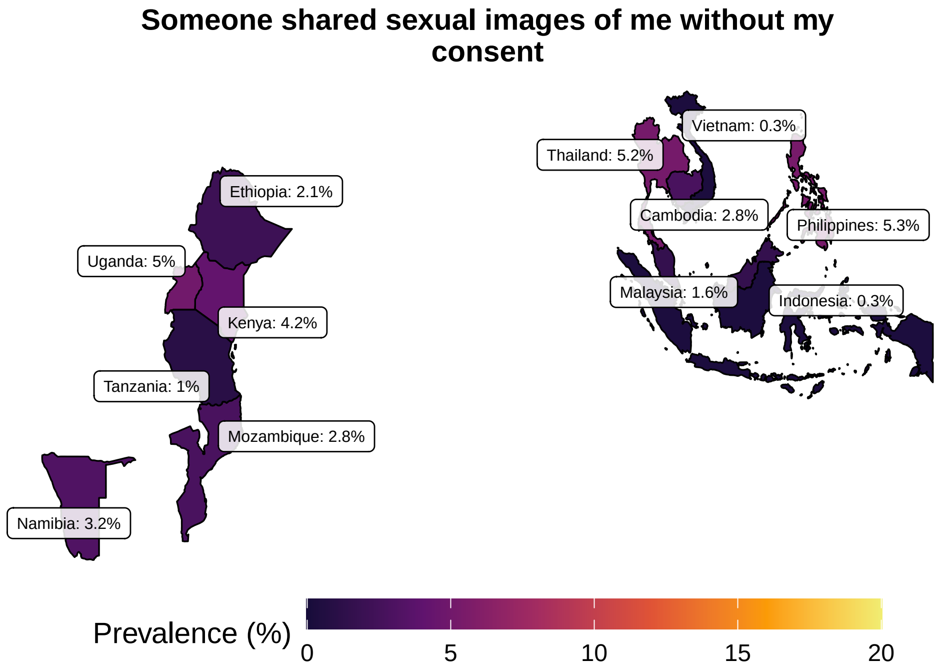

Figure 9: Survey-weighted prevalence of nine forms of technology-facilitated CSEA among internet-using children aged 12–17 across 12 countries in Africa and Asia (2020–2021). Basemap boundaries: Natural Earth (naturalearthdata.com; public domain).

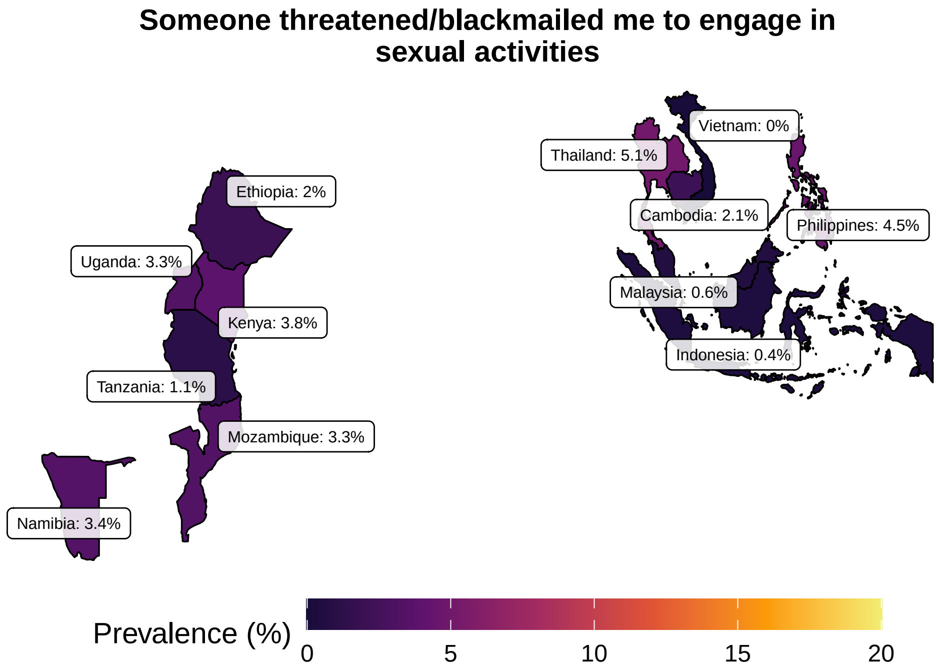

Figure 10: Survey-weighted prevalence of nine forms of technology-facilitated CSEA among internet-using children aged 12–17 across 12 countries in Africa and Asia (2020–2021). Basemap boundaries: Natural Earth (naturalearthdata.com; public domain).

2.1 Prevalence of Technology-facilitated CSEA Among All Children

Data Sources: To compute the dual reporting approach (among internet-using versus all children), we estimate the share of children who go online using the following two comparative sources.

a) **Estimating exposure (primary source)**: We estimate the share of 12–17-year-olds who use the internet with the Disrupting Harm (DH) household module (see Figure 11). During each household visit, enumerators first confirm whether any 12–17-year-olds live in the household and then ask whether those children use the internet (any use). The module was fielded nationally with probability sampling (approximately 1,500–10,000 households per country, depending on connectivity and country size), yielding age-specific estimates aligned to our fieldwork period. The DH household item is intentionally simple, a household-level screen about internet use among resident 12–17-year-olds to ensure consistent implementation across countries and years. This preserves both age specificity (12–17) and timing (aligned with the child survey). The module captures any internet use irrespective of device, making it appropriate for defining exposure for technology-facilitated outcomes.

Prevalence among all children was estimated as:

$$P_{\text{all}} = P_{\text{online}} \times \pi_{\text{internet}}$$

where P_{online} is the survey-weighted prevalence among internet-using children and π_{internet} is the estimated proportion of 12–17-year-olds using the internet from the DH household module.

b) **External context (not used in main estimates)**: For context and face validity, we next compare youth internet-use indicators from the International Telecommunication Union DataHub (datahub.itu.int) (see Figure 13). ITU compiles country-reported statistics, primarily nationally representative household surveys using standard ICT questions, into a harmonized database. Where recent observations are missing, ITU may publish estimates informed by earlier surveys and regional patterns. Age breakdowns are commonly available for youth (often 15–24 years), with additional disaggregation by gender and urban/rural in some settings (Figure 12). Internet use is defined broadly (any location, any device), but reference periods and exact wording can vary by country and year. Availability is uneven and reference years do not align with our fieldwork. To preserve age specificity (12–17) and timing, we retain the Disrupting Harm household estimate as the primary exposure measure and present ITU series as context.

2.1.1 Disrupting Harm Household survey vs International Telecommunication Union data sources

Figure 11 compares internet-use estimates from the DH household module (our primary internet exposure measure, ages 12–17) with the most recent ITU youth statistics (ages 15–24) for each study country.

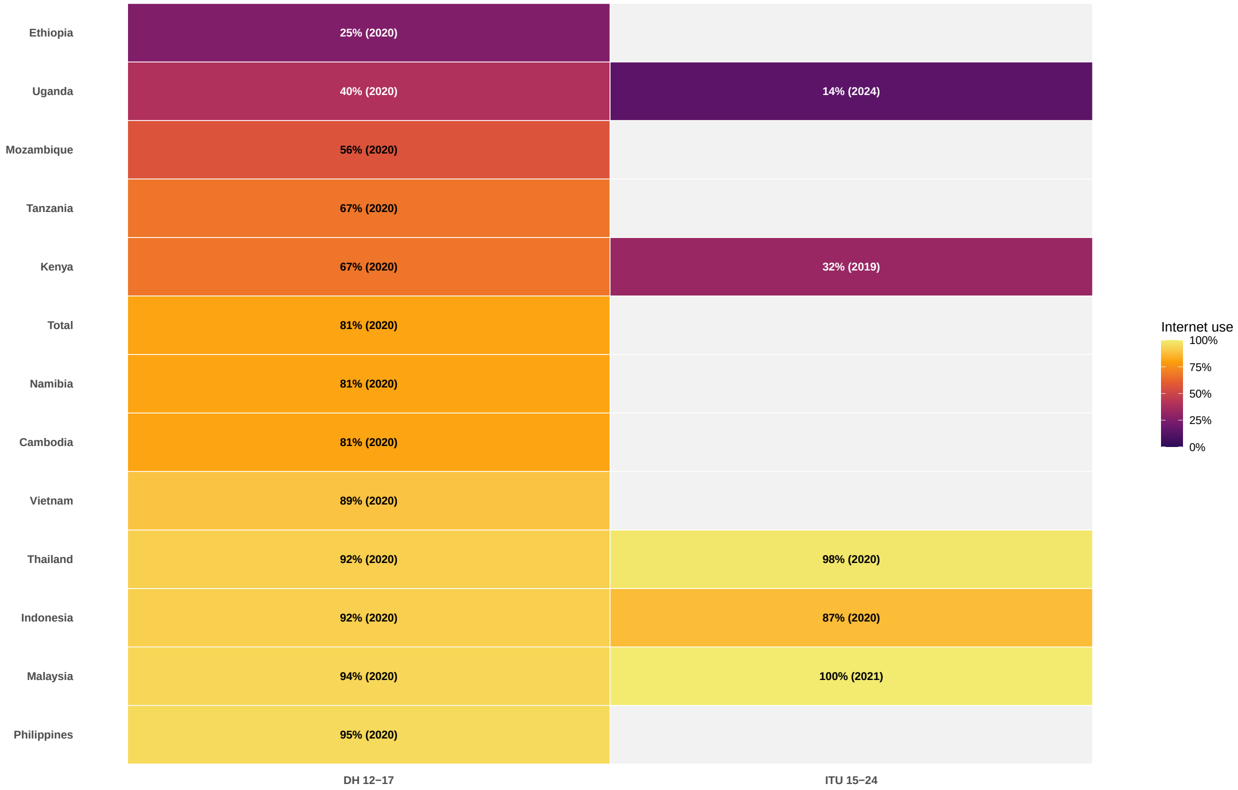

Figure 11: Availability of internet-use data from Disrupting Harm survey versus ITU DataHub. Heatmap showing internet use for study countries using DH household module estimates (ages 12–17) and ITU youth statistics (ages 15–24) as the closest available proxy to our target group (12–17 years). Each cell reports the percent using the internet and the reference year in parentheses (latest available; or nearest to 2020 if specified), with colour mapping 0–100%. ITU data source: ITU DataHub (datahub.itu.int), CC BY-NC-SA 3.0 IGO; used here for contextual comparison only.

2.1.2 ITU Gender Disaggregated data

Figure 12 presents gender-disaggregated internet-use estimates from ITU for the nine study countries where such data are available.

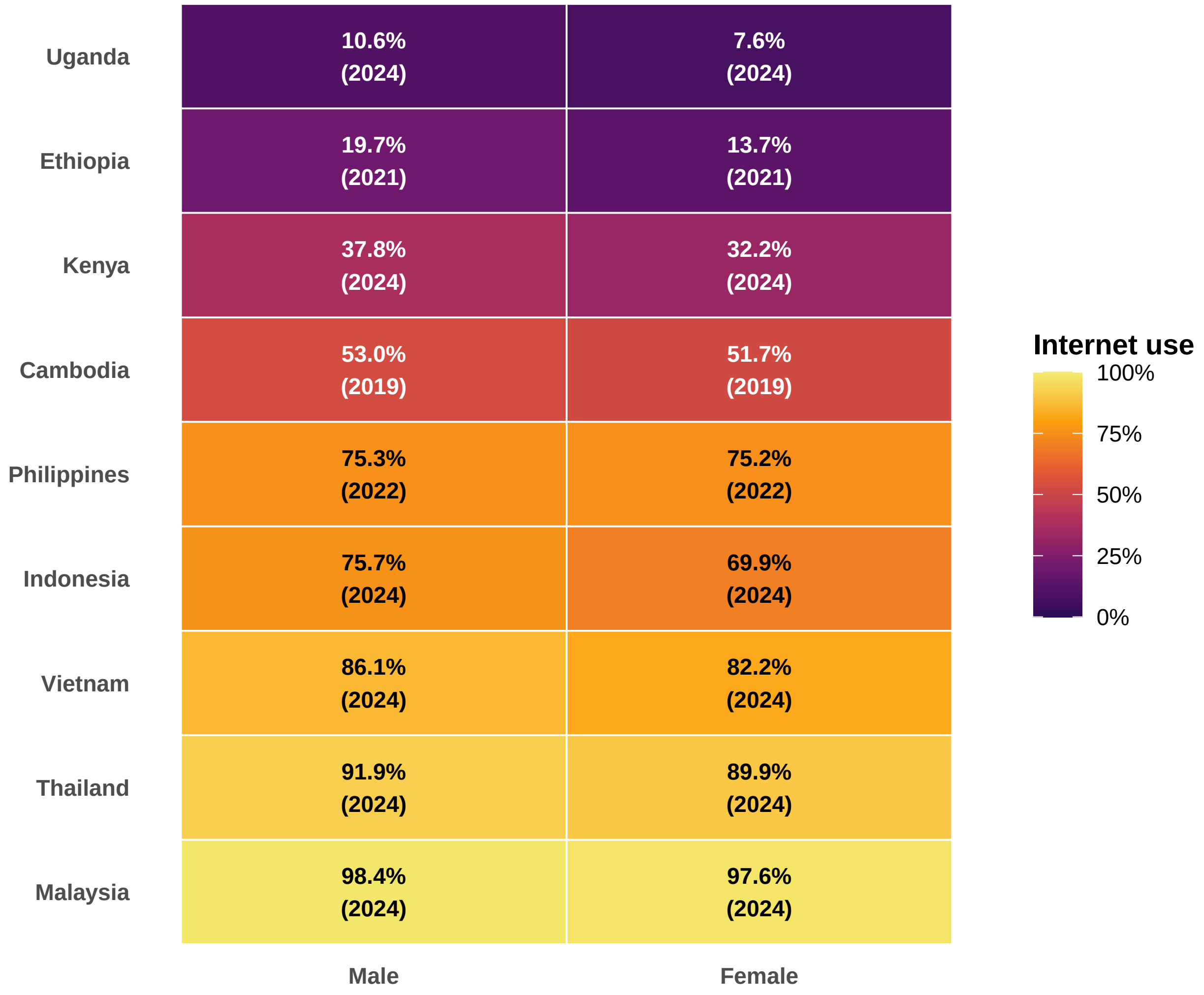

Figure 12: Availability of gender-disaggregated internet-use data in study countries. Heatmap showing youth (15–24 years) internet use from ITU DataHub statistics, used as the closest available proxy to our target group (12–17 years). For each country and sex, cells show the percentage using the internet and the reference year (latest available, or nearest to 2020 if specified), with colours mapping 0–100%. Only nine of the twelve study countries have gender-disaggregated ITU data and are shown here, ordered by data availability. ITU data source: ITU DataHub (datahub.itu.int), CC BY-NC-SA 3.0 IGO; used here for contextual comparison only.

2.1.3 Prevalence of technology-faciltated CSEA (DH household survey vs ITU comparisons)

Figure 13 plots country-level prevalence estimates against the twointernet exposure measures to highlight how the choice of denominator affects estimated prevalence among all children.

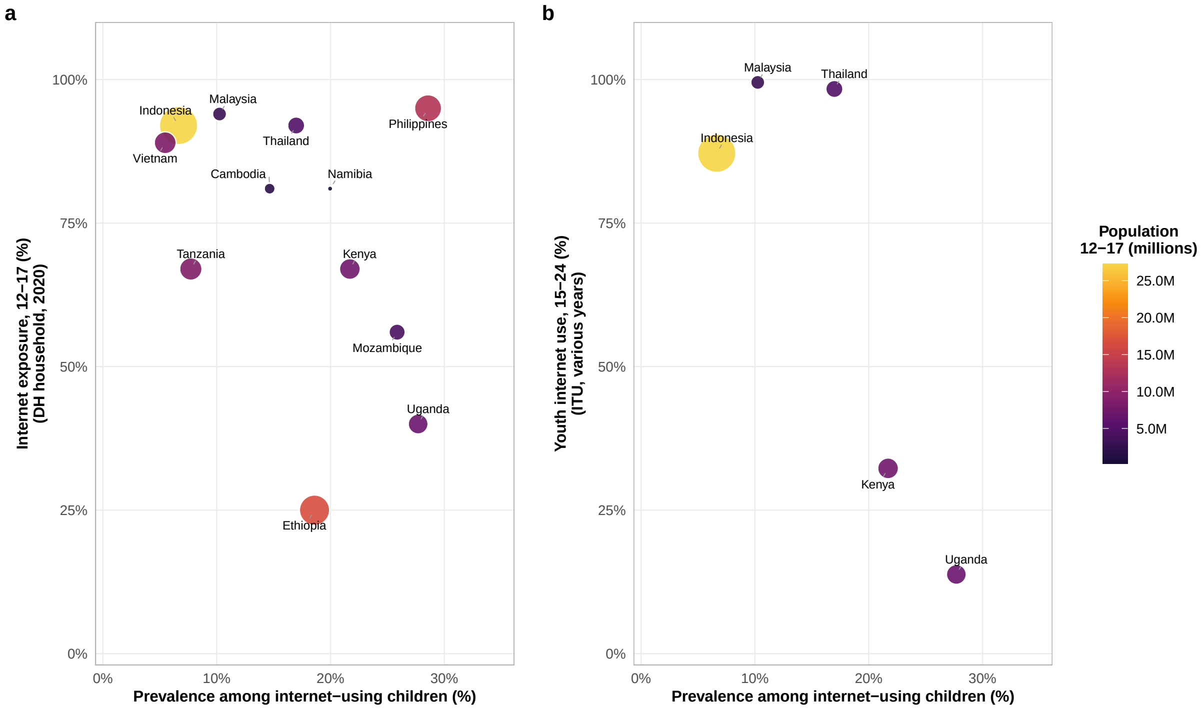

Figure 13: Comparison of Disrupting Harm (DH) and International Telecommunication Union (ITU) internet exposure measures. Panel (a) Prevalence among internet users (x-axis) versus DH household exposure estimates (y-axis, 12–17-year-olds, 2020). Panel (b) Prevalence among internet users (x-axis) versus ITU youth internet indicators (y-axis, 15–24-year-olds, various years). Point size and colour represent the 12–17-year-old population (millions). DH estimates (a) are used as the primary exposure measure; ITU indicators (b) provide validation. ITU data source: ITU DataHub (datahub.itu.int), CC BY-NC-SA 3.0 IGO; used here for contextual comparison only

2.1.4 Three Panel Comparisons of Prevalence and Internet Exposure

Figure 14 presents three panels linking technology-facilitated CSEA prevalence among internet users (Disrupting Harm (DH) child survey, internet exposure rates (DH household survey), and the resulting population-level prevalence.

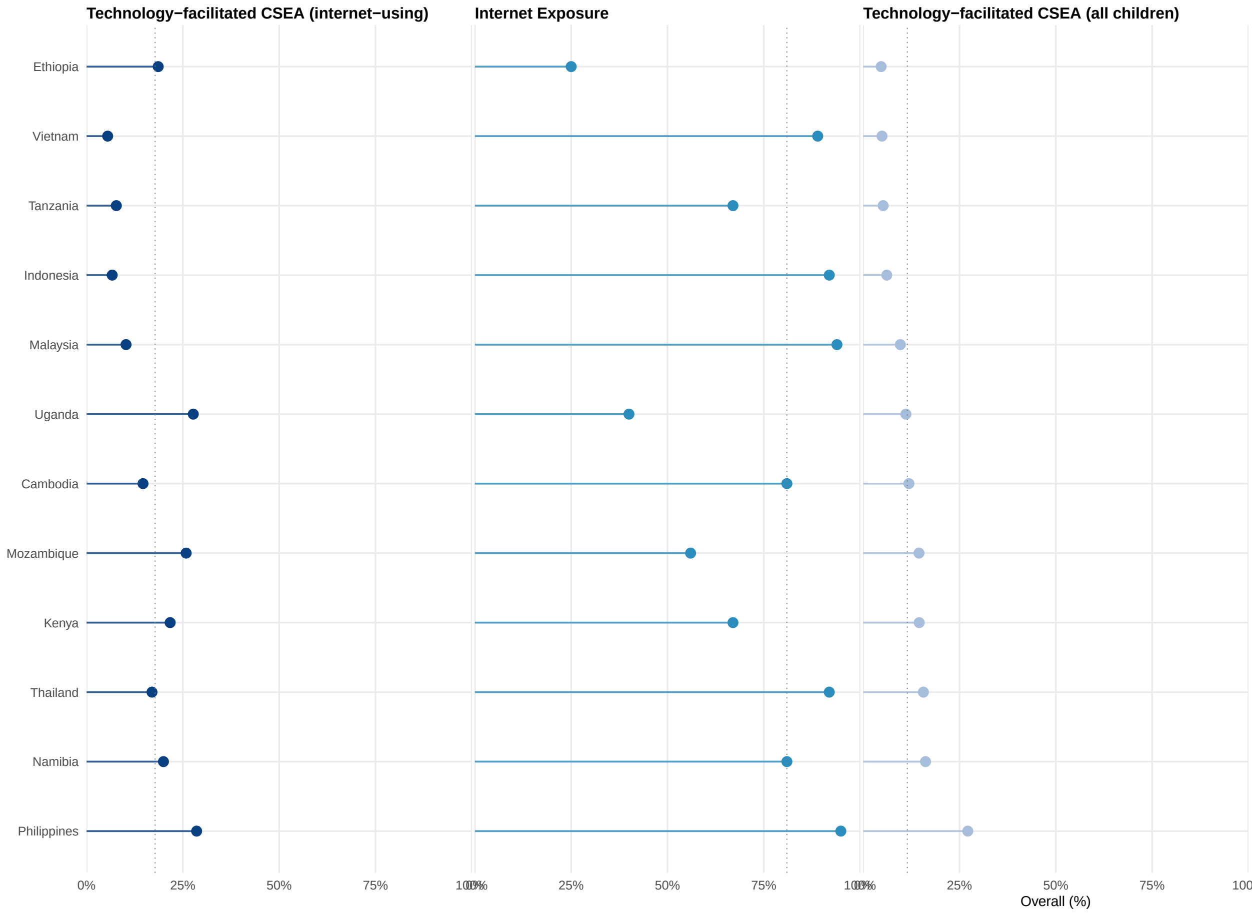

Figure 14: Technology-facilitated CSEA, exposure, and overall population prevalence (ages 12–17). Panel (a) shows the prevalence of technology-facilitated CSEA among internet-using 12–17-year-olds (Disrupting Harm (DH) child survey; survey-weighted). Panel (b) shows internet exposure from the DH household survey: the percentage of all 12–17-year-olds who use the internet. Panel (c) shows the overall prevalence among all 12–17-year-olds, computed as Panel (a) multiplied by Panel (b) for each country and year. Countries are ordered by the overall estimate in Panel (c); dashed vertical lines mark medians.

2.2 Uncertainty Propagation

We quantify uncertainty in overall prevalence by propagating sampling error from both data sources, the DH child survey (prevalence among internet-using children) and the DH household survey (proportion of 12–17-year-olds online), using a parametric Monte Carlo procedure on the logit scale. For each country, we generate 5,000 random draws from the sampling distribution of each component, assuming approximate normality on the logit scale with means equal to the point estimates and standard deviations equal to the survey-design standard errors (or conservative defaults when standard errors are unavailable). Each draw is inverse-logit transformed back to the probability scale, and overall prevalence is calculated as the product of the paired draws from the two components. We report 95% confidence intervals as the 2.5th and 97.5th percentiles of the resulting distribution.We calculated standard errors using effective sample sizes (Kish, 1965), which account for survey weights

The DH household survey module asked enumerators to record whether 12-17 year-olds in each household used the internet, yielding nationally representative proportions. However, the aggregated data available to researchers contains only the final weighted proportions without the underlying sample counts or design parameters needed to calculate standard errors. This data structure requires our conservative default SE approach for the exposure component of the uncertainty propagation. Our conservative default of 3 percentage points ensures confidence intervals remain appropriately wide, avoiding false precision in population estimates.

This approach treats the child and household surveys as independent samples, which is appropriate given their separate sampling frames and data collection procedures. National population sizes for ages 12–17 are treated as fixed. The confidence intervals for estimated numbers affected reflect uncertainty in the two prevalence rates but not in population counts. Point estimates of overall prevalence are calculated as the direct product of the two component estimates and do not depend on the Monte Carlo procedure or default standard error assumptions. This approach follows standard methods for propagating uncertainty in products of independent estimates (Wolter, 2007).

Applying this approach provides an overall estimated *10,713,632 (95% CI: 9,924,786–11,495,650)* children affected across the 12 countries.

Methodological Caveats: Several factors mean that our estimates should be interpreted as conservative lower bounds on the true burden:

- *Temporal limitations*: Internet exposure data derive from the 2020 DH household survey, while DH data span 2020–2021. In countries experiencing rapid digitalization, exposure rates may have changed between these measurement periods and have increased substantially by 2025. These estimates therefore reflect 2020–2021 conditions and do not represent current population burden.
- *Core analytical assumption*: Our framework treats children without internet access as unexposed to technology-facilitated harms. While this is a reasonable assumption for outcomes that require online interaction, it cannot be empirically verified and assumes a clear online/offline boundary that may not fully capture hybrid harm pathways.
- *Internet exposure measurement*: The DH household measure captures reported internet use by resident 12–17-year-olds but may not capture all occasional or informal internet access occurring outside the household (e.g., at schools, libraries, friends' homes, or public venues). Children reported as non-users in the household module may therefore have intermittent exposure to online harms meaning the true exposed population could be larger than estimated.
- *Uncertainty propagation methods*: We use Monte Carlo simulation (5,000 draws per country) with logit-transformed proportions, a standard approach for bounded measures that can be less accurate at extreme values (very close to 0 or 1). We bound all estimates to mitigate this limitation.
- *Survey independence assumption*: The DH child and household modules use separate sampling frames and are treated as statistically independent in our uncertainty propagation. While this is appropriate for sampling error, any unmeasured correlation between the two estimates within countries (e.g., due to shared fieldwork timing or geographic clustering) could affect confidence interval widths.

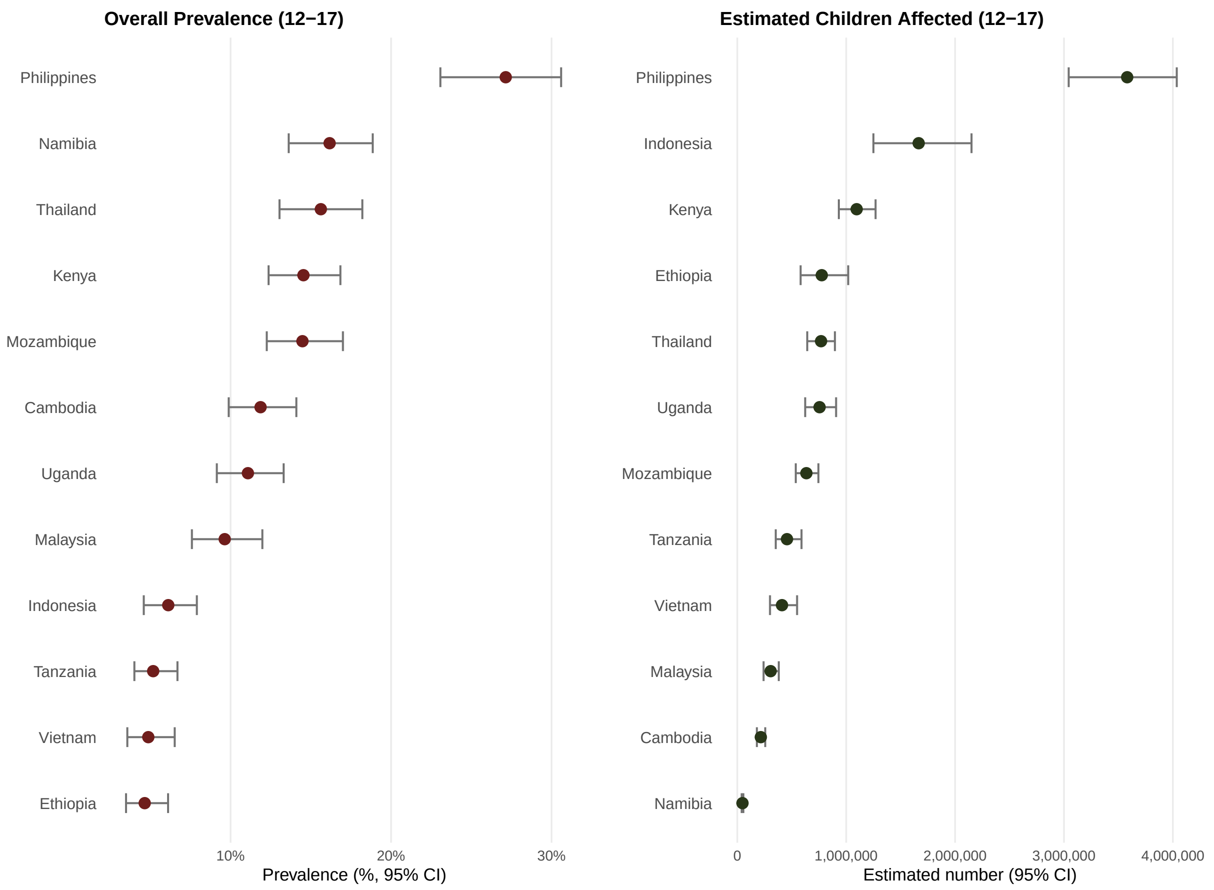

Figure 15: Country-level prevalence and burden of technology-facilitated CSEA (ages 12–17). Left: estimated overall prevalence among all 12–17-year-olds (% with 95% CIs). Right: estimated number of affected children (95% CIs). For each country, overall prevalence is computed as the product of (a) prevalence among internet-using children (DH child survey) and (b) the proportion of all 12–17-year-olds who use the internet (DH household survey). Counts equal overall prevalence × national 12–17 population (UN WPP 2022, year 2020). Uncertainty is propagated via parametric Monte Carlo on the logit scale (5,000 draws per country); population counts are treated as fixed. Countries are ordered separately within each panel by the point estimate.

2.3 Categorisation of Online Child Sexual Abuse and Exploitation (OCSEA)

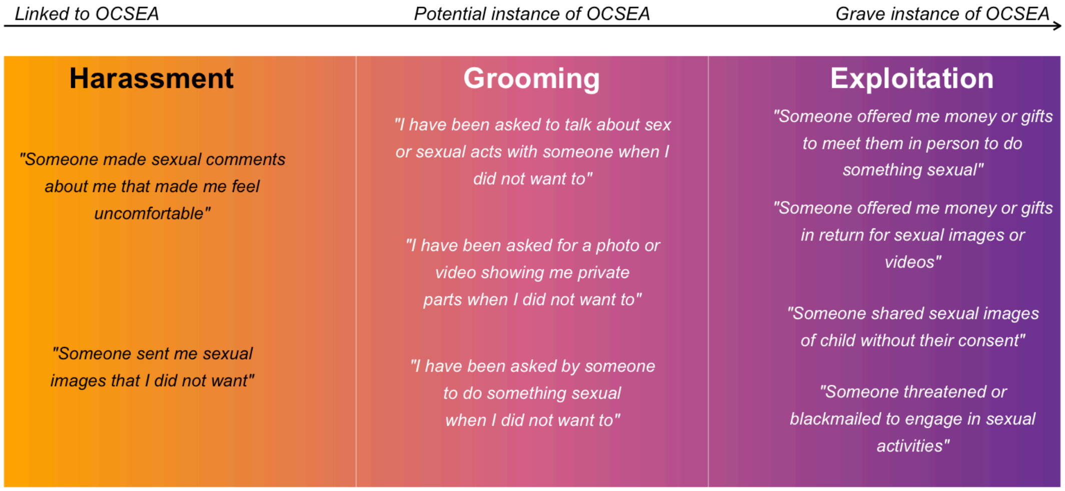

Figure 16: Spectrum of online child sexual exploitation and abuse (OCSEA) measured in the Disrupting Harm survey. Items are organised along a continuum from experiences linked to OCSEA (left) through potential instances (centre) to grave instances (right).

We initially conceptualised OCSEA along three categories in our preregistration:

1) **Solicit or Harassment** includes instances of unwanted exposure to sexual content: a) *Unwanted sexual comments*: remarks of a sexual nature made about a child, such as jokes, anecdotes, or comments regarding their body, appearance, or sexual activities. b) *Unwanted sexual images*: someone sending the child explicit photographs or videos of a sexual nature without their consent.

2) **Grooming** includes possible instances of online sexual exploitation and abuse, such as solicitation for sexual engagement or sharing of child sexual abuse material (CSAM): c) *Unwanted sexual talk*: a child is solicited by a perpetrator to engage in conversations about sex or sexual acts against their will. d) *Unwanted sexual act requests*: a child is pressured or asked by a perpetrator to engage in sexual activities that they do not wish to participate in. e). *Solicited sexual images/videos*: a perpetrator asks for photographs or videos showing the private parts or nudity of a child without their consent.

3) **Preparate or Exploitation** includes grave instances of online sexual exploitation and abuse, such as coercion or blackmail for sex, or the exchange of money for CSAM: f) *Commercial images/videos*: a perpetrator offers money or gifts to a child in exchange for sexual images or videos. g) *Commercial sexual act requests*: a child is offered money, gifts, or other incentives in exchange for meeting a perpetrator in person to engage in sexual activities. h) *Non-consensual image sharing*: distributing or sharing sexual images or videos of a child without their explicit consent. i) *Sextortion*: a form of extortion where a perpetrator uses coercion, threats, or blackmail to compel a child to engage in sexual activity.

However, we encountered theoretical challenges in maintaining these distinctions. Attempting to impose rigid boundaries risked oversimplifying the complexity of how children experience and describe harm online. Several survey items captured experiences that could plausibly fall under multiple categories, further complicating consistent classification.

Given the updated terminologies proposed by the Luxembourg Guidelines (Interagency Working Group on Sexual Exploitation of Children, 2016; updated 2023), we deviated from our preregistered framework and adopted the umbrella

term "technology-facilitated CSEA," analysing all nine items as a unified construct rather than maintaining the three-category distinction.

2.4 Measurement of Technology-Facilitated CSEA

This section details the operationalisation of CSEA prevalence indicators derived from the DH survey. All measures are based on survey items capturing nine distinct forms of CSEA, with children indicating the context in which each incident occurred (social media, online game, in person, or other). Children were asked whether they had experienced each of the following nine forms of CSEA (Supplementary Figures 2-9). For each item, children indicated where the incident took place using binary flags; response options also included "don't know" and "prefer not to say." See Supplementary Table 54 in the Descriptives Summaries section for country-level estimates.

Operationalisation of CSEA Indicators

Primary Measure: Technology-Facilitated CSEA

This dichotomous variable identifies children who experienced at least one form of CSEA explicitly through a digital platform (social media or online game). The variable equals 1 if a respondent reported any of the nine abuse items and indicated that the incident took place either on social media or in an online game. Children who reported no abuse, or abuse that occurred only in person, through "some other way," or who did not specify a location were coded 0.

This measure requires direct evidence of a technology-mediated context. This prioritises specificity, which likely produces a conservative estimate. Consistent with the Luxembourg Guidelines (Interagency Working Group on Sexual Exploitation of Children, 2016; updated March 2025) which incorporated input from survivors to avoid stigmatizing, victim-blaming language, we prioritise precise terminology for technology-facilitated offences, while noting that the Guidelines do not prescribe a particular measurement design. This is the primary dependent variable in the regression models reported in the main manuscript.

Inferred Technology-Facilitated CSEA

This indicator captures technology-facilitated CSEA by recognising that certain abuse types are inherently digital in nature, even when respondents did not explicitly confirm the platform. Building on the Disrupting Harm Data Insight 1 (UNICEF Office of Research–Innocenti, 2022) approach of treating certain items as inherently online by content, image-based items are treated as digitally mediated based on item content, while mixed-modality items are classified as technology-facilitated only when an explicit online channel is confirmed.

Intrinsically digital items: the following four items were coded as technology-facilitated regardless of whether a digital platform was explicitly confirmed, as their content is inherently image- or media-based: someone sent the child sexual images they did not want (`abuse_b`); the child was asked for a photo or video showing their private parts (`abuse_e`); someone offered money or gifts in return for sexual images or videos (`abuse_f`); and someone shared sexual images of the child without their consent (`abuse_h`).

Mixed-modality items: the following five items could occur either online or offline and were therefore coded as technology-facilitated only when the child explicitly confirmed the incident occurred on social media or in an online game: someone made unwanted sexual comments (`abuse_a`); the child was asked to talk about sex or sexual acts with someone (`abuse_c`); the child was asked by someone to do something sexual (`abuse_d`); someone offered money or gifts to meet in person to do something sexual (`abuse_g`); and someone threatened or blackmailed the child to engage in sexual activities (`abuse_i`).

Any CSEA Exposure

This aggregate indicator captures overall CSEA burden regardless of location or content. The variable equals 1 if a child reported experiencing any of the nine CSEA items, regardless of where the incident occurred. This includes incidents occurring via social media, online games, in person, some other way, or where location was not specified (don't know/prefer not to say responses). This captures the broadest estimate of CSEA exposure and contextualises the two location-specific measures above.

Note on missing data: Primary TF-CSEA and Inferred TF-CSEA are conditioned on platform flags (social media or online gaming), allowing item-level non-response to be coded as 0 (no online incident reported). Any CSEA exposure is unconditional on location: capturing incidents across social media, online games, in-person contact, other contexts, and unspecified locations (including "don't know" and "prefer not to say" responses; see Supplementary Table 4 for the location distribution). We therefore do not use location flags to resolve item-level missingness. For this measure, the denominator is restricted to respondents with valid responses on the nine abuse items, excluding 728 (weighted) respondents. The main manuscript reports Any CSEA exposure prevalence as 31.1% on this restricted sample (weighted n = 11,184); a sensitivity estimate over the full weighted sample (n = 11,912), treating item-level non-response as no reported experience, gives 29.2%.

Table 3: Survey-weighted prevalence of three operationalisations of CSEA among internet-using children aged 12–17 across 12 countries (2020–2021). TF-CSEA (primary) requires explicit confirmation that the incident occurred via social media or an online gaming platform. Inferred TF-CSEA additionally treats four intrinsically digital abuse types (unwanted sexual images received, requests for intimate photos/videos, money or gifts offered in exchange for sexual images, and non-consensual sharing of sexual images) as technology-facilitated regardless of platform confirmation. Any CSEA exposure captures all nine abuse types regardless of location; its denominator is restricted to respondents with valid data on the nine abuse items (weighted n = 11,184). Note: Values are n (%); all estimates are survey-weighted.

**Characteristic**	**N = 11,912**
TF-CSEA (primary)	2,025 (17%)
Infered TF-CSEA	2,761 (23%)
Any CSEA exposure	3,477 (31%)
Unknown	728

[1] n (%)

2.5 Location of Technology-Facilitated CSEA Incidents

For each of the nine CSEA items, children who reported an experience were asked where the incident occurred. Response options were social media, online game, in person, some other way, don't know and prefer not to say, with multiple selections permitted. Responses of "don't know" and "prefer not to say" are also reported. This design allowed children to indicate both known locations and uncertainty, for example, by selecting social media and "don't know". We therefore report location information while preserving this uncertainty: a) Cases with only substantive locations are classified by those locations; b) Cases with only "don't know" or "prefer not to say" are classified as uncertain; c) Cases with both substantive and uncertain responses are also reported to maintain transparency.

Table 4: Survey-weighted distribution of location patterns for technology-facilitated CSEA incidents (2020–2021). Each row represents a mutually exclusive combination of reported locations: single locations (social media only, online game only, in person only, other), multi-location combinations (digital and in person, digital and other), cases with three or more simultaneous locations, and cases with uncertain responses ('don't know' or 'prefer not to say'). Children could select multiple locations per incident; categories are constructed to be mutually exclusive. Weighted N uses survey probability weights (wgt_scaled). '% of Total' is relative to all incidents with any location response; '% of Substantive' excludes uncertain responses from the denominator. 'Substantive + uncertain' indicates incidents where a specific location and an uncertain response were selected simultaneously.

Location Pattern[1]	N[2]	% of Total	% of Substantive
In person only	498	14.3	17.5
Social media only	826	23.8	29.0
Online game only	98	2.8	3.4
Some other way only	126	3.6	4.4
Digital + in person	375	10.8	13.1
Digital + some other way	131	3.8	4.6
In person + some other way	56	1.6	2.0
Three+ locations	264	7.6	9.2
Substantive + uncertain[3]	[3]531	[3]15.3	[3]NA
Don't know only	335	9.6	NA
Prefer not to say only	229	6.6	NA
**Any digital (social/game)**	**2,025**	**58.2**	**NA**
**Any substantive location**	**2,853**	**82.0**	**NA**
**Total with any response**	**3,477**	**100.0**	**NA**

[1]Children could select multiple location options simultaneously. 'Digital' refers to social media or online game platforms.
[2]Weighted using survey weights (wgt_scaled).
[3]'Substantive + uncertain' indicates cases where respondents selected both a specific location AND 'don't know' or 'prefer not to say'.

2.6 Heatplot (Location of Technology-Facilitated CSEA Incidents)

This heatplot visualises the distribution of CSEA incidents across locations (social media, in person, online games, and other) by CSEA type and country.The most commonly reported location was social media, followed by in person and online games.

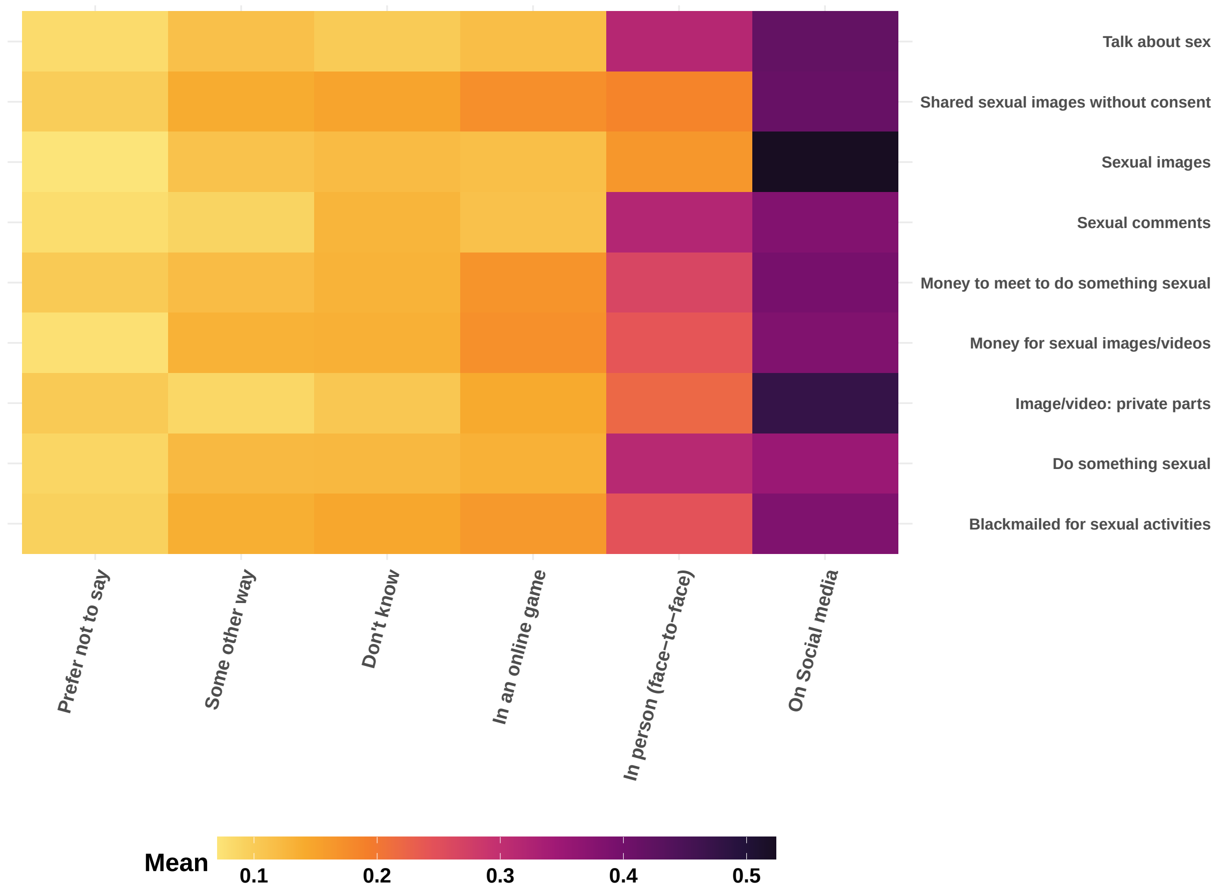

Figure 17: Location of technology-facilitated CSEA incidents by abuse type (survey-weighted proportions, 2020–2021). Each cell shows the proportion of children who experienced a given CSEA type and reported the incident occurring in that location. Children could select multiple locations. Darker cells indicate higher proportions.

2.7 Online Platforms Involved in Technology-Facilitated CSEA Incidents

This heatplot visualises online platforms involved in technology-facilitated CSEA incidents. While most of the DH survey is platform-agnostic, adolescents were asked to identify the specific platform where they experienced technology-facilitated CSEA (On which platforms or websites did this happen?). Three most common platforms where children experienced technology-facilitated CSEA were Facebook, WhatsApp and Instagram.

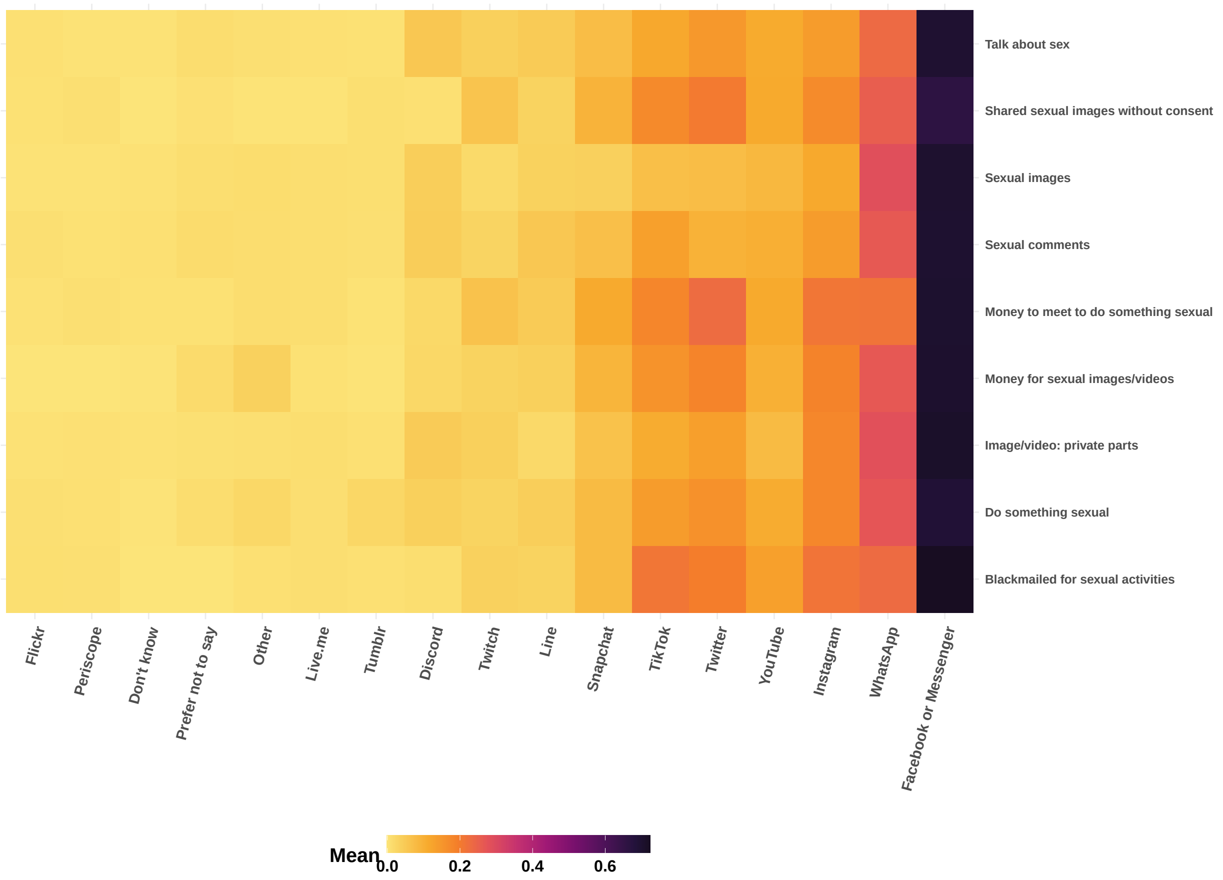

Figure 18: Social media platforms involved in technology-facilitated CSEA by abuse type (survey-weighted proportions, 2020–2021). Each cell shows the proportion of children who experienced a given form of CSEA and reported it occurring on that platform. Children could select multiple platforms. Darker cells indicate higher proportions.

2.8 Perpetrator Identity in Technology-Facilitated CSEA Incidents

Children who experienced technology-facilitated CSEA were asked to identify the perpetrator from a set of response options. Children were asked: Thinking now about the last time this happened, as far as you know, who was the person or people who did this? and could select someone in their family, a romantic ex, a friend or someone else below or above 18 years old or someone they didn't know before this happened or someone else (incl. prefer not to say, or they didn't know who the person was).

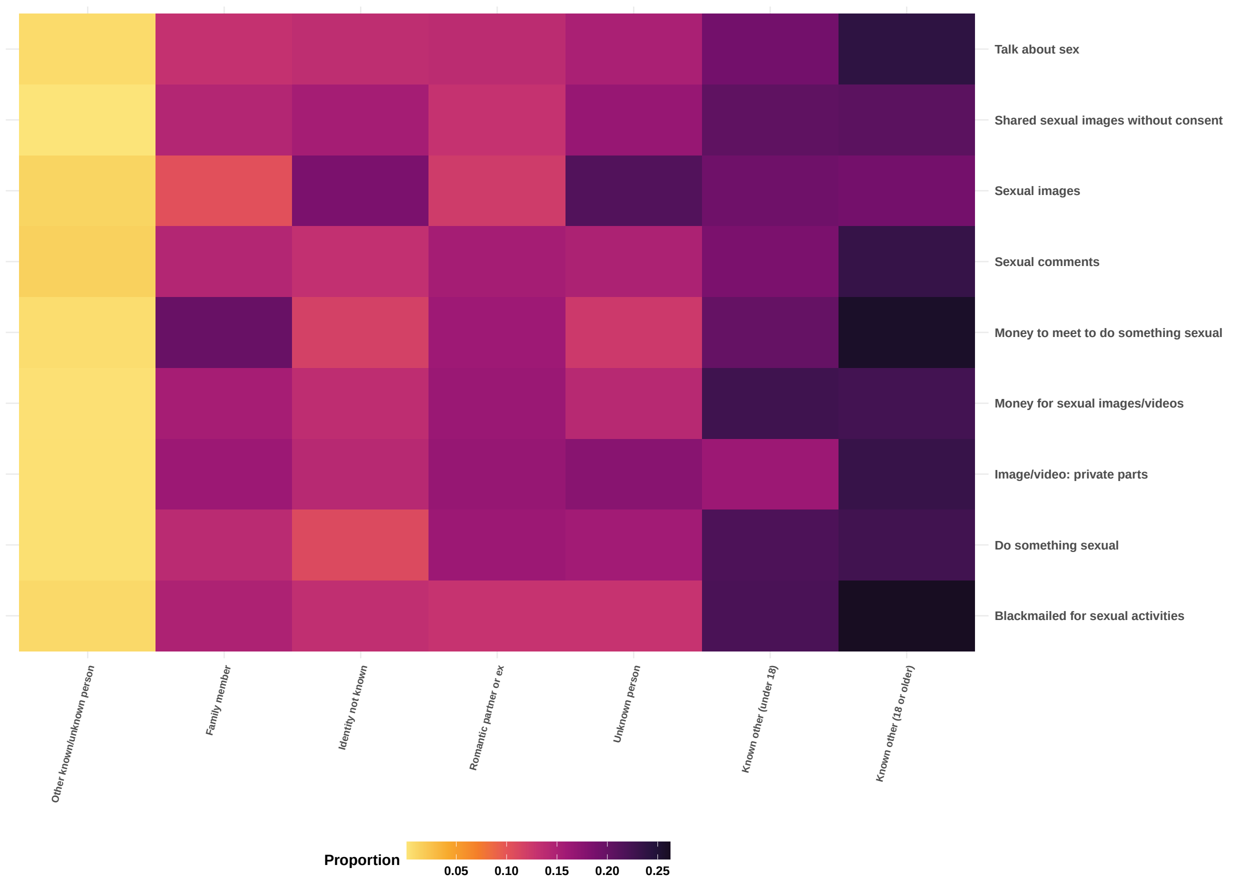

Figure 19: Perpetrator identity in technology-facilitated CSEA incidents by abuse type (survey-weighted proportions, 2020–2021). Each cell shows the proportion of children who experienced a given form of CSEA and identified that perpetrator type. Children could report multiple perpetrator types. Darker cells indicate higher proportions.

2.9 Demographic Predictors of Technology-facilitated CSEA

This analysis was not part of our preregistered analysis plan. We conducted it post hoc based on the expectation that demographic factors: age, gender, and degree of urbanisation may be relevant to children's self-reported experiences of technology-facilitated CSEA. We report three multilevel logistic regression specifications. In all models, the outcome is binary (experienced technology-facilitated CSEA vs. not), a logit link is used, and survey weights (wgt_scaled) are applied. Country is modelled as a grouping factor to capture between-country heterogeneity.

Model 1: Additive fixed effects (gender + age) This specification includes fixed effects for gender and age without their interaction, and allows these effects to vary by country through random intercepts and random slopes. The term (gender × age | country) expands to a country-specific intercept and country-specific slopes for gender, age, and their interaction, with a full covariance structure. Thus, while the average (fixed) association is additive, countries are permitted to deviate in both baseline prevalence and the magnitude of gender and age effects.

Table 5: Population-level parameter summaries for Model 1: multilevel logistic regression with additive gender and age effects (N = 11912; 12 countries). Estimates are posterior means on the log-odds scale with 95% credible intervals. PD = probability of direction.

Parameter	Mean	SD	2.5% CI	97.5% CI	PD > 0)
Girls (vs Boys)	0.00	0.12	-0.25	0.23	51.42%
Age (per year)	0.18	0.05	0.09	0.28	99.90%

Model 2: Interaction fixed effects (gender × age)

This specification adds a fixed interaction between gender and age, allowing the gender difference to change with age on average across countries, while retaining the same random-effects structure. Both population-level and country-specific age-by-gender patterns are estimated: the fixed part captures the overall interaction, and the random part permits each country to differ in its intercept and in the slopes for gender, age, and their interaction.

Table 6: Population-level parameter summaries for Model 2: multilevel logistic regression with gender × age interaction (N = 11912; 12 countries). Estimates are posterior means on the log-odds scale with 95% credible intervals. PD = probability of direction.

Parameter	Mean	SD	2.5% CI	97.5% CI	PD > 0)
Girls (vs Boys)	-0.01	0.11	-0.22	0.20	53.55%
Age	0.18	0.04	0.10	0.26	> 99.9%
Age:Gender interaction	0.09	0.04	0.01	0.17	98.50%

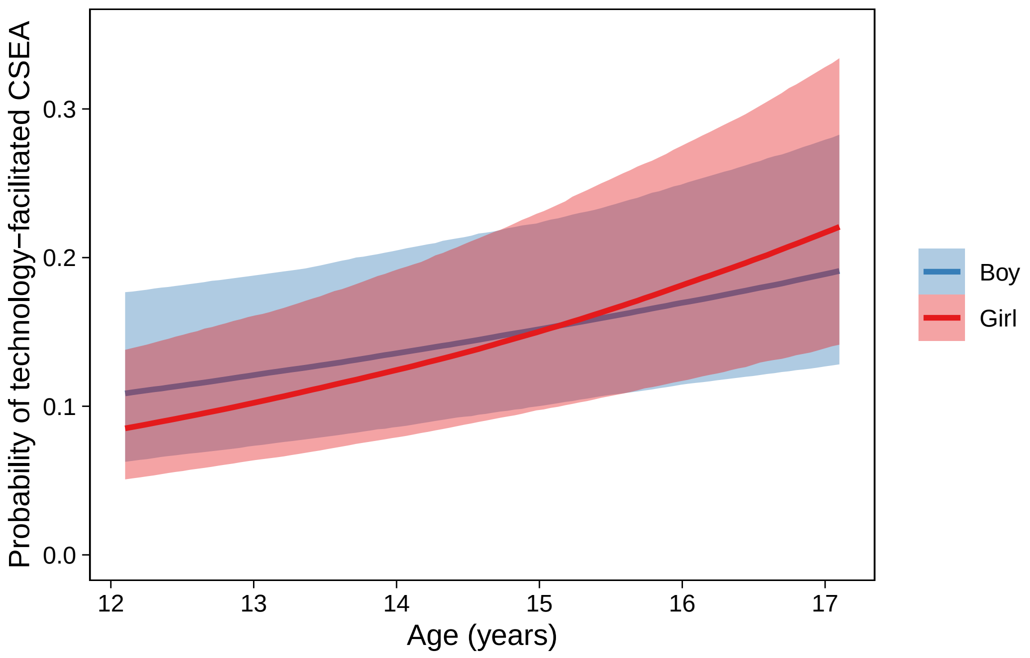

Figure 20: Predicted probability of technology-facilitated CSEA by age and sex (Model 2: gender × age interaction), evaluated at the average country. Shaded bands represent 95% credible intervals. Age shown on original scale (12–17 years).

Table 7: Predicted probabilities of technology-facilitated CSEA at ages 12 and 17 for boys and girls in each country (Model 2: gender × age interaction reported in main manuscript). Point estimates and 95% Bayesian credible intervals are derived from posterior predictions.

Country	Age	Sex	Estimate	Lower Bound	Upper Bound
Cambodia	12	Girl	0.11	0.08	0.16
Cambodia	12	Boy	0.18	0.12	0.23
Cambodia	17	Girl	0.15	0.10	0.19
Cambodia	17	Boy	0.15	0.11	0.20
Ethiopia	12	Girl	0.06	0.04	0.10
Ethiopia	12	Boy	0.06	0.03	0.10
Ethiopia	17	Girl	0.29	0.23	0.36
Ethiopia	17	Boy	0.22	0.19	0.26
Indonesia	12	Girl	0.03	0.02	0.06
Indonesia	12	Boy	0.05	0.03	0.08
Indonesia	17	Girl	0.09	0.06	0.13
Indonesia	17	Boy	0.10	0.07	0.15
Kenya	12	Girl	0.11	0.08	0.15
Kenya	12	Boy	0.15	0.10	0.20
Kenya	17	Girl	0.30	0.25	0.36
Kenya	17	Boy	0.28	0.23	0.34
Malaysia	12	Girl	0.07	0.05	0.11
Malaysia	12	Boy	0.09	0.06	0.14
Malaysia	17	Girl	0.14	0.09	0.18
Malaysia	17	Boy	0.12	0.08	0.16
Mozambique	12	Girl	0.19	0.14	0.25
Mozambique	12	Boy	0.23	0.17	0.30
Mozambique	17	Girl	0.32	0.26	0.38
Mozambique	17	Boy	0.26	0.21	0.31
Namibia	12	Girl	0.10	0.07	0.14
Namibia	12	Boy	0.11	0.07	0.15
Namibia	17	Girl	0.34	0.28	0.40
Namibia	17	Boy	0.24	0.19	0.30
Philippines	12	Girl	0.17	0.12	0.22
Philippines	12	Boy	0.23	0.17	0.30
Philippines	17	Girl	0.40	0.34	0.47
Philippines	17	Boy	0.34	0.27	0.40
Tanzania	12	Girl	0.04	0.02	0.06
Tanzania	12	Boy	0.06	0.03	0.09
Tanzania	17	Girl	0.11	0.07	0.15
Tanzania	17	Boy	0.11	0.08	0.14
Thailand	12	Girl	0.11	0.08	0.15
Thailand	12	Boy	0.18	0.12	0.24
Thailand	17	Girl	0.19	0.14	0.24
Thailand	17	Boy	0.21	0.16	0.27
Uganda	12	Girl	0.11	0.07	0.16
Uganda	12	Boy	0.11	0.07	0.16
Uganda	17	Girl	0.42	0.36	0.49
Uganda	17	Boy	0.34	0.29	0.39
Vietnam	12	Girl	0.03	0.02	0.05

Vietnam	12	Boy	0.03	0.02	0.06
Vietnam	17	Girl	0.10	0.06	0.14
Vietnam	17	Boy	0.08	0.05	0.11

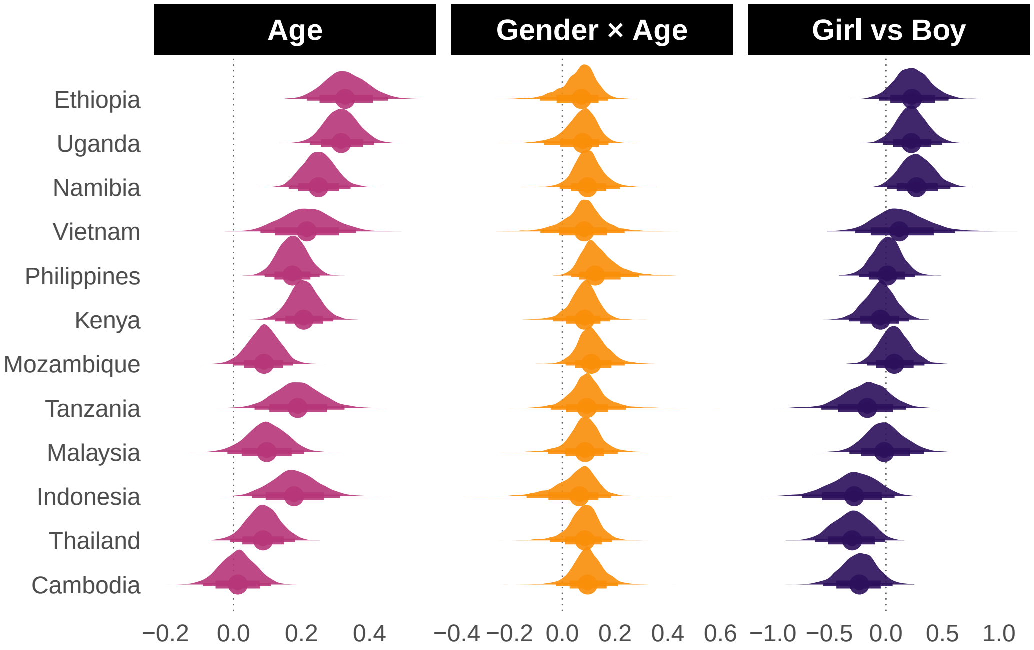

Figure 21: Country-specific posterior distributions for Model 2 coefficients (log-odds scale). Each panel shows one parameter: age (+1 year), gender (girls vs. boys), and their interaction. Thick and thin horizontal bars represent 80% and 95% credible intervals, respectively. Vertical dotted lines mark zero (no association). Countries are ordered by posterior median.

2.10 Leave-One-Out Cross-Validation (Demographic models)

We used leave-one-out cross-validation (LOO) to compare two specifications of the CSEA exposure model that differ in their fixed effects: an additive model (gender + age) and an interaction model (gender × age). Both models included identical random-effects structures (random intercepts and slopes for gender, age, and their interaction by country). Both showed acceptable diagnostics (all Pareto-k < 0.7). Predictive performance did not meaningfully differ between specifications (elpd_diff = −0.9, SE = 1.9; interaction model as reference), indicating that including the fixed interaction does not improve out-of-sample prediction. Given the theoretical relevance of potential age-by-gender differences in risk of experiencing CSEA, and because predictive performance is not worsened by its inclusion, we retain the interaction model as our primary specification.

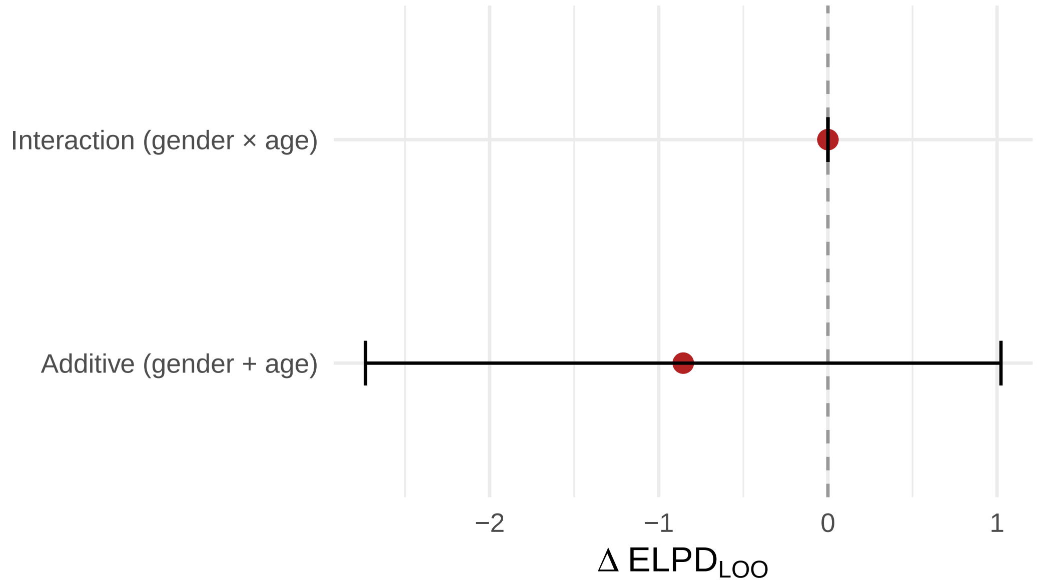

Figure 22: Leave-one-out cross-validation (LOO) comparison of two model specifications of CSEA exposure: an additive model (gender + age) versus an interaction model (gender × age). Both models included identical random-effects structures. Points show the ELPD difference (ΔELPD) relative to the best model; error bars are ±1 SE. Positive ΔELPD indicates better out-of-sample predictive fit.

2.11 Degree of Urbanisation Differences

Model 3: Degree of urbanisation model (gender × age + urban-rural-periurban)

Building on Model 2, this specification adds an additive term for degree of urbanisation, estimating systematic differences between urbanisation categories while holding gender and age constant. Random effects by country remain the same as above (intercept and slopes for gender, age, and their interaction). Degree of urbanisation is treated as a population-level modifier without country-varying slopes, providing a parsimonious adjustment for differences across urban, rural and peri-urban areas.

Table 8: Population-level parameter summaries for Model 3: multilevel logistic regression with gender × age interaction and degree of urbanisation (N = 11912; 12 countries). Estimates are posterior means on the log-odds scale with 95% credible intervals. PD = probability of direction.

Parameter	Mean	SD	2.5% CI	97.5% CI	PD > 0)
Girls (vs Boys)	-0.01	0.10	-0.23	0.19	55.05%
Age	0.18	0.04	0.10	0.26	99.98%
Rural	-0.32	0.09	-0.49	-0.14	> 99.9%
Urban	-0.14	0.09	-0.32	0.05	93.05%
Age:Gender interaction	0.08	0.04	0.00	0.16	97.42%

2.11.1 Weighted predicted probabilities by degree of urbanisation

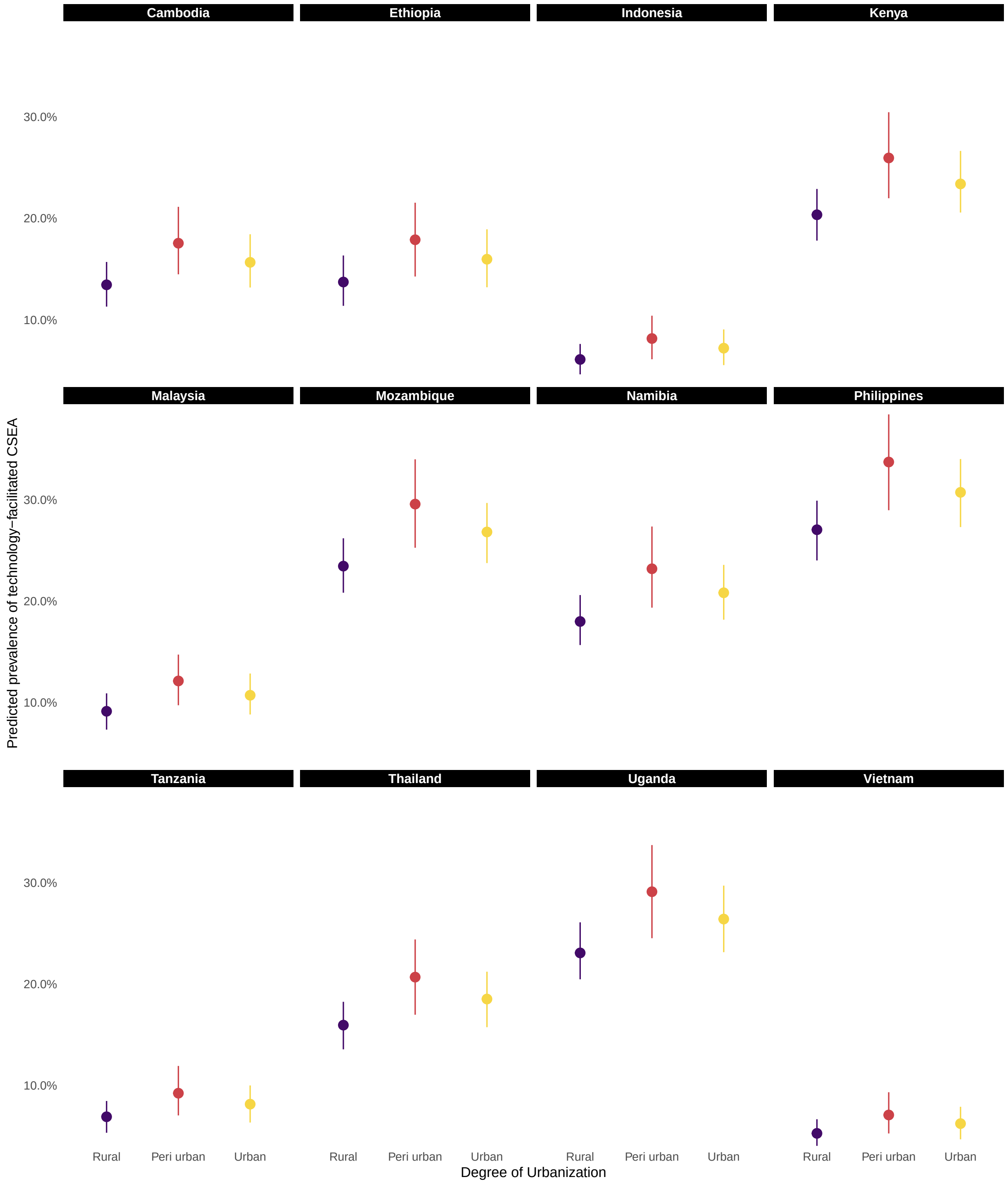

Figure 23: Predicted prevalence of technology-facilitated CSEA by degree of urbanisation (Rural, Peri-urban, Urban), with 95% credible intervals. Values are posterior means from the survey-weighted Bayesian regression, marginalised over covariates in the fitted model (e.g. age, gender). Within each country, estimates are contrasted across rural, peri-urban and urban areas; panels correspond to countries.

Table 9: Predicted prevalence (%) of technology-facilitated CSEA by degree of urbanisation (rural, peri-urban, urban), with 95% Bayesian credible intervals. Values are posterior means from the survey-weighted Bayesian regression, marginalised over covariates in the fitted model (e.g. age, gender). Each row shows the posterior mean and 95% credible interval for a given country and degree of urbanisation.

COUNTRY	Degree of urbanization	Mean %	95% CI
Cambodia	Rural	13.4%	[11.3%, 15.7%]
	Peri urban	17.5%	[14.5%, 21.1%]
	Urban	15.6%	[13.2%, 18.4%]
Ethiopia	Rural	13.7%	[11.4%, 16.3%]
	Peri urban	17.9%	[14.2%, 21.5%]
	Urban	16.0%	[13.2%, 18.9%]
Indonesia	Rural	6.1%	[4.6%, 7.6%]
	Peri urban	8.1%	[6.1%, 10.4%]
	Urban	7.2%	[5.5%, 9.0%]
Kenya	Rural	20.3%	[17.8%, 22.9%]
	Peri urban	25.9%	[22.0%, 30.4%]
	Urban	23.4%	[20.6%, 26.6%]
Malaysia	Rural	9.1%	[7.3%, 10.9%]
	Peri urban	12.1%	[9.7%, 14.7%]
	Urban	10.7%	[8.8%, 12.9%]
Mozambique	Rural	23.4%	[20.8%, 26.2%]
	Peri urban	29.6%	[25.3%, 34.0%]
	Urban	26.8%	[23.7%, 29.7%]
Namibia	Rural	18.0%	[15.7%, 20.6%]
	Peri urban	23.2%	[19.4%, 27.3%]
	Urban	20.8%	[18.2%, 23.6%]
Philippines	Rural	27.0%	[24.0%, 29.9%]
	Peri urban	33.7%	[29.0%, 38.4%]
	Urban	30.7%	[27.3%, 34.0%]
Tanzania	Rural	6.9%	[5.3%, 8.5%]
	Peri urban	9.2%	[7.1%, 11.9%]
	Urban	8.2%	[6.4%, 10.0%]
Thailand	Rural	15.9%	[13.6%, 18.2%]
	Peri urban	20.7%	[17.0%, 24.4%]
	Urban	18.5%	[15.7%, 21.2%]
Uganda	Rural	23.1%	[20.5%, 26.1%]
	Peri urban	29.1%	[24.5%, 33.7%]
	Urban	26.4%	[23.1%, 29.7%]
	Rural	5.3%	[4.1%, 6.7%]

Vietnam	Peri urban	7.1%	[5.3%, 9.3%]
	Urban	6.2%	[4.7%, 7.9%]

3 Disclosure Analysis

3.1 Disclosure by Types of Technology-facilitated CSEA

For each of the nine technology-facilitated CSEA types, respondents indicated all disclosure targets to whom they had told about the experience. This item was multi-select: respondents could choose all applicable targets and were not asked to pick a single option or rank a first disclosure. We constructed three non-mutually-exclusive composite outcomes: (i) any disclosure (disclosed to at least one target); (ii) informal disclosure (family, friends, or other trusted adults); and (iii) formal disclosure (teacher, police, helpline, or social worker). Children selecting only "didn't tell anyone," "prefer not to say," or "don't know" were coded as non-disclosures. Because respondents could select multiple targets, children who disclosed to both formal and informal channels contribute to both outcomes, and category proportions do not sum to 100%. This coding reflects three considerations. First, each disclosure decision may be influenced by distinct factors (e.g., parental mediation for family disclosures; helpseeking knowledge for formal disclosures). Second, this follows standard practice for modelling multiple non-mutually-exclusive behavioural outcomes. Third, our interest was in predictors of disclosure to specific channels, rather than forcing mutually exclusive pathways.

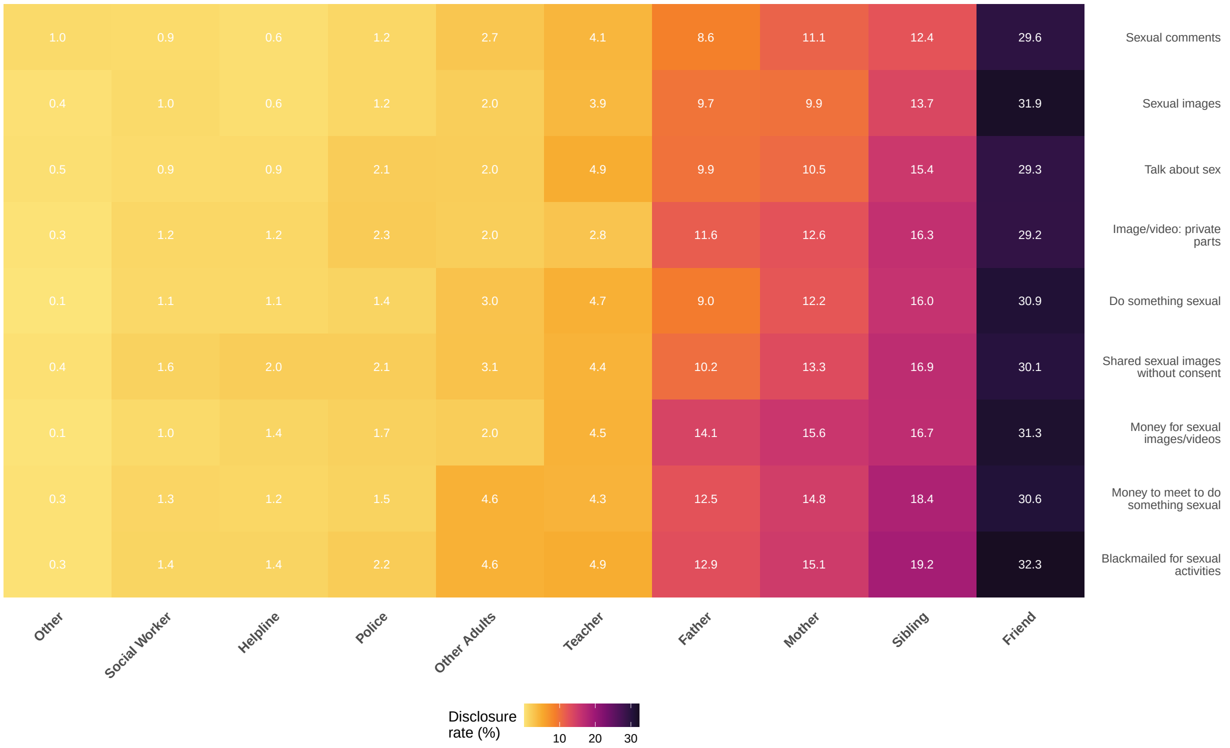

Figure 24: Survey-weighted percentage of children who experienced technology-facilitated CSEA and disclosed, by harm type and disclosure channel (2020–2021). Each cell represents the proportion of children who experienced that specific harm type and disclosed to that channel. Rows represent the nine CSEA types (a–i); columns represent disclosure channels across informal (parents, siblings, friends, other adults) and formal (police, helpline, social worker, teacher) categories. Percentages are non-additive as children could disclose to multiple channels.

3.2 Mutually Exclusive Categories of Disclosure

To complement the non-mutually-exclusive composites described above, we constructed a four-category classification: no disclosure, informal only, formal only, and both. These categories sum to 100% within each country, allowing direct comparison of children who disclosed via a single route versus both. The any-disclosure indicator (logical OR across all channels) is overlaid for reference.

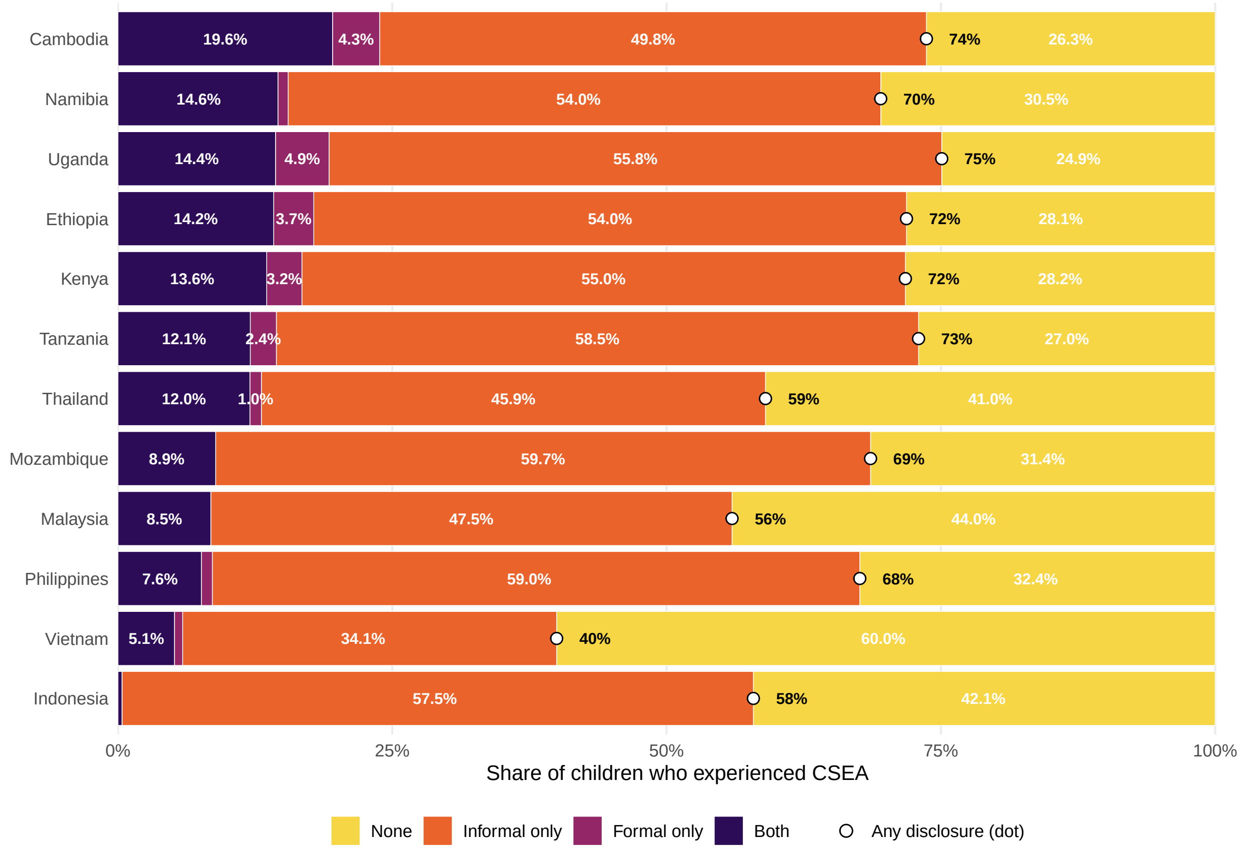

Figure 25: Mutually exclusive disclosure categories by country. Bars show the proportion of children who experienced CSEA in each category (none, informal only, formal only, both) and sum to 100% within each country. Dots indicate the any-disclosure proportion (logical OR across all channels); percentage labels show values. Countries are ordered by the proportion disclosing through both routes.

3.3 Disclosure Barriers Full Co-occurrence Matrix

We conducted a survey-weighted co-occurrence analysis of the 13 non-disclosure barriers among incidents where children reported telling no one. Each non-disclosed incident (child × CSEA type combination) was coded for which barriers were reported. The analysis is presented as a 13 × 13 heat map, where diagonal cells show the weighted prevalence of each individual barrier and off-diagonal cells show the weighted joint prevalence of barrier pairs.

Among non-disclosed incidents (weighted N = 2751; unweighted N = 2,921), 9.6% selected no barrier, 74.6% cited a single barrier, 13.3% cited two or three, and 2.5% cited four or more. The most prevalent individual barriers were "did not know where to go/who to tell" (37.6%), "embarrassed/ashamed/too difficult" (19.6%), and "did not think it was serious enough" (14.2%).

The most common two-barrier combination was "embarrassed/ashamed/too difficult" and "did not know where to go/who to tell" (2.7% of non-disclosed incidents), followed by "embarrassed/ashamed/too difficult" and "worried I would get in trouble" (1.8%). Among children citing "embarrassed/ashamed/too difficult," 14.0% also cited "did not know where to go/who to tell"; conversely, among those citing "did not know where to go/who to tell," 7.3% also cited "embarrassed/ashamed/too difficult."

Importantly, barrier co-occurrence patterns indicate substantial heterogeneity in children's experiences. Rather than collapsing into a single profile, barriers reflect distinct obstacles: informational (e.g., not knowing where to go), emotional (e.g., shame), and anticipatory concerns about consequences (e.g., fear of consequences). This heterogeneity suggests that disclosure support may require strategies tailored to the specific combinations of barriers relevant to children's circumstances.

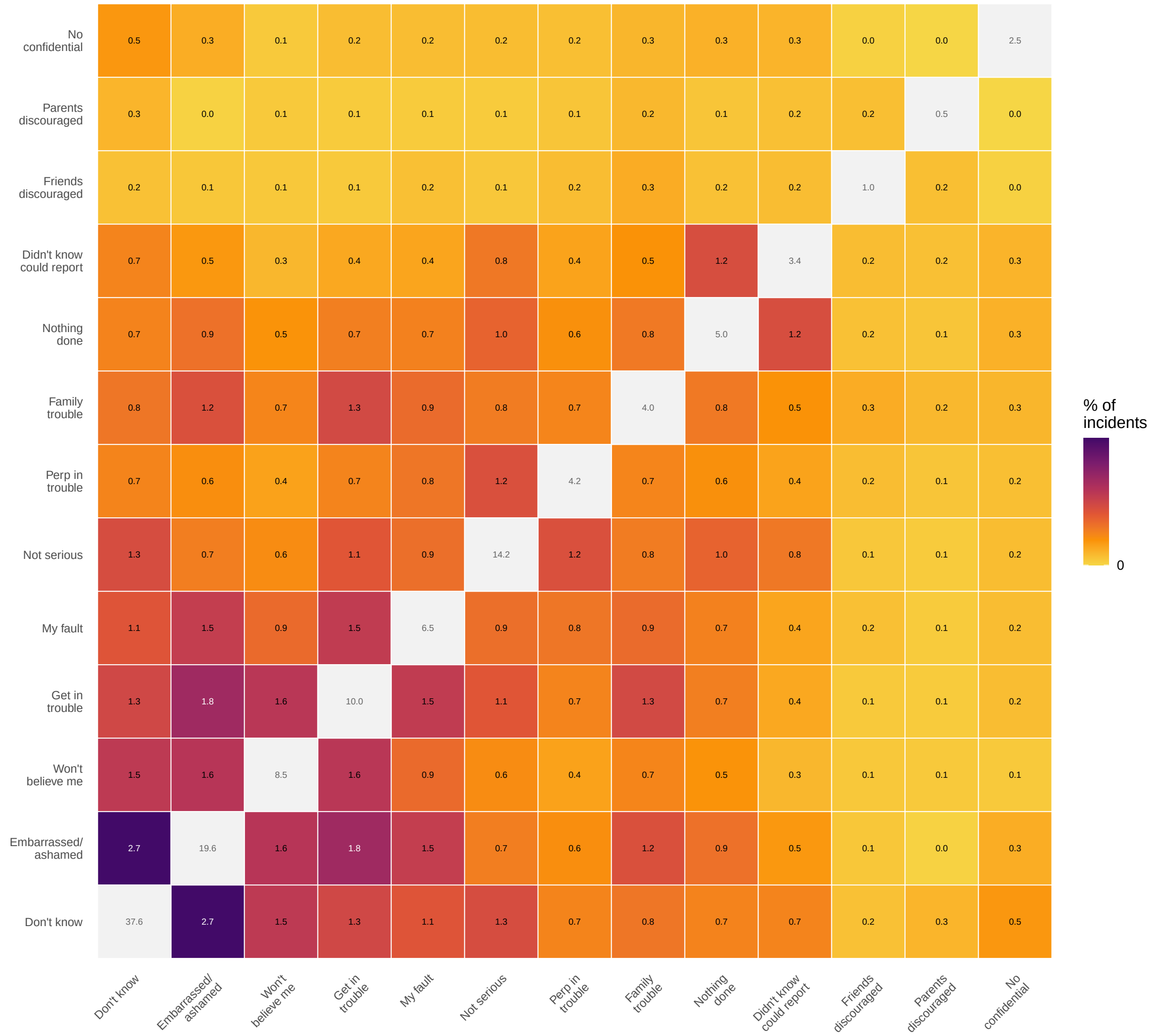

Figure 26: Co-occurrence of barriers to disclosure among non-disclosed incidents (N = 2,921 incidents across 12 countries). Diagonal cells show individual barrier prevalence; off-diagonal cells show the weighted percentage of incidents in which both barriers were cited. All estimates are survey-weighted.

3.4 Disclosure Inferential Analysis Overview

Note on Preregistration: Prior to conducting any data analysis, we developed a detailed preregistration document (authors: S.G, M.V and A.O) outlining our hypotheses, key variables, and analytic strategies. This document was uploaded to the OSF repository on April 15, 2022, and is archived in the project's storage as Secondary Pre-registration_The extent of reporting.pdf. While the preregistration was not formally submitted through a structured registry, a detailed pre-analysis plan was completed in advance, time-stamped, and archived on the OSF repository See metadata

Models: The preregistration specified one confirmatory logistic regression model for testing our primary hypothesis and outlined several exploratory approaches, including: (a) interaction terms, (b) multilevel models with random intercepts, (c) comparisons between formal and informal disclosure channels, and (d) regularised regression models. We transparently disclose all deviations and provide results for both preregistered and extended models.

To examine the relationship between demographic and socio-cultural factors and the likelihood of disclosure, we implemented a range of statistical models with varying specifications. These included both confirmatory and exploratory models, incorporating different predictor combinations and disclosure outcomes (overall, formal, informal). We used multiple imputation to handle missing data and applied multilevel models with random intercepts and random slopes to account for country-level heterogeneity. Regularised regression models served as robustness checks. This modelling strategy aimed to ensure consistency of results across specifications and improve interpretability. Each model is clearly labeled with its registration status and role within the analytical framework, with full specifications, diagnostics, and posterior summaries provided.

Table 10: Overview of the Bayesian logistic regression models fitted for the disclosure analysis. Columns indicate model description, random-effects structure, and pre-registrated analysis status. All models use a Bernoulli likelihood with logit link and survey probability weights. Multiple imputation (MI) used M = 30 imputed datasets via brm_multiple.

Model	Description	Random Effects	Pre-registered
1. Logistic Regression (Complete Case)	Confirmatory analysis. Baseline model with no imputation or random effects.	None	Yes
2. Logistic Regression (MI)	Confirmatory analysis. Handles missing data via multiple imputation (MI).	None	Yes
3. Random Intercepts Model (Complete Case)	Exploratory sensitivity model. Accounts for country-level clustering without imputation.	Intercept only	Yes
4. Random Intercepts Model (MI)	Exploratory sensitivity model. Controls for country-level clustering with random intercepts.	Intercept only	Yes
5. Random Intercepts + Slopes Model (MI)	Main reported model. Exploratory analysis allowing intercepts and slopes to vary by country.	Intercepts + Slopes	No
6. Regularised Regression (MI, Intercepts Only)	Exploratory robustness check. Pre-registered as penalised regression (LASSO); implemented as Bayesian regularisation with horseshoe prior.	Intercept only	Yes*
7. Regularised Regression (MI, Slopes & Intercepts)	Exploratory robustness extension. Adds random slopes to regularised model.	Intercepts + Slopes	No
8. Random Slopes + Age*Sex Interaction (MI)	Exploratory extension testing variation by age and sex across countries. *Partial: interaction terms were pre-registered but random slopes specification was not	Intercepts + Slopes	Partial*
9. Channel-specific Logistic Regressions (Complete Case)	Exploratory per-channel models (police, helpline, social worker, teacher, family, peer, other). Each treated as an independent binary outcome; random intercepts only; complete-case analysis.	Intercept only	Yes
10. Random Intercepts + Single Random Slope (PSIS–LOO)	Sensitivity analysis: fit separate models allowing one predictor's slope to vary by country; out-of-sample fit assessed with PSIS–LOO.	Intercept + single slope (per predictor)	No

4 Disclosure Inferential Analysis: Complete Case

4.1 Investigate Missingness

The primary disclosure models were initially estimated using complete cases. To address potential bias from missing data, we re-estimated all models using multiple imputation methods. We generated M = 30 imputed datasets, fitted the Bayesian multilevel logistic regression separately to each, and pooled results to obtain final estimates, thereby propagating imputation uncertainty into the inference. The extent of missingness in key variables is summarised below.

Table 11: Summary of missingness for key variables

Predictor	N Missing	% Missing
Inequitable gender attitudes	698	33.8%
Premarital sex	301	14.6%
Digital skills to report	118	5.7%
Help-seeking	108	5.2%
Sex education	98	4.7%
Enabling parental mediation	5	0.2%
Sex	0	0.0%
Age	0	0.0%

4.2 Pre-Registered Confirmatory Analysis: Logistic Regression

A Bayesian logistic regression was fitted to examine predictors of disclosure. Survey weights were applied. The model was estimated on complete cases without imputation or random effects.

Table 12: Posterior means, 95% credible intervals, and odds ratios from the pre-registered Bayesian logistic regression model predicting disclosure of technology-facilitated CSEA to any channel. No country-level random effects; fit on complete-case analytic sample.

Parameter	Estimate	Lower CI	Upper CI	Odds Ratio	Lower OR CI	Upper OR CI
Girls (vs Boys)	0.302	0.034	0.569	1.35	1.03	1.77
Age (per year)	-0.115	-0.205	-0.025	0.89	0.81	0.98
Premarital sex	0.137	-0.155	0.427	1.15	0.86	1.53
Inequitable gender attitudes	0.306	0.168	0.446	1.36	1.18	1.56
Enabling parental mediation	0.063	-0.070	0.196	1.06	0.93	1.22
Sex education	-0.215	-0.488	0.053	0.81	0.61	1.05
Help-seeking	0.346	0.069	0.621	1.41	1.07	1.86
Digital skills to report	-0.097	-0.208	0.013	0.91	0.81	1.01

4.3 Pre-Registered Exploratory Analysis: Random Intercepts Model

This Bayesian multilevel logistic regression accounts for the hierarchical structure of the data, with children nested within countries. Random intercepts for country allow the model to adjust for baseline differences in disclosure rates across the 12 study countries.

Table 13: Posterior means and 95% credible intervals (log-odds scale) from Bayesian multilevel logistic regression models predicting disclosure of technology-facilitated CSEA to any, formal, and informal channels. Models include random intercepts for country only, fit on the complete-case analytic sample without multiple imputation. Intercept excluded from table.

predictor	All	Formal	Informal
Girls (vs Boys)	0.33 [0.07, 0.60]	0.29 [-0.10, 0.69]	0.24 [-0.02, 0.50]
Age (per year)	-0.14 [-0.24, -0.05]	-0.24 [-0.37, -0.12]	-0.13 [-0.23, -0.04]
Premarital sex	0.23 [-0.09, 0.55]	-0.23 [-0.70, 0.23]	0.28 [-0.03, 0.60]
Inequitable gender attitudes	0.30 [0.15, 0.44]	0.18 [-0.03, 0.37]	0.30 [0.16, 0.45]
Enabling parental mediation	0.19 [0.03, 0.36]	0.30 [0.08, 0.52]	0.17 [0.00, 0.33]
Sex education	-0.15 [-0.44, 0.15]	0.18 [-0.23, 0.60]	-0.10 [-0.39, 0.19]
Help-seeking	0.33 [0.06, 0.62]	-0.13 [-0.52, 0.27]	0.39 [0.12, 0.66]
Digital skills to report	-0.01 [-0.14, 0.11]	-0.02 [-0.20, 0.15]	-0.05 [-0.17, 0.07]

4.4 Multicollinearity Diagnostics

4.4.1 Correlations Between Predictors

To assess potential multicollinearity among predictors, we examined pairwise correlations between all variables included in the disclosure models. Correlations were computed on standardised predictors using survey weights. Pairwise associations were modest: the largest positive correlation was 0.28 (premarital sex attitudes with inequitable gender attitudes), and the largest negative correlation was −0.15 (age with parental mediation). No pair of predictors exceeded $r = 0.30$, indicating that multicollinearity among model covariates was minimal.

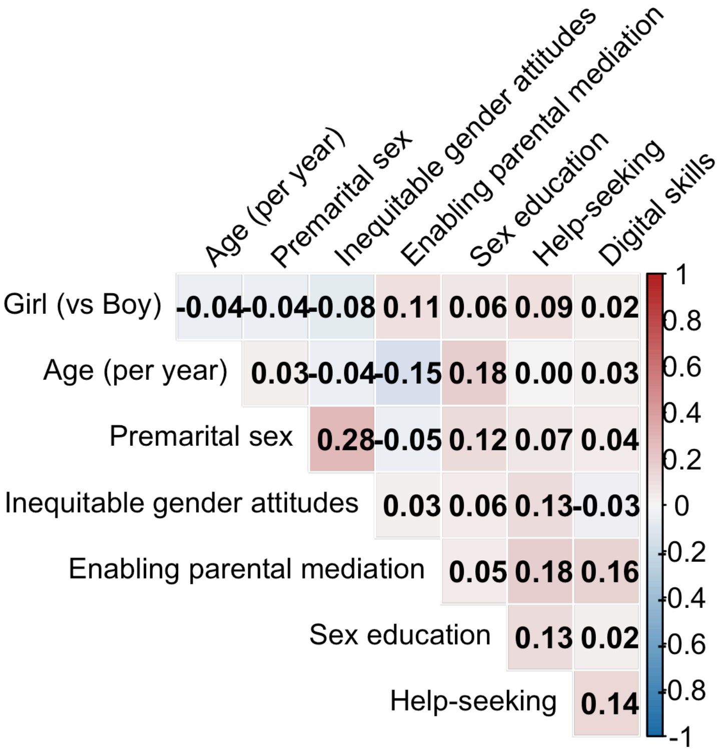

Figure 27: Weighted Pearson correlations among predictors used in the disclosure models (N = 1149 complete cases). Cells show pairwise correlations computed on standardised predictors with survey weights applied. Diagonal cells are omitted.

4.4.2 Variance Inflation Factors

We further computed variance inflation factors (VIFs) for all predictors in each disclosure model, using the complete-case dataset. All predictors enter with one degree of freedom. To provide a scale-free summary we also report Inflation which is the square root of VIF. Across models, VIFs were 1-1.2 ($\sqrt{\texttt{VIF}}$ =1.0-1.1), well below common concern thresholds (~2–5), indicating minimal collinearity.

Table 14: Variance inflation factors (VIF) and their square roots ($\sqrt{\text{VIF}}$) for all predictors across disclosure models predicting any, formal, and informal disclosure. Computed on the complete-case analytic sample. All predictors enter with one degree of freedom. VIF values of 1–1.2 ($\sqrt{\text{VIF}}$ = 1.0–1.1) indicate minimal multicollinearity across all models.

Outcome	Term	VIF	$\sqrt{\text{VIF}}$
Any channels	Premarital sex	1.1	1.1
Any channels	Inequitable gender attitudes	1.1	1.0
Any channels	Sex education	1.1	1.0
Any channels	Enabling parental mediation	1.1	1.0
Any channels	Help-seeking	1.1	1.0
Any channels	Digital skills to report	1.1	1.0
Any channels	Age (per year)	1.1	1.0
Any channels	Girls (vs Boys)	1.0	1.0
Formal channels	Premarital sex	1.2	1.1
Formal channels	Inequitable gender attitudes	1.1	1.1
Formal channels	Help-seeking	1.1	1.1
Formal channels	Enabling parental mediation	1.1	1.1
Formal channels	Sex education	1.1	1.0
Formal channels	Digital skills to report	1.1	1.0
Formal channels	Age (per year)	1.1	1.0
Formal channels	Girls (vs Boys)	1.0	1.0
Informal channels	Premarital sex	1.1	1.1
Informal channels	Sex education	1.1	1.0
Informal channels	Enabling parental mediation	1.1	1.0
Informal channels	Inequitable gender attitudes	1.1	1.0
Informal channels	Help-seeking	1.1	1.0
Informal channels	Digital skills to report	1.1	1.0
Informal channels	Age (per year)	1.1	1.0
Informal channels	Girls (vs Boys)	1.0	1.0

4.4.3 R2

To contextualise overall explanatory power, we report Bayesian R^2 (posterior mean with 95% credible interval) and Nakagawa's marginal R^2 (variance explained by fixed effects) and conditional R^2 (variance explained by both fixed and random effects) for each disclosure model. As expected for behavioural disclosure outcomes, overall explanatory power was modest. The gap between marginal and conditional R^2 indicates meaningful between-country heterogeneity captured by the random intercepts.

Table 15: Bayesian R^2 (posterior mean and 95% credible interval) and Nakagawa's marginal and conditional R^2 for Bayesian logistic regression models predicting disclosure of technology-facilitated CSEA to any, formal, and informal channels. Marginal R^2 reflects variance explained by fixed effects only; conditional R^2 reflects variance explained by both fixed and random effects. Models fit on complete-case analytic sample without multiple imputation.

Outcome	Bayesian R^2	Lower	Upper	Marginal R^2	Conditional R^2
Any channels	0.06	0.02	0.10	0.12	0.23
Formal channels	0.05	0.02	0.09	0.09	0.19
Informal channels	0.05	0.01	0.09	0.12	0.18

4.5 Channel Specific Regressions

To provide granular insight into predictors of disclosure through specific pathways, we conducted supplementary analyses examining each disclosure channel separately (mother, father, sibling, friend, teacher, police, helpline, social worker, other adults). These channel-specific models used identical predictor sets to the main analyses but examined each channel independently, allowing us to identify which factors distinguish disclosure to one particular channel versus non-disclosure to that channel.

Statistical Power Assessment: We first evaluated statistical power for each channel using events-per-variable (EPV) criteria. We excluded one channel (other: n=19, 0.9%, EPV=2.1) from regression analysis due to insufficient events. Three formal channels (police: n=59, helpline: n=63, social worker: n=58; EPV=6.4–7.0) have limited power and should be interpreted as exploratory findings. All other channels met conventional power thresholds (EPV>10).

The channel-specific analyses reveal several consistent patterns. Age shows a broadly negative association with disclosure across most channels, suggesting lower odds among older adolescents regardless of pathway. Gender associations are channel-dependent: girls have higher odds of disclosure for most channels but lower odds to fathers, pointing to gendered dynamics in parent–child communication. Parental mediation is positively associated with disclosure across both informal and formal pathways, whereas more positive attitudes toward premarital sex are associated primarily with disclosure to family and friends. Help-seeking knowledge is associated with higher odds of disclosure overall without strong differentiation by specific formal service. Sex education shows a specific association with disclosure to teachers, suggesting the school context may be particularly relevant for that disclosure pathway, though this cannot be taken as causal evidence.

Channel-specific models used complete case analysis (no multiple imputation) and random intercepts only (no random slopes), reflecting the simpler models needed given reduced sample sizes when disaggregating by channel. These methodological differences mean that estimates are not directly comparable to the primary models reported in the main manuscript, which used multiple imputation and random slopes. Channel-specific results should therefore be read as supplementary, within-channel associations that highlight heterogeneity across disclosure pathways.

Table 16: Channel-specific power (events-per-variable, EPV) and inclusion notes. Counts are complete-case. EPV = events ÷ predictors.

Channel	N disclosed	N total	Prevalence (%)	EPV	Analysis
Friend	956	2,067	46.3	106.2	Regression
Sibling	516	2,067	25.0	57.3	Regression
Mother	420	2,067	20.3	46.7	Regression
Father	384	2,067	18.6	42.7	Regression
Teacher	179	2,067	8.7	19.9	Regression
Other Adult	130	2,067	6.3	14.4	Regression
Helpline	63	2,067	3.0	7.0	Regression (exploratory)
Police	59	2,067	2.9	6.6	Regression (exploratory)
Social Worker	58	2,067	2.8	6.4	Regression (exploratory)
Other	19	2,067	0.9	2.1	Descriptive only

Table 17: Odds ratios and 95% credible intervals from Bayesian logistic regression models predicting disclosure to any, formal, and informal channels following technology-facilitated CSEA. Separate models fit per channel on the complete-case analytic sample without multiple imputation or country-level random effects. Bold values indicate 95% CI excludes zero; † denotes OR ≥ 1.5 or ≤ 0.67. Not directly comparable to main manuscript models.).

Predictors	Any disclosure channel	Formal disclosure channel	Informal disclosure channel
Girls (vs Boys)	**1.40 (1.07–1.83)**	1.34 (0.90–1.99)	1.27 (0.98–1.64)
Age (per year)	**0.87 (0.79–0.95)**	**0.79 (0.69–0.89)**	**0.87 (0.80–0.96)**
Premarital sex	1.26 (0.92–1.73)	0.79 (0.50–1.25)	1.32 (0.97–1.83)
Inequitable gender attitudes	**1.35 (1.16–1.56)**	1.19 (0.97–1.45)	**1.35 (1.17–1.57)**
Enabling parental mediation	**1.21 (1.03–1.43)**	**1.35 (1.08–1.68)**	**1.18 (1.00–1.39)**
Sex education	0.86 (0.64–1.16)	1.20 (0.80–1.82)	0.90 (0.68–1.21)
Help-seeking	**1.40 (1.06–1.86)**	0.88 (0.59–1.31)	**1.47 (1.13–1.93)**
Digital skills to report	0.99 (0.87–1.12)	0.98 (0.82–1.16)	0.95 (0.84–1.07)

Table 18: Channel-specific predictors of disclosure across formal channels (odds ratios with 95% CIs; log scale).

Predictors	Police	Helpline	Social worker	Teacher
Girls (vs Boys)	1.12 (0.57–2.17)	0.84 (0.41–1.70)	0.95 (0.45–2.04)	**1.73 (1.06–2.85)†**
Age (per year)	**0.77 (0.63–0.94)**	**0.72 (0.56–0.90)**	**0.78 (0.61–1.00)**	0.88 (0.75–1.04)
Premarital sex	0.47 (0.19–1.07)	0.89 (0.37–2.05)	1.37 (0.58–3.18)	0.69 (0.37–1.24)
Inequitable gender attitudes	1.38 (0.99–1.92)	1.18 (0.83–1.69)	1.00 (0.65–1.49)	1.24 (0.97–1.60)
Enabling parental mediation	1.39 (0.96–1.99)	1.18 (0.80–1.78)	1.25 (0.84–1.89)	1.22 (0.93–1.61)
Sex education	0.70 (0.34–1.40)	1.61 (0.72–3.60)	0.47 (0.20–1.12)	**2.50 (1.41–4.58)†**
Help-seeking	0.79 (0.38–1.56)	0.52 (0.24–1.10)	0.83 (0.36–1.85)	0.99 (0.61–1.64)
Digital skills to report	0.81 (0.60–1.07)	0.86 (0.62–1.19)	1.18 (0.85–1.63)	0.95 (0.75–1.18)

Table 19: Channel-specific predictors of disclosure across informal channels (odds ratios with 95% CIs; log scale).

Predictors	Mother	Father	Sibling	Friend	Other adult
Girls (vs Boys)	1.16 (0.85–1.60)	**0.68 (0.50–0.93)**	1.27 (0.96–1.71)	**1.36 (1.06–1.76)**	1.02 (0.59–1.78)
Age (per year)	**0.76 (0.69–0.84)**	**0.83 (0.75–0.92)**	**0.86 (0.78–0.95)**	0.97 (0.89–1.06)	**0.83 (0.71–0.99)**
Premarital sex	1.05 (0.72–1.48)	**1.59 (1.09–2.28)†**	**1.47 (1.08–2.03)**	**1.33 (1.01–1.76)**	1.38 (0.75–2.49)
Inequitable gender attitudes	**1.36 (1.16–1.59)**	**1.33 (1.14–1.57)**	**1.39 (1.20–1.60)**	**1.30 (1.14–1.49)**	1.19 (0.91–1.54)
Enabling parental mediation	**1.29 (1.08–1.54)**	**1.23 (1.03–1.47)**	1.15 (0.98–1.34)	0.98 (0.85–1.13)	1.20 (0.90–1.59)
Sex education	1.32 (0.93–1.85)	0.96 (0.68–1.35)	1.25 (0.92–1.72)	1.19 (0.91–1.55)	1.06 (0.59–1.86)
Help-seeking	1.14 (0.83–1.57)	1.20 (0.87–1.70)	1.18 (0.89–1.57)	**1.54 (1.20–2.00)†**	0.68 (0.39–1.22)
Digital skills to report	1.05 (0.91–1.21)	**0.77 (0.66–0.88)**	1.10 (0.97–1.24)	1.06 (0.95–1.19)	0.96 (0.76–1.21)

4.6 Forest Plot for Channel Specific Predictors

To visualise the magnitude and direction of predictor associations across disclosure channels, we present odds ratios with 95% confidence intervals from the channel-specific logistic regressions reported above.

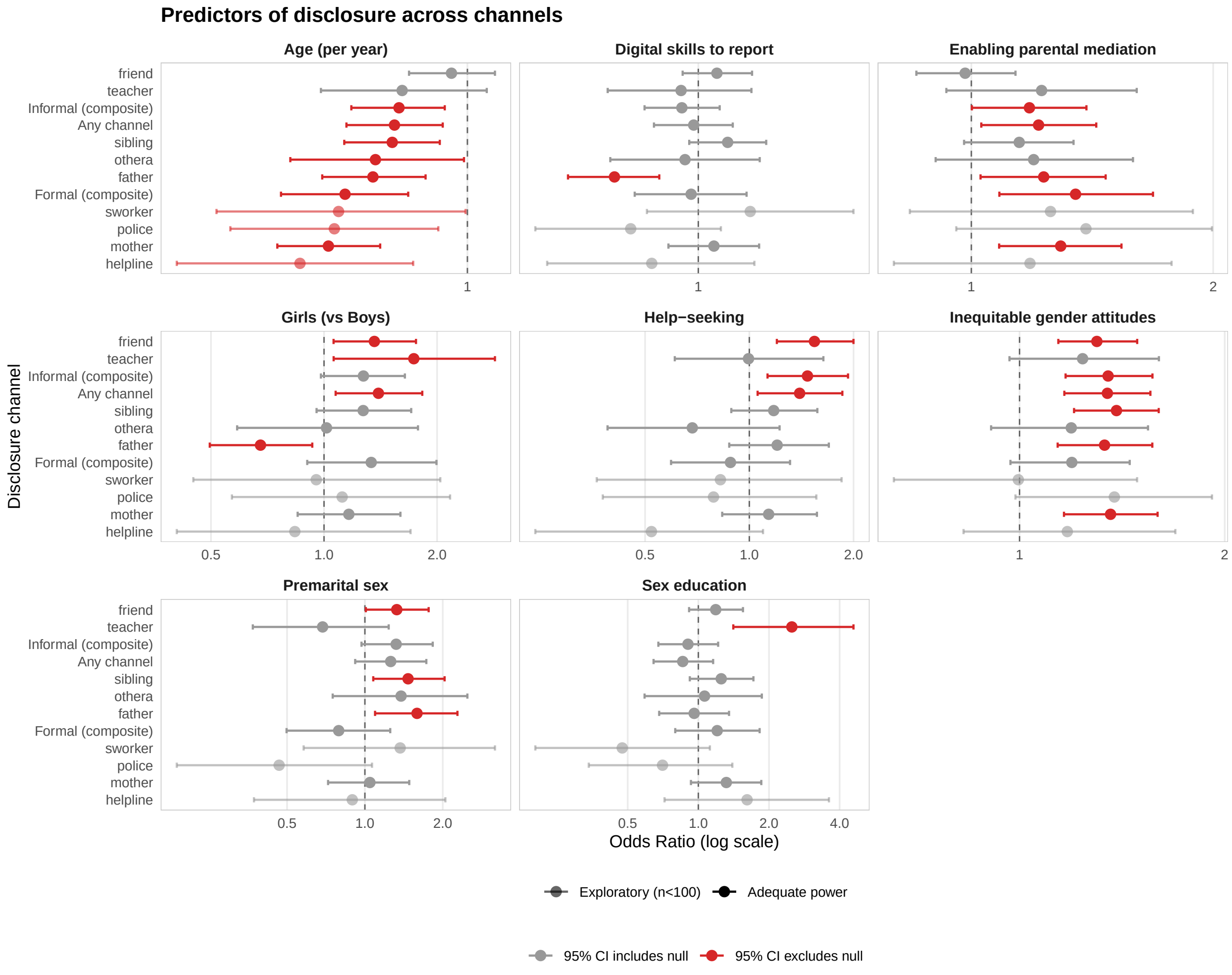

Figure 28: Forest plot of odds ratios and 95% credible intervals for all predictors across individual disclosure channels. Each panel shows estimates from a separate Bayesian logistic regression model fit on the complete-case analytic sample. Vertical dashed line at OR = 1 indicates the null.

4.7 Sensitivity Analysis: Predictors Varying by Random Slopes

Using Pareto-smoothed importance-sampling leave-one-out cross-validation (PSIS-LOO), we assessed whether allowing any single predictor's slope to vary by country improves predictive performance over a random-intercepts-only specification. All models showed acceptable diagnostics, with maximum Pareto k diagnostic values ranging from 0.28 to 0.53 and no values exceeding the 0.7 threshold indicating unreliable importance weights.

ΔELPD relative to the best-fitting model ranged from -3.1 to 0.0, with standard errors of 3.4–4.3 for non-reference models, placing all differences within one SE of the best-fitting model and indicating no supported predictive gain from allowing any single slope to vary by country. The model allowing inequitable gender attitudes to vary by country as a random slope attained the highest ELPD (ΔELPD = 0.0, reference). The fixed-slope baseline performed 1.6 ELPD units worse (ΔELPD = -1.6, SE = 3.4), and the next closest was the random-slope model for age (ΔELPD = -0.9, SE = 4.2), both well within one SE of the best-fitting model. These comparisons therefore suggest that country differences are well captured by random intercepts, while slopes are broadly similar across settings in out-of-sample terms.

This analysis used the complete-case dataset rather than the multiply imputed data. Because multiple imputation partially pools information across countries, it could artificially reduce or inflate evidence for cross-national slope heterogeneity. The complete-case approach avoids this potential confound, ensuring that observed patterns reflect genuine between-country variation rather than artefacts of the imputation model.

Table 20: Associations between different predictors varying by random slopes and the probability of disclosing technology-facilitated CSEA from a Bayesian logistic regression. ΔELPD: difference in expected log pointwise predictive density (model predictive performance).

Model	ΔELPD	SE
Inequitable gender attitudes	0.0	0.0
Age (per year)	-0.9	4.2
Sex education	-1.1	4.3
Full model	-1.6	3.4
Digital skills to report	-1.9	3.6
Premarital sex	-2.1	3.9
Girl (vs Boy)	-2.6	3.6
Enabling parental mediation	-2.8	3.9
Help-seeking	-3.1	3.4

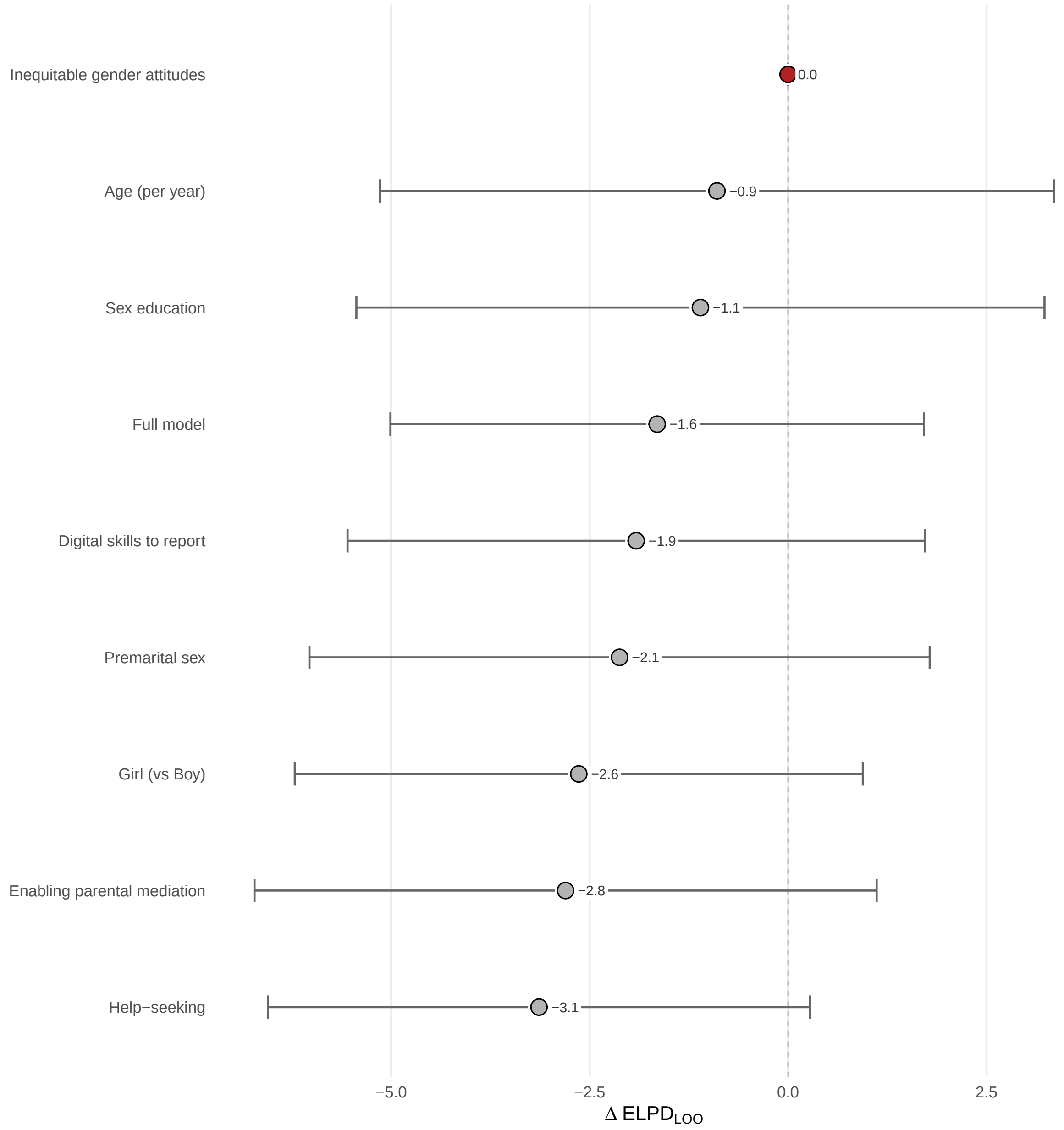

Figure 29: Model comparison using PSIS-LOO. Points show the difference in expected log predictive density (ΔELPD) relative to the best-fitting model (marked in red; higher = better; 0 = best). Negative values indicate worse predictive performance. Error bars show ±1 SE of the difference. Across the baseline and predictor-specific random-slope models, ΔELPD values are small (0.0 to −3.1, SE 3.4–4.3), placing all models within 1 SE of the best and therefore LOO-comparable. The random-slope model for inequitable gender attitudes attained the highest ELPD, but provides no supported improvement over the fixed-slope baseline.

5 Disclosure Inferential Analysis: Multiple Imputations

5.1 Pre-Registered Confirmatory Analysis: Logistic Regression Model

As pre-registered, we first fitted a Bayesian logistic regression model without random effects to predict any disclosure from gender, age, attitudes towards premarital sex, inequitable gender attitudes, enabling parental mediation, sex education, help-seeking knowledge and digital skills, using multiply imputed data (M = 30).

Table 21: Posterior means and 95% credible intervals from a Bayesian logistic regression model predicting disclosure of technology-facilitated CSEA, fit via brm_multiple across M = 30 imputed datasets. Results are presented on both the log-odds scale and as exponentiated odds ratios with 95% credible intervals.

Parameter	Estimate	Lower CI	Upper CI	Odds Ratio	Lower OR CI	Upper OR CI
Girls (vs Boys)	0.147	-0.047	0.342	1.16	0.95	1.41
Age (per year)	-0.078	-0.141	-0.015	0.93	0.87	0.98
Premarital sex	0.226	-0.014	0.471	1.25	0.99	1.60
Inequitable gender attitudes	0.268	0.141	0.401	1.31	1.15	1.49
Enabling parental mediation	0.094	-0.008	0.196	1.10	0.99	1.22
Sex education	-0.158	-0.359	0.044	0.85	0.70	1.04
Help-seeking	0.342	0.140	0.545	1.41	1.15	1.72
Digital skills to report	-0.109	-0.194	-0.024	0.90	0.82	0.98

5.2 Pre-registered Exploratory Analysis: Random Intercepts Model

This is the imputed version of the pre-registered exploratory model, with random intercepts for country.

Table 22: Posterior means and 95% credible intervals (log-odds scale) from Bayesian multilevel logistic regression models predicting disclosure of technology-facilitated CSEA, fit via brm_multiple across M = 30 imputed datasets. Results are shown for three outcomes: disclosure to any channel (All), formal channels only (Formal), and informal channels only (Informal). All models include random intercepts for country only (no random slopes).

predictor	All	Formal	Informal
Girls (vs Boys)	0.18 [-0.02, 0.38]	-0.07 [-0.34, 0.20]	0.15 [-0.04, 0.35]
Age (per year)	-0.12 [-0.19, -0.06]	-0.26 [-0.35, -0.18]	-0.09 [-0.16, -0.03]
Premarital sex	0.30 [0.04, 0.56]	-0.09 [-0.47, 0.28]	0.34 [0.09, 0.59]
Inequitable gender attitudes	0.25 [0.13, 0.39]	0.34 [0.16, 0.52]	0.22 [0.10, 0.35]
Enabling parental mediation	0.22 [0.11, 0.34]	0.31 [0.16, 0.46]	0.20 [0.09, 0.31]
Sex education	-0.15 [-0.37, 0.08]	0.08 [-0.22, 0.39]	-0.10 [-0.32, 0.12]
Help-seeking	0.35 [0.14, 0.56]	0.02 [-0.27, 0.30]	0.37 [0.17, 0.58]
Digital skills to report	-0.02 [-0.11, 0.07]	0.01 [-0.12, 0.14]	-0.06 [-0.15, 0.04]

5.3 Exploratory Analysis (Not Pre-Registered): Random Intercepts and Slopes Model (Main Reported Analysis)

This is the primary model reported in the manuscript section *"Demographic and Socio-Cultural Factors Associated with Disclosure."* It extends the previous random-intercepts model by including both random intercepts and random slopes for all predictors, allowing their effects to vary across countries. This specification captures cross-country differences not only in baseline disclosure rates but also in how demographic and socio-cultural factors relate to disclosure.Although not pre-registered, this model builds directly on the pre-registered random-intercepts specification and was included to better account for cross-country heterogeneity in the associations between predictors and disclosure.

Table 23: Posterior means and 95% credible intervals (log-odds scale) from Bayesian multilevel logistic regression models predicting disclosure of technology-facilitated CSEA, fit via brm_multiple across M = 30 imputed datasets. Results are shown for three outcomes: disclosure to any channel (All), formal channels only (Formal), and informal channels only (Informal). All models include random intercepts and slopes by country (adapt_delta = 0.99). The intercept excluded from table.

predictor	All	Formal	Informal
Girls (vs Boys)	0.13 [-0.19, 0.42]	-0.17 [-0.80, 0.45]	0.12 [-0.19, 0.40]
Age (per year)	-0.12 [-0.22, -0.03]	-0.27 [-0.41, -0.14]	-0.11 [-0.23, 0.01]
Premarital sex	0.29 [-0.03, 0.61]	-0.10 [-0.61, 0.38]	0.33 [0.02, 0.64]
Inequitable gender attitudes	0.23 [-0.02, 0.48]	0.27 [-0.02, 0.50]	0.20 [-0.04, 0.43]
Enabling parental mediation	0.22 [0.06, 0.38]	0.32 [0.11, 0.54]	0.19 [0.04, 0.35]
Sex education	-0.15 [-0.48, 0.16]	0.06 [-0.34, 0.44]	-0.08 [-0.44, 0.27]
Help-seeking	0.38 [0.11, 0.67]	0.03 [-0.40, 0.45]	0.40 [0.15, 0.66]
Digital skills to report	-0.03 [-0.18, 0.10]	0.02 [-0.15, 0.22]	-0.08 [-0.24, 0.07]

5.4 Correlations Among Predictors in the Exploratory Model

The plots below display posterior correlations among fixed-effect parameter estimates in the random intercepts and slopes models, separately for any, formal, and informal disclosure. These correlations are derived from the posterior variance-covariance matrix and indicate how uncertainty in one parameter estimate relates to uncertainty in another, rather than correlations among the predictor variables themselves

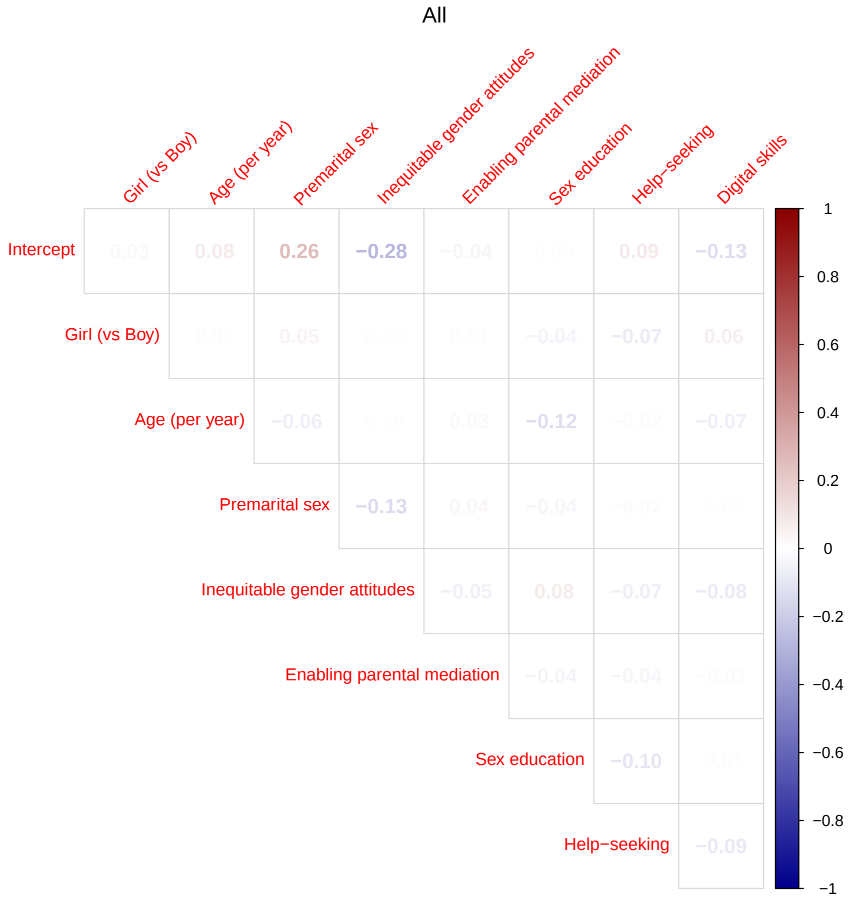

Figure 30: Posterior correlation matrices of fixed-effect parameter estimates from Bayesian multilevel logistic regression models (random intercepts and slopes by country) for any, formal, and informal disclosure outcomes. Correlations derived from the posterior variance-covariance matrix. Upper triangle shown; intercept excluded.

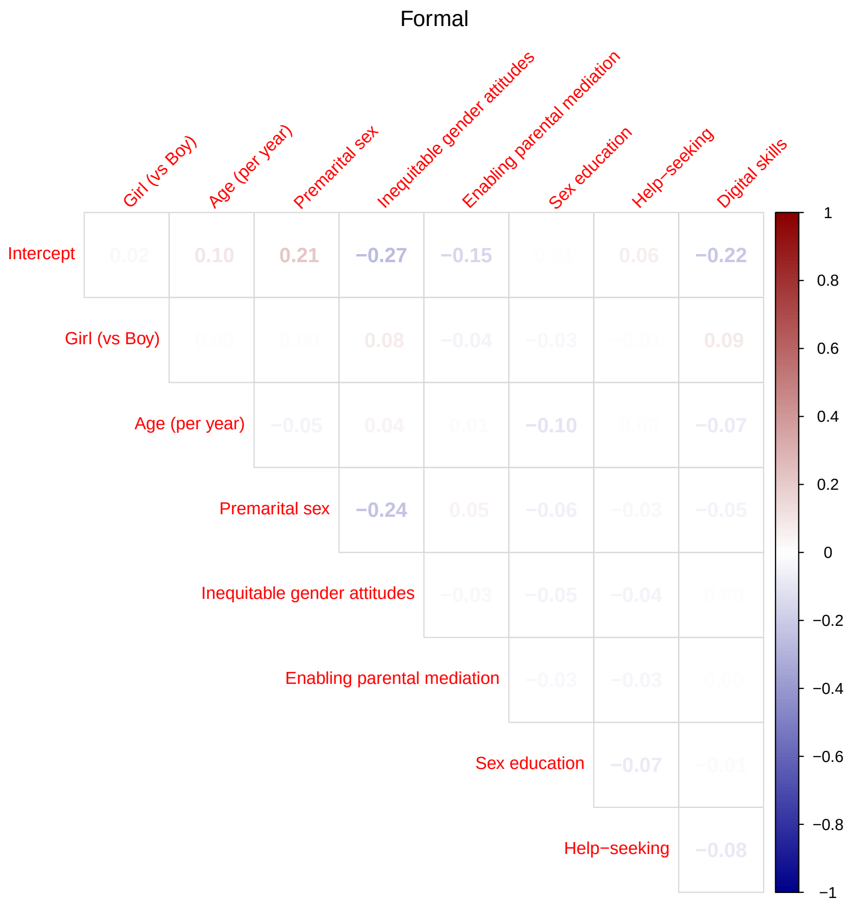

Figure 31: Posterior correlation matrices of fixed-effect parameter estimates from Bayesian multilevel logistic regression models (random intercepts and slopes by country) for any, formal, and informal disclosure outcomes. Correlations derived from the posterior variance-covariance matrix. Upper triangle shown; intercept excluded.

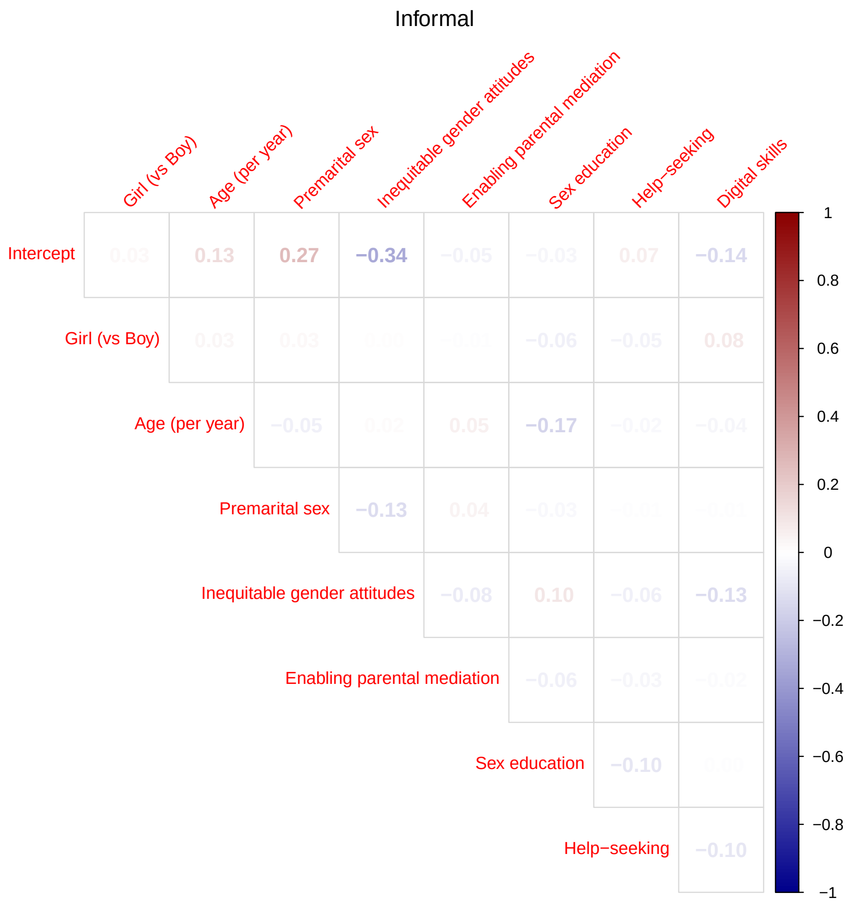

Figure 32: Posterior correlation matrices of fixed-effect parameter estimates from Bayesian multilevel logistic regression models (random intercepts and slopes by country) for any, formal, and informal disclosure outcomes. Correlations derived from the posterior variance-covariance matrix. Upper triangle shown; intercept excluded.

5.4.1 Leave-one-out cross-validation (disclosure models)

We further compare the all disclosure models with random intercepts only and models with both random intercepts and slopes by country. While the intercept-only model performs slightly better, the difference in predictive performance is not substantial.

Table 24: Leave-one-out cross-validation (PSIS-LOO comparison between Bayesian multilevel logistic regression models predicting disclosure of technology-facilitated CSEA to any channel: random intercepts only versus random intercepts and slopes by country. The reference model (ELPD difference = 0) is the better-fitting model; negative ELPD differences indicate worse predictive performance relative to the reference. Standard errors reflect uncertainty in the ELPD difference. PSIS-LOO computed via the loo package using Pareto-smoothed importance sampling.

Model	ELPD Difference	Standard Error
Random Intercepts	0.0	0.0
Random Intercepts & Slopes	-9.1	4.9

5.5 Pre-registered Exploratory Analysis: Random Intercepts, Slopes and Age-Gender Interactions

We extended the model by specifying random intercepts and random slopes by country for all predictors, including sex, age, and the sex × age interaction, thereby fitting a maximal random-effects structure. This goes beyond our pre-registered exploratory model, which included the age × gender interaction as a fixed effect but only random intercepts by country, with no random slopes.

Table 25: Posterior means and 95% credible intervals (log-odds scale) from a Bayesian multilevel logistic regression model predicting disclosure of technology-facilitated CSEA to any channel, including an age by sex interaction term. The model allows all predictor slopes to vary by country. Estimated via brm_multiple across M = 30 imputed datasets with adapt_delta = 0.99. The intercept excluded from table.

predictor	Interaction model
Girls (vs Boys)	0.13 [-0.18, 0.42]
Age (per year)	-0.12 [-0.23, -0.03]
Premarital sex	0.30 [-0.05, 0.65]
Inequitable gender attitudes	0.16 [-0.10, 0.39]
Enabling parental mediation	0.23 [0.07, 0.38]
Sex education	-0.15 [-0.47, 0.16]
Help-seeking	0.37 [0.10, 0.65]
Digital skills to report	-0.03 [-0.17, 0.10]
Age:Sex interaction	0.10 [-0.17, 0.34]

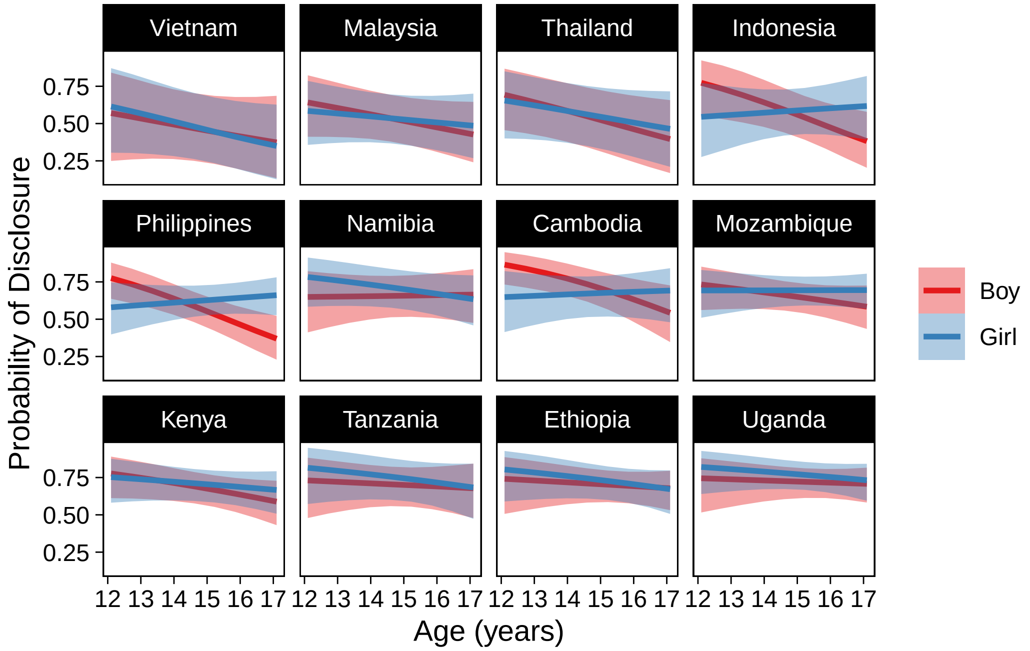

Figure 33: Predicted probability of disclosing technology-facilitated CSEA by age and sex, shown separately for each of the 12 study countries. Predictions derived from the Bayesian multilevel logistic regression model with age by sex interaction and country-specific random slopes. Shaded bands represent 95% credible intervals. Countries ordered by average predicted disclosure probability. Age shown on original scale (12–17 years)

Table 26: Predicted probabilities of disclosing technology-facilitated CSEA at ages 12 and 17, by sex and country, derived from the Bayesian multilevel logistic regression model with age by sex interaction and country-specific random slopes. Values represent posterior mean predicted probability and 95% credible interval bounds. Predictions are country-specific, reflecting random slope variation across the 12 study countries.

Country	Age	Sex	Estimate	Lower Bound	Upper Bound
Ethiopia	12	Boy	0.74	0.51	0.89
Ethiopia	12	Girl	0.80	0.59	0.93
Ethiopia	17	Boy	0.68	0.53	0.80
Ethiopia	17	Girl	0.67	0.51	0.80
Kenya	12	Boy	0.78	0.61	0.89
Kenya	12	Girl	0.75	0.58	0.88
Kenya	17	Boy	0.59	0.43	0.73
Kenya	17	Girl	0.67	0.51	0.79
Mozambique	12	Boy	0.73	0.56	0.85
Mozambique	12	Girl	0.69	0.51	0.83
Mozambique	17	Boy	0.58	0.43	0.73
Mozambique	17	Girl	0.69	0.57	0.81
Namibia	12	Boy	0.65	0.41	0.82
Namibia	12	Girl	0.78	0.58	0.91
Namibia	17	Boy	0.66	0.48	0.84
Namibia	17	Girl	0.63	0.46	0.79
Tanzania	12	Boy	0.73	0.48	0.88
Tanzania	12	Girl	0.82	0.57	0.95
Tanzania	17	Boy	0.68	0.48	0.84
Tanzania	17	Girl	0.68	0.47	0.84
Uganda	12	Boy	0.74	0.52	0.88
Uganda	12	Girl	0.82	0.64	0.93
Uganda	17	Boy	0.71	0.58	0.82
Uganda	17	Girl	0.73	0.59	0.84
Cambodia	12	Boy	0.87	0.73	0.95
Cambodia	12	Girl	0.65	0.41	0.82
Cambodia	17	Boy	0.54	0.35	0.73
Cambodia	17	Girl	0.69	0.48	0.84
Indonesia	12	Boy	0.77	0.55	0.92
Indonesia	12	Girl	0.55	0.28	0.77
Indonesia	17	Boy	0.38	0.20	0.58
Indonesia	17	Girl	0.62	0.39	0.82
Malaysia	12	Boy	0.64	0.41	0.82
Malaysia	12	Girl	0.58	0.36	0.79
Malaysia	17	Boy	0.43	0.24	0.65
Malaysia	17	Girl	0.49	0.27	0.70
Philippines	12	Boy	0.78	0.64	0.88
Philippines	12	Girl	0.58	0.40	0.75
Philippines	17	Boy	0.37	0.23	0.52
Philippines	17	Girl	0.66	0.53	0.78
Thailand	12	Boy	0.69	0.46	0.87
Thailand	12	Girl	0.65	0.40	0.85
Thailand	17	Boy	0.40	0.17	0.66
Thailand	17	Girl	0.46	0.21	0.72

Vietnam	12	Boy	0.57	0.25	0.84
Vietnam	12	Girl	0.61	0.30	0.87
Vietnam	17	Boy	0.37	0.13	0.69
Vietnam	17	Girl	0.35	0.13	0.63

5.6 Pre-registered Exploratory Analysis: Regularised Horseshoe Regression

Our pre-registration specified regularisation to reduce overfitting and improve model stability, following recommendations by Gelman et al. (2020). We originally planned to use Bayesian LASSO regression, with the penalty strength estimated from the data. Because brms does not support LASSO priors, we instead implemented a regularised horseshoe prior, a sparsity-inducing alternative that shrinks small coefficients towards zero while allowing larger coefficients to remain comparatively less shrunk. This analysis was used as a robustness check on the full model: if substantive predictors retained non-negligible coefficients under the horseshoe prior, this was taken as evidence that their associations were not solely attributable to overfitting. We specified horseshoe(df = 3, scale_global = 0.5) on all fixed-effect coefficients, where df = 3 yields moderately heavy tails for the local scales and scale_global = 0.5 reflects a prior expectation that most predictors have effects close to zero.

Table 27: Posterior means and 95% credible intervals (log-odds scale) from Bayesian multilevel logistic regression models with regularised horseshoe priors predicting disclosure of technology-facilitated CSEA to any channel. Two model specifications are compared: random intercepts only (Intercepts) and random intercepts and slopes by country (Slopes). Horseshoe prior: df = 3, scale_global = 0.5. Models fit via brm_multiple across M = 30 imputed datasets with adapt_delta = 0.99.

predictor	Horseshoe Random Intercept	Horseshoe Random Slopes
Girls (vs Boys)	0.10 [-0.05, 0.31]	0.05 [-0.13, 0.28]
Age (per year)	-0.12 [-0.18, -0.05]	-0.10 [-0.20, 0.00]
Premarital sex	0.24 [-0.01, 0.52]	0.16 [-0.07, 0.51]
Inequitable gender attitudes	0.17 [0.03, 0.29]	0.10 [-0.07, 0.32]
Enabling parental mediation	0.21 [0.09, 0.33]	0.18 [0.00, 0.34]
Sex education	-0.06 [-0.28, 0.09]	-0.05 [-0.30, 0.12]
Help-seeking	0.28 [0.05, 0.50]	0.25 [-0.01, 0.54]
Digital skills to report	-0.01 [-0.09, 0.07]	-0.01 [-0.11, 0.08]

5.7 Posterior Probability of Direction

This table presents the posterior probability of direction (PD) for each predictor across models of disclosure of technology-facilitated CSEA: (A) the random-intercepts model and (B) the regularised horseshoe regression. The probability of direction indicates the certainty that an estimated coefficient is positive or negative, expressed as a percentage. For example, PD = 97% means there is a 97% posterior probability that the coefficient is above or below zero in the estimated direction. Values closer to 100% indicate greater directional certainty, whereas values near 50% indicate that the data are uninformative about the sign of the association.

Table 28: Posterior probabilities of direction (PD) for each predictor across the random intercepts and slopes disclosure models (All, Formal, Informal channels). Columns report posterior mean (log-odds), PD, and estimated direction. Estimates pooled across M = 30 imputed datasets.

Model	Predictor	Mean	PD	Direction
All	Girls (vs Boys)	0.13	81.9%	positive
All	Age (per year)	-0.12	99.4%	negative
All	Premarital sex	0.29	96.2%	positive
All	Inequitable gender attitudes	0.23	96.9%	positive
All	Enabling parental mediation	0.22	99.4%	positive
All	Sex education	-0.15	83.9%	negative
All	Help-seeking	0.38	99.5%	positive
All	Digital skills to report	-0.04	70.5%	negative
Formal	Girls (vs Boys)	-0.17	72.4%	negative
Formal	Age (per year)	-0.27	99.9%	negative
Formal	Premarital sex	-0.10	65.4%	negative
Formal	Inequitable gender attitudes	0.27	96.8%	positive
Formal	Enabling parental mediation	0.32	99.7%	positive
Formal	Sex education	0.06	62.7%	positive
Formal	Help-seeking	0.03	57.4%	positive
Formal	Digital skills to report	0.02	57.4%	positive
Informal	Girls (vs Boys)	0.12	80.6%	positive
Informal	Age (per year)	-0.11	96.6%	negative
Informal	Premarital sex	0.33	98.0%	positive
Informal	Inequitable gender attitudes	0.20	95.6%	positive
Informal	Enabling parental mediation	0.19	99.0%	positive
Informal	Sex education	-0.08	68.9%	negative
Informal	Help-seeking	0.40	99.8%	positive
Informal	Digital skills to report	-0.08	86.3%	negative

Table 29: Posterior probabilities of direction (PD) for each predictor across the regularised horseshoe regression models (random intercepts only vs random intercepts and slopes). Columns report posterior mean (log-odds), PD, and estimated direction. Estimates pooled across M = 30 imputed datasets.

Model	Predictor	Mean	PD	Direction
Intercepts	Girls (vs Boys)	0.10	86.9%	positive
Intercepts	Age (per year)	-0.12	100.0%	negative
Intercepts	Premarital sex	0.24	96.2%	positive
Intercepts	Inequitable gender attitudes	0.17	99.4%	positive
Intercepts	Enabling parental mediation	0.21	100.0%	positive
Intercepts	Sex education	-0.06	75.1%	negative
Intercepts	Help-seeking	0.28	99.4%	positive
Intercepts	Digital skills to report	-0.01	58.8%	negative
Slopes	Girls (vs Boys)	0.05	69.5%	positive
Slopes	Age (per year)	-0.10	97.7%	negative
Slopes	Premarital sex	0.16	86.2%	positive
Slopes	Inequitable gender attitudes	0.10	82.9%	positive
Slopes	Enabling parental mediation	0.18	97.6%	positive
Slopes	Sex education	-0.05	69.2%	negative
Slopes	Help-seeking	0.25	95.4%	positive
Slopes	Digital skills to report	-0.01	59.5%	negative

5.8 Country-specific Coefficients Predicting Disclosure

Country-specific posterior coefficients (log-odds scale) from Bayesian multilevel logistic regression models predicting disclosure of technology-facilitated CSEA to any channel (All), formal channels only (Formal), and informal channels only (Informal). For each country and parameter, the table reports the posterior mean (Estimate), 95% credible interval [Q2.5, Q97.5], Posterior probability of direction (PD), and a binary flag indicating whether the 95% credible interval excludes zero. Models were fit using multiple imputation (M = 30 datasets) via brm_multiple with random intercepts and random slopes for country. Intercept terms are excluded.

Table 30: Country-specific posterior coefficients (log-odds) for disclosure models (All, Formal, Informal).

Country	Model	Parameter	Estimate	Q2.5	Q97.5	PD	Excludes 0?
Cambodia	All	Age (per year)	-0.15	-0.33	-0.01	97.2%	Yes
Cambodia	All	Digital skills to report	-0.08	-0.36	0.13	74.6%	No
Cambodia	All	Enabling parental mediation	0.13	-0.27	0.37	79.2%	No
Cambodia	All	Girls (vs Boys)	-0.23	-1.06	0.31	73.4%	No
Cambodia	All	Help-seeking	0.47	0.03	1.09	96.4%	Yes
Cambodia	All	Inequitable gender attitudes	0.43	0.05	0.89	97.7%	Yes
Cambodia	All	Premarital sex	0.36	-0.14	1.03	90.0%	No
Cambodia	All	Sex education	-0.03	-0.53	0.56	55.1%	No
Cambodia	Formal	Age (per year)	-0.21	-0.38	0.00	98.8%	Yes
Cambodia	Formal	Digital skills to report	-0.06	-0.37	0.16	67.6%	No
Cambodia	Formal	Enabling parental mediation	0.20	-0.22	0.48	86.3%	No
Cambodia	Formal	Girls (vs Boys)	-0.27	-1.03	0.45	76.6%	No
Cambodia	Formal	Help-seeking	-0.06	-0.82	0.52	57.8%	No
Cambodia	Formal	Inequitable gender attitudes	0.26	-0.10	0.58	94.4%	No
Cambodia	Formal	Premarital sex	-0.10	-0.77	0.54	62.1%	No
Cambodia	Formal	Sex education	0.12	-0.36	0.67	67.9%	No
Cambodia	Informal	Age (per year)	-0.17	-0.37	0.01	96.2%	No
Cambodia	Informal	Digital skills to report	-0.18	-0.51	0.05	89.4%	No
Cambodia	Informal	Enabling parental mediation	0.10	-0.28	0.34	74.3%	No
Cambodia	Informal	Girls (vs Boys)	-0.15	-0.87	0.32	68.5%	No
Cambodia	Informal	Help-seeking	0.43	0.02	0.93	97.6%	Yes
Cambodia	Informal	Inequitable gender attitudes	0.38	0.02	0.78	97.3%	Yes
Cambodia	Informal	Premarital sex	0.44	-0.01	1.10	94.6%	No
Cambodia	Informal	Sex education	0.12	-0.40	0.79	65.9%	No
Ethiopia	All	Age (per year)	-0.10	-0.26	0.07	88.7%	No
Ethiopia	All	Digital skills to report	0.07	-0.12	0.33	71.8%	No
Ethiopia	All	Enabling parental mediation	0.23	0.01	0.48	97.9%	Yes
Ethiopia	All	Girls (vs Boys)	0.10	-0.42	0.56	66.0%	No
Ethiopia	All	Help-seeking	0.36	-0.07	0.81	95.4%	No
Ethiopia	All	Inequitable gender attitudes	0.17	-0.20	0.50	82.6%	No
Ethiopia	All	Premarital sex	0.37	-0.10	1.01	91.4%	No
Ethiopia	All	Sex education	0.09	-0.35	0.72	62.8%	No
Ethiopia	Formal	Age (per year)	-0.22	-0.42	0.01	98.4%	No

Ethiopia	Formal	Digital skills to report	-0.04	-0.30	0.17	63.2%	No
Ethiopia	Formal	Enabling parental mediation	0.27	-0.05	0.53	96.7%	No
Ethiopia	Formal	Girls (vs Boys)	-0.37	-1.16	0.38	82.9%	No
Ethiopia	Formal	Help-seeking	0.14	-0.40	0.78	68.0%	No
Ethiopia	Formal	Inequitable gender attitudes	0.31	-0.03	0.70	96.2%	No
Ethiopia	Formal	Premarital sex	0.01	-0.62	0.76	50.8%	No
Ethiopia	Formal	Sex education	0.08	-0.41	0.58	62.7%	No
Ethiopia	Informal	Age (per year)	-0.08	-0.29	0.12	78.5%	No
Ethiopia	Informal	Digital skills to report	0.06	-0.14	0.33	69.5%	No
Ethiopia	Informal	Enabling parental mediation	0.23	0.01	0.48	97.4%	Yes
Ethiopia	Informal	Girls (vs Boys)	0.11	-0.37	0.55	68.4%	No
Ethiopia	Informal	Help-seeking	0.34	-0.08	0.71	96.1%	No
Ethiopia	Informal	Inequitable gender attitudes	0.09	-0.26	0.40	70.6%	No
Ethiopia	Informal	Premarital sex	0.33	-0.17	0.81	91.6%	No
Ethiopia	Informal	Sex education	0.17	-0.31	0.79	72.1%	No
Indonesia	All	Age (per year)	-0.15	-0.34	0.01	95.5%	No
Indonesia	All	Digital skills to report	-0.10	-0.42	0.11	77.6%	No
Indonesia	All	Enabling parental mediation	0.14	-0.24	0.37	82.4%	No
Indonesia	All	Girls (vs Boys)	0.15	-0.43	0.74	70.9%	No
Indonesia	All	Help-seeking	0.37	-0.13	0.88	93.3%	No
Indonesia	All	Inequitable gender attitudes	0.16	-0.28	0.61	76.7%	No
Indonesia	All	Premarital sex	0.29	-0.26	0.86	85.9%	No
Indonesia	All	Sex education	-0.22	-0.87	0.33	76.9%	No
Indonesia	Formal	Age (per year)	-0.29	-0.66	0.04	95.9%	No
Indonesia	Formal	Digital skills to report	0.03	-0.36	0.48	55.2%	No
Indonesia	Formal	Enabling parental mediation	0.30	-0.25	0.81	88.8%	No
Indonesia	Formal	Girls (vs Boys)	-0.21	-1.91	1.47	59.9%	No
Indonesia	Formal	Help-seeking	-0.02	-1.15	0.99	51.5%	No
Indonesia	Formal	Inequitable gender attitudes	0.15	-0.69	0.64	67.8%	No
Indonesia	Formal	Premarital sex	0.02	-0.98	1.25	51.3%	No
Indonesia	Formal	Sex education	0.06	-0.77	0.89	56.5%	No
Indonesia	Informal	Age (per year)	-0.15	-0.38	0.06	91.0%	No
Indonesia	Informal	Digital skills to report	-0.15	-0.48	0.09	85.5%	No
Indonesia	Informal	Enabling parental mediation	0.12	-0.26	0.35	77.8%	No
Indonesia	Informal	Girls (vs Boys)	0.13	-0.42	0.69	69.1%	No
Indonesia	Informal	Help-seeking	0.40	-0.05	0.86	96.5%	No
Indonesia	Informal	Inequitable gender attitudes	0.17	-0.26	0.60	78.4%	No
Indonesia	Informal	Premarital sex	0.34	-0.19	0.86	90.6%	No
Indonesia	Informal	Sex education	-0.16	-0.90	0.47	68.9%	No
Kenya	All	Age (per year)	-0.13	-0.27	0.00	97.3%	Yes
Kenya	All	Digital skills to report	-0.06	-0.25	0.10	75.4%	No
Kenya	All	Enabling parental mediation	0.23	0.01	0.46	98.1%	Yes
Kenya	All	Girls (vs Boys)	0.16	-0.28	0.60	76.8%	No

Kenya	All	Help-seeking	0.49	0.13	1.02	98.6%	Yes
Kenya	All	Inequitable gender attitudes	0.36	0.07	0.69	98.8%	Yes
Kenya	All	Premarital sex	0.24	-0.24	0.67	86.3%	No
Kenya	All	Sex education	-0.08	-0.50	0.38	64.6%	No
Kenya	Formal	Age (per year)	-0.33	-0.54	-0.16	100.0%	Yes
Kenya	Formal	Digital skills to report	-0.02	-0.24	0.18	57.4%	No
Kenya	Formal	Enabling parental mediation	0.31	0.00	0.60	98.1%	Yes
Kenya	Formal	Girls (vs Boys)	-0.34	-1.03	0.34	83.2%	No
Kenya	Formal	Help-seeking	0.22	-0.27	0.90	77.3%	No
Kenya	Formal	Inequitable gender attitudes	0.32	0.02	0.64	98.3%	Yes
Kenya	Formal	Premarital sex	-0.14	-0.80	0.45	67.2%	No
Kenya	Formal	Sex education	0.20	-0.26	0.84	76.6%	No
Kenya	Informal	Age (per year)	-0.08	-0.24	0.07	85.3%	No
Kenya	Informal	Digital skills to report	-0.08	-0.26	0.10	80.6%	No
Kenya	Informal	Enabling parental mediation	0.17	-0.07	0.38	93.7%	No
Kenya	Informal	Girls (vs Boys)	0.14	-0.27	0.55	75.6%	No
Kenya	Informal	Help-seeking	0.48	0.15	0.94	99.2%	Yes
Kenya	Informal	Inequitable gender attitudes	0.33	0.06	0.64	98.6%	Yes
Kenya	Informal	Premarital sex	0.33	-0.09	0.75	94.2%	No
Kenya	Informal	Sex education	0.03	-0.40	0.54	55.8%	No
Malaysia	All	Age (per year)	-0.13	-0.30	0.04	93.8%	No
Malaysia	All	Digital skills to report	0.04	-0.17	0.38	62.9%	No
Malaysia	All	Enabling parental mediation	0.25	-0.01	0.57	96.4%	No
Malaysia	All	Girls (vs Boys)	0.03	-0.62	0.53	54.2%	No
Malaysia	All	Help-seeking	0.49	0.06	1.13	96.7%	Yes
Malaysia	All	Inequitable gender attitudes	-0.11	-0.67	0.32	66.3%	No
Malaysia	All	Premarital sex	0.28	-0.34	0.86	83.2%	No
Malaysia	All	Sex education	-0.42	-1.24	0.11	88.1%	No
Malaysia	Formal	Age (per year)	-0.38	-0.78	-0.16	99.1%	Yes
Malaysia	Formal	Digital skills to report	0.06	-0.24	0.48	62.6%	No
Malaysia	Formal	Enabling parental mediation	0.32	-0.11	0.73	94.2%	No
Malaysia	Formal	Girls (vs Boys)	-0.16	-1.27	0.95	61.2%	No
Malaysia	Formal	Help-seeking	0.13	-0.60	1.05	63.2%	No
Malaysia	Formal	Inequitable gender attitudes	0.17	-0.45	0.56	75.0%	No
Malaysia	Formal	Premarital sex	-0.15	-1.20	0.67	63.3%	No
Malaysia	Formal	Sex education	-0.06	-0.94	0.51	57.1%	No
Malaysia	Informal	Age (per year)	-0.11	-0.32	0.10	85.3%	No
Malaysia	Informal	Digital skills to report	0.04	-0.21	0.40	59.9%	No
Malaysia	Informal	Enabling parental mediation	0.24	-0.02	0.58	95.1%	No
Malaysia	Informal	Girls (vs Boys)	0.05	-0.56	0.53	57.1%	No
Malaysia	Informal	Help-seeking	0.48	0.09	1.07	97.8%	Yes
Malaysia	Informal	Inequitable gender attitudes	-0.12	-0.67	0.29	68.7%	No
Malaysia	Informal	Premarital sex	0.32	-0.26	0.86	88.1%	No

Malaysia	Informal	Sex education	-0.47	-1.38	0.15	87.6%	No
Mozambique	All	Age (per year)	-0.06	-0.18	0.09	81.1%	No
Mozambique	All	Digital skills to report	0.00	-0.17	0.20	50.0%	No
Mozambique	All	Enabling parental mediation	0.22	0.01	0.41	98.6%	Yes
Mozambique	All	Girls (vs Boys)	0.25	-0.13	0.68	88.8%	No
Mozambique	All	Help-seeking	0.27	-0.17	0.61	91.4%	No
Mozambique	All	Inequitable gender attitudes	0.10	-0.19	0.38	76.2%	No
Mozambique	All	Premarital sex	0.30	-0.09	0.69	93.5%	No
Mozambique	All	Sex education	-0.17	-0.59	0.23	79.8%	No
Mozambique	Formal	Age (per year)	-0.36	-0.62	-0.17	99.9%	Yes
Mozambique	Formal	Digital skills to report	-0.04	-0.35	0.19	63.0%	No
Mozambique	Formal	Enabling parental mediation	0.34	0.05	0.65	98.9%	Yes
Mozambique	Formal	Girls (vs Boys)	-0.51	-1.39	0.30	88.1%	No
Mozambique	Formal	Help-seeking	-0.10	-0.89	0.49	61.5%	No
Mozambique	Formal	Inequitable gender attitudes	0.39	0.06	0.82	98.0%	Yes
Mozambique	Formal	Premarital sex	0.16	-0.47	1.02	66.0%	No
Mozambique	Formal	Sex education	-0.01	-0.66	0.50	51.5%	No
Mozambique	Informal	Age (per year)	-0.01	-0.15	0.14	56.8%	No
Mozambique	Informal	Digital skills to report	-0.01	-0.19	0.22	53.2%	No
Mozambique	Informal	Enabling parental mediation	0.20	0.00	0.41	98.0%	Yes
Mozambique	Informal	Girls (vs Boys)	0.22	-0.14	0.64	87.3%	No
Mozambique	Informal	Help-seeking	0.30	-0.12	0.62	95.1%	No
Mozambique	Informal	Inequitable gender attitudes	0.09	-0.19	0.36	74.0%	No
Mozambique	Informal	Premarital sex	0.32	-0.07	0.69	95.3%	No
Mozambique	Informal	Sex education	-0.16	-0.63	0.26	76.6%	No
Namibia	All	Age (per year)	-0.08	-0.21	0.09	85.0%	No
Namibia	All	Digital skills to report	-0.10	-0.34	0.08	81.7%	No
Namibia	All	Enabling parental mediation	0.26	0.08	0.48	99.4%	Yes
Namibia	All	Girls (vs Boys)	0.20	-0.24	0.66	81.4%	No
Namibia	All	Help-seeking	0.43	0.06	0.90	98.1%	Yes
Namibia	All	Inequitable gender attitudes	0.31	0.00	0.66	96.8%	Yes
Namibia	All	Premarital sex	0.15	-0.43	0.56	73.1%	No
Namibia	All	Sex education	-0.27	-0.85	0.17	85.2%	No
Namibia	Formal	Age (per year)	-0.19	-0.36	0.05	96.5%	No
Namibia	Formal	Digital skills to report	0.07	-0.15	0.38	70.1%	No
Namibia	Formal	Enabling parental mediation	0.45	0.17	0.85	99.5%	Yes
Namibia	Formal	Girls (vs Boys)	-0.26	-1.00	0.47	75.4%	No
Namibia	Formal	Help-seeking	0.09	-0.47	0.71	62.6%	No
Namibia	Formal	Inequitable gender attitudes	0.33	-0.02	0.71	96.7%	No
Namibia	Formal	Premarital sex	-0.27	-1.17	0.35	76.3%	No
Namibia	Formal	Sex education	0.05	-0.51	0.57	56.9%	No
Namibia	Informal	Age (per year)	0.01	-0.16	0.20	53.1%	No
Namibia	Informal	Digital skills to report	-0.16	-0.41	0.04	91.0%	No

Namibia	Informal	Enabling parental mediation	0.23	0.04	0.44	98.7%	Yes
Namibia	Informal	Girls (vs Boys)	0.22	-0.19	0.69	84.1%	No
Namibia	Informal	Help-seeking	0.46	0.12	0.91	99.0%	Yes
Namibia	Informal	Inequitable gender attitudes	0.28	-0.03	0.64	95.1%	No
Namibia	Informal	Premarital sex	0.22	-0.35	0.61	81.5%	No
Namibia	Informal	Sex education	-0.30	-0.96	0.19	84.6%	No
Philippines	All	Age (per year)	-0.13	-0.26	-0.02	98.6%	Yes
Philippines	All	Digital skills to report	-0.01	-0.18	0.17	56.5%	No
Philippines	All	Enabling parental mediation	0.21	-0.03	0.43	97.1%	No
Philippines	All	Girls (vs Boys)	0.37	-0.01	0.89	94.3%	No
Philippines	All	Help-seeking	0.26	-0.20	0.62	90.0%	No
Philippines	All	Inequitable gender attitudes	0.12	-0.16	0.42	80.1%	No
Philippines	All	Premarital sex	0.38	-0.04	0.95	93.9%	No
Philippines	All	Sex education	-0.22	-0.75	0.24	81.2%	No
Philippines	Formal	Age (per year)	-0.25	-0.44	-0.06	99.6%	Yes
Philippines	Formal	Digital skills to report	0.03	-0.22	0.32	57.7%	No
Philippines	Formal	Enabling parental mediation	0.38	0.05	0.81	97.9%	Yes
Philippines	Formal	Girls (vs Boys)	-1.01	-1.97	-0.14	98.5%	Yes
Philippines	Formal	Help-seeking	-0.28	-1.29	0.35	74.6%	No
Philippines	Formal	Inequitable gender attitudes	0.18	-0.31	0.51	80.3%	No
Philippines	Formal	Premarital sex	-0.03	-0.83	0.83	53.0%	No
Philippines	Formal	Sex education	0.10	-0.52	0.75	62.0%	No
Philippines	Informal	Age (per year)	-0.12	-0.26	0.01	95.3%	No
Philippines	Informal	Digital skills to report	-0.04	-0.22	0.15	66.5%	No
Philippines	Informal	Enabling parental mediation	0.19	-0.05	0.41	95.6%	No
Philippines	Informal	Girls (vs Boys)	0.37	-0.02	0.91	93.7%	No
Philippines	Informal	Help-seeking	0.34	-0.06	0.67	97.0%	No
Philippines	Informal	Inequitable gender attitudes	0.10	-0.17	0.37	76.4%	No
Philippines	Informal	Premarital sex	0.38	-0.03	0.87	95.6%	No
Philippines	Informal	Sex education	-0.18	-0.76	0.32	75.6%	No
Tanzania	All	Age (per year)	-0.09	-0.26	0.09	86.6%	No
Tanzania	All	Digital skills to report	0.00	-0.22	0.27	50.6%	No
Tanzania	All	Enabling parental mediation	0.24	-0.01	0.53	96.9%	No
Tanzania	All	Girls (vs Boys)	0.22	-0.33	0.86	77.7%	No
Tanzania	All	Help-seeking	0.43	-0.02	1.01	95.6%	No
Tanzania	All	Inequitable gender attitudes	0.25	-0.16	0.68	88.3%	No
Tanzania	All	Premarital sex	0.23	-0.42	0.73	79.2%	No
Tanzania	All	Sex education	-0.11	-0.65	0.48	65.2%	No
Tanzania	Formal	Age (per year)	-0.23	-0.46	0.04	97.1%	No
Tanzania	Formal	Digital skills to report	-0.02	-0.34	0.26	54.7%	No
Tanzania	Formal	Enabling parental mediation	0.33	-0.01	0.71	97.2%	No
Tanzania	Formal	Girls (vs Boys)	-0.54	-1.77	0.52	82.3%	No
Tanzania	Formal	Help-seeking	0.28	-0.35	1.33	74.9%	No

Tanzania	Formal	Inequitable gender attitudes	0.17	-0.39	0.52	77.0%	No
Tanzania	Formal	Premarital sex	-0.20	-1.19	0.53	68.5%	No
Tanzania	Formal	Sex education	0.04	-0.63	0.64	55.1%	No
Tanzania	Informal	Age (per year)	-0.07	-0.29	0.15	73.6%	No
Tanzania	Informal	Digital skills to report	-0.04	-0.30	0.24	63.1%	No
Tanzania	Informal	Enabling parental mediation	0.20	-0.06	0.47	94.4%	No
Tanzania	Informal	Girls (vs Boys)	0.23	-0.26	0.86	79.6%	No
Tanzania	Informal	Help-seeking	0.41	0.00	0.90	97.1%	No
Tanzania	Informal	Inequitable gender attitudes	0.26	-0.11	0.68	90.5%	No
Tanzania	Informal	Premarital sex	0.27	-0.34	0.74	84.9%	No
Tanzania	Informal	Sex education	0.00	-0.59	0.69	50.6%	No
Thailand	All	Age (per year)	-0.20	-0.43	-0.05	98.2%	Yes
Thailand	All	Digital skills to report	-0.07	-0.41	0.20	69.3%	No
Thailand	All	Enabling parental mediation	0.16	-0.17	0.39	86.9%	No
Thailand	All	Girls (vs Boys)	0.06	-0.51	0.55	59.4%	No
Thailand	All	Help-seeking	0.22	-0.41	0.67	79.6%	No
Thailand	All	Inequitable gender attitudes	0.58	0.20	1.06	99.5%	Yes
Thailand	All	Premarital sex	0.33	-0.17	0.89	90.0%	No
Thailand	All	Sex education	0.04	-0.50	0.81	54.5%	No
Thailand	Formal	Age (per year)	-0.22	-0.42	0.03	97.6%	No
Thailand	Formal	Digital skills to report	0.10	-0.19	0.61	69.1%	No
Thailand	Formal	Enabling parental mediation	0.39	0.07	0.85	97.9%	Yes
Thailand	Formal	Girls (vs Boys)	0.66	-0.23	1.66	91.5%	No
Thailand	Formal	Help-seeking	0.09	-0.62	0.88	59.4%	No
Thailand	Formal	Inequitable gender attitudes	0.41	0.10	0.82	98.8%	Yes
Thailand	Formal	Premarital sex	-0.05	-0.82	0.82	54.7%	No
Thailand	Formal	Sex education	0.08	-0.55	0.77	60.0%	No
Thailand	Informal	Age (per year)	-0.29	-0.54	-0.07	99.1%	Yes
Thailand	Informal	Digital skills to report	-0.16	-0.57	0.15	82.0%	No
Thailand	Informal	Enabling parental mediation	0.10	-0.28	0.35	72.6%	No
Thailand	Informal	Girls (vs Boys)	0.00	-0.60	0.47	50.3%	No
Thailand	Informal	Help-seeking	0.33	-0.20	0.76	92.1%	No
Thailand	Informal	Inequitable gender attitudes	0.58	0.18	1.06	99.4%	Yes
Thailand	Informal	Premarital sex	0.41	-0.07	0.99	93.9%	No
Thailand	Informal	Sex education	0.34	-0.32	1.33	78.3%	No
Uganda	All	Age (per year)	-0.06	-0.20	0.11	80.0%	No
Uganda	All	Digital skills to report	-0.03	-0.20	0.14	64.2%	No
Uganda	All	Enabling parental mediation	0.25	0.06	0.49	99.2%	Yes
Uganda	All	Girls (vs Boys)	0.25	-0.14	0.70	88.3%	No
Uganda	All	Help-seeking	0.28	-0.14	0.63	92.9%	No
Uganda	All	Inequitable gender attitudes	0.36	0.09	0.66	99.3%	Yes
Uganda	All	Premarital sex	0.34	-0.05	0.82	94.1%	No
Uganda	All	Sex education	-0.15	-0.61	0.26	75.9%	No

Uganda	Formal	Age (per year)	-0.25	-0.42	-0.06	99.7%	Yes
Uganda	Formal	Digital skills to report	0.04	-0.16	0.27	65.3%	No
Uganda	Formal	Enabling parental mediation	0.25	-0.04	0.49	96.9%	No
Uganda	Formal	Girls (vs Boys)	0.89	0.23	1.57	99.5%	Yes
Uganda	Formal	Help-seeking	0.01	-0.51	0.49	51.7%	No
Uganda	Formal	Inequitable gender attitudes	0.35	0.05	0.66	98.9%	Yes
Uganda	Formal	Premarital sex	-0.18	-0.79	0.37	72.7%	No
Uganda	Formal	Sex education	-0.01	-0.55	0.45	51.0%	No
Uganda	Informal	Age (per year)	0.05	-0.12	0.25	71.5%	No
Uganda	Informal	Digital skills to report	-0.09	-0.27	0.09	82.8%	No
Uganda	Informal	Enabling parental mediation	0.25	0.07	0.50	99.0%	Yes
Uganda	Informal	Girls (vs Boys)	0.10	-0.31	0.47	69.6%	No
Uganda	Informal	Help-seeking	0.33	-0.06	0.65	97.1%	No
Uganda	Informal	Inequitable gender attitudes	0.25	0.01	0.51	97.7%	Yes
Uganda	Informal	Premarital sex	0.40	0.03	0.86	97.1%	Yes
Uganda	Informal	Sex education	-0.17	-0.67	0.27	76.2%	No
Vietnam	All	Age (per year)	-0.20	-0.48	-0.02	95.5%	Yes
Vietnam	All	Digital skills to report	-0.07	-0.43	0.21	68.1%	No
Vietnam	All	Enabling parental mediation	0.32	0.04	0.85	94.3%	Yes
Vietnam	All	Girls (vs Boys)	-0.01	-0.79	0.57	51.1%	No
Vietnam	All	Help-seeking	0.45	-0.07	1.13	94.0%	No
Vietnam	All	Inequitable gender attitudes	0.05	-0.60	0.57	56.5%	No
Vietnam	All	Premarital sex	0.18	-0.59	0.70	71.5%	No
Vietnam	All	Sex education	-0.27	-1.09	0.37	77.9%	No
Vietnam	Formal	Age (per year)	-0.31	-0.67	-0.03	97.6%	Yes
Vietnam	Formal	Digital skills to report	0.09	-0.21	0.64	67.0%	No
Vietnam	Formal	Enabling parental mediation	0.36	-0.05	0.88	94.8%	No
Vietnam	Formal	Girls (vs Boys)	0.03	-1.32	1.52	51.6%	No
Vietnam	Formal	Help-seeking	-0.12	-1.29	0.67	59.9%	No
Vietnam	Formal	Inequitable gender attitudes	0.14	-0.69	0.57	66.7%	No
Vietnam	Formal	Premarital sex	-0.22	-1.39	0.59	67.8%	No
Vietnam	Formal	Sex education	0.05	-0.70	0.77	55.7%	No
Vietnam	Informal	Age (per year)	-0.25	-0.59	0.00	94.9%	No
Vietnam	Informal	Digital skills to report	-0.13	-0.53	0.20	76.7%	No
Vietnam	Informal	Enabling parental mediation	0.30	0.01	0.84	92.4%	Yes
Vietnam	Informal	Girls (vs Boys)	-0.01	-0.76	0.56	50.7%	No
Vietnam	Informal	Help-seeking	0.47	0.01	1.10	96.2%	Yes
Vietnam	Informal	Inequitable gender attitudes	0.02	-0.65	0.53	52.6%	No
Vietnam	Informal	Premarital sex	0.25	-0.47	0.77	80.0%	No
Vietnam	Informal	Sex education	-0.19	-1.10	0.63	67.4%	No

6 Model Checking

Because models were fit using multiple imputed datasets via brm_multiple, we took additional steps to ensure robust convergence diagnostics beyond default summaries. Standard diagnostics such as $\hat{R}$ can be misleading when computed by pooling chains across imputations, as chains are technically independent across imputations and may exhibit good within-imputation mixing but poor convergence when aggregated, particularly for parameters linked to variables with high missingness (e.g., inequitable gender attitudes).

We computed $\hat{R}$ separately for each set of chains corresponding to each imputed dataset and report the worst-case (maximum) value observed across all parameters and imputations. This conservative approach ensures that convergence difficulties are not masked by aggregation. A few parameters showed $\hat{R}$ values slightly exceeding the recommended threshold of 1.01; these likely reflect natural variability introduced by the imputation process rather than substantive convergence failures, and were evaluated further using trace plots and posterior predictive checks Vehtari et al. 2021). We supplemented numerical diagnostics with graphical checks for each model: (A) trace plots for fixed- and group-level parameters to assess mixing; (B) histograms of $\hat{R}$ values across all parameters to assess overall convergence distribution; and (C) posterior predictive checks stratified by sex to assess model fit to the observed data.

Table 31 reports, for each model, the worst-case $\hat{R}$ across parameters and imputations, total divergent transitions, minimum bulk and tail effective sample sizes (ESS), treedepth exceedances, and E-BFMI. Rows where divergences were present or $\hat{R}$ exceeded 1.05 are highlighted.

6.1 R-hat Diagnostics

Table 31: Imputation-aware convergence diagnostics by model. R-hat is computed within each imputation; the table reports the worst (maximum) across parameters and imputations, along with divergent transitions, minimum ESS (bulk/tail), treedepth hits, and E-BFMI.

Model	Worst $\hat{R}$	Min ESS (bulk)	Min ESS (tail)	Divergences	Divergences OK?	Treedepth hits
Disclosure: All	1.0116	926	591	0	Yes	0
Disclosure: Formal	1.0061	806	594	0	Yes	0
Disclosure: Informal	1.0131	754	473	0	Yes	0
Disclosure: Horseshoe (RI)	1.0082	726	1068	0	Yes	0
Disclosure: Horseshoe (RS)	1.0118	521	567	0	Yes	0

6.2 Graphical Diagnostics

We conducted comprehensive convergence diagnostics for all models. Below we present graphical diagnostics for the two main demographic models and the three main disclosure models (all channels, formal only, informal only). Additional diagnostics for other models are available in the source file `3b-disclosure-modelling-imp.qmd`

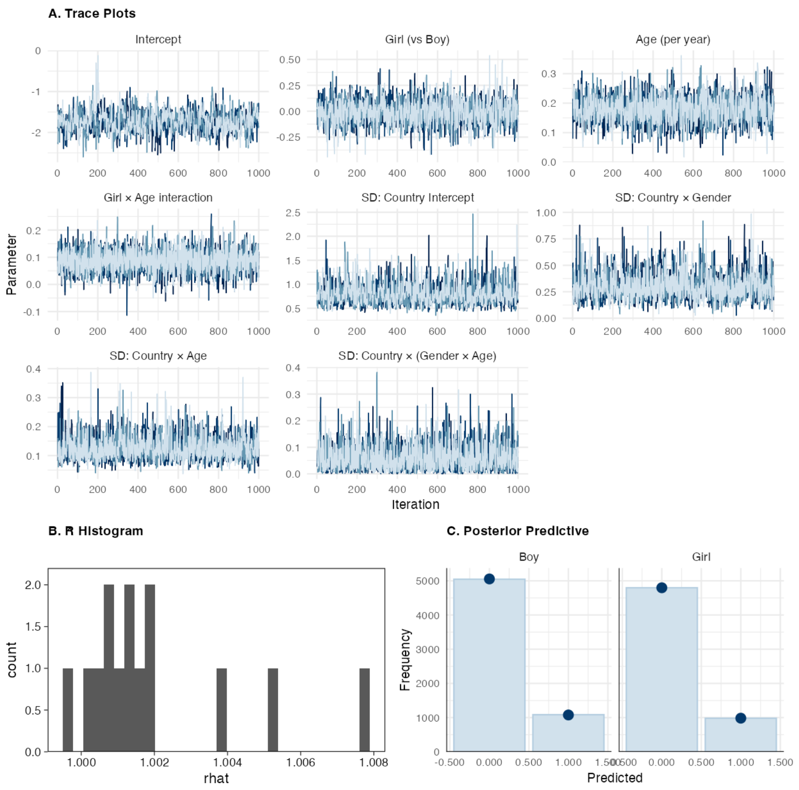

Figure 34: Graphical convergence diagnostics for the Bayesian multilevel logistic regression model predicting technology-facilitated CSEA exposure (age and gender model). Panel A shows trace plots for fixed effects (intercept, gender, age, age × gender interaction) and the country-level random intercept, assessing chain mixing across imputations. Panel B shows a histogram of worst-case $\hat{R}$ values across all parameters and imputed datasets. Panel C shows posterior predictive checks stratified by sex, comparing model-predicted to observed response distributions.

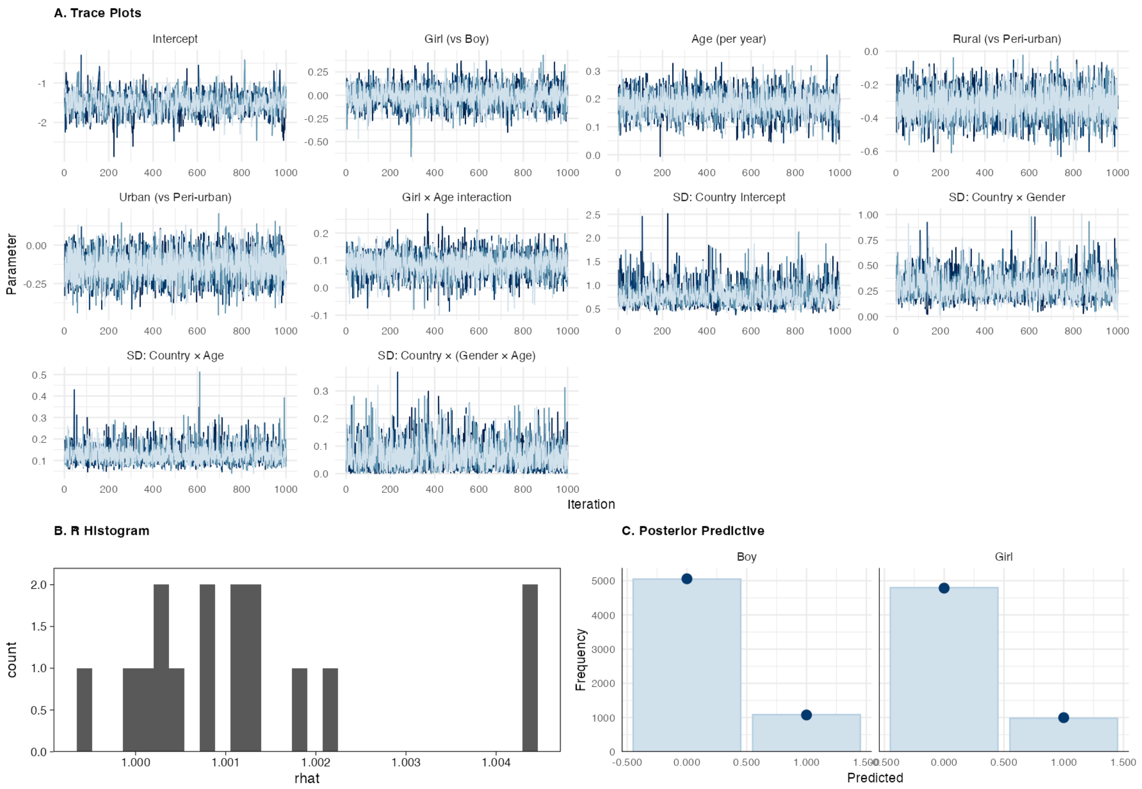

Figure 35: Graphical convergence diagnostics for the Bayesian multilevel logistic regression model predicting technology-facilitated CSEA exposure by degree of urbanisation. Panels as in the preceding figure: trace plots (A), worst-case $\hat{R}$ histogram (B), and posterior predictive check stratified by sex (C).

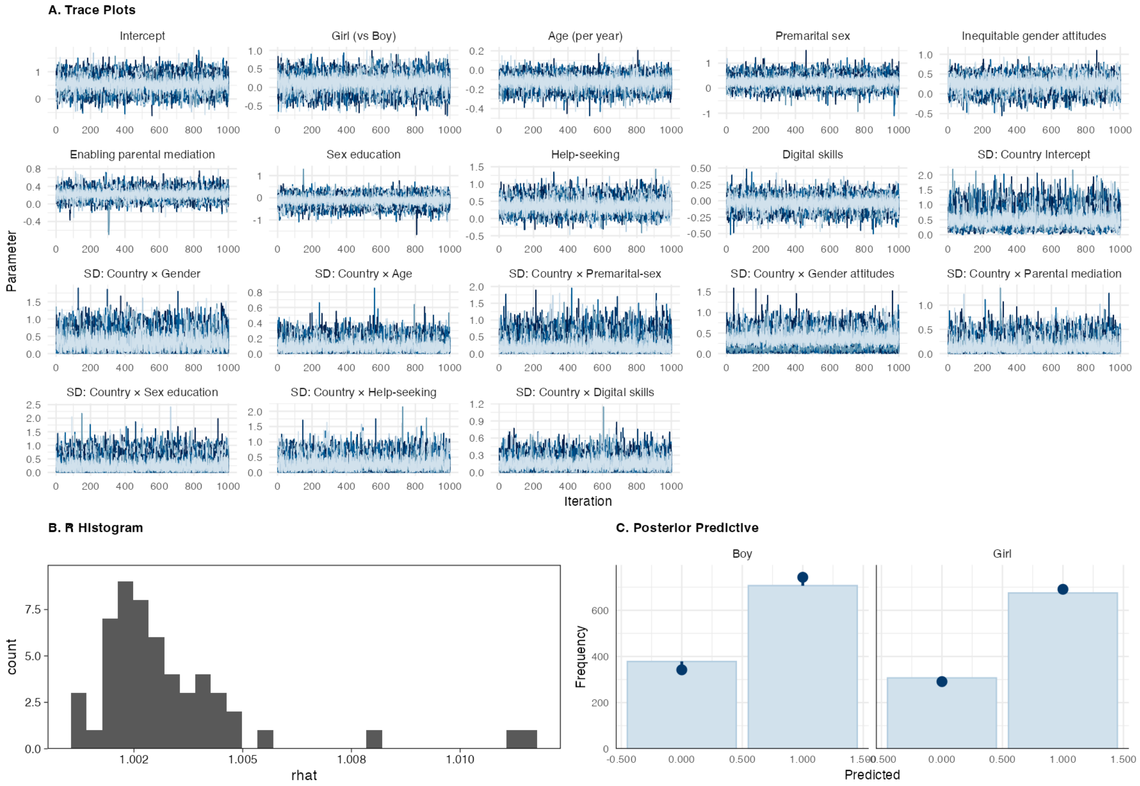

Figure 36: Graphical convergence diagnostics for the multiple-imputed Bayesian multilevel logistic regression model predicting disclosure to any channel. Panels as above: trace plots (A), worst-case $\hat{R}$ histogram across imputations (B), and posterior predictive check stratified by sex (C).

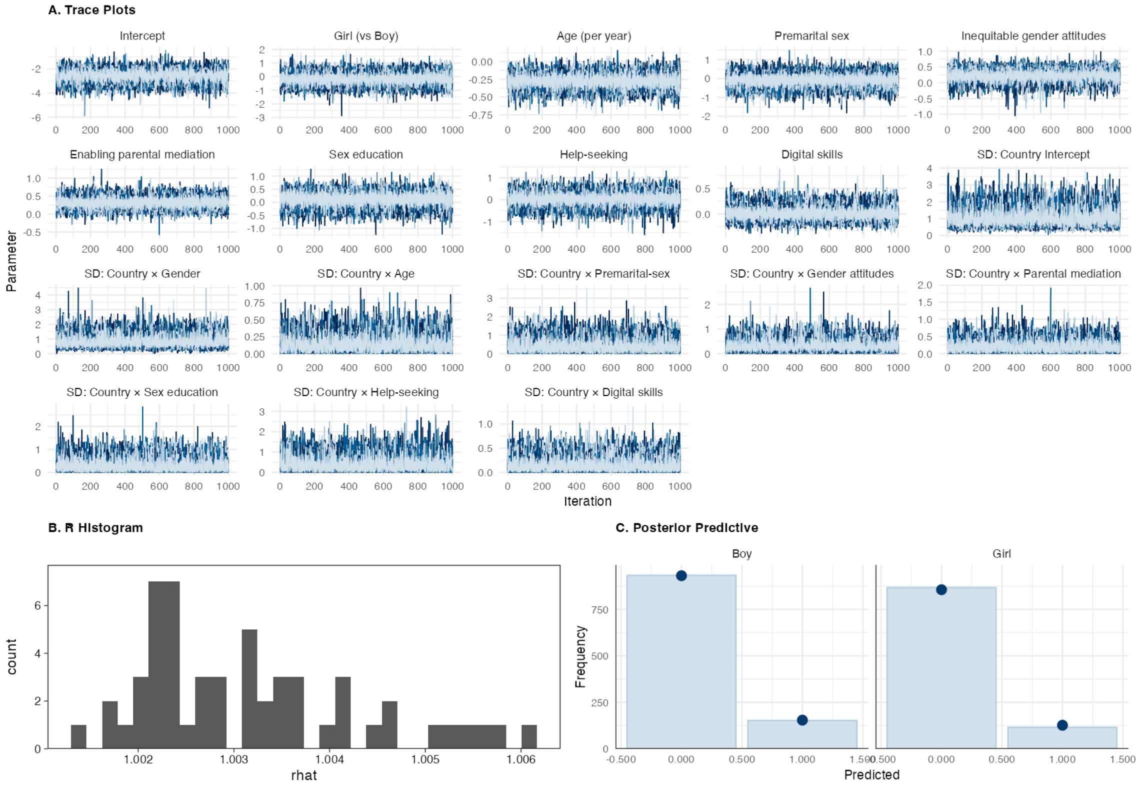

Figure 37: Graphical convergence diagnostics for the multiple-imputed Bayesian multilevel logistic regression model predicting formal disclosure (police, helpline, social worker, teacher). Panels as above: trace plots (A), worst-case $\hat{R}$ histogram across imputations (B), and posterior predictive check stratified by sex (C).

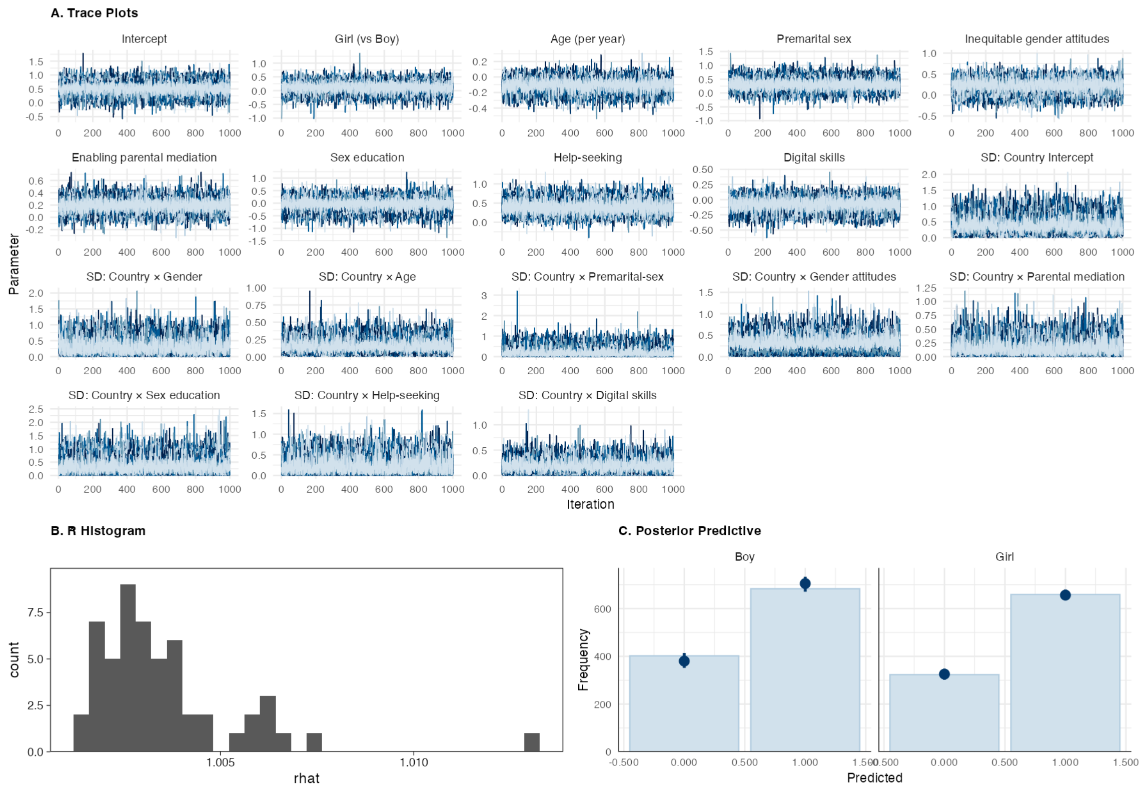

Figure 38: Graphical convergence diagnostics for the multiple-imputed Bayesian multilevel logistic regression model predicting informal disclosure (parents, siblings, friends, other trusted adults). Panels as above: trace plots (A), worst-case $\hat{R}$ histogram across imputations (B), and posterior predictive check stratified by sex (C).

7 Descriptives Summaries

This section presents supplementary descriptive tables supporting the main analyses reported in the paper. Tables 32–37 describe the study sample and measures. Table 32 summarises demographic characteristics of surveyed children across all 12 countries. Table 33 provides the coding scheme for all independent variables, and Table 34 presents the role-based classification of disclosure channels used as an analytic heuristic. Tables 35 and 36 report survey-weighted descriptive statistics for independent variables in the Africa and Asia subsamples respectively. Table 37 shows the full distribution of Likert-scale responses for nine forms of technology-facilitated CSEA, including 'Prefer not to say' and 'Don't know' categories.

Tables 38–41 address CSEA exposure and disclosure patterns. Tables 38 and 39 describe the demographic characteristics and prevalence of technology-facilitated CSEA by subgroup. Tables 40 and 41 examine disclosure patterns, reporting to whom children disclosed across countries and channels, and weighted disclosure rates by CSEA type.

Tables 42–44 characterise perpetrator identity. Table 42 presents full response descriptions and short labels for perpetrator categories; Table 43 reports weighted perpetrator type distributions disaggregated by CSEA form; and Table 44 presents weighted frequencies of perpetrator type combinations.

Tables 45–48 address barriers to disclosure and abuse context. Tables 45 and 46 provide full barrier descriptions and survey-weighted barrier prevalence by CSEA type, with reliability annotations. Table 47 reports weighted percentages of experiences occurring in online versus offline contexts, and Table 48 presents platform-specific estimates among children who experienced CSEA via social media.

Tables 49–54 present prevalence estimates. Table 49 reports overall prevalence of each CSEA type pooled across countries regardless of context, and Table 50 reports prevalence restricted to technology-facilitated incidents occurring via social media or online gaming platforms; both are presented with 95% confidence intervals. Table 51 reports country-level prevalence of any CSEA across online and offline contexts, and Table 52 reports country-level prevalence of technology-facilitated CSEA with complete-case denominators and weighted estimates of affected children. Table 53 reports the conditional distribution of incident location among children who experienced each harm type. Table 54 compares three operationalisations of technology-facilitated CSEA exposure: online-only, any context, and inferred online by country.

Tables 55 and 56 examine barrier co-occurrence among non-disclosed incidents. Table 55 shows that most incidents involved a single reported barrier, and Table 56 lists the most commonly co-occurring barrier pairs. Together these tables indicate that barriers to disclosure operated largely independently, reflecting heterogeneous rather than clustered pathways

Table 32: Demographic characteristics of internet-using children aged 12–17 who participated in the Disrupting Harm child survey across 12 countries in Africa and Asia (2020–2021). Characteristics reported include age, gender, and degree of urbanisation. Counts and proportions are survey-weighted to reflect the national population of internet-using children within each country.

**Characteristic**	**Overall** N = 11,912[1]	**Cambodia** N = 992[1]	**Ethiopia** N = 1,000[1]	**Indonesia** N = 995[1]	**Kenya** N = 1,014[1]	**Malaysia** N = 995[1]	**Mozambique** N = 999[1]	**Namibia** N = 994[1]	**Philippines** N = 950[1]	**Tanzania** N = 996[1]	**Thailand** N = 967[1]	**Uganda** N = 1,016[1]	**Vietnam** N = 994[1]
**Gender**													
Boy	6,044 (51%)	518 (52%)	639 (64%)	451 (45%)	480 (47%)	517 (52%)	522 (52%)	496 (50%)	421 (44%)	561 (56%)	411 (42%)	572 (56%)	455 (46%)
Girl	5,868 (49%)	474 (48%)	361 (36%)	544 (55%)	534 (53%)	478 (48%)	477 (48%)	498 (50%)	529 (56%)	435 (44%)	556 (58%)	444 (44%)	539 (54%)
**Age**													
12	1,412 (12%)	151 (15%)	43 (4.3%)	139 (14%)	129 (13%)	152 (15%)	87 (8.7%)	117 (12%)	155 (16%)	91 (9.2%)	138 (14%)	60 (5.9%)	150 (15%)
13	1,700 (14%)	160 (16%)	81 (8.1%)	156 (16%)	165 (16%)	155 (16%)	120 (12%)	147 (15%)	154 (16%)	140 (14%)	158 (16%)	98 (9.7%)	164 (17%)
14	1,896 (16%)	166 (17%)	125 (13%)	169 (17%)	156 (15%)	164 (17%)	143 (14%)	173 (17%)	158 (17%)	169 (17%)	162 (17%)	139 (14%)	170 (17%)
15	2,058 (17%)	173 (17%)	141 (14%)	173 (17%)	162 (16%)	172 (17%)	174 (17%)	187 (19%)	162 (17%)	185 (19%)	171 (18%)	186 (18%)	173 (17%)
16	2,311 (19%)	164 (17%)	241 (24%)	177 (18%)	192 (19%)	178 (18%)	239 (24%)	183 (18%)	160 (17%)	200 (20%)	173 (18%)	233 (23%)	171 (17%)
17	2,535 (21%)	178 (18%)	369 (37%)	180 (18%)	210 (21%)	174 (18%)	236 (24%)	188 (19%)	160 (17%)	210 (21%)	164 (17%)	299 (29%)	166 (17%)
**Degree of urbanisation**													
Peri urban	1,177 (9.9%)	160 (16%)	152 (15%)	160 (16%)	25 (2.4%)	201 (20%)	69 (6.9%)	53 (5.3%)	95 (10.0%)	26 (2.6%)	82 (8.4%)	56 (5.5%)	100 (10%)
Rural	6,586 (55%)	678 (68%)	526 (53%)	519 (52%)	650 (64%)	340 (34%)	502 (50%)	470 (47%)	459 (48%)	575 (58%)	534 (55%)	681 (67%)	651 (65%)
Urban	4,149 (35%)	155 (16%)	322 (32%)	316 (32%)	339 (33%)	454 (46%)	428 (43%)	471 (47%)	396 (42%)	395 (40%)	351 (36%)	280 (28%)	243 (24%)

Table 33: Coding scheme for all independent variables included in the multivariable disclosure models, organised by ecological level. Micro-level variables capture demographic factors (age, gender). Meso-level variables capture positive parental mediation, operationalised as four survey items assessing how frequently parents engage with the child's online activities (1–5 scale; E1). Macro-level variables capture community-level gender norms, including attitudes toward premarital sex (Yes/No; I7) and a three-item gender inequitable attitudes index (0–3; I7) assessing beliefs about male sexual decision-making, male reputation, and tolerance of intimate partner violence. Protective factor variables capture sex education (Yes/No; A16), digital skills to report harmful content (1–4 scale; D1), and help-seeking knowledge (Yes/No; G17).

Factors	Independent Variables	Response Options	Variable
**Micro (individual)**	**Age**	· Age of Selected child	12-17 years age
	**Gender**	· Gender of Selected child	Boys (0)<br>Girls (1)
**Meso (family)**	**Parental mediation**	· E1. When you use the internet, how often does your parent/carer/guardian do any of these things? - Encourages me to explore and learn things on the internet<br>· E1. When you use the internet, how often does your parent/carer/guardian do any of these things? - Suggests ways to use the internet safely<br>· E1. When you use the internet, how often does your parent/carer/guardian do any of these things? - Helps me when something bothers me on the internet<br>· E1. When you use the internet, how often does your parent/carer/guardian do any of these things? - Does shared activities together with me on the internet	1 to 5 scale
**Macro (community)**	**Premarital sex**	· I7. Do you believe that: - Having sex before marriage is acceptable?	Yes/No
	**Gender inequitable attitudes**	· I7. Do you believe that: - Only men, not women, should decide when to have sex?<br>· I7. Do you believe that: - If someone insults a boy or man, he should defend his reputation with force if he needs to?<br>· I7. Do you believe that: - A woman should tolerate violence to keep her family together?	Index (0-3).
**Protective factors**	**Sex-education**	· A16. Have you received any sex education?	Yes/No
	**Digital skills to report**	· D1. Think about how you use the internet. How true are these things for you? - I know how to report harmful content relating to me or a group to which	1 to 4 scale
	**Help-seeking**	· G17. Do you know where to get help if you or a friend experience sexual assault or sexual harassment?	Yes/No

Table 34: Role-based classification of disclosure channels used in the disclosure analysis, distinguishing formal from informal channels based on the recipient's institutional position and relationship to statutory child-protection systems. Formal channels included recipients with statutory duties or formal referral pathways (police, social workers, teachers, helplines); informal channels include recipients providing personal or familial support without statutory mandate (parents, siblings, friends, other trusted adults)

Channel type	Role-based definition	Typical recipients	Link to statutory system	Boundary/fluidity	Key references
**Formal**	Positioned within or directly linked to child-protection systems; recipients have statutory duties or formal referral pathways	Police; social services; teachers; helplines	Typically embedded in, or mandated to interface with, statutory protection	Not all disclosures to "formal" recipients trigger an investigation; roles vary across LMIC contexts	Paine & Hansen (2002); Gilbert et al. (2011); Larner (2022)
**Informal**	Provide personal, familial, or peer support without statutory mandate	Parents/caregivers; siblings; friends; other trusted adults	Not typically linked to statutory systems	Often the first line of disclosure; may facilitate (or impede) movement to formal channels	Paine & Hansen (2002); McElvaney (2015)

Table 35: Survey-weighted descriptive statistics for independent variables among internet-using children aged 12–17 in the Africa subsample (Ethiopia, Kenya, Mozambique, Namibia, Tanzania, Uganda). Variables are grouped into thematic domains: protective factors (help-seeking, sex education, digital skills), gender norms and attitudes (premarital sex, inequitable attitudes toward sex: men decide, male reputation, tolerance of violence against women), and enabling parental mediation (five items capturing parental engagement with the child's online activities). Responses are presented in original coded form without recoding, binary items are shown as Yes/No/Don't know/Prefer not to say, and Likert-type items retain their original response scale. Proportions are survey-weighted to reflect national population estimates of internet-using children within each country. The parental mediation item 'help with difficult online tasks' (pm_difficult) had elevated missingness across countries and is retained for transparency.

Responses	Overall N = 6,019[1]	Ethiopia N = 1,000[1]	Kenya N = 1,014[1]	Mozambique N = 999[1]	Namibia N = 994[1]	Tanzania N = 996[1]	Uganda N = 1,016[1]
**Do you know where to get help if you or a friend experience sexual assault or sexual harassment?**							
No	3,561 (59%)	572 (57%)	618 (61%)	670 (67%)	477 (48%)	666 (67%)	559 (55%)
Yes	2,296 (38%)	381 (38%)	370 (37%)	292 (29%)	505 (51%)	316 (32%)	432 (42%)
Prefer not to say	163 (2.7%)	48 (4.8%)	26 (2.5%)	38 (3.8%)	12 (1.2%)	14 (1.4%)	25 (2.5%)
**Have you received any sex education?**							
No	2,994 (50%)	486 (49%)	464 (46%)	642 (64%)	367 (37%)	684 (69%)	351 (35%)
Yes	2,825 (47%)	483 (48%)	542 (53%)	283 (28%)	579 (58%)	295 (30%)	643 (63%)
Prefer not to say	69 (1.2%)	2 (0.2%)	1 (0.1%)	28 (2.8%)	24 (2.4%)	4 (0.4%)	9 (0.9%)
Don't know	131 (2.2%)	29 (2.9%)	6 (0.6%)	46 (4.6%)	24 (2.5%)	13 (1.3%)	13 (1.3%)
**Having sex before marriage is acceptable?**							
No	4,017 (67%)	808 (81%)	783 (77%)	551 (55%)	339 (34%)	805 (81%)	731 (72%)
Yes	1,167 (19%)	86 (8.6%)	150 (15%)	303 (30%)	356 (36%)	88 (8.8%)	185 (18%)
Prefer not to say	190 (3.2%)	31 (3.1%)	27 (2.6%)	47 (4.7%)	62 (6.2%)	6 (0.6%)	18 (1.8%)
I am not sure	644 (11%)	75 (7.5%)	54 (5.3%)	98 (9.9%)	238 (24%)	97 (9.8%)	82 (8.1%)
**I know how to report harmful content on social media**							
Not at all true for me	2,729 (45%)	380 (38%)	622 (61%)	309 (31%)	223 (22%)	540 (54%)	656 (65%)
Not very true for me	960 (16%)	190 (19%)	130 (13%)	124 (12%)	130 (13%)	274 (28%)	113 (11%)

Responses	Overall N = 6,019[1]	Ethiopia N = 1,000[1]	Kenya N = 1,014[1]	Mozambique N = 999[1]	Namibia N = 994[1]	Tanzania N = 996[1]	Uganda N = 1,016[1]
Mostly true for me	855 (14%)	189 (19%)	91 (9.0%)	291 (29%)	181 (18%)	37 (3.7%)	67 (6.6%)
Very true for me	1,113 (18%)	172 (17%)	151 (15%)	137 (14%)	438 (44%)	87 (8.8%)	127 (13%)
I am not sure	66 (1.1%)	14 (1.4%)	5 (0.5%)	39 (3.9%)	4 (0.4%)	0 (0%)	5 (0.4%)
Prefer not to say	296 (4.9%)	56 (5.6%)	15 (1.5%)	100 (10%)	18 (1.9%)	58 (5.8%)	48 (4.8%)
**Only men, not women, should decide when to have sex?**							
No	3,814 (63%)	814 (81%)	677 (67%)	687 (69%)	455 (46%)	653 (66%)	529 (52%)
Yes	948 (16%)	86 (8.6%)	144 (14%)	171 (17%)	213 (21%)	123 (12%)	212 (21%)
Prefer not to say	265 (4.4%)	36 (3.6%)	36 (3.5%)	47 (4.7%)	79 (8.0%)	25 (2.5%)	43 (4.2%)
I am not sure	992 (16%)	64 (6.4%)	157 (16%)	95 (9.5%)	247 (25%)	196 (20%)	233 (23%)
**If someone insults a boy or man, he should defend his reputation with force if he needs to?**							
No	3,419 (57%)	730 (73%)	564 (56%)	665 (67%)	362 (36%)	571 (57%)	527 (52%)
Yes	1,353 (22%)	162 (16%)	261 (26%)	205 (20%)	210 (21%)	243 (24%)	271 (27%)
Prefer not to say	236 (3.9%)	27 (2.7%)	42 (4.1%)	40 (4.0%)	74 (7.5%)	14 (1.4%)	40 (3.9%)
I am not sure	1,011 (17%)	81 (8.1%)	147 (14%)	90 (9.0%)	347 (35%)	168 (17%)	178 (18%)
**A woman should tolerate violence to keep her family together?**							
No	3,654 (61%)	596 (60%)	584 (58%)	768 (77%)	712 (72%)	543 (55%)	450 (44%)
Yes	1,535 (26%)	291 (29%)	315 (31%)	120 (12%)	92 (9.3%)	306 (31%)	412 (41%)
Prefer not to say	188 (3.1%)	36 (3.6%)	22 (2.2%)	38 (3.8%)	43 (4.4%)	18 (1.8%)	31 (3.0%)
I am not sure	642 (11%)	77 (7.7%)	94 (9.2%)	73 (7.3%)	147 (15%)	128 (13%)	123 (12%)
**Does shared activities together with me on the internet**							
Never	3,358 (56%)	637 (64%)	571 (56%)	627 (63%)	247 (25%)	707 (71%)	569 (56%)
Rarely	817 (14%)	120 (12%)	137 (14%)	158 (16%)	97 (9.8%)	157 (16%)	147 (14%)
Sometimes	1,042 (17%)	127 (13%)	210 (21%)	132 (13%)	319 (32%)	45 (4.5%)	207 (20%)
Often	460 (7.6%)	50 (5.0%)	57 (5.6%)	32 (3.2%)	229 (23%)	45 (4.5%)	47 (4.6%)
Very often	185 (3.1%)	14 (1.4%)	20 (1.9%)	17 (1.8%)	89 (9.0%)	20 (2.0%)	25 (2.4%)

Responses	Overall<br>N = 6,019[1]	Ethiopia<br>N = 1,000[1]	Kenya<br>N = 1,014[1]	Mozambique<br>N = 999[1]	Namibia<br>N = 994[1]	Tanzania<br>N = 996[1]	Uganda<br>N = 1,016[1]
Prefer not to say	54 (0.9%)	7 (0.7%)	10 (1.0%)	19 (1.9%)	7 (0.7%)	3 (0.3%)	8 (0.8%)
Don't know	111 (1.8%)	49 (4.9%)	8 (0.8%)	13 (1.3%)	8 (0.8%)	19 (1.9%)	14 (1.4%)
**Suggests ways to use the internet safely**							
Never	2,390 (40%)	458 (46%)	335 (33%)	480 (48%)	197 (20%)	521 (52%)	399 (39%)
Rarely	1,029 (17%)	176 (18%)	175 (17%)	193 (19%)	95 (9.6%)	212 (21%)	178 (18%)
Sometimes	1,149 (19%)	156 (16%)	252 (25%)	169 (17%)	290 (29%)	46 (4.6%)	236 (23%)
Often	836 (14%)	114 (11%)	150 (15%)	70 (7.0%)	261 (26%)	133 (13%)	109 (11%)
Very often	431 (7.2%)	40 (4.0%)	79 (7.8%)	43 (4.3%)	143 (14%)	56 (5.6%)	68 (6.7%)
Prefer not to say	46 (0.8%)	5 (0.5%)	8 (0.8%)	25 (2.5%)	4 (0.4%)	1 (<0.1%)	4 (0.4%)
Don't know	139 (2.3%)	51 (5.1%)	15 (1.4%)	20 (2.0%)	4 (0.4%)	28 (2.8%)	21 (2.1%)
**Encourages me to explore and learn things on the internet**							
Never	2,565 (43%)	458 (46%)	416 (41%)	471 (47%)	233 (23%)	616 (62%)	371 (37%)
Rarely	1,154 (19%)	180 (18%)	194 (19%)	234 (23%)	75 (7.5%)	247 (25%)	224 (22%)
Sometimes	1,291 (21%)	188 (19%)	269 (27%)	180 (18%)	327 (33%)	53 (5.3%)	274 (27%)
Often	622 (10%)	98 (9.8%)	96 (9.5%)	41 (4.1%)	262 (26%)	52 (5.2%)	73 (7.2%)
Very often	249 (4.1%)	35 (3.5%)	29 (2.8%)	35 (3.5%)	90 (9.0%)	11 (1.1%)	50 (4.9%)
Prefer not to say	50 (0.8%)	8 (0.8%)	6 (0.6%)	24 (2.4%)	1 (0.1%)	2 (0.2%)	9 (0.9%)
Don't know	88 (1.5%)	34 (3.4%)	4 (0.4%)	13 (1.3%)	7 (0.7%)	15 (1.5%)	15 (1.5%)
pm_difficult	NA (NA, NA)	NA (NA, NA)	NA (NA, NA)	NA (NA, NA)	NA (NA, NA)	NA (NA, NA)	NA (NA, NA)
Unknown	6,019	1,000	1,014	999	994	996	1,016
**Helps me when something bothers me on the internet**							
Never	3,037 (50%)	575 (58%)	487 (48%)	555 (56%)	253 (25%)	650 (65%)	516 (51%)
Rarely	914 (15%)	144 (14%)	156 (15%)	180 (18%)	105 (11%)	191 (19%)	137 (13%)
Sometimes	1,026 (17%)	138 (14%)	231 (23%)	154 (15%)	233 (23%)	51 (5.1%)	220 (22%)
Often	575 (9.6%)	51 (5.1%)	90 (8.9%)	46 (4.6%)	255 (26%)	54 (5.4%)	80 (7.9%)

Responses	Overall N = 6,019[1]	Ethiopia N = 1,000[1]	Kenya N = 1,014[1]	Mozambique N = 999[1]	Namibia N = 994[1]	Tanzania N = 996[1]	Uganda N = 1,016[1]
Very often	274 (4.5%)	24 (2.4%)	34 (3.3%)	23 (2.3%)	124 (12%)	27 (2.7%)	42 (4.1%)
Prefer not to say	49 (0.8%)	12 (1.2%)	6 (0.6%)	16 (1.6%)	8 (0.8%)	3 (0.3%)	4 (0.4%)
Don't know	144 (2.4%)	56 (5.6%)	10 (1.0%)	25 (2.5%)	16 (1.6%)	20 (2.0%)	17 (1.7%)

Table 36: Survey-weighted descriptive statistics for independent variables among internet-using children aged 12–17 in the Asia subsample (Cambodia, Indonesia, Malaysia, Thailand, Philippines, Vietnam). Variables and thematic groupings mirror those reported for the Africa subsample in Table 35, comprising protective factors (help-seeking, sex education, digital skills), gender norms and attitudes (premarital sex, inequitable attitudes toward sex: men decide, male reputation, tolerance of violence against women), and enabling parental mediation (five items capturing parental engagement with the child's online activities). Responses are presented in original coded form without recoding. Proportions are survey-weighted to reflect national population estimates of internet-using children within each country.

	Overall N = 5,893[1]	Cambodia N = 992[1]	Indonesia N = 995[1]	Malaysia N = 995[1]	Philippines N = 950[1]	Thailand N = 967[1]	Vietnam N = 994[1]
**Do you know where to get help if you or a friend experience sexual assault or sexual harassment?**							
No	3,456 (59%)	658 (66%)	618 (62%)	553 (56%)	421 (44%)	451 (47%)	754 (76%)
Yes	2,037 (35%)	215 (22%)	324 (33%)	385 (39%)	437 (46%)	457 (47%)	218 (22%)
Prefer not to say	400 (6.8%)	118 (12%)	53 (5.4%)	56 (5.7%)	91 (9.6%)	59 (6.1%)	22 (2.2%)
**Have you received any sex education?**							
No	3,193 (54%)	689 (69%)	719 (72%)	592 (59%)	783 (82%)	190 (20%)	221 (22%)
Yes	2,177 (37%)	269 (27%)	203 (20%)	291 (29%)	85 (9.0%)	665 (69%)	663 (67%)
Prefer not to say	240 (4.1%)	5 (0.5%)	36 (3.6%)	59 (5.9%)	27 (2.8%)	73 (7.5%)	41 (4.1%)
Don't know	282 (4.8%)	29 (2.9%)	37 (3.7%)	53 (5.3%)	55 (5.8%)	39 (4.1%)	70 (7.0%)
**Having sex before marriage is acceptable?**							
No	3,818 (65%)	672 (68%)	838 (84%)	729 (73%)	651 (69%)	513 (53%)	415 (42%)
Yes	840 (14%)	102 (10%)	47 (4.7%)	93 (9.3%)	82 (8.6%)	301 (31%)	216 (22%)
Prefer not to say	372 (6.3%)	34 (3.4%)	89 (8.9%)	32 (3.2%)	58 (6.1%)	29 (3.0%)	130 (13%)
I am not sure	863 (15%)	185 (19%)	22 (2.2%)	141 (14%)	158 (17%)	125 (13%)	233 (23%)

	Overall N = 5,893[1]	Cambodia N = 992[1]	Indonesia N = 995[1]	Malaysia N = 995[1]	Philippines N = 950[1]	Thailand N = 967[1]	Vietnam N = 994[1]
**I know how to report harmful content on social media**							
Not at all true for me	1,100 (19%)	455 (46%)	242 (24%)	89 (8.9%)	179 (19%)	59 (6.1%)	76 (7.7%)
Not very true for me	791 (13%)	93 (9.4%)	153 (15%)	202 (20%)	148 (16%)	105 (11%)	90 (9.1%)
Mostly true for me	1,877 (32%)	245 (25%)	203 (20%)	371 (37%)	159 (17%)	471 (49%)	427 (43%)
Very true for me	1,574 (27%)	89 (9.0%)	282 (28%)	285 (29%)	358 (38%)	281 (29%)	279 (28%)
I am not sure	51 (0.9%)	4 (0.4%)	7 (0.7%)	7 (0.7%)	11 (1.1%)	12 (1.2%)	11 (1.1%)
Prefer not to say	499 (8.5%)	106 (11%)	108 (11%)	42 (4.2%)	94 (9.9%)	39 (4.1%)	110 (11%)
**Only men, not women, should decide when to have sex?**							
No	3,411 (58%)	519 (52%)	581 (58%)	643 (65%)	453 (48%)	692 (72%)	523 (53%)
Yes	545 (9.2%)	86 (8.7%)	78 (7.8%)	47 (4.7%)	88 (9.3%)	125 (13%)	120 (12%)
Prefer not to say	598 (10%)	37 (3.7%)	248 (25%)	61 (6.1%)	108 (11%)	24 (2.4%)	120 (12%)
I am not sure	1,339 (23%)	350 (35%)	88 (8.8%)	245 (25%)	300 (32%)	127 (13%)	230 (23%)
**If someone insults a boy or man, he should defend his reputation with force if he needs to?**							
No	2,782 (47%)	458 (46%)	532 (53%)	512 (52%)	248 (26%)	666 (69%)	366 (37%)
Yes	1,330 (23%)	213 (21%)	194 (19%)	226 (23%)	378 (40%)	148 (15%)	171 (17%)
Prefer not to say	508 (8.6%)	33 (3.3%)	182 (18%)	38 (3.8%)	84 (8.9%)	32 (3.3%)	139 (14%)
I am not sure	1,273 (22%)	288 (29%)	87 (8.7%)	218 (22%)	240 (25%)	122 (13%)	318 (32%)
**A woman should tolerate violence to keep her family together?**							
No	3,493 (59%)	540 (54%)	412 (41%)	578 (58%)	492 (52%)	712 (74%)	759 (76%)
Yes	913 (15%)	185 (19%)	293 (29%)	135 (14%)	159 (17%)	114 (12%)	27 (2.7%)
Prefer not to say	452 (7.7%)	32 (3.2%)	201 (20%)	43 (4.4%)	77 (8.1%)	27 (2.8%)	72 (7.3%)

	Overall N = 5,893[1]	Cambodia N = 992[1]	Indonesia N = 995[1]	Malaysia N = 995[1]	Philippines N = 950[1]	Thailand N = 967[1]	Vietnam N = 994[1]
I am not sure	1,035 (18%)	234 (24%)	89 (8.9%)	239 (24%)	223 (23%)	115 (12%)	136 (14%)
**Does shared activities together with me on the internet**							
Never	1,656 (28%)	565 (57%)	277 (28%)	172 (17%)	239 (25%)	126 (13%)	277 (28%)
Rarely	1,250 (21%)	180 (18%)	271 (27%)	277 (28%)	198 (21%)	73 (7.6%)	251 (25%)
Sometimes	1,786 (30%)	170 (17%)	218 (22%)	328 (33%)	348 (37%)	394 (41%)	328 (33%)
Often	873 (15%)	50 (5.1%)	182 (18%)	163 (16%)	110 (12%)	259 (27%)	109 (11%)
Very often	211 (3.6%)	4 (0.4%)	36 (3.7%)	42 (4.3%)	20 (2.1%)	96 (9.9%)	12 (1.2%)
Prefer not to say	35 (0.6%)	3 (0.3%)	4 (0.4%)	4 (0.4%)	9 (1.0%)	11 (1.2%)	3 (0.3%)
Don't know	83 (1.4%)	19 (1.9%)	8 (0.8%)	9 (0.9%)	26 (2.7%)	8 (0.8%)	14 (1.4%)
**Suggests ways to use the internet safely**							
Never	1,257 (21%)	512 (52%)	153 (15%)	100 (10%)	85 (9.0%)	117 (12%)	288 (29%)
Rarely	866 (15%)	155 (16%)	201 (20%)	199 (20%)	60 (6.3%)	59 (6.1%)	192 (19%)
Sometimes	1,680 (29%)	193 (19%)	219 (22%)	307 (31%)	339 (36%)	303 (31%)	319 (32%)
Often	1,483 (25%)	89 (9.0%)	325 (33%)	276 (28%)	327 (34%)	330 (34%)	135 (14%)
Very often	445 (7.5%)	11 (1.1%)	73 (7.3%)	92 (9.3%)	106 (11%)	134 (14%)	29 (2.9%)
Prefer not to say	44 (0.8%)	7 (0.7%)	3 (0.3%)	3 (0.3%)	6 (0.6%)	13 (1.3%)	12 (1.2%)
Don't know	104 (1.8%)	24 (2.4%)	16 (1.6%)	17 (1.7%)	24 (2.5%)	9 (0.9%)	14 (1.4%)
**Encourages me to explore and learn things on the internet**							
Never	1,084 (18%)	378 (38%)	188 (19%)	130 (13%)	128 (13%)	115 (12%)	145 (15%)
Rarely	1,059 (18%)	224 (23%)	264 (27%)	224 (22%)	96 (10%)	57 (5.9%)	194 (20%)
Sometimes	2,013 (34%)	230 (23%)	229 (23%)	383 (39%)	453 (48%)	336 (35%)	383 (39%)
Often	1,315 (22%)	130 (13%)	257 (26%)	177 (18%)	204 (21%)	316 (33%)	231 (23%)
Very often	273 (4.6%)	14 (1.5%)	45 (4.5%)	49 (4.9%)	25 (2.7%)	119 (12%)	21 (2.1%)
Prefer not to say	55 (0.9%)	7 (0.7%)	2 (0.2%)	9 (0.9%)	14 (1.5%)	17 (1.7%)	6 (0.6%)
Don't know	93 (1.6%)	9 (0.9%)	10 (1.0%)	22 (2.3%)	30 (3.1%)	7 (0.8%)	14 (1.4%)

	Overall N = 5,893[1]	Cambodia N = 992[1]	Indonesia N = 995[1]	Malaysia N = 995[1]	Philippines N = 950[1]	Thailand N = 967[1]	Vietnam N = 994[1]
**Helps me when something is difficult to do or find on the internet**							
Never	789 (39%)	535 (58%)	80 (27%)	0 (NA%)	0 (NA%)	34 (9.7%)	139 (31%)
Rarely	350 (17%)	165 (18%)	59 (20%)	0 (NA%)	0 (NA%)	33 (9.4%)	93 (21%)
Sometimes	511 (25%)	147 (16%)	64 (21%)	0 (NA%)	0 (NA%)	165 (47%)	135 (30%)
Often	275 (14%)	50 (5.4%)	76 (25%)	0 (NA%)	0 (NA%)	85 (24%)	64 (14%)
Very often	54 (2.7%)	4 (0.4%)	18 (5.9%)	0 (NA%)	0 (NA%)	22 (6.3%)	10 (2.3%)
Prefer not to say	18 (0.9%)	6 (0.6%)	4 (1.2%)	0 (NA%)	0 (NA%)	9 (2.5%)	0 (0%)
Don't know	32 (1.6%)	19 (2.0%)	0 (0%)	0 (NA%)	0 (NA%)	4 (1.2%)	8 (1.8%)
Unknown	3,864	67	695	995	950	613	545
**Helps me when something bothers me on the internet**							
Never	1,704 (29%)	635 (64%)	254 (26%)	174 (18%)	162 (17%)	118 (12%)	360 (36%)
Rarely	976 (17%)	170 (17%)	239 (24%)	187 (19%)	109 (11%)	96 (10.0%)	175 (18%)
Sometimes	1,679 (28%)	120 (12%)	202 (20%)	280 (28%)	397 (42%)	453 (47%)	228 (23%)
Often	1,055 (18%)	32 (3.3%)	218 (22%)	237 (24%)	196 (21%)	224 (23%)	148 (15%)
Very often	260 (4.4%)	6 (0.6%)	48 (4.8%)	77 (7.8%)	41 (4.4%)	57 (5.9%)	31 (3.1%)
Prefer not to say	58 (1.0%)	3 (0.3%)	11 (1.1%)	12 (1.2%)	11 (1.2%)	12 (1.3%)	9 (0.9%)
Don't know	160 (2.7%)	26 (2.6%)	24 (2.4%)	28 (2.8%)	33 (3.5%)	6 (0.6%)	43 (4.3%)

Table 37: Distribution of Likert-scale responses for nine forms of technology-facilitated CSEA among internet-using children aged 12–17 (N = 11,912; 2020–2021). For each item, survey-weighted proportions are reported across five frequency categories (Never, Rarely, Sometimes, Often, Very Often) as well as 'Prefer not to say' and 'Don't know' responses. Proportions are row percentages and sum to 100% within each item.

Responses	Overall N = 11,912[1]	Cambodia N = 992[1]	Ethiopia N = 1,000[1]	Indonesia N = 995[1]	Kenya N = 1,014[1]	Malaysia N = 995[1]	Mozambique N = 999[1]	Namibia N = 994[1]	Philippines N = 950[1]	Tanzania N = 996[1]	Thailand N = 967[1]	Uganda N = 1,016[1]	Vietnam N = 994[1]
**Someone made sexual comments about me (e.g. jokes, stories or comments about my body, appearance or sexual activities) that made me feel uncomfortable**													
Never	9,488 (80%)	771 (78%)	715 (72%)	889 (89%)	777 (77%)	870 (87%)	766 (77%)	806 (81%)	622 (65%)	891 (89%)	775 (80%)	726 (72%)	880 (89%)
Rarely	898 (7.5%)	87 (8.8%)	95 (9.5%)	47 (4.7%)	88 (8.6%)	58 (5.8%)	71 (7.1%)	62 (6.3%)	156 (16%)	38 (3.8%)	46 (4.7%)	82 (8.1%)	68 (6.8%)
Sometimes	775 (6.5%)	52 (5.3%)	66 (6.6%)	21 (2.1%)	92 (9.0%)	29 (2.9%)	105 (10%)	84 (8.5%)	96 (10%)	9 (0.9%)	76 (7.9%)	132 (13%)	12 (1.2%)
Often	172 (1.4%)	10 (1.0%)	25 (2.5%)	3 (0.3%)	21 (2.1%)	3 (0.3%)	11 (1.1%)	24 (2.4%)	13 (1.4%)	3 (0.3%)	30 (3.1%)	27 (2.7%)	2 (0.2%)
Very Often	63 (0.5%)	6 (0.6%)	5 (0.5%)	1 (<0.1%)	12 (1.1%)	1 (0.1%)	7 (0.7%)	5 (0.5%)	0 (0%)	0 (0%)	15 (1.5%)	12 (1.1%)	0 (0%)
Prefer not to say	169 (1.4%)	18 (1.8%)	11 (1.1%)	18 (1.8%)	12 (1.2%)	21 (2.2%)	23 (2.3%)	4 (0.4%)	27 (2.8%)	2 (0.2%)	12 (1.2%)	12 (1.2%)	10 (1.0%)
Don’t know	346 (2.9%)	48 (4.8%)	82 (8.2%)	17 (1.7%)	13 (1.2%)	13 (1.3%)	17 (1.7%)	10 (1.0%)	36 (3.8%)	53 (5.3%)	13 (1.3%)	24 (2.3%)	22 (2.3%)
**Someone sent me sexual images I did not want**													
Never	9,545 (80%)	772 (78%)	727 (73%)	891 (90%)	793 (78%)	882 (89%)	700 (70%)	827 (83%)	618 (65%)	895 (90%)	796 (82%)	723 (71%)	922 (93%)
Rarely	815 (6.8%)	75 (7.5%)	99 (9.9%)	36 (3.6%)	71 (7.0%)	60 (6.1%)	74 (7.4%)	55 (5.5%)	147 (15%)	39 (3.9%)	25 (2.6%)	89 (8.8%)	45 (4.5%)
Sometimes	813 (6.8%)	71 (7.1%)	54 (5.4%)	22 (2.2%)	98 (9.6%)	23 (2.3%)	135 (14%)	74 (7.4%)	108 (11%)	6 (0.6%)	64 (6.6%)	153 (15%)	6 (0.6%)
Often	175 (1.5%)	12 (1.2%)	26 (2.6%)	5 (0.5%)	20 (2.0%)	2 (0.2%)	20 (2.0%)	19 (1.9%)	21 (2.2%)	2 (0.2%)	34 (3.5%)	14 (1.3%)	1 (<0.1%)
Very Often	99 (0.8%)	3 (0.3%)	7 (0.7%)	1 (<0.1%)	11 (1.1%)	0 (0%)	30 (3.0%)	8 (0.8%)	3 (0.3%)	2 (0.2%)	27 (2.8%)	7 (0.7%)	0 (0%)
Prefer not to say	184 (1.5%)	12 (1.2%)	14 (1.4%)	29 (2.9%)	13 (1.3%)	19 (1.9%)	33 (3.4%)	5 (0.5%)	17 (1.8%)	3 (0.3%)	10 (1.1%)	18 (1.8%)	10 (1.0%)
Don’t know	280 (2.4%)	47 (4.8%)	73 (7.3%)	12 (1.2%)	8 (0.8%)	8 (0.8%)	7 (0.7%)	6 (0.6%)	36 (3.8%)	49 (4.9%)	10 (1.0%)	12 (1.2%)	11 (1.1%)
**I have been asked to talk about sex or sexual acts with someone when I did not want to**													
Never	10,199 (86%)	839 (85%)	780 (78%)	934 (94%)	861 (85%)	903 (91%)	848 (85%)	890 (90%)	719 (76%)	881 (88%)	827 (86%)	758 (75%)	958 (96%)
Rarely	614 (5.2%)	55 (5.5%)	62 (6.2%)	17 (1.7%)	58 (5.7%)	34 (3.4%)	46 (4.6%)	33 (3.3%)	116 (12%)	47 (4.7%)	32 (3.3%)	97 (9.5%)	18 (1.8%)
Sometimes	379 (3.2%)	26 (2.7%)	44 (4.4%)	5 (0.5%)	47 (4.6%)	9 (0.9%)	33 (3.3%)	36 (3.6%)	38 (4.0%)	6 (0.6%)	34 (3.5%)	100 (9.8%)	1 (<0.1%)
Often	100 (0.8%)	6 (0.6%)	10 (1.0%)	0 (0%)	10 (1.0%)	2 (0.2%)	22 (2.2%)	9 (1.0%)	7 (0.8%)	3 (0.3%)	18 (1.8%)	12 (1.2%)	0 (0%)
Very Often	67 (0.6%)	5 (0.5%)	4 (0.4%)	1 (0.1%)	12 (1.2%)	1 (0.1%)	9 (0.9%)	6 (0.6%)	2 (0.3%)	1 (<0.1%)	18 (1.8%)	7 (0.7%)	0 (0%)
Prefer not to say	237 (2.0%)	13 (1.3%)	17 (1.7%)	28 (2.8%)	13 (1.3%)	28 (2.8%)	35 (3.5%)	9 (0.9%)	25 (2.6%)	7 (0.7%)	33 (3.4%)	19 (1.9%)	11 (1.1%)
Don’t know	316 (2.7%)	48 (4.8%)	83 (8.3%)	10 (1.0%)	12 (1.2%)	18 (1.8%)	5 (0.5%)	10 (1.0%)	42 (4.5%)	53 (5.3%)	6 (0.7%)	23 (2.3%)	6 (0.6%)

Responses	Overall N = 11,912[1]	Cambodia N = 992[1]	Ethiopia N = 1,000[1]	Indonesia N = 995[1]	Kenya N = 1,014[1]	Malaysia N = 995[1]	Mozambique N = 999[1]	Namibia N = 994[1]	Philippines N = 950[1]	Tanzania N = 996[1]	Thailand N = 967[1]	Uganda N = 1,016[1]	Vietnam N = 994[1]
**I have been asked by someone to do something sexual when I did not want to**													
Never	10,335 (87%)	829 (84%)	787 (79%)	946 (95%)	851 (84%)	935 (94%)	859 (86%)	908 (91%)	761 (80%)	861 (86%)	837 (87%)	796 (78%)	966 (97%)
Rarely	558 (4.7%)	58 (5.8%)	60 (6.0%)	3 (0.3%)	56 (5.6%)	23 (2.3%)	51 (5.1%)	33 (3.3%)	108 (11%)	60 (6.0%)	24 (2.5%)	69 (6.8%)	12 (1.2%)
Sometimes	336 (2.8%)	30 (3.0%)	43 (4.3%)	6 (0.6%)	59 (5.8%)	3 (0.3%)	35 (3.5%)	18 (1.8%)	21 (2.2%)	11 (1.1%)	22 (2.3%)	86 (8.5%)	0 (<0.1%)
Often	106 (0.9%)	3 (0.3%)	13 (1.3%)	2 (0.2%)	12 (1.2%)	4 (0.4%)	3 (0.3%)	11 (1.1%)	6 (0.7%)	7 (0.7%)	26 (2.7%)	18 (1.8%)	0 (0%)
Very Often	61 (0.5%)	4 (0.4%)	2 (0.2%)	0 (0%)	14 (1.3%)	0 (0%)	9 (0.9%)	3 (0.3%)	5 (0.5%)	1 (<0.1%)	15 (1.5%)	8 (0.8%)	0 (0%)
Prefer not to say	223 (1.9%)	11 (1.1%)	13 (1.3%)	28 (2.8%)	12 (1.2%)	20 (2.1%)	37 (3.7%)	13 (1.3%)	19 (2.0%)	3 (0.3%)	37 (3.8%)	21 (2.0%)	8 (0.8%)
Don't know	294 (2.5%)	57 (5.8%)	81 (8.1%)	9 (0.9%)	10 (1.0%)	10 (1.0%)	4 (0.4%)	7 (0.7%)	30 (3.2%)	53 (5.3%)	6 (0.6%)	18 (1.7%)	8 (0.8%)
**I have been asked for a photo or video showing my private parts when I did not want to**													
Never	10,529 (88%)	840 (85%)	818 (82%)	954 (96%)	886 (87%)	932 (94%)	847 (85%)	895 (90%)	757 (80%)	918 (92%)	855 (88%)	854 (84%)	974 (98%)
Rarely	422 (3.5%)	50 (5.1%)	37 (3.7%)	3 (0.3%)	35 (3.5%)	20 (2.0%)	54 (5.4%)	33 (3.3%)	98 (10%)	20 (2.0%)	13 (1.3%)	56 (5.5%)	4 (0.4%)
Sometimes	319 (2.7%)	29 (2.9%)	35 (3.5%)	6 (0.6%)	52 (5.1%)	4 (0.4%)	36 (3.6%)	35 (3.5%)	39 (4.1%)	3 (0.3%)	22 (2.3%)	57 (5.7%)	0 (<0.1%)
Often	84 (0.7%)	4 (0.4%)	9 (0.9%)	3 (0.3%)	7 (0.7%)	1 (0.1%)	6 (0.6%)	13 (1.3%)	6 (0.6%)	1 (0.1%)	30 (3.1%)	5 (0.5%)	0 (0%)
Very Often	60 (0.5%)	5 (0.5%)	4 (0.4%)	0 (0%)	7 (0.7%)	0 (0%)	16 (1.6%)	7 (0.7%)	3 (0.3%)	2 (0.2%)	8 (0.8%)	8 (0.8%)	0 (0%)
Prefer not to say	210 (1.8%)	15 (1.5%)	9 (0.9%)	22 (2.3%)	16 (1.6%)	28 (2.8%)	34 (3.4%)	5 (0.5%)	19 (2.0%)	2 (0.2%)	31 (3.2%)	20 (1.9%)	10 (1.0%)
Don't know	289 (2.4%)	49 (4.9%)	89 (8.9%)	7 (0.7%)	11 (1.1%)	9 (0.9%)	6 (0.6%)	7 (0.7%)	29 (3.0%)	50 (5.0%)	9 (0.9%)	17 (1.6%)	6 (0.6%)
**Someone offered me money or gifts in return for sexual images or videos**													
Never	10,819 (91%)	867 (87%)	848 (85%)	965 (97%)	922 (91%)	951 (96%)	886 (89%)	926 (93%)	800 (84%)	928 (93%)	863 (89%)	879 (86%)	985 (99%)
Rarely	291 (2.4%)	27 (2.7%)	32 (3.2%)	4 (0.4%)	30 (3.0%)	12 (1.2%)	29 (2.9%)	15 (1.5%)	74 (7.8%)	14 (1.4%)	18 (1.9%)	35 (3.4%)	2 (0.2%)
Sometimes	248 (2.1%)	30 (3.0%)	28 (2.8%)	3 (0.4%)	25 (2.5%)	5 (0.5%)	41 (4.1%)	19 (1.9%)	19 (2.0%)	2 (0.2%)	19 (1.9%)	56 (5.5%)	0 (0%)
Often	75 (0.6%)	4 (0.4%)	5 (0.5%)	1 (0.1%)	9 (0.9%)	0 (0%)	3 (0.3%)	12 (1.2%)	5 (0.5%)	1 (0.1%)	26 (2.7%)	9 (0.9%)	0 (0%)
Very Often	38 (0.3%)	1 (0.1%)	9 (0.9%)	0 (0%)	3 (0.3%)	1 (0.1%)	4 (0.4%)	3 (0.3%)	2 (0.2%)	1 (0.1%)	8 (0.8%)	5 (0.5%)	0 (0%)
Prefer not to say	180 (1.5%)	13 (1.3%)	9 (0.9%)	18 (1.8%)	17 (1.7%)	15 (1.5%)	30 (3.0%)	3 (0.3%)	21 (2.3%)	1 (0.1%)	28 (2.9%)	20 (1.9%)	4 (0.4%)
Don't know	260 (2.2%)	49 (4.9%)	70 (7.0%)	3 (0.3%)	8 (0.7%)	11 (1.1%)	7 (0.7%)	15 (1.5%)	28 (2.9%)	48 (4.9%)	6 (0.6%)	12 (1.2%)	3 (0.3%)

Responses	Overall N = 11,912[1]	Cambodia N = 992[1]	Ethiopia N = 1,000[1]	Indonesia N = 995[1]	Kenya N = 1,014[1]	Malaysia N = 995[1]	Mozambique N = 999[1]	Namibia N = 994[1]	Philippines N = 950[1]	Tanzania N = 996[1]	Thailand N = 967[1]	Uganda N = 1,016[1]	Vietnam N = 994[1]
**I have been asked by someone to do something sexual when I did not want to**													
Never	10,335 (87%)	829 (84%)	787 (79%)	946 (95%)	851 (84%)	935 (94%)	859 (86%)	908 (91%)	761 (80%)	861 (86%)	837 (87%)	796 (78%)	966 (97%)
Rarely	558 (4.7%)	58 (5.8%)	60 (6.0%)	3 (0.3%)	56 (5.6%)	23 (2.3%)	51 (5.1%)	33 (3.3%)	108 (11%)	60 (6.0%)	24 (2.5%)	69 (6.8%)	12 (1.2%)
Sometimes	336 (2.8%)	30 (3.0%)	43 (4.3%)	6 (0.6%)	59 (5.8%)	3 (0.3%)	35 (3.5%)	18 (1.8%)	21 (2.2%)	11 (1.1%)	22 (2.3%)	86 (8.5%)	0 (<0.1%)
Often	106 (0.9%)	3 (0.3%)	13 (1.3%)	2 (0.2%)	12 (1.2%)	4 (0.4%)	3 (0.3%)	11 (1.1%)	6 (0.7%)	7 (0.7%)	26 (2.7%)	18 (1.8%)	0 (0%)
Very Often	61 (0.5%)	4 (0.4%)	2 (0.2%)	0 (0%)	14 (1.3%)	0 (0%)	9 (0.9%)	3 (0.3%)	5 (0.5%)	1 (<0.1%)	15 (1.5%)	8 (0.8%)	0 (0%)
Prefer not to say	223 (1.9%)	11 (1.1%)	13 (1.3%)	28 (2.8%)	12 (1.2%)	20 (2.1%)	37 (3.7%)	13 (1.3%)	19 (2.0%)	3 (0.3%)	37 (3.8%)	21 (2.0%)	8 (0.8%)
Don't know	294 (2.5%)	57 (5.8%)	81 (8.1%)	9 (0.9%)	10 (1.0%)	10 (1.0%)	4 (0.4%)	7 (0.7%)	30 (3.2%)	53 (5.3%)	6 (0.6%)	18 (1.7%)	8 (0.8%)
**I have been asked for a photo or video showing my private parts when I did not want to**													
Never	10,529 (88%)	840 (85%)	818 (82%)	954 (96%)	886 (87%)	932 (94%)	847 (85%)	895 (90%)	757 (80%)	918 (92%)	855 (88%)	854 (84%)	974 (98%)
Rarely	422 (3.5%)	50 (5.1%)	37 (3.7%)	3 (0.3%)	35 (3.5%)	20 (2.0%)	54 (5.4%)	33 (3.3%)	98 (10%)	20 (2.0%)	13 (1.3%)	56 (5.5%)	4 (0.4%)
Sometimes	319 (2.7%)	29 (2.9%)	35 (3.5%)	6 (0.6%)	52 (5.1%)	4 (0.4%)	36 (3.6%)	35 (3.5%)	39 (4.1%)	3 (0.3%)	22 (2.3%)	57 (5.7%)	0 (<0.1%)
Often	84 (0.7%)	4 (0.4%)	9 (0.9%)	3 (0.3%)	7 (0.7%)	1 (0.1%)	6 (0.6%)	13 (1.3%)	6 (0.6%)	1 (0.1%)	30 (3.1%)	5 (0.5%)	0 (0%)
Very Often	60 (0.5%)	5 (0.5%)	4 (0.4%)	0 (0%)	7 (0.7%)	0 (0%)	16 (1.6%)	7 (0.7%)	3 (0.3%)	2 (0.2%)	8 (0.8%)	8 (0.8%)	0 (0%)
Prefer not to say	210 (1.8%)	15 (1.5%)	9 (0.9%)	22 (2.3%)	16 (1.6%)	28 (2.8%)	34 (3.4%)	5 (0.5%)	19 (2.0%)	2 (0.2%)	31 (3.2%)	20 (1.9%)	10 (1.0%)
Don't know	289 (2.4%)	49 (4.9%)	89 (8.9%)	7 (0.7%)	11 (1.1%)	9 (0.9%)	6 (0.6%)	7 (0.7%)	29 (3.0%)	50 (5.0%)	9 (0.9%)	17 (1.6%)	6 (0.6%)
**Someone offered me money or gifts in return for sexual images or videos**													
Never	10,819 (91%)	867 (87%)	848 (85%)	965 (97%)	922 (91%)	951 (96%)	886 (89%)	926 (93%)	800 (84%)	928 (93%)	863 (89%)	879 (86%)	985 (99%)
Rarely	291 (2.4%)	27 (2.7%)	32 (3.2%)	4 (0.4%)	30 (3.0%)	12 (1.2%)	29 (2.9%)	15 (1.5%)	74 (7.8%)	14 (1.4%)	18 (1.9%)	35 (3.4%)	2 (0.2%)
Sometimes	248 (2.1%)	30 (3.0%)	28 (2.8%)	3 (0.4%)	25 (2.5%)	5 (0.5%)	41 (4.1%)	19 (1.9%)	19 (2.0%)	2 (0.2%)	19 (1.9%)	56 (5.5%)	0 (0%)
Often	75 (0.6%)	4 (0.4%)	5 (0.5%)	1 (0.1%)	9 (0.9%)	0 (0%)	3 (0.3%)	12 (1.2%)	5 (0.5%)	1 (0.1%)	26 (2.7%)	9 (0.9%)	0 (0%)
Very Often	38 (0.3%)	1 (0.1%)	9 (0.9%)	0 (0%)	3 (0.3%)	1 (0.1%)	4 (0.4%)	3 (0.3%)	2 (0.2%)	1 (0.1%)	8 (0.8%)	5 (0.5%)	0 (0%)
Prefer not to say	180 (1.5%)	13 (1.3%)	9 (0.9%)	18 (1.8%)	17 (1.7%)	15 (1.5%)	30 (3.0%)	3 (0.3%)	21 (2.3%)	1 (0.1%)	28 (2.9%)	20 (1.9%)	4 (0.4%)
Don't know	260 (2.2%)	49 (4.9%)	70 (7.0%)	3 (0.3%)	8 (0.7%)	11 (1.1%)	7 (0.7%)	15 (1.5%)	28 (2.9%)	48 (4.9%)	6 (0.6%)	12 (1.2%)	3 (0.3%)

Responses	Overall N = 11,912[1]	Cambodia N = 992[1]	Ethiopia N = 1,000[1]	Indonesia N = 995[1]	Kenya N = 1,014[1]	Malaysia N = 995[1]	Mozambique N = 999[1]	Namibia N = 994[1]	Philippines N = 950[1]	Tanzania N = 996[1]	Thailand N = 967[1]	Uganda N = 1,016[1]	Vietnam N = 994[1]
**Someone offered me money or gifts to meet them in person to do something sexual**													
Never	10,799 (91%)	874 (88%)	854 (85%)	964 (97%)	922 (91%)	950 (95%)	892 (89%)	924 (93%)	796 (84%)	918 (92%)	863 (89%)	857 (84%)	985 (99%)
Rarely	284 (2.4%)	29 (2.9%)	28 (2.8%)	6 (0.6%)	24 (2.4%)	10 (1.0%)	18 (1.8%)	21 (2.2%)	68 (7.2%)	21 (2.1%)	13 (1.3%)	42 (4.2%)	3 (0.3%)
Sometimes	236 (2.0%)	23 (2.3%)	19 (1.9%)	4 (0.4%)	29 (2.9%)	3 (0.3%)	30 (3.0%)	16 (1.7%)	24 (2.5%)	6 (0.6%)	16 (1.7%)	65 (6.4%)	0 (0%)
Often	104 (0.9%)	6 (0.6%)	16 (1.6%)	2 (0.2%)	7 (0.7%)	0 (0%)	8 (0.8%)	11 (1.1%)	12 (1.3%)	3 (0.3%)	28 (2.9%)	11 (1.0%)	0 (0%)
Very Often	48 (0.4%)	3 (0.3%)	4 (0.4%)	1 (0.1%)	6 (0.6%)	0 (0%)	9 (0.9%)	6 (0.6%)	1 (0.1%)	1 (0.1%)	9 (1.0%)	7 (0.7%)	0 (0%)
Prefer not to say	199 (1.7%)	11 (1.1%)	9 (0.9%)	15 (1.5%)	19 (1.9%)	21 (2.1%)	33 (3.3%)	6 (0.6%)	26 (2.8%)	2 (0.2%)	33 (3.4%)	18 (1.8%)	4 (0.4%)
Don't know	243 (2.0%)	46 (4.7%)	70 (7.0%)	3 (0.3%)	5 (0.5%)	10 (1.0%)	10 (1.0%)	9 (0.9%)	23 (2.4%)	44 (4.4%)	5 (0.5%)	15 (1.5%)	2 (0.2%)
**Someone shared sexual images of me without my consent**													
Never	10,740 (90%)	852 (86%)	858 (86%)	967 (97%)	899 (89%)	947 (95%)	893 (89%)	926 (93%)	749 (79%)	929 (93%)	864 (89%)	882 (87%)	974 (98%)
Rarely	303 (2.5%)	35 (3.5%)	20 (2.0%)	5 (0.5%)	31 (3.0%)	17 (1.7%)	23 (2.3%)	32 (3.2%)	83 (8.7%)	9 (0.9%)	19 (2.0%)	26 (2.5%)	3 (0.3%)
Sometimes	216 (1.8%)	26 (2.6%)	17 (1.7%)	4 (0.5%)	31 (3.1%)	9 (0.9%)	27 (2.7%)	10 (1.0%)	29 (3.1%)	2 (0.2%)	11 (1.1%)	49 (4.8%)	1 (<0.1%)
Often	79 (0.7%)	6 (0.6%)	11 (1.1%)	1 (0.1%)	6 (0.6%)	0 (0%)	7 (0.7%)	7 (0.7%)	9 (0.9%)	4 (0.4%)	18 (1.9%)	8 (0.8%)	1 (<0.1%)
Very Often	48 (0.4%)	7 (0.7%)	2 (0.2%)	0 (<0.1%)	3 (0.2%)	0 (0%)	4 (0.4%)	4 (0.4%)	2 (0.2%)	1 (<0.1%)	20 (2.1%)	6 (0.6%)	0 (<0.1%)
Prefer not to say	198 (1.7%)	19 (1.9%)	11 (1.1%)	14 (1.4%)	22 (2.2%)	10 (1.0%)	30 (3.0%)	5 (0.5%)	33 (3.5%)	4 (0.4%)	27 (2.8%)	15 (1.5%)	7 (0.7%)
Don't know	329 (2.8%)	48 (4.8%)	81 (8.1%)	3 (0.3%)	21 (2.1%)	13 (1.3%)	14 (1.4%)	10 (1.0%)	44 (4.7%)	47 (4.7%)	8 (0.8%)	31 (3.0%)	8 (0.8%)
**Someone threatened or blackmailed me to engage in sexual activities**													
Never	10,815 (91%)	882 (89%)	853 (85%)	964 (97%)	904 (89%)	962 (97%)	886 (89%)	927 (93%)	759 (80%)	930 (93%)	870 (90%)	894 (88%)	983 (99%)
Rarely	294 (2.5%)	20 (2.0%)	28 (2.8%)	4 (0.4%)	37 (3.6%)	9 (0.9%)	22 (2.2%)	31 (3.2%)	77 (8.1%)	13 (1.3%)	18 (1.9%)	33 (3.2%)	3 (0.3%)
Sometimes	182 (1.5%)	22 (2.2%)	13 (1.3%)	6 (0.6%)	24 (2.4%)	3 (0.3%)	24 (2.4%)	12 (1.2%)	29 (3.1%)	3 (0.3%)	8 (0.8%)	36 (3.5%)	0 (0%)
Often	82 (0.7%)	10 (1.0%)	12 (1.2%)	0 (<0.1%)	3 (0.3%)	0 (<0.1%)	8 (0.8%)	12 (1.2%)	9 (1.0%)	3 (0.3%)	20 (2.1%)	4 (0.4%)	0 (0%)
Very Often	63 (0.5%)	3 (0.3%)	2 (0.2%)	2 (0.2%)	7 (0.7%)	0 (0%)	14 (1.4%)	3 (0.3%)	3 (0.4%)	1 (<0.1%)	16 (1.7%)	11 (1.1%)	0 (<0.1%)
Prefer not to say	196 (1.6%)	11 (1.2%)	11 (1.1%)	17 (1.7%)	24 (2.4%)	15 (1.5%)	36 (3.6%)	3 (0.3%)	25 (2.7%)	3 (0.3%)	27 (2.8%)	18 (1.8%)	5 (0.5%)
Don't know	281 (2.4%)	44 (4.4%)	82 (8.2%)	2 (0.2%)	14 (1.4%)	6 (0.6%)	9 (0.9%)	5 (0.5%)	47 (4.9%)	43 (4.3%)	7 (0.7%)	20 (2.0%)	3 (0.3%)

[1]n (%)

Table 38: Demographic characteristics of internet-using children aged 12–17 who experienced one or more forms of technology facilitated CSEA (N = 11,912). Counts and proportions are survey-weighted to reflect the national population of internet-using children in each country.

Characteristic	Overall N = 2,025[1]	Cambodia N = 145[1]	Ethiopia N = 186[1]	Indonesia N = 66[1]	Kenya N = 220[1]	Malaysia N = 102[1]	Mozambique N = 258[1]	Namibia N = 198[1]	Philippines N = 271[1]	Tanzania N = 77[1]	Thailand N = 164[1]	Uganda N = 281[1]	Vietnam N = 54[1]
**Child exposed to technology facilitated CSEA**													
1	2,025 (100%)	145 (100%)	186 (100%)	66 (100%)	220 (100%)	102 (100%)	258 (100%)	198 (100%)	271 (100%)	77 (100%)	164 (100%)	281 (100%)	54 (100%)
**Sex**													
Boy	1,026 (51%)	85 (59%)	109 (59%)	38 (58%)	109 (50%)	51 (50%)	127 (49%)	83 (42%)	122 (45%)	49 (64%)	85 (52%)	146 (52%)	20 (36%)
Girl	999 (49%)	60 (41%)	77 (41%)	28 (42%)	111 (50%)	51 (50%)	132 (51%)	115 (58%)	150 (55%)	28 (36%)	79 (48%)	135 (48%)	35 (64%)
**Age**													
12	161 (8.0%)	24 (17%)	1 (0.7%)	8 (12%)	17 (7.5%)	14 (14%)	17 (6.6%)	14 (7.0%)	34 (13%)	3 (4.1%)	17 (10%)	9 (3.1%)	3 (5.3%)
13	190 (9.4%)	19 (13%)	6 (3.0%)	2 (2.3%)	21 (9.5%)	15 (15%)	36 (14%)	15 (7.7%)	33 (12%)	3 (4.5%)	26 (16%)	9 (3.3%)	4 (8.0%)
14	269 (13%)	29 (20%)	14 (7.5%)	12 (19%)	31 (14%)	12 (12%)	30 (12%)	29 (15%)	33 (12%)	16 (21%)	29 (18%)	20 (7.1%)	13 (23%)
15	365 (18%)	28 (19%)	25 (13%)	12 (18%)	38 (17%)	17 (17%)	39 (15%)	39 (20%)	56 (20%)	17 (22%)	28 (17%)	56 (20%)	11 (20%)
16	480 (24%)	24 (17%)	45 (24%)	16 (24%)	56 (25%)	28 (27%)	71 (27%)	48 (24%)	54 (20%)	13 (17%)	35 (21%)	80 (28%)	11 (20%)
17	558 (28%)	21 (14%)	95 (51%)	16 (25%)	58 (26%)	15 (15%)	65 (25%)	53 (27%)	61 (23%)	24 (31%)	29 (18%)	108 (38%)	13 (24%)
**Areas**													
Peri urban	213 (11%)	23 (16%)	29 (16%)	13 (20%)	5 (2.2%)	20 (20%)	34 (13%)	12 (6.2%)	22 (8.3%)	2 (2.5%)	34 (20%)	15 (5.2%)	4 (8.2%)
Rural	1,049 (52%)	88 (60%)	95 (51%)	28 (42%)	140 (64%)	35 (34%)	114 (44%)	102 (51%)	133 (49%)	39 (51%)	55 (34%)	188 (67%)	33 (61%)
Urban	763 (38%)	34 (24%)	62 (33%)	26 (39%)	75 (34%)	47 (46%)	110 (43%)	84 (43%)	115 (43%)	36 (47%)	76 (46%)	79 (28%)	17 (31%)

Table 39: Survey-weighted prevalence of technology-facilitated CSEA by demographic subgroup among internet-using children aged 12–17 (N = 11,912). For each subgroup, unweighted counts, weighted estimates of affected children, and survey-weighted prevalence with 95% confidence intervals are reported. Prevalence reflects the proportion of internet-using children who experienced one or more forms of technology-facilitated CSEA through social media or gaming platforms in 2020–2021

**Demographics**	**N (unweighted)**	**Affected Children (unweighted)**	**Affected Children (weighted)**	**Prevalence % (95%% CI)**
12	1,474	151	161	11.4 (9.5–13.3)
13	1,569	165	190	11.2 (9.4–13.0)
14	1,768	244	269	14.2 (12.4–16.1)
15	1,968	338	365	17.7 (15.8–19.7)
16	2,130	444	480	20.8 (18.9–22.7)
17	3,003	725	558	22.0 (20.4–23.7)
Boy	6,132	1085	1026	17.0 (15.9–18.0)
Girl	5,780	982	999	17.0 (15.9–18.1)
Peri urban	1,153	209	213	18.1 (15.5–20.7)
Rural	6,787	1,120	1049	15.9 (15.0–16.9)
Urban	3,972	738	763	18.4 (17.0–19.8)

Table 40: Survey-weighted disclosure rates by channel among children who experienced one or more forms of technology-facilitated CSEA (weighted N = 2,025). For each channel, the overall row pools estimates across all 12 countries; country-specific rows show national estimates. Estimates reflect the proportion of children who disclosed to each channel; percentages are non-additive as children could select multiple channels. Confidence intervals calculated using survey-weighted means (95% CI).

**Country**	**N(unweighted)**	**N Total Sample (unweighted)**	**N (weighted)**	**N Total Sample (weighted)**	**Prevalence (%)**	**Lower CI**	**Upper CI**
			**Did not disclose**				
**Overall**	**1,080**	**2,067**	**1,034**	**2,025**	**51.1**	**48.6**	**53.6**
Cambodia	74	137	78	145	53.8	44.8	62.8
Ethiopia	101	195	96	186	51.5	42.9	60.0
Indonesia	35	66	36	66	53.8	40.8	66.9
Kenya	111	230	102	220	46.3	39.3	53.2
Malaysia	62	109	56	102	54.9	44.0	65.8
Mozambique	108	231	117	258	45.2	37.7	52.6
Namibia	139	225	117	198	58.8	51.0	66.5
Philippines	137	269	138	271	50.7	43.9	57.5
Tanzania	47	88	38	77	48.9	37.3	60.4
Thailand	93	157	97	164	59.1	50.8	67.5
Uganda	148	311	130	281	46.3	39.9	52.8

Vietnam	25	49	31	54	57.3	40.9	73.6
**Father**							
**Overall**	**384**	**2,067**	**403**	**2,025**	**19.9**	**17.9**	**21.9**
Cambodia	51	137	57	145	39.2	30.3	48.1
Ethiopia	33	195	37	186	19.8	12.7	27.0
Indonesia	8	66	6	66	8.6	2.2	15.0
Kenya	38	230	38	220	17.3	12.0	22.6
Malaysia	20	109	22	102	21.2	12.0	30.5
Mozambique	39	231	53	258	20.4	13.8	26.9
Namibia	44	225	41	198	20.6	14.5	26.8
Philippines	37	269	42	271	15.6	10.4	20.8
Tanzania	16	88	15	77	19.7	10.6	28.8
Thailand	51	157	51	164	31.0	23.3	38.8
Uganda	45	311	41	281	14.5	10.0	19.0
Vietnam	2	49	1	54	1.9	-0.8	4.7
**Friend**							
**Overall**	**956**	**2,067**	**923**	**2,025**	**45.6**	**43.1**	**48.1**
Cambodia	53	137	51	145	35.1	26.8	43.5
Ethiopia	94	195	84	186	45.0	36.5	53.4

Indonesia	29	66	30	66	45.1	32.1	58.2
Kenya	107	230	106	220	48.3	41.3	55.3
Malaysia	44	109	39	102	38.7	28.3	49.1
Mozambique	112	231	124	258	48.2	40.7	55.7
Namibia	106	225	93	198	46.8	39.1	54.6
Philippines	126	269	126	271	46.3	39.5	53.1
Tanzania	40	88	37	77	48.2	36.7	59.8
Thailand	70	157	75	164	45.5	37.0	54.1
Uganda	160	311	142	281	50.4	43.9	56.9
Vietnam	15	49	16	54	29.7	14.7	44.7
				**Helpline**			
**Overall**	**63**	**2,067**	**67**	**2,025**	**3.3**	**2.4**	**4.2**
Cambodia	7	137	8	145	5.4	1.0	9.9
Ethiopia	14	195	10	186	5.2	1.9	8.4
Indonesia	0	66	0	66	0.0	0.0	0.0
Kenya	8	230	10	220	4.7	1.4	8.1
Malaysia	2	109	2	102	1.8	-0.7	4.3
Mozambique	3	231	4	258	1.7	-0.6	4.0
Namibia	8	225	7	198	3.6	0.9	6.4

Philippines	3	269	3	271	1.2	-0.2	2.5
Tanzania	1	88	2	77	2.6	-2.4	7.6
Thailand	4	157	4	164	2.7	0.0	5.5
Uganda	11	311	13	281	4.6	1.3	7.9
Vietnam	2	49	3	54	5.2	-3.3	13.6
				**Mother**			
**Overall**	**420**	**2,067**	**432**	**2,025**	**21.3**	**19.3**	**23.4**
Cambodia	33	137	35	145	23.8	16.2	31.4
Ethiopia	26	195	27	186	14.3	8.1	20.5
Indonesia	7	66	7	66	10.8	3.0	18.5
Kenya	34	230	34	220	15.4	10.2	20.6
Malaysia	25	109	22	102	21.7	12.8	30.5
Mozambique	43	231	52	258	20.3	14.1	26.5
Namibia	49	225	47	198	23.7	17.0	30.3
Philippines	62	269	70	271	25.8	19.6	31.9
Tanzania	15	88	14	77	17.9	9.3	26.6
Thailand	64	157	64	164	39.0	30.7	47.2
Uganda	52	311	51	281	18.0	12.9	23.0
Vietnam	10	49	10	54	18.3	5.9	30.8

			**Other**				
**Overall**	**19**	**2,067**	**22**	**2,025**	**1.1**	**0.6**	**1.6**
Cambodia	1	137	2	145	1.5	-1.4	4.3
Ethiopia	1	195	1	186	0.5	-0.5	1.6
Indonesia	0	66	0	66	0.0	0.0	0.0
Kenya	1	230	0	220	0.2	-0.1	0.4
Malaysia	0	109	0	102	0.0	0.0	0.0
Mozambique	8	231	9	258	3.4	0.9	5.9
Namibia	1	225	2	198	0.9	-0.8	2.6
Philippines	5	269	5	271	2.0	0.2	3.7
Tanzania	0	88	0	77	0.0	0.0	0.0
Thailand	0	157	0	164	0.0	0.0	0.0
Uganda	2	311	2	281	0.8	-0.3	2.0
Vietnam	0	49	0	54	0.0	0.0	0.0
			**Other Adults**				
**Overall**	**130**	**2,067**	**127**	**2,025**	**6.3**	**5.1**	**7.5**
Cambodia	12	137	13	145	8.9	3.9	13.9
Ethiopia	10	195	9	186	4.7	1.3	8.0
Indonesia	2	66	2	66	2.7	-1.0	6.4

Kenya	16	230	14	220	6.3	3.0	9.6
Malaysia	4	109	4	102	4.4	-0.2	8.9
Mozambique	7	231	10	258	3.8	0.6	7.1
Namibia	25	225	21	198	10.7	6.2	15.2
Philippines	17	269	17	271	6.2	2.9	9.5
Tanzania	4	88	5	77	6.2	0.3	12.2
Thailand	8	157	8	164	4.7	1.3	8.2
Uganda	22	311	22	281	7.8	4.0	11.6
Vietnam	3	49	3	54	5.8	-2.8	14.3
				**Police**			
**Overall**	**59**	**2,067**	**66**	**2,025**	**3.3**	**2.3**	**4.2**
Cambodia	7	137	7	145	4.9	1.1	8.7
Ethiopia	11	195	7	186	4.0	1.5	6.4
Indonesia	1	66	0	66	0.4	-0.4	1.1
Kenya	5	230	7	220	3.2	0.3	6.1
Malaysia	2	109	3	102	2.5	-1.0	6.1
Mozambique	7	231	11	258	4.2	0.9	7.4
Namibia	3	225	3	198	1.3	-0.4	3.1
Philippines	7	269	11	271	4.2	0.8	7.6

Tanzania	2	88	2	77	2.8	-1.0	6.6
Thailand	5	157	5	164	3.2	0.2	6.3
Uganda	9	311	10	281	3.5	0.5	6.4
Vietnam	0	49	0	54	0.0	0.0	0.0
			**Sibling**				
**Overall**	**516**	**2,067**	**523**	**2,025**	**25.8**	**23.6**	**28.0**
Cambodia	43	137	47	145	32.0	23.6	40.5
Ethiopia	46	195	42	186	22.8	15.7	29.8
Indonesia	7	66	8	66	12.5	3.3	21.7
Kenya	40	230	39	220	17.5	12.3	22.8
Malaysia	25	109	23	102	22.1	13.4	30.9
Mozambique	61	231	80	258	30.8	23.6	38.1
Namibia	77	225	73	198	36.6	29.0	44.1
Philippines	48	269	53	271	19.6	14.0	25.1
Tanzania	19	88	17	77	22.7	13.2	32.2
Thailand	54	157	55	164	33.6	25.7	41.6
Uganda	90	311	78	281	27.7	22.1	33.4
Vietnam	6	49	8	54	15.0	2.8	27.2
			**Social Worker**				

**Overall**	**58**	**2,067**	**59**	**2,025**	**2.9**	**2.0**	**3.8**
Cambodia	8	137	7	145	5.1	1.6	8.6
Ethiopia	8	195	7	186	3.8	0.7	6.9
Indonesia	1	66	0	66	0.4	-0.4	1.1
Kenya	3	230	3	220	1.4	-0.2	3.0
Malaysia	1	109	1	102	0.9	-0.9	2.7
Mozambique	3	231	5	258	1.7	-0.6	4.1
Namibia	11	225	11	198	5.6	1.9	9.3
Philippines	6	269	7	271	2.4	0.2	4.6
Tanzania	1	88	1	77	1.4	-1.3	4.1
Thailand	2	157	3	164	1.6	-0.6	3.7
Uganda	13	311	12	281	4.2	1.5	6.9
Vietnam	1	49	2	54	4.4	-4.0	12.8
				**Teacher**			
**Overall**	**179**	**2,067**	**177**	**2,025**	**8.7**	**7.3**	**10.1**
Cambodia	21	137	21	145	14.7	8.4	21.0
Ethiopia	27	195	21	186	11.2	6.1	16.3
Indonesia	1	66	0	66	0.4	-0.4	1.1
Kenya	22	230	23	220	10.4	6.0	14.9

Malaysia	8	109	6	102	5.9	1.7	10.1
Mozambique	8	231	9	258	3.6	1.0	6.3
Namibia	21	225	20	198	10.0	5.3	14.8
Philippines	12	269	12	271	4.5	1.8	7.1
Tanzania	8	88	7	77	9.2	2.4	15.9
Thailand	12	157	12	164	7.1	3.0	11.3
Uganda	37	311	42	281	15.0	9.8	20.2
Vietnam	2	49	3	54	5.1	-3.3	13.6

Table 41: Survey-weighted percentages of children who experienced technology-facilitated CSEA and disclosed to each channel, broken down by CSEA type (2020–2021). The denominator for each cell is all children who experienced that specific CSEA type. Percentages are non-additive as children could disclose to multiple channels.

CSEA Type	Father	Friend	Helpline	Mother	Other	Other Adults	Police	Sibling	Social Worker	Teacher
Sexual comments	9%	30%	1%	11%	1%	3%	1%	12%	1%	4%
Sexual images	10%	32%	1%	10%	0%	2%	1%	14%	1%	4%
Talk about sex	10%	29%	1%	11%	1%	2%	2%	15%	1%	5%
Do something sexual	9%	31%	1%	12%	0%	3%	1%	16%	1%	5%
Image/video: private parts	12%	29%	1%	13%	0%	2%	2%	16%	1%	3%
Money for sexual images/videos	14%	31%	1%	16%	0%	2%	2%	17%	1%	4%
Money to meet to do something sexual	13%	31%	1%	15%	0%	5%	2%	18%	1%	4%
Shared sexual images without consent	10%	30%	2%	13%	0%	3%	2%	17%	2%	4%
Blackmailed for sexual activities	13%	32%	1%	15%	0%	5%	2%	19%	1%	5%

Table 42: Full survey response descriptions and corresponding short labels for perpetrator identity categories used in the analysis of technology-facilitated CSEA (2020–2021). Full descriptions reflect original survey wording as presented to child respondents aged 12–17; short labels are analytic abbreviations used in figures and tables throughout the supplementary materials.

Full Response Description	Short Label
1. Someone in my family	Family member
2. A romantic partner (or ex-)	Romantic partner or ex
3. A friend or someone I knew who is 18 years or older	Known other (18 or older)
4. A friend or someone I knew who is younger than 18	Known other (under 18)
5. Someone I didn't know before this happened	Unknown person
6. Someone else (please specify)	Other known/unknown person
7. I don't know who the person was	Identity not known
8. Prefer not to say	Prefer not to say

Table 43: Survey-weighted percentages of children reporting each perpetrator type, broken down by form of technology-facilitated CSEA (2020–2021). The denominator for each cell is all children who experienced that specific CSEA type. Percentages are non-additive as children could report multiple perpetrator types. Perpetrator categories reflect original survey response options capturing the relationship between the child and the perpetrator.

CSEA type	Known other (18 or older)	Known other (under 18)	Unknown person	Family member	Identity not known	Romantic partner or ex	Other known/unknown person
Blackmailed for sexual activities	26.2%	22%	13%	15%	13.4%	13%	0.9%
Money to meet to do something sexual	25.8%	20%	12.4%	19.9%	11.7%	16.1%	0.6%
Talk about sex	24%	19.1%	15.2%	13.1%	13.6%	13.8%	0.8%
Sexual comments	23.4%	18.6%	15%	14.5%	13.3%	15.6%	1.5%
Image/video: private parts	23.3%	16.2%	17.6%	16.2%	14.1%	16.6%	0.4%
Money for sexual images/videos	22.4%	22.7%	14.1%	15.5%	13.6%	16.4%	0.4%
Do something sexual	22.6%	21.8%	15.8%	13.9%	10.9%	16.2%	0.5%
Sexual images	19%	19.4%	21.4%	10.4%	18.5%	12.2%	1.2%
Shared sexual images without consent	20.7%	20.4%	16.6%	14.5%	15.5%	13%	0.1%

Table 44: Survey-weighted frequencies of perpetrator type combination patterns reported by children who experienced technology-facilitated CSEA across 12 countries in Africa and Asia (2020–2021). Each row represents a unique co-occurrence pattern of perpetrator types, encoded as a binary string indicating which perpetrator categories were selected. Weighted frequencies reflect population-level estimates of how commonly each pattern occurs. Single-perpetrator-type patterns (e.g., unknown person only) are distinguished from mixed patterns where children reported multiple perpetrator types.

Pattern	Weighted Frequency	Types
1.0.0.0	546	Unknown only
0.0.0.1	369	Other only
1.0.0.1	227	Unknown + Other
0.1.0.1	116	Romantic + Other
1.1.0.1	105	Unknown + Romantic + Other
0.1.0.0	93	Romantic only
0.0.1.0	90	Family only
0.0.1.1	83	Family + Other
0.1.1.1	80	Romantic + Family + Other
1.0.1.1	58	Unknown + Family + Other
1.1.1.1	55	All four types
1.1.0.0	41	Unknown + Romantic
1.0.1.0	39	Unknown + Family
0.1.1.0	32	Romantic + Family
1.1.1.0	16	Unknown + Romantic + Family
0.0.0.0	0	Other combination

Table 45: Full survey response descriptions and corresponding short labels for barriers to disclosure used in the analysis of technology-facilitated CSEA (2020–2021). Full descriptions reflect original survey wording as presented to children aged 12–17 who did not disclose their experience; short labels are analytic abbreviations used in figures and tables throughout the supplementary materials.

	**Full Response Description**	**Short Label**
**1**	I did not know where to go or who to tell	Did not know who to tell
**2**	I felt embarrassed, ashamed or that it would be too emotionally difficult to tell	Embarrassed and ashamed
**3**	I did not think anyone would believe me or understand my situation	No one would believe
**4**	I was worried I would get in trouble if I told someone	In trouble
**5**	I felt that I did something wrong and did not want to tell	Did something wrong
**6**	I did not think it was serious enough to report	Not serious enough
**7**	I did not want the person who did this to get into trouble	Perpetrator into trouble
**8**	I feared it would cause trouble for me or my family	Family into trouble
**9**	I did not think anything would be done	No action
**10**	I did not know you could report these things	Didn't know they could report
**11**	My friends discouraged me from reporting	Friends discouraged
**12**	My parents discouraged me from reporting	Parents discouraged
**13**	I feared it would not be kept confidential	Not confidential

Note on Reliability Classification

All prevalence estimates in Table 46 were calculated using survey weights and Wilson score confidence intervals. Wilson intervals were preferred over Wald intervals because they remain within the [0, 100] range and provide more accurate uncertainty estimates when sample sizes are small or proportions are near 0% or 100%, conditions that arise when stratifying barriers by CSEA type. Each estimate was classified by reliability against three criteria: unweighted sample size ≥10 observations, ≥ 3 positive responses, and confidence interval width ≤20 percentage points. Estimates meeting all three criteria are shown without annotation and can be considered reliable. Estimates failing the sample size or event threshold are marked † and shown without confidence intervals, as data are insufficient for stable estimation. Estimates meeting minimum sample requirements but with confidence interval width >20 percentage points are marked ‡, indicating substantial uncertainty due to data sparsity. These annotations allow readers to distinguish well-estimated parameters from those where sparsity introduces meaningful uncertainty.

Table 46: Survey-weighted percentages of children reporting each barrier to disclosure, broken down by type of technology-facilitated CSEA (2020–2021). Values are presented as point estimate [lower CI, upper CI] in percentage points. The denominator for each cell is all children who experienced that specific CSEA type and did not disclose. Confidence intervals calculated using the Wilson score method. Estimates marked † are based on insufficient sample sizes (unweighted n < 10 or fewer than 3 positive responses) and are shown without confidence intervals. Estimates marked ‡ meet minimum sample requirements but have confidence interval width > 20 percentage points, indicating substantial uncertainty. Barriers capture a range of reasons children did not disclose their experience.

Barrier to Disclosure	Overall	Blackmailed for sexual activities	Do something sexual	Image/video: private parts	Money for sexual images/videos	Money to meet to do something sexual	Sexual comments	Sexual images	Shared sexual images without consent	Talk about sex
Did not know who to tell	37.6 [35.6,39.6]	44.9[35.8,54.3]	40.2[34.3,46.4]	39.9[33.3,47.0]	42.8[34.4,51.6]	36.0[28.2,44.6]	37.0[32.9,41.2]	30.7[26.9,34.8]	48.1[39.8,56.5]	37.9[32.5,43.6]
Embarrassed and ashamed	19.6 [18.0,21.3]	17.6[11.6,25.9]	23.6[18.8,29.3]	24.2[18.7,30.6]	24.6[17.8,32.8]	17.5[11.9,25.1]	17.7[14.7,21.2]	18.9[15.8,22.5]	18.4[12.8,25.9]	17.8[13.8,22.6]
Not serious enough	14.2 [12.9,15.7]	5.9[2.8,12.0]	9.9[6.8,14.2]	13.9[9.7,19.4]	12.1[7.4,19.0]	9.9[5.8,16.3]	17.3[14.3,20.8]	18.5[15.4,22.0]	7.7[4.3,13.5]	14.1[10.6,18.6]
In trouble	10.0 [8.8,11.3]	12.7[7.6,20.3]	14.4[10.6,19.3]	11.8[8.0,17.0]	11.4[6.9,18.3]	14.6[9.5,21.8]	7.5[5.5,10.1]	6.9[5.1,9.5]	11.4[7.0,17.9]	10.8[7.7,14.9]
No one would believe	8.5 [7.4,9.7]	11.5[6.8,18.9]	11.6[8.2,16.1]	8.4[5.2,13.1]	9.2[5.3,15.6]	12.3[7.7,19.1]	8.8[6.7,11.5]	5.0[3.4,7.2]	8.0[4.5,13.9]	8.9[6.1,12.7]
Did something wrong	6.5 [5.6,7.6]	3.0[1.0,8.1]	6.4[4.0,10.2]	9.7[6.3,14.7]	8.4[4.7,14.6]	8.0[4.4,14.0]	5.3[3.7,7.6]	5.7[4.0,8.0]	8.8[5.1,14.8]	6.8[4.4,10.3]
No action	5.0 [4.2,6.0]	1.4†	2.7[1.3,5.6]	4.9[2.6,8.9]	2.7[1.0,7.3]	2.8[1.0,7.3]	7.1[5.2,9.6]	7.3[5.4,9.9]	2.6[1.0,6.9]	3.7[2.1,6.5]
Perpetrator into trouble	4.2 [3.4,5.1]	1.1†	4.6[2.6,8.0]	3.8[1.9,7.5]	4.4[1.9,9.6]	5.8[2.9,11.3]	4.7[3.2,6.8]	4.5[3.0,6.7]	1.4[0.4,5.1]	4.3[2.5,7.3]
Family into trouble	4.0 [3.2,4.9]	1.7[0.4,6.3]	3.5[1.8,6.6]	5.9[3.4,10.1]	3.8[1.6,8.7]	2.8[1.1,7.4]	3.9[2.6,5.9]	3.9[2.6,6.0]	8.0[4.5,13.8]	3.0[1.6,5.7]
Didn't know they could report	3.4 [2.8,4.3]	3.9[1.6,9.4]	1.3[0.5,3.6]	2.8[1.3,6.2]	2.6[0.9,7.2]	3.0[1.2,7.6]	4.3[2.8,6.3]	4.8[3.2,6.9]	1.6[0.4,5.4]	3.1[1.6,5.8]
Not confidential	2.5 [2.0,3.2]	3.0[1.1,8.2]	2.4[1.1,5.1]	1.7[0.6,4.7]	1.2[0.3,5.1]	3.4[1.4,8.2]	2.9[1.8,4.8]	2.6[1.5,4.3]	2.6[1.0,6.9]	2.2[1.1,4.7]
Friends discouraged	1.0 [0.7,1.5]	0.0†	1.1[0.4,3.3]	0.5†	0.8†	0.0†	1.2[0.6,2.5]	1.2[0.6,2.6]	0.4†	1.9[0.8,4.2]
Parents discouraged	0.5 [0.2,0.8]	0.0†	0.3†	0.5†	0.7†	0.5†	0.9[0.4,2.1]	0.1†	1.1†	0.0†

Table 47: Survey-weighted percentages of children who experienced each form of CSEA in online versus offline contexts (2020–2021). The denominator for each cell is all children who experienced that specific CSEA type. Percentages are non-additive as children could report experiences in multiple contexts. Context categories reflect original survey response options distinguishing technology-mediated from in-person settings.

CSEA Type	On Social media	In person (face-to-face)	In an online game	Don't know	Some other way	Prefer not to say
Sexual images	52.3%	16.5%	11.6%	12.1%	11.2%	7.1%
Image/video: private parts	47.5%	22%	14.2%	10.6%	8.6%	10.3%
Talk about sex	41.9%	31.7%	11.7%	10.2%	11.4%	8.2%
Shared sexual images without consent	41.4%	18.6%	17.4%	14.9%	13.9%	9.9%
Money to meet to do something sexual	39.4%	26.5%	16.9%	13.1%	12%	10.3%
Blackmailed for sexual activities	38.5%	24.5%	16.2%	14.6%	13.7%	9.4%
Money for sexual images/videos	38.4%	24.2%	17.2%	13.4%	13.3%	7.6%
Sexual comments	38.2%	32%	11.4%	12.9%	9%	8%
Do something sexual	35.3%	31.4%	13.3%	12.4%	12.4%	8.8%

Table 48: Survey-weighted percentages of children reporting technology-facilitated CSEA occurring on specific social media platforms (2020–2021). The denominator is children who reported experiencing CSEA via social media and completed the platform follow-up question; children who did not answer the follow-up question are excluded. Percentages are non-additive as children could report experiences across multiple platforms. Platform categories reflect original survey response options.

Platform	Blackmailed for sexual activities	Do something sexual	Image/video: private parts	Money for sexual images/videos	Money to meet to do something sexual	Sexual comments	Sexual images	Shared sexual images without consent	Talk about sex
Discord	1.2%	3.9%	4.8%	2.3%	2.2%	4.5%	4.3%	0.8%	5.6%
Don't know	0%	0.2%	0.5%	0.2%	0.7%	0.8%	0.5%	0%	0.3%
Facebook or Messenger	72.3%	69%	70.9%	70.3%	70.1%	69.9%	70.1%	66.2%	69.7%
Flickr	1%	0.9%	0.5%	0%	0.5%	0.9%	0.4%	0.7%	0.8%
Instagram	21.4%	17.8%	17.9%	18.7%	21.1%	14%	11.6%	17.1%	14.1%
Line	3.3%	4.2%	2%	3.9%	4.9%	5.6%	3.5%	3.2%	4.9%
Live.me	1.1%	1.1%	1.2%	0.6%	1.2%	1.2%	1.1%	0.3%	0.7%
Other	0.8%	2.3%	1%	3.7%	1.3%	1.3%	1.4%	0.2%	0.9%
Periscope	0.9%	0.8%	0.7%	0%	0.9%	0.6%	0.3%	0.9%	0.4%

Platform	Blackmailed for sexual activities	Do something sexual	Image/video: private parts	Money for sexual images/videos	Money to meet to do something sexual	Sexual comments	Sexual images	Shared sexual images without consent	Talk about sex
Prefer not to say	0%	1.3%	0.9%	1.6%	0.6%	1.5%	1.2%	0.7%	1.3%
Snapchat	8%	8.1%	6.7%	9.2%	11.3%	7.1%	3.8%	9.5%	7.6%
TikTok	21.1%	14%	10.9%	15.7%	18.1%	13.3%	7.3%	17.3%	11.7%
Tumblr	0.8%	2.4%	0.7%	0.3%	0.5%	1%	1%	1%	0.7%
Twitch	3.6%	3.1%	3.9%	3.2%	6.6%	3%	2%	6.2%	3.9%
Twitter	19.6%	16%	13.4%	18.4%	23%	9.8%	7.6%	20.2%	14.8%
WhatsApp	23.1%	27.2%	28.3%	26.8%	21.4%	26.6%	28.6%	25.7%	23.5%
YouTube	13.2%	11%	8.1%	10.2%	11.4%	10.4%	8.7%	11.3%	11.2%

Table 49: Survey-weighted prevalence of each form of CSEA among internet-using children aged 12–17, pooled across all 12 countries (2020–2021). Values are presented as point estimate (%) and 95% confidence interval. Denominator is all children who completed the relevant survey item. Estimates reflect experiences occurring on online or offline contexts. CSEA types are ordered by overall prevalence.

**Type of CSEA**	**n/N Weighted (unweighted)**	**Prevalence (95% CI)**
Sexual comments	1,908/11,396 (1952/11410)	16.7% [16.0 - 17.5]
Sexual images	1,902/11,447 (1940/11439)	16.6% [15.8 - 17.4]
Talk about sex	1,160/11,359 (1200/11373)	10.2% [9.6 - 10.8]
Do something sexual	1,060/11,395 (1078/11389)	9.3% [8.7 - 9.9]
Image/video: private parts	885/11,413 (878/11413)	7.8% [7.2 - 8.3]
Money for sexual images/videos	671/11,470 (694/11473)	5.8% [5.4 - 6.3]
Money to meet to do something sexual	653/11,472 (681/11471)	5.7% [5.2 - 6.2]
Shared sexual images without consent	646/11,386 (671/11406)	5.7% [5.2 - 6.1]
Blackmailed for sexual activities	621/11,435 (636/11441)	5.4% [5.0 - 5.9]

Table 50: Survey-weighted prevalence of each form of technology-facilitated CSEA among internet-using children aged 12–17, pooled across all 12 countries (2020–2021). Values are presented as point estimate (%) and 95% confidence interval. Denominator is all children who completed the relevant survey item. Estimates reflect experiences occurring through social media or online gaming platforms. CSEA types are ordered by overall prevalence.

Type of CSEA	n/N Weighted (unweighted)	Prevalence (95% CI)
Experienced any form of technology-facilitated CSEA	2,025/11,912 (2067/11912)	17.0% [16.2 - 17.8]
Sent unwanted sexual images	1,143/11,912 (1176/11912)	9.6% [9.0 - 10.2]
Unwanted sexual comments	889/11,912 (901/11912)	7.5% [6.9 - 8.0]
Asked to talk about sex/acts	571/11,912 (581/11912)	4.8% [4.4 - 5.2]
Asked for intimate photos/videos	498/11,912 (499/11912)	4.2% [3.8 - 4.6]
Asked to do something sexual	462/11,912 (472/11912)	3.9% [3.5 - 4.3]
Shared sexual images without consent	332/11,912 (338/11912)	2.8% [2.5 - 3.1]
Offered money/gifts for in-person acts	328/11,912 (331/11912)	2.8% [2.4 - 3.1]
Offered money/gifts for images	324/11,912 (335/11912)	2.7% [2.4 - 3.1]
Threatened/blackmailed for sex	294/11,912 (288/11912)	2.5% [2.1 - 2.8]

Table 51: Survey-weighted prevalence of any CSEA (online and offline) among internet-using children aged 12–17, by country (2020–2021). Includes all nine CSEA types regardless of whether the incident occurred online or in person. Values show unweighted and weighted counts of affected children over total sample, with survey-weighted prevalence and 95% confidence intervals. Countries ordered by prevalence.

**Country**	**n/N (weighted)**	**Prevalence (%)**	**95% CI**
Uganda	514/983 (549/982)	52	52.3% [48.7 - 55.9]
Philippines	458/877 (449/885)	52	52.2% [48.4 - 55.9]
Mozambique	435/938 (423/939)	46	46.4% [42.6 - 50.1]
Ethiopia	355/885 (374/892)	40	40.2% [36.3 - 44.0]
Kenya	370/975 (379/975)	38	37.9% [34.7 - 41.2]
Cambodia	287/877 (288/868)	33	32.7% [29.3 - 36.0]
Namibia	273/968 (303/963)	28	28.2% [24.9 - 31.5]
Thailand	245/929 (240/928)	26	26.4% [23.3 - 29.5]
Malaysia	151/945 (158/948)	16	16.0% [13.3 - 18.7]
Tanzania	144/928 (159/928)	16	15.5% [13.0 - 18.0]
Indonesia	122/934 (125/928)	13	13.1% [10.8 - 15.5]
Vietnam	123/944 (117/947)	13	13.0% [10.4 - 15.6]

Table 52: Overall prevalence of technology-facilitated CSEA outcomes by country (survey-weighted estimates with 95% confidence intervals; complete-case denominators). This table reports the estimated prevalence of each technology-facilitated CSEA harm across all 12 study countries. Prevalence estimates are survey-weighted to reflect the national child population. Denominators represent the number of respondents with complete data for each outcome. The final column provides the weighted estimated number of children who experienced each form of technology-facilitated CSEA.

Country	Type of Abuse	Prevalence (95% CI)	Denominator	Children Affected
**Cambodia**	**Any online CSEA**	**14.7% (12.2–17.1)**	**992 (weighted 11,912)**	**137 (weighted 145)**
Cambodia	Sent unwanted sexual images	7.4% (5.6–9.2)	992 (weighted 11,912)	69 (weighted 74)
Cambodia	Unwanted sexual comments	7.0% (5.2–8.8)	992 (weighted 11,912)	62 (weighted 69)
Cambodia	Asked to do something sexual	4.4% (3.0–5.8)	992 (weighted 11,912)	40 (weighted 44)
Cambodia	Asked for intimate photos/videos	3.9% (2.6–5.2)	992 (weighted 11,912)	35 (weighted 38)
Cambodia	Asked to talk about sex/acts	3.1% (1.9–4.3)	992 (weighted 11,912)	28 (weighted 31)
Cambodia	Offered money/gifts for in-person acts	3.0% (1.9–4.2)	992 (weighted 11,912)	28 (weighted 30)
Cambodia	Shared sexual images without consent	2.8% (1.6–3.9)	992 (weighted 11,912)	26 (weighted 27)
Cambodia	Threatened/blackmailed for sex	2.1% (1.2–3.1)	992 (weighted 11,912)	20 (weighted 21)
Cambodia	Offered money/gifts for images	1.9% (1.1–2.8)	992 (weighted 11,912)	20 (weighted 19)
**Ethiopia**	**Any online CSEA**	**18.6% (15.7–21.5)**	**1,000 (weighted 11,912)**	**195 (weighted 186)**
Ethiopia	Sent unwanted sexual images	8.7% (6.8–10.7)	1,000 (weighted 11,912)	98 (weighted 87)
Ethiopia	Unwanted sexual comments	7.8% (5.8–9.7)	1,000 (weighted 11,912)	80 (weighted 78)
Ethiopia	Asked to talk about sex/acts	5.9% (4.1–7.7)	1,000 (weighted 11,912)	59 (weighted 59)
Ethiopia	Asked to do something sexual	4.2% (2.6–5.7)	1,000 (weighted 11,912)	41 (weighted 42)
Ethiopia	Asked for intimate photos/videos	3.7% (2.3–5.2)	1,000 (weighted 11,912)	36 (weighted 37)
Ethiopia	Offered money/gifts for images	2.8% (1.7–3.9)	1,000 (weighted 11,912)	35 (weighted 28)
Ethiopia	Offered money/gifts for in-person acts	2.5% (1.4–3.6)	1,000 (weighted 11,912)	29 (weighted 25)
Ethiopia	Shared sexual images without consent	2.1% (1.2–3.0)	1,000 (weighted 11,912)	28 (weighted 21)
Ethiopia	Threatened/blackmailed for sex	2.0% (1.1–2.9)	1,000 (weighted 11,912)	24 (weighted 20)
**Indonesia**	**Any online CSEA**	**6.6% (5.0–8.3)**	**995 (weighted 11,912)**	**66 (weighted 66)**
Indonesia	Sent unwanted sexual images	3.8% (2.5–5.1)	995 (weighted 11,912)	37 (weighted 38)

Country	Type of Abuse	Prevalence (95% CI)	Denominator	Children Affected
Indonesia	Unwanted sexual comments	2.5% (1.5–3.5)	995 (weighted 11,912)	27 (weighted 25)
Indonesia	Asked to talk about sex/acts	1.1% (0.4–1.8)	995 (weighted 11,912)	12 (weighted 11)
Indonesia	Offered money/gifts for in-person acts	0.6% (0.1–1.1)	995 (weighted 11,912)	7 (weighted 6)
Indonesia	Asked to do something sexual	0.5% (-0.0–1.0)	995 (weighted 11,912)	4 (weighted 5)
Indonesia	Threatened/blackmailed for sex	0.4% (-0.0–0.9)	995 (weighted 11,912)	4 (weighted 4)
Indonesia	Asked for intimate photos/videos	0.4% (0.0–0.8)	995 (weighted 11,912)	6 (weighted 4)
Indonesia	Shared sexual images without consent	0.3% (-0.1–0.7)	995 (weighted 11,912)	3 (weighted 3)
Indonesia	Offered money/gifts for images	0.2% (-0.1–0.5)	995 (weighted 11,912)	2 (weighted 2)
**Kenya**	**Any online CSEA**	**21.7% (19.0–24.4)**	**1,014 (weighted 11,912)**	**230 (weighted 220)**
Kenya	Sent unwanted sexual images	12.8% (10.6–15.0)	1,014 (weighted 11,912)	132 (weighted 129)
Kenya	Unwanted sexual comments	9.3% (7.4–11.2)	1,014 (weighted 11,912)	100 (weighted 95)
Kenya	Asked for intimate photos/videos	6.0% (4.4–7.6)	1,014 (weighted 11,912)	62 (weighted 61)
Kenya	Asked to do something sexual	5.9% (4.3–7.5)	1,014 (weighted 11,912)	60 (weighted 60)
Kenya	Asked to talk about sex/acts	5.7% (4.2–7.2)	1,014 (weighted 11,912)	60 (weighted 58)
Kenya	Offered money/gifts for images	4.3% (3.0–5.7)	1,014 (weighted 11,912)	45 (weighted 44)
Kenya	Shared sexual images without consent	4.2% (2.8–5.5)	1,014 (weighted 11,912)	42 (weighted 42)
Kenya	Threatened/blackmailed for sex	3.8% (2.5–5.0)	1,014 (weighted 11,912)	38 (weighted 38)
Kenya	Offered money/gifts for in-person acts	3.8% (2.5–5.0)	1,014 (weighted 11,912)	38 (weighted 38)
**Malaysia**	**Any online CSEA**	**10.2% (8.1–12.4)**	**995 (weighted 11,912)**	**109 (weighted 102)**
Malaysia	Sent unwanted sexual images	6.4% (4.6–8.1)	995 (weighted 11,912)	67 (weighted 63)
Malaysia	Unwanted sexual comments	4.7% (3.3–6.2)	995 (weighted 11,912)	47 (weighted 47)
Malaysia	Asked to talk about sex/acts	2.4% (1.5–3.4)	995 (weighted 11,912)	28 (weighted 24)
Malaysia	Asked to do something sexual	2.0% (1.0–3.0)	995 (weighted 11,912)	19 (weighted 20)
Malaysia	Shared sexual images without consent	1.6% (0.7–2.5)	995 (weighted 11,912)	15 (weighted 16)
Malaysia	Asked for intimate photos/videos	1.6% (0.7–2.4)	995 (weighted 11,912)	16 (weighted 16)

Country	Type of Abuse	Prevalence (95% CI)	Denominator	Children Affected
Malaysia	Offered money/gifts for images	0.8% (0.3–1.3)	995 (weighted 11,912)	10 (weighted 8)
Malaysia	Threatened/blackmailed for sex	0.6% (0.1–1.1)	995 (weighted 11,912)	6 (weighted 6)
Malaysia	Offered money/gifts for in-person acts	0.5% (0.0–1.1)	995 (weighted 11,912)	4 (weighted 5)
**Mozambique**	**Any online CSEA**	**25.9% (22.6–29.1)**	**999 (weighted 11,912)**	**231 (weighted 258)**
Mozambique	Sent unwanted sexual images	16.1% (13.3–18.9)	999 (weighted 11,912)	149 (weighted 161)
Mozambique	Unwanted sexual comments	8.4% (6.3–10.6)	999 (weighted 11,912)	74 (weighted 84)
Mozambique	Asked for intimate photos/videos	7.2% (5.2–9.3)	999 (weighted 11,912)	57 (weighted 72)
Mozambique	Asked to talk about sex/acts	6.3% (4.4–8.2)	999 (weighted 11,912)	49 (weighted 63)
Mozambique	Asked to do something sexual	4.2% (2.6–5.8)	999 (weighted 11,912)	33 (weighted 42)
Mozambique	Offered money/gifts for images	3.7% (2.2–5.2)	999 (weighted 11,912)	29 (weighted 37)
Mozambique	Threatened/blackmailed for sex	3.3% (1.9–4.8)	999 (weighted 11,912)	23 (weighted 33)
Mozambique	Offered money/gifts for in-person acts	3.2% (1.7–4.6)	999 (weighted 11,912)	24 (weighted 32)
Mozambique	Shared sexual images without consent	2.8% (1.4–4.1)	999 (weighted 11,912)	21 (weighted 28)
**Namibia**	**Any online CSEA**	**20.0% (17.1–22.8)**	**994 (weighted 11,912)**	**225 (weighted 198)**
Namibia	Unwanted sexual comments	10.6% (8.4–12.8)	994 (weighted 11,912)	118 (weighted 106)
Namibia	Sent unwanted sexual images	9.9% (7.8–12.0)	994 (weighted 11,912)	113 (weighted 99)
Namibia	Asked for intimate photos/videos	5.9% (4.2–7.7)	994 (weighted 11,912)	64 (weighted 59)
Namibia	Asked to talk about sex/acts	5.2% (3.9–6.6)	994 (weighted 11,912)	70 (weighted 52)
Namibia	Threatened/blackmailed for sex	3.4% (2.0–4.7)	994 (weighted 11,912)	33 (weighted 33)
Namibia	Offered money/gifts for images	3.3% (2.0–4.7)	994 (weighted 11,912)	34 (weighted 33)
Namibia	Shared sexual images without consent	3.2% (2.1–4.2)	994 (weighted 11,912)	40 (weighted 31)
Namibia	Asked to do something sexual	3.1% (1.9–4.3)	994 (weighted 11,912)	36 (weighted 31)
Namibia	Offered money/gifts for in-person acts	3.0% (1.8–4.2)	994 (weighted 11,912)	32 (weighted 30)
**Philippines**	**Any online CSEA**	**28.6% (25.3–31.9)**	**950 (weighted 11,912)**	**269 (weighted 271)**
Philippines	Sent unwanted sexual images	16.0% (13.4–18.7)	950 (weighted 11,912)	156 (weighted 152)

Country	Type of Abuse	Prevalence (95% CI)	Denominator	Children Affected
Philippines	Unwanted sexual comments	14.5% (11.8–17.1)	950 (weighted 11,912)	128 (weighted 137)
Philippines	Asked to talk about sex/acts	9.4% (7.2–11.5)	950 (weighted 11,912)	80 (weighted 89)
Philippines	Asked for intimate photos/videos	7.3% (5.4–9.2)	950 (weighted 11,912)	71 (weighted 70)
Philippines	Asked to do something sexual	6.8% (5.0–8.6)	950 (weighted 11,912)	65 (weighted 64)
Philippines	Shared sexual images without consent	5.3% (3.7–7.0)	950 (weighted 11,912)	50 (weighted 51)
Philippines	Offered money/gifts for images	5.3% (3.7–6.9)	950 (weighted 11,912)	53 (weighted 51)
Philippines	Offered money/gifts for in-person acts	4.7% (3.2–6.1)	950 (weighted 11,912)	48 (weighted 44)
Philippines	Threatened/blackmailed for sex	4.5% (2.9–6.0)	950 (weighted 11,912)	42 (weighted 42)
**Tanzania**	**Any online CSEA**	**7.7% (6.0–9.5)**	**996 (weighted 11,912)**	**88 (weighted 77)**
Tanzania	Sent unwanted sexual images	3.4% (2.3–4.6)	996 (weighted 11,912)	40 (weighted 34)
Tanzania	Asked to do something sexual	2.5% (1.5–3.5)	996 (weighted 11,912)	27 (weighted 25)
Tanzania	Unwanted sexual comments	2.4% (1.5–3.3)	996 (weighted 11,912)	32 (weighted 24)
Tanzania	Asked to talk about sex/acts	2.0% (1.1–2.9)	996 (weighted 11,912)	23 (weighted 20)
Tanzania	Offered money/gifts for in-person acts	1.1% (0.5–1.8)	996 (weighted 11,912)	12 (weighted 11)
Tanzania	Offered money/gifts for images	1.1% (0.4–1.9)	996 (weighted 11,912)	11 (weighted 11)
Tanzania	Threatened/blackmailed for sex	1.1% (0.3–1.9)	996 (weighted 11,912)	8 (weighted 11)
Tanzania	Asked for intimate photos/videos	1.0% (0.3–1.7)	996 (weighted 11,912)	9 (weighted 10)
Tanzania	Shared sexual images without consent	1.0% (0.3–1.6)	996 (weighted 11,912)	9 (weighted 10)
**Thailand**	**Any online CSEA**	**17.0% (14.3–19.6)**	**967 (weighted 11,912)**	**157 (weighted 164)**
Thailand	Sent unwanted sexual images	11.4% (9.2–13.7)	967 (weighted 11,912)	105 (weighted 111)
Thailand	Unwanted sexual comments	10.3% (8.2–12.4)	967 (weighted 11,912)	96 (weighted 99)
Thailand	Asked to talk about sex/acts	7.5% (5.7–9.3)	967 (weighted 11,912)	70 (weighted 72)
Thailand	Asked to do something sexual	6.5% (4.8–8.2)	967 (weighted 11,912)	63 (weighted 63)
Thailand	Asked for intimate photos/videos	6.1% (4.4–7.8)	967 (weighted 11,912)	55 (weighted 59)
Thailand	Offered money/gifts for in-person acts	5.5% (3.9–7.1)	967 (weighted 11,912)	50 (weighted 53)

Country	Type of Abuse	Prevalence (95% CI)	Denominator	Children Affected
Thailand	Shared sexual images without consent	5.2% (3.6–6.7)	967 (weighted 11,912)	48 (weighted 50)
Thailand	Threatened/blackmailed for sex	5.1% (3.6–6.7)	967 (weighted 11,912)	49 (weighted 50)
Thailand	Offered money/gifts for images	5.0% (3.5–6.5)	967 (weighted 11,912)	46 (weighted 48)
**Uganda**	**Any online CSEA**	**27.7% (24.6–30.8)**	**1,016 (weighted 11,912)**	**311 (weighted 281)**
Uganda	Sent unwanted sexual images	16.2% (13.6–18.7)	1,016 (weighted 11,912)	185 (weighted 164)
Uganda	Unwanted sexual comments	9.9% (7.8–11.9)	1,016 (weighted 11,912)	112 (weighted 100)
Uganda	Asked to talk about sex/acts	8.2% (6.3–10.1)	1,016 (weighted 11,912)	96 (weighted 84)
Uganda	Asked for intimate photos/videos	6.9% (5.3–8.6)	1,016 (weighted 11,912)	86 (weighted 71)
Uganda	Asked to do something sexual	6.3% (4.7–7.8)	1,016 (weighted 11,912)	81 (weighted 64)
Uganda	Offered money/gifts for in-person acts	5.1% (3.6–6.5)	1,016 (weighted 11,912)	57 (weighted 51)
Uganda	Shared sexual images without consent	5.0% (3.5–6.4)	1,016 (weighted 11,912)	53 (weighted 50)
Uganda	Offered money/gifts for images	4.2% (2.8–5.5)	1,016 (weighted 11,912)	48 (weighted 42)
Uganda	Threatened/blackmailed for sex	3.3% (2.2–4.4)	1,016 (weighted 11,912)	41 (weighted 34)
**Vietnam**	**Any online CSEA**	**5.5% (3.7–7.3)**	**994 (weighted 11,912)**	**49 (weighted 54)**
Vietnam	Sent unwanted sexual images	3.1% (1.7–4.5)	994 (weighted 11,912)	25 (weighted 31)
Vietnam	Unwanted sexual comments	2.4% (1.2–3.6)	994 (weighted 11,912)	25 (weighted 24)
Vietnam	Asked to talk about sex/acts	0.8% (0.0–1.5)	994 (weighted 11,912)	6 (weighted 8)
Vietnam	Asked to do something sexual	0.4% (-0.1–0.8)	994 (weighted 11,912)	3 (weighted 4)
Vietnam	Shared sexual images without consent	0.3% (-0.1–0.6)	994 (weighted 11,912)	3 (weighted 3)
Vietnam	Asked for intimate photos/videos	0.2% (-0.1–0.5)	994 (weighted 11,912)	2 (weighted 2)
Vietnam	Offered money/gifts for in-person acts	0.2% (-0.1–0.4)	994 (weighted 11,912)	2 (weighted 2)
Vietnam	Offered money/gifts for images	0.1% (-0.1–0.4)	994 (weighted 11,912)	2 (weighted 1)
Vietnam	Threatened/blackmailed for sex	0.0% (0.0–0.0)	994 (weighted 11,912)	0 (weighted 0)

Table 53: Survey-weighted percentages of children reporting each location context for nine forms of technology-facilitated CSEA (2020–2021). For each CSEA type, the denominator is restricted to children who experienced that specific harm, so estimates reflect the conditional distribution of location among those affected. Location categories capture whether the incident occurred via social media, online gaming platform, in person, or other context; percentages are non-additive as children could report multiple locations. Weighted Ns reflect survey-scaled totals used for estimation. Confidence intervals are design-based (95%), calculated using survey-weighted variance.

**CSEA type**	**Children Affected (N; unweighted, weighted)**	**Social media**	**Gaming**	**In person**	**Other**
Unwanted sexual comments	1,952 (weighted 1908)	38.2% (35.7–40.7)	11.4% (9.8–13.0)	32.0% (29.6–34.3)	9.0% (7.5–10.4)
Sent unwanted sexual images	1,940 (weighted 1902)	52.3% (49.8–54.9)	11.6% (10.0–13.3)	16.5% (14.6–18.5)	11.2% (9.6–12.8)
Asked to talk about sex/sexual acts	1,200 (weighted 1160)	41.9% (38.7–45.1)	11.7% (9.6–13.8)	31.7% (28.7–34.7)	11.4% (9.4–13.5)
Asked to do something sexual	1,078 (weighted 1060)	35.3% (32.1–38.5)	13.3% (11.0–15.6)	31.4% (28.3–34.5)	12.4% (10.2–14.6)
Asked for intimate photos/videos	878 (weighted 885)	47.5% (43.7–51.3)	14.2% (11.5–16.8)	22.0% (18.9–25.2)	8.6% (6.6–10.6)
Offered money/gifts for sexual images	681 (weighted 653)	38.4% (34.2–42.6)	17.2% (14.0–20.4)	24.2% (20.5–27.9)	13.3% (10.5–16.0)
Offered money/gifts for in-person sexual acts	694 (weighted 671)	39.4% (35.3–43.6)	16.9% (13.7–20.0)	26.5% (22.9–30.2)	12.0% (9.3–14.7)
Shared sexual images without consent	671 (weighted 646)	41.4% (37.1–45.6)	17.4% (14.2–20.6)	18.6% (15.3–21.9)	13.9% (11.0–16.9)
Threatened/blackmailed for sexual activities	636 (weighted 621)	38.5% (34.2–42.8)	16.2% (13.1–19.4)	24.5% (20.8–28.2)	13.7% (10.6–16.7)

Table 54: Survey-weighted prevalence of technology-facilitated CSEA (TF-CSEA) across three operationalisations, by country (2020–2021). TF-CSEA (primary measure) captures experiences occurring exclusively via social media or online gaming platforms. Any CSEA exposure captures any experience across all contexts regardless of platform, including in-person incidents. Inferred TF- CSEA treats intrinsically digital items (receiving unwanted sexual images, requests for intimate images, offers of money for sexual images, and non-consensual sharing of sexual images) as online by definition, and requires an explicit platform flag for contextually ambiguous items. Differences across operationalisations within countries reflect the proportion of CSEA that is platform-specific versus context-independent. Values are survey-weighted prevalence estimates with 95% confidence intervals.

**Characteristic**	**Overall** N = 11,912[1]	**Cambodia** N = 992[1]	**Ethiopia** N = 1,000[1]	**Indonesia** N = 995[1]	**Kenya** N = 1,014[1]	**Malaysia** N = 995[1]	**Mozambique** N = 999[1]	**Namibia** N = 994[1]	**Philippines** N = 950[1]	**Tanzania** N = 996[1]	**Thailand** N = 967[1]	**Uganda** N = 1,016[1]	**Vietnam** N = 994[1]
TF-CSEA (primary)	2,025 (17%)	145 (15%)	186 (19%)	66 (6.6%)	220 (22%)	102 (10%)	258 (26%)	198 (20%)	271 (29%)	77 (7.7%)	164 (17%)	281 (28%)	54 (5.5%)
Inferred TF-CSEA	2,761 (23%)	239 (24%)	267 (27%)	93 (9.3%)	283 (28%)	126 (13%)	353 (35%)	242 (24%)	393 (41%)	95 (9.6%)	206 (21%)	389 (38%)	75 (7.6%)
Any CSEA exposure	3,477 (31%)	287 (33%)	355 (40%)	122 (13%)	370 (38%)	151 (16%)	435 (46%)	273 (28%)	458 (52%)	144 (16%)	245 (26%)	514 (52%)	123 (13%)
Unknown	728	115	115	61	39	50	61	26	73	68	38	33	50

[1]n (%)

Table 55: Weighted distribution of barrier multiplicity among non-disclosed incidents of technology-facilitated CSEA (2020–2021). Each row represents the number of barriers reported per non-disclosed incident (child by CSEA type combination), grouped into four categories: none selected, one barrier, two to three barriers, and four or more barriers. Weighted percentages use survey probability weights (wgt_scaled) to provide population-representative estimates; no confidence intervals are reported as variance estimation was not applied at this stage. The denominator is all non-disclosed incidents with survey weight data. Results show that most children cited only one barrier to disclosure, indicating that non-disclosure was typically driven by a single dominant concern rather than multiple overlapping barriers.

Number of barriers	Weighted % of non-disclosed incidents
0 (none selected)	9.6
1	74.6
2–3	13.3
4+	2.5

Table 56: The five most commonly co-occurring pairs of barriers to disclosure among non-disclosed incidents of technology-facilitated CSEA (2020–2021). For each pair, the weighted percentage reflects the proportion of non-disclosed incidents in which both barriers were endorsed simultaneously. Estimates are derived from the weighted 13×13 co-occurrence matrix using survey probability weights (wgt_scaled); off-diagonal cells represent joint occurrence. Low joint percentages relative to individual barrier prevalence indicate that barriers largely operated independently rather than clustering into fixed profiles, suggesting heterogeneous pathways to non-disclosure across children.

Barrier 1	Barrier 2	Percent
Did not know where to go/who to tell	Embarrassed/ashamed/too difficult	2.7
Embarrassed/ashamed/too difficult	Worried I would get in trouble	1.8
Embarrassed/ashamed/too difficult	Thought no one would believe me	1.6
Thought no one would believe me	Worried I would get in trouble	1.6
Did not know where to go/who to tell	Thought no one would believe me	1.5

8 References

Arel-Bundock, V., Greifer, N., & Heiss, A. (2024). How to interpret statistical models using marginaleffects for R and Python. *Journal of Statistical Software*, 111(9), 1–32. https://doi.org/10.18637/jss.v111.i09

Brown, L. D., Cai, T. T., & DasGupta, A. (2001). Interval estimation for a binomial proportion. *Statistical Science*, 16(2), 101–133.

Gelman, A., Vehtari, A., Simpson, D., Margossian, C. C., Carpenter, B., Yao, Y., … & Modrák, M. (2020). *Bayesian workflow*. arXiv preprint arXiv:2011.01808.

International Telecommunication Union. ITU DataHub: Individuals using the internet by age, sex, and location. ITU Statistics Division, Geneva. https://datahub.itu.int (accessed October 2025).

Kish, L. Survey Sampling. (Wiley, New York, 1965).

Larner, S. (2022). Facilitating children's informal disclosures of sexual abuse: The role of online counsellors at a national children's helpline. *Journal of Child Sexual Abuse*, 31(3), 276–296. https://doi.org/10.1080/10538712.2022.2047854

McElvaney, R. (2015). Disclosure of child sexual abuse: Delays, non□disclosure and partial disclosure. What the research tells us and implications for practice. *Child Abuse Review*, 24(3), 159–169. https://doi.org/10.1002/car.2280

Price, H. L., & Kehn, A. (2023). Potential reporters of suspected child maltreatment are sensitive to the amount of evidence and the potential consequences of reporting. *Journal of Interpersonal Violence*, 38(1–2), 391–417. https://doi.org/10.1177/08862605221081934

United Nations Development Programme (UNDP). (2020). *Human Development Report data*. https://hdr.undp.org/en/data

UNICEF Office of Research – Innocenti (2022). Children's Experiences of Online Sexual Exploitation and Abuse in 12 Countries in Eastern and Southern Africa and Southeast Asia. Disrupting Harm Data Insight 1. Global Partnership to End Violence Against Children.https://safeonline.global/wp-content/uploads/2023/12/DH-data-insight-1_Final.pdf

Vehtari, A., Gelman, A., Simpson, D., Carpenter, B., & Bürkner, P.-C. (2021). Rank-normalization, folding, and localization: An improved $\hat{R}$ for assessing convergence of MCMC. *Bayesian Analysis*, 16(2), 667–718.

Wolter, K. M. (2007). *Introduction to variance estimation* (2nd ed.). Springer Science + Business Media.
