# Supplementary material for: Technology mediation in child sexual exploitation and abuse in Africa and Asia
Source: Nature. 2026 May 27;654(8118):408–18. doi: 10.1038/s41586-026-10525-4 (PMC13253325; doi:10.1038/s41586-026-10525-4)
Supplement: Supplementary file 2 — Reporting Summary [file 41586_2026_10525_MOESM2_ESM.pdf]

nature portfolio

Corresponding author(s): Sakshi Ghai

Last updated by author(s): 9/03/2026

Reporting Summary

Nature Portfolio wishes to improve the reproducibility of the work that we publish. This form provides structure for consistency and transparency in reporting. For further information on Nature Portfolio policies, see our Editorial Policies and the Editorial Policy Checklist.

Statistics

For all statistical analyses, confirm that the following items are present in the figure legend, table legend, main text, or Methods section.

n/a	Confirmed	
☐	☒	The exact sample size (n) for each experimental group/condition, given as a discrete number and unit of measurement
☒	☐	A statement on whether measurements were taken from distinct samples or whether the same sample was measured repeatedly
☐	☒	The statistical test(s) used AND whether they are one- or two-sided<br>*Only common tests should be described solely by name; describe more complex techniques in the Methods section.*
☐	☒	A description of all covariates tested
☐	☒	A description of any assumptions or corrections, such as tests of normality and adjustment for multiple comparisons
☐	☒	A full description of the statistical parameters including central tendency (e.g. means) or other basic estimates (e.g. regression coefficient) AND variation (e.g. standard deviation) or associated estimates of uncertainty (e.g. confidence intervals)
☐	☒	For null hypothesis testing, the test statistic (e.g. F, t, r) with confidence intervals, effect sizes, degrees of freedom and P value noted<br>*Give P values as exact values whenever suitable.*
☐	☒	For Bayesian analysis, information on the choice of priors and Markov chain Monte Carlo settings
☐	☒	For hierarchical and complex designs, identification of the appropriate level for tests and full reporting of outcomes
☒	☐	Estimates of effect sizes (e.g. Cohen's d, Pearson's r), indicating how they were calculated

Our web collection on statistics for biologists contains articles on many of the points above.

Software and code

Policy information about availability of computer code

Data collection	Ipsos Mori collected the data using a combination of face-to-face household interviews and online interviews (due to the COVID-19 pandemic restrictions) to ensure maximum national coverage of the total population included in the survey.
Data analysis	All analyses were conducted in R (version 4.4.1).Key R packages used include: brms, cmdstanr, posterior, loo, bayestestR, marginaleffects, emmeans, tidyverse, survey, srvyr, mice, ggplot2, and ggdist. A full list of packages and version numbers is provided in the GitHub repository README (https://github.com/sghai9/technology-mediation-csea)

For manuscripts utilizing custom algorithms or software that are central to the research but not yet described in published literature, software must be made available to editors and reviewers. We strongly encourage code deposition in a community repository (e.g. GitHub). See the Nature Portfolio guidelines for submitting code & software for further information.

Data

Policy information about availability of data

All manuscripts must include a data availability statement. This statement should provide the following information, where applicable:

- Accession codes, unique identifiers, or web links for publicly available datasets
- A description of any restrictions on data availability
- For clinical datasets or third party data, please ensure that the statement adheres to our policy

The Disrupting Harm survey data used in this study were shared with the research team (S.G. and A.O.) under a data-sharing agreement with UNICEF Innocenti – Global Office of Research and Foresight. Due to the sensitive nature of the data, they cannot be made publicly available.

Research involving human participants, their data, or biological material

Policy information about studies with human participants or human data. See also policy information about sex, gender (identity/presentation), and sexual orientation and race, ethnicity and racism.

Reporting on sex and gender	Demographic data was collected on gender.
Reporting on race, ethnicity, or other socially relevant groupings	Demographic data was collected on material deprivation, religious affiliation, classification of local area (urban-rural), and education. No ethnicity data was collected.
Population characteristics	Children aged 12-17 years.
Recruitment	This study was designed to be representative of each country's digitally connected population. A random probability clustered sample design was used to ensure all households had an equal chance of being sampled.
Ethics oversight	The Disrupting Harm Survey was reviewed and approved by a global institutional review board (HML IRB Research and Ethics). In addition, ethics approval was obtained from national or institutional ethics review bodies in each of the 12 participating countries. These included: the National Commission for Science, Technology and Innovation (Kenya); Makerere University School of Public Health and the Uganda National Council of Science and Technology (Uganda); the Cambodia National Council for Children and the Ministry of Interior (Cambodia); the Ministry of Health, National Committee on Bioethics for Health (Mozambique); the Medical Research and Ethics Committee (Malaysia); the Health Research Ethics Committee, National Institute of Health Research and Development (Indonesia); the Ministry of Health and Social Services Ethical Review Board (Namibia); the Ministry of Labour, Invalids and Social Affairs (Vietnam); the Philippine Social Science Council Ethical Review Board (Philippines); the Ethiopian Society of Sociologists, Social Workers and Anthropologists (Ethiopia); and multiple bodies in Tanzania: the Medical Research, National Bureau of Statistics, and the President's Office–Regional Administration and Local Government, with permits from the Tanzania Commission for Science and Technology and additional approvals from the Zanzibar Health Research Institute. In Thailand, the study was reviewed by a special panel at Mahidol University's Institute of Human Rights and Peace Studies, as no formal government ethics review process exists for social research

Note that full information on the approval of the study protocol must also be provided in the manuscript.

Field-specific reporting

Please select the one below that is the best fit for your research. If you are not sure, read the appropriate sections before making your selection.

☐ Life sciences ☒ Behavioural & social sciences ☐ Ecological, evolutionary & environmental sciences

For a reference copy of the document with all sections, see nature.com/documents/nr-reporting-summary-flat.pdf

Behavioural & social sciences study design

All studies must disclose on these points even when the disclosure is negative.

Study description	This study uses a quantitative, cross-sectional survey design. Data are drawn from the nationally representative household survey component of the Disrupting Harm project: a multi-sectoral research initiative led by UNICEF Office of Research – Innocenti, ECPAT International, and INTERPOL, with funding from Safe Online. While the broader Disrupting Harm project employed a mixed-methods design across its three partner organisations, this paper analyses only the quantitative survey data collected by UNICEF. Approximately 1,000 internet-using children aged 12–17 were surveyed in each of 12 countries across Eastern and Southern Africa (Ethiopia, Kenya, Mozambique, Namibia, Tanzania, Uganda) and Southeast Asia (Cambodia, Indonesia, Malaysia, Philippines, Thailand, Vietnam).

Research sample	Children aged 12-17 living in Ethiopia, Kenya, Mozambique, Namibia, Tanzania, Uganda, Cambodia, Indonesia, Malaysia, the Philippines, Thailand, and Vietnam.
Sampling strategy	The sample is nationally representative of internet-using children aged 12–17 in each country. A stratified random cluster sample with random walk within clusters was used. Children were randomly selected at household level if they were aged 12–17 and had used the internet at least once in the past three months. Survey weights were applied in three stages: design weight adjustments to reflect probabilities of selection (inverse probability weights), non-response weights to reduce non-response bias, and post-stratification weights to adjust for differences between the sample and population distributions. The sampling design aimed at achieving full or near-full national coverage, making findings representative of internet-using children aged 12–17 in each participating country, rather than the general child population. The total analytic sample comprises N = 11,912 internet-using children across 12 countries. No formal a priori power calculation was conducted. Sample sizes of approximately 1,000 children per country were determined by UNICEF Office of Research: Innocenti and Ipsos as a standard survey design target for nationally representative household surveys, sufficient to provide adequate precision for country-level prevalence estimates of technology-facilitated CSEA. In some countries, conflict-affected or very remote areas were excluded from fieldwork coverage. Full coverage details are reported in the Supplementary Information.
Data collection	Data was collected by Ipsos MORI (and local Ipsos affiliates) on behalf of UNICEF Office of Research – Innocenti between 2020 and 2021. The survey instrument was adapted from the Global Kids Online questionnaire (originally developed by UNICEF Innocenti and the London School of Economics), with roots in the EU Kids Online survey (2012) with additional modules developed specifically for the Disrupting Harm project. The survey used a mixed-mode design combining computer-assisted personal interviewing and computer-assisted self-interviewing. Questions relating to children's life context and general internet use were administered face-to-face by trained enumerators to build rapport. Sensitive sections covering sex and sexual violence including the nine items assessing technology-facilitated CSEA were completed by children themselves in self-complete mode on a tablet or phone, primarily to protect privacy and to allow children to feel more comfortable responding. Enumerators remained available throughout to answer questions, address concerns, and activate the safeguarding and referral protocol if needed. Regardless of mode, children retained the right to skip any question or section or to stop the interview at any time. As this is an observational survey study with no experimental conditions, blinding of interviewers to the study topic was not applicable. While other household members were sometimes present during face-to-face portions, the self-complete mode for sensitive questions provided additional privacy protection.
Timing	Data collection took place between January 2020 and November 2021 across 12 countries. In Eastern and Southern Africa, fieldwork began earliest in Uganda (January 2020). Fieldwork in Ethiopia, Kenya and Tanzania was conducted between December 2020 and January 2021, whereas Namibia ran from December 2020 to February 2021. Mozambique had a staggered fieldwork schedule, beginning in the south in February 2021 and in the central and northern regions in May 2021, with all fieldwork completed in July 2021. In South-East Asia, fieldwork began in Cambodia (November 2020), followed by Thailand and Vietnam (November 2020 to February 2021) and Indonesia (November 2020 to February 2021). Fieldwork in the Philippines ran from January to April 2021, and Malaysia had the latest collection window, from April to November 2021. Each country targeted a sample of 1,000 internet-using children aged 12–17 years. Achieved unweighted sample sizes ranged from 975 (Philippines) to 1,111 (Mozambique), and weighted sample sizes ranged from 950 (Philippines) to 1,016 (Uganda).
Data exclusions	In the Disrupting Harm survey, we adjusted each county s sample size to reflect survey weights, which led to the exclusion of 707 cases. We then removed planned exclusions, including non-responders and one-item variable with high missing data.
Non-participation	NA
Randomization	Randomization was employed at multiple stages of the sampling procedure. A stratified random cluster sampling approach was used, with primary sampling units (clusters) selected with probability proportional to size. Within selected clusters, households were identified through a random walk procedure. Within eligible households, if more than one child aged 12–17 met the eligibility criteria (internet use in the past three months), a single respondent was selected. This multi-stage random sampling procedure ensured that every eligible child in each country had a non-zero probability of selection, and survey weights were applied to account for unequal probabilities of selection, non-response, and post-stratification.

Reporting for specific materials, systems and methods

We require information from authors about some types of materials, experimental systems and methods used in many studies. Here, indicate whether each material, system or method listed is relevant to your study. If you are not sure if a list item applies to your research, read the appropriate section before selecting a response.

Materials & experimental systems

n/a	Involved in the study	
☒	☐	Antibodies
☒	☐	Eukaryotic cell lines
☒	☐	Palaeontology and archaeology
☒	☐	Animals and other organisms
☒	☐	Clinical data
☒	☐	Dual use research of concern
☒	☐	Plants

Methods

n/a	Involved in the study	
☒	☐	ChIP-seq
☒	☐	Flow cytometry
☒	☐	MRI-based neuroimaging

Plants

Seed stocks	*Report on the source of all seed stocks or other plant material used. If applicable, state the seed stock centre and catalogue number. If plant specimens were collected from the field, describe the collection location, date and sampling procedures.*
Novel plant genotypes	*Describe the methods by which all novel plant genotypes were produced. This includes those generated by transgenic approaches, gene editing, chemical/radiation-based mutagenesis and hybridization. For transgenic lines, describe the transformation method, the number of independent lines analyzed and the generation upon which experiments were performed. For gene-edited lines, describe the editor used, the endogenous sequence targeted for editing, the targeting guide RNA sequence (if applicable) and how the editor was applied.*
Authentication	*Describe any authentication procedures for each seed stock used or novel genotype generated. Describe any experiments used to assess the effect of a mutation and, where applicable, how potential secondary effects (e.g. second site T-DNA insertions, mosiacism, off-target gene editing) were examined.*
