# Supplementary material for: Technology mediation in child sexual exploitation and abuse in Africa and Asia
Source: Nature. 2026 May 27;654(8118):408–18. doi: 10.1038/s41586-026-10525-4 (PMC13253325; doi:10.1038/s41586-026-10525-4)
Supplement: Supplementary file 3 — Peer Review File [file 41586_2026_10525_MOESM3_ESM.pdf]

nature portfolio

Peer Review File

Technology mediation in child sexual exploitation and abuse in Africa and Asia

Corresponding Author: Dr Sakshi Ghai

Any redactions in this file are there to maintain patient confidentiality, the confidentiality of unpublished data, or to remove third-party material.

This file contains all reviewer reports in order by version, followed by all author rebuttals in order by version.

Version 1:

Reviewer comments:

Referee #1

(Remarks to the Author)
Thank you for the opportunity to review, “Technology-facilitated child sexual exploitation and abuse in Eastern and Southern Africa and Southeast Asia.” Below, I have detailed comments related to this review, as provided by the guideline for reviewers. I am happy to provide any clarification on points listed below.

1. Key Results

One of the most valuable contributions of this manuscript is its focus on low- and middle-income countries, an area where empirical research on technology-facilitated child sexual abuse (CSA) remains particularly limited. By situating the study in these contexts, the authors significantly expand the global scope of scholarship on CSA.

Similarly, the attention to technology-facilitated CSA is important, as this remains an underdeveloped area of research.

I found the authors’ harm-focused framing—particularly their observation that “although not every online risk leads to harm, adopting a harm focused framing is crucial to fully recognising the severity of children’s experiences, and safeguarding their rights”—to be a powerful and persuasive approach that directly addresses common criticisms of being “too sensitive” about behaviors such as online harassment.

Methodologically, the approach is well described and appropriate, and the paper is written in an engaging, accessible style. Indeed, the opening sections are so well framed that I could imagine using them as an example for my graduate students.

2. Validity

I did not identify any flaws that would prohibit publication of this manuscript. The methods appear sound and the analyses are appropriately applied. My main concern lies with the underdeveloped discussion section, which would benefit from further elaboration before publication. Expanding this section would allow the authors to highlight the broader implications of their findings and strengthen the overall contribution of the paper.

3. Originality and Significance

While the methodological approach itself is not novel, the focus on low- and middle-income countries is both original and highly significant. Scholars have increasingly called for greater attention to the Global South in research on child sexual abuse and prevention, yet the literature remains sparse. By centering these contexts, this paper addresses a pressing gap and provides insights that are highly relevant across disciplines, including criminology, public health, sociology, and international development. Given this, I expect the article has strong potential to be widely cited and influential.

4. Data and Methodology

The methods employed are appropriate for the research questions, and the data are presented with care. However, some of the figures are overly complex and may be difficult for the average Nature reader to interpret efficiently. For instance, I found Figure 3 somewhat challenging and required more time than expected to distill its main points. Similarly, Extended Figure 1 is dense and may limit its utility. While I do not have a ready alternative, I encourage the authors to consider whether the data could be presented in a clearer visual format that prioritizes accessibility for a broad readership.

5. Appropriate Use of Statistics and Treatment of Uncertainties

The statistical approaches appear appropriate and meets expected standards. One point that warrants clarification is the authors’ use of multiple imputation by chained equations (MICE) to address missing data. While this is a reasonable choice, the manuscript does not explain why this method was selected over alternatives. A brief rationale would help strengthen confidence in the robustness of the analytic approach.

6. Conclusions

The conclusions are well grounded in the analyses and are presented with appropriate caution. None appear overstated. The results align with prior research while also highlighting meaningful variation across countries, which adds an important dimension to the literature. Overall, the conclusions are robust, valid, and reliable.

7. Suggested Improvements

Several areas could be strengthened in revision. First, the introduction and discussion would benefit from a more substantive treatment of primary prevention. The current framing positions detection as the first step in addressing CSA, and it is, when viewing this problem through a criminal justice lens. However, from a public health perspective, primary prevention is the more foundational step. The data presented here suggest insights into what prevention might look like, and further exploration of this theme would add significant value.

Second, the discussion of protective factors—such as sex education, digital skills, and knowledge of help-seeking resources—would benefit from greater clarity. Do the authors conceptualize these as individual-level factors within the social ecological model, or do they also operate at broader levels?

Third, the treatment of barriers to disclosure could be refined. Since only those who did not disclose were asked about barriers, important nuances may be missed, as individuals who did disclose may also have faced barriers but eventually overcame them. I imagine that this is a feature of the design of the survey, and not the analytic decision made by the authors. Still, noting this limitation explicitly would improve transparency.

Finally, although the manuscript acknowledges the importance of including rural and peri-urban contexts in research, the discussion does not meaningfully engage with findings from these settings. Incorporating this dimension would further strengthen the contribution.

8. References

The manuscript cites relevant and appropriate literature throughout. The references are up to date and sufficiently comprehensive.

9. Clarity and Context

The abstract is clear, accessible, and appropriately structured according to the journal’s guidelines. It provides an accurate and concise summary of the study, highlighting its scope and contributions in a way that will be understandable to a wide audience. The introduction is similarly well framed and situates the study effectively within the existing literature.

Again, I would like to highlight the quality of writing in these sections.

(Remarks on code availability)
No--I am currently abroad and working on an old laptop, so I do not have the capacity to check the code. So I defer to the other reviewers and the editor here.

Referee #2

(Remarks to the Author)
The study examined children’s self-reported experiences of technology-facilitated CSEA across 12 countries. The topic is of great interest, and the paper is well-written, but I have several concerns and comments for the authors to consider.

1. The authors should clearly specify the reference time period used to measure the prevalence of technology-facilitated CSEA—was it the past 6 months, past year, or past two years?

2. When estimating the prevalence of technology-facilitated CSEA, the authors used only internet-using children as the

denominator. It would be helpful for the authors to justify this choice. In my view, both approaches—using all children (e.g., all children aged 12–17 in the population) and only internet-using children—provide valuable but different insights. Prevalence among all children reflects the overall population burden, while prevalence among internet users more accurately captures individual-level risk. The authors may wish to consider a dual reporting approach that presents prevalence estimates for both the general child population and internet users.

3. In lines 183–184, the sentence "We found that 51% of boys (n=1,026) and 49% of girls (n=999) ranging from 12 to 17 years old experienced at least one of the different forms of technology-facilitated CSEA" is not particularly informative. As written, it does not clarify whether boys or girls are more likely to experience technology-facilitated CSEA, since it lacks context on the underlying gender distribution among internet-using children. Depending on the original gender ratio (e.g., 52% boys and 48% girls, or vice versa), the interpretation of these numbers could vary significantly. A more informative approach would be to present the prevalence of experiencing at least one form of technology-facilitated CSEA separately for boys and for girls. This would better highlight any differences in risk between the two groups.

4. In the subsection "Disclosure of Technology-Facilitated CSEA," it would be helpful for the authors to clarify at the outset whether participants' responses to the disclosure questions were limited to a single choice or allowed for multiple selections. For example, if a child disclosed their experience to a parent, a friend, and a helpline, were they allowed to select all applicable options, or only the first person they disclosed to? This distinction is critical, as the interpretation of disclosure prevalence differs significantly depending on whether responses were single- or multiple-choice. The authors later mention that "children could select more than one disclosure type." If that is the case, it would be important to explain how disclosures to both formal and informal channels were handled in the subsequent analyses. Specifically, if a child disclosed to both types, were they classified as disclosing to both, or counted separately in each category? If the latter, how was this overlap accounted for, and what implications might it have for the findings and their interpretation? Children who disclose to both formal and informal channels may differ meaningfully from those who disclose only to one type. Simply including these children in both categories could blur distinctions between the groups, potentially limiting the clarity and validity of group comparisons.

5. I found Figure 3 somewhat difficult to interpret. For example, in the statement, "The most common barriers include children not knowing to whom they should disclose (e.g., 48% of instances involving sharing sexual images of the child without their consent)," does this mean that among all non-disclosure cases citing this specific barrier, 48% involved the sharing of sexual images without the child's consent? It would also be helpful for the authors to clarify whether the barrier question was asked only of children who did not disclose their CSEA experience, and whether respondents were allowed to select multiple barriers or only one. If multiple responses were permitted, how were cases reporting several barriers handled in the analysis? Were such cases counted multiple times across categories? The interpretation of the findings could vary considerably depending on the degree of overlap among reported barriers, so further clarification would strengthen readers' understanding. The authors may also wish to consider whether the overlap among barriers should be analyzed, in addition to treating them separately.

6. I have some concerns about the use of a set of strongly interrelated predictors in modeling disclosure outcomes. When predictors are highly correlated, there is a risk of multicollinearity, which can distort the estimation of coefficients in a regression model. Specifically, multicollinearity can make it difficult to disentangle the unique effect of each predictor, potentially leading to misleading conclusions. A variable that is not causally related to the outcome might appear significant simply because it is correlated with a true causal factor whose effect is masked by multicollinearity. This can also result in apparent inconsistencies—where some correlated variables are significant predictors for one disclosure outcome but not another—due to statistical noise rather than meaningful differences. Such issues may partly explain findings that seem random or that contradict prior literature. The authors should consider testing for multicollinearity (e.g., using variance inflation factors) and may want to explore dimension reduction techniques or alternative model specifications to address this concern.

7. In addition, the possibility of reverse causality is a critical concern, especially given the cross-sectional nature of the data. Since all variables are measured at the same point in time, it is difficult to determine whether certain factors preceded the disclosure or occurred as a result of it. For example, the finding that "children who knew where to seek help following an assault were more likely to disclose" (lines 262–263) could reflect the reverse: children who disclosed may have subsequently received guidance about where to seek help. Similarly, the finding that "positive parental mediation was positively associated with a higher likelihood of disclosing through informal channels" (lines 271-273) may also be subject to reverse causality—children who disclosed to their parents might have prompted greater parental involvement in their media use as a response, rather than a cause.
Regardless of direction, the associations may still appear statistically significant in regression models. However, the policy implications would differ drastically depending on whether the factor is a cause or a consequence. It is important that the authors avoid interpreting such associations as causal effects without stronger methodological support. If the intent is to inform policy or intervention design, the authors should consider methods that better address causality, such as instrumental variable techniques or longitudinal designs, where feasible.

(Remarks on code availability)

Referee #3

(Remarks to the Author)
I am focusing my review on data and methodology, and appropriate use of statistics and treatment of uncertainties.

1. Clarity for in-text descriptions of parameter estimates: On page 10 there are some in-text descriptions of parameter estimates from the models that require some additional explication to be meaningful. For instance, “Older children are more likely to experience technology-facilitated CSEA (posterior mean = 0.18, SD = 0.04, 95% Bayesian Credible Interval (CI) = [0.10 to 0.26], and posterior probability of direction (PD) = 99.9%)”. In this instance, how age was parameterized in the models is unclear, so it’s unclear whether this posterior reflects a year-over-year difference, or a contrast between some specific coding of “older” vs “younger”. Similarly, continuing in the same sentence, “whereas gender was not credibly associated with it (posterior mean = -0.01, SD = 0.11, 95% CI = [-0.22 to 0.20], PD = 46%)” the paramererization of gender is unclear, so whether this posterior is describing the difference from girls to boys or boys to girls is not apparent. I recommend the authors carefully consider whether they are supplying sufficient information to map their conclusions to the posterior distributions for parameters of interest.

2. Clarity on priors: I believe the authors use of the horseshoe prior is a reasonable choice given the lack of LASSO capabilities in brms. However, the authors are not clear on their prior strategy across all other models, and for the other parameters in the horseshoe model. From reviewing the code, it is apparent that the authors use the brms defaults in all models, save for the horseshoe regression, in which they apply the horseshoe prior to the beta parameters, and leave all other parameters at the default. This means that there are flat, uninformative priors set on all beta parameters, and half student-t distributions set on the random effects. The authors should be clear about this, and justify this decision. Otherwise, just as with the horseshoe prior employed to achieve a specific purpose, the authors can be more deliberate in their choice of prior distributions to achieve valid inference.

3. Appropriate parameter labeling in supplement tables: Similar to the latter point in the comment above, in Table 3 in the supplemental file, the parameter “Sex” should be labeled as whichever category is being contrasted to the intercept. Again, is this the difference from girls compared to boys, or vice-versa. This same point applies to all tables in the supplement file, as well as Figure 4 and extended Figures 1 and 2 in the main text

4. Professional labeling for graphics: Similarly, Figure 16 in the supplement should alter the parameter labeling for “Sex1” to something more human-readable.

5. Determination age x sex interaction and differences across countries: In the “Demographic Differences” section, the authors describe the positive association between age and CSEA, and the uncertain gender differences. However, then they note “a slight increase in the prevalence of … CSEA for older girls compared to boys…” presumably coming from the interaction effect in Table 3 in the supplementary materials. However, the use of the non-linear link function in the logit regression makes the interpretation of interactions from product terms somewhat unreliable (see Long & Mustillo, 2021). Similar to another analysis the authors employed, I would recommend using LOO cross validation here to compare a specification in which age and sex are additive, to this alternate specification in which they interact. This should be the basis of determining whether the difference the authors describe in the demographics section is systematic.

Long, J. S., & Mustillo, S. A. (2021). Using predictions and marginal effects to compare groups in regression models for binary outcomes. Sociological Methods & Research, 50(3), 1284-1320.

6. Determination of country differences in associations via random slopes and multimodel comparison: In Table 13, the author(s) present the results of their LOO cross validation to determine whether the association between predictors of CSEA disclosure vary across countries. This seems to contrast two possibilities – a model in which the associations between each predictor and the outcome are consistent across all countries, and then a second model in which the association between *every* predictor and the outcome varies across countries. It is not surprising that the LOO reveals the latter model to not be a systematic improvement over the baseline. I believe the authors are at risk of throwing the baby out with the bathwater here. What I would recommend is that the authors need to consider each predictor individually here. You have a baseline additive model, and then estimate a series of supplemental models in which each predictor is allowed to have a random slope, one at a time (while still including all other predicitors as fixed effects). The LOO is then compared for each model back to the baseline to determine whether *that specific predictor* has an association that varies across the countries.

The results from this analysis will make a more compelling and informative case regarding whether specific predictors indeed have associations that span multiple countries, or are specific to certain countries.

Signed: Jason Rydberg, University of Massachusetts Lowell

(Remarks on code availability)

Referee #4

(Remarks to the Author)

A. Summary of the key results
Thank you for the opportunity to review the manuscript titled “Technology-facilitated child sexual exploitation and abuse in Eastern and Southern Africa and Southeast Asia”. The paper uses nationally representative household data from 12

countries in Eastern and Southern Africa and Southeast Asia that were part of the Disrupting Harm surveys to examine technology-facilitated child sexual exploitation and abuse (CSEA).

B. Originality and significance: if not novel, please include reference
The manuscript addresses a significant gap in the technology-facilitated CSEA literature by investigating the prevalence of online abuse disclosure across countries. This is a highly significant, well-written manuscript.

C. Data & methodology: validity of approach, quality of data, quality of presentation
Appropriate.

D. Appropriate use of statistics and treatment of uncertainties
Appropriate.

E. Conclusions: robustness, validity, reliability
See suggestions in section H.

F. Suggested improvements: experiments, data for possible revision
No data revisions suggested.

G. References: appropriate credit to previous work?
Suggest updated search for prevalence of technology-facilitated abuse:
See: Ben Mathews recent work in Australia; David Finkelhor in the USA; also:
Fry, Deborah, et al. "Prevalence estimates and nature of online child sexual exploitation and abuse: a systematic review and meta-analysis." The Lancet Child & Adolescent Health 9.3 (2025): 184-193.

H. Clarity and context: lucidity of abstract/summary, appropriateness of abstract, introduction and conclusions

(minor) Lines 225 – 227 “Addressing other barriers such as feelings of shame, fears that the child, the perpetrator, or their family might get in trouble and underestimations of the severity of technology-facilitated CSEA will also be crucial for ensuring effective future interventions.” Suggest adding “Addressing other barriers identified in this study such as (…)

Lines 382 - 384 “These estimates, however, provide likely conservative approximations of the true scale of technology-facilitated CSEA, as one would expect severe underreporting of this sensitive issue." There seems to be some conflation between disclosure and reporting in this sentence. The severe underreporting is often associated with reports made to formal channels, such as law enforcement and child protective services. Given that this is a victim survey at the population level, would you still expect severe underreporting? Perhaps the numbers in the present study don't reflect the true prevalence, given social desirability bias in the data collected from face-to-face interviews, or because some participants may still experience victimization until they turn 18. However, the underrepresentation in a victim survey differs from that in crime or social services data.

Lines 379 – 384 “Approximately one in six of the 11,912 internet-using children surveyed reported experiencing at least one instance of technology-facilitated CSEA during 2020-2021. When extrapolated to national population estimates, this translates to millions of children exposed to potentially harmful experiences online in these countries alone.” It would be helpful to the reader if early on the authors included the range of internet using children across the 12 studied countries. Because the statistics “one in six” may be compared with other child sexual abuse related statistics that account for the entire population of children, I would suggest the authors add one sentence further highlighting that these numbers relate to internet using children, and cannot be directly extrapolated to the general population of children. In the sentence, “When extrapolated to national population estimates, this translates to millions of children exposed to potentially harmful experiences online in these countries alone.” Is this accounting only the proportion of children who use technology? I recommend that the authors clarify.

Line 406 – Heterogeneity refers to heterogeneity between countries?

Lines 557 to 568 – I suggest the authors include a rationale for why they chose to classify disclosure between formal vs. informal channels. Teachers and helplines could also be classified as informal channels in the sense that they don't trigger an investigation, as with a report to the police or social service agencies.

Line 682 – What is the range of percent missingness in the imputed variables?

(Remarks on code availability)

Version 2:

Reviewer comments:

Referee #1

(Remarks to the Author)
Thank you for the opportunity to review a revised version of , "Technology-facilitated child sexual exploitation and abuse in Eastern and Southern Africa and Southeast Asia." Below, I have included my original comments (#), the authors' response, often abbreviated for space (A), and any new response from me (B). I am happy to discuss any of the points below in more detail if needed.

1. Key Results

One of the most valuable contributions of this manuscript is its focus on low- and middle-income countries, an area where empirical research on technology-facilitated child sexual abuse (CSA) remains particularly limited. By situating the study in these contexts, the authors significantly expand the global scope of scholarship on CSA.

Similarly, the attention to technology-facilitated CSA is important, as this remains an underdeveloped area of research.

I found the authors' harm-focused framing—particularly their observation that "although not every online risk leads to harm, adopting a harm focused framing is crucial to fully recognising the severity of children's experiences, and safeguarding their rights"—to be a powerful and persuasive approach that directly addresses common criticisms of being "too sensitive" about behaviors such as online harassment.

Methodologically, the approach is well described and appropriate, and the paper is written in an engaging, accessible style. Indeed, the opening sections are so well framed that I could imagine using them as an example for my graduate students.

A. We are grateful to the reviewer for their generous comments on the framing, methodological clarity, and accessibility of the manuscript.

B. No further discussion.

2. Validity

I did not identify any flaws that would prohibit publication of this manuscript. The methods appear sound and the analyses are appropriately applied. My main concern lies with the underdeveloped discussion section, which would benefit from further elaboration before publication. Expanding this section would allow the authors to highlight the broader implications of their findings and strengthen the overall contribution of the paper.

A. To frame our revisions, we have reorganised our Introduction (see Point 7 below) around a children's rights and public health prevention framework that distinguishes primary prevention (prevention before abuse occurs), secondary prevention priorities (child protection and detection), and early tertiary priorities (response and recovery). To address the Reviewer 1's concerns, we have substantially revised the Discussion section by: (a) organising findings within this three-tier framework; (b) explicitly linking empirical patterns to actionable strategies at individual, family, cultural, and system levels; (c) proposing concrete future research directions; and (d) maintaining appropriate epistemic caution about causal inference while identifying prevention priorities. We acknowledge that our cross-sectional design cannot establish causal relationships or determine directionality of observed associations. However, our findings provide essential baseline data about factors potentially related to children's disclosure of technology-facilitated CSEA in contexts where such evidence has been largely absent.

B. I thank the authors for the effort put towards this suggestion. In my view, the discussion section feels more comprehensive and richer.

3. Originality and Significance

While the methodological approach itself is not novel, the focus on low- and middle-income countries is both original and highly significant. Scholars have increasingly called for greater attention to the Global South in research on child sexual abuse and prevention, yet the literature remains sparse. By centering these contexts, this paper addresses a pressing gap and provides insights that are highly relevant across disciplines, including criminology, public health, sociology, and international development. Given this, I expect the article has strong potential to be widely cited and influential.

A. We thank the reviewer for this generous assessment. By documenting both the prevalence and disclosure barriers of technology-facilitated CSEA, we provide foundational data that can potentially inform prevention programming, and future research agendas in LMICs.

B. No further discussion needed.

4. Data and Methodology

The methods employed are appropriate for the research questions, and the data are presented with care. However, some of the figures are overly complex and may be difficult for the average Nature reader to interpret efficiently. For instance, I found Figure 3 somewhat challenging and required more time than expected to distill its main points. Similarly, Extended Figure 1 is dense and may limit its utility. While I do not have a ready alternative, I encourage the authors to consider whether the data could be presented in a clearer visual format that prioritizes accessibility for a broad readership.

A. We thank the reviewer for this constructive feedback. We have substantially revised Figure 3 and Extended Data Figure 1 to improve interpretability for a broad readership.

B. I went back to the original manuscript to remind myself what Figure 3 looked like. The revised Figure 3 is much better. By leaps and bounds. Nice work.

5. Appropriate Use of Statistics and Treatment of Uncertainties

The statistical approaches appear appropriate and meets expected standards. One point that warrants clarification is the authors' use of multiple imputation by chained equations (MICE) to address missing data. While this is a reasonable choice, the manuscript does not explain why this method was selected over alternatives. A brief rationale would help strengthen confidence in the robustness of the analytic approach.

A. We appreciate the reviewer's attention to the statistical rigor and have now expanded the Methods section to clarify our choice of MICE. We selected multiple imputation by chained equations (MICE) for three reasons. First, it flexibly accommodates the Disrupting Harm dataset's mix of continuous and categorical variables through sequential models. Second, it is a well-validated approach widely used in social science and public health (van Buuren, 2018). Third, it allows us to generate multiple imputed datasets (M = 30) and pool estimates using Rubin's rules, thereby propagating imputation uncertainty.

B. I thank the authors for this explanation. That approach seems justified to me.

6. Conclusions

The conclusions are well grounded in the analyses and are presented with appropriate caution. None appear overstated. The results align with prior research while also highlighting meaningful variation across countries, which adds an important dimension to the literature. Overall, the conclusions are robust, valid, and reliable.

A. We thank the reviewer for their assessment of our conclusions as cautious. In revising the manuscript, we have reviewed the discussion section carefully to make sure that all conclusions remain appropriately framed within the limits of the data (as detailed above in Point 2).

B. I thank the authors for this effort.

7. Suggested Improvements

Several areas could be strengthened in revision. First, the introduction and discussion would benefit from a more substantive treatment of primary prevention. The current framing positions detection as the first step in addressing CSA, and it is, when viewing this problem through a criminal justice lens. However, from a public health perspective, primary prevention is the more foundational step. The data presented here suggest insights into what prevention might look like, and further exploration of this theme would add significant value.

A. We agree with the reviewer that our original framing leaned too heavily on detection within a criminal justice lens and underplayed prevention as the foundational public-health priority. We have now restructured the manuscript such that disclosure evidence is explicitly positioned within a prevention framework.

B. I think the revisions made are effective. A prevention lens (as well as the children's rights perspective) to me offer a richer framework to understand response to sexual harm.

Second, the discussion of protective factors—such as sex education, digital skills, and knowledge of help-seeking resources—would benefit from greater clarity. Do the authors conceptualize these as individual-level factors within the social ecological model, or do they also operate at broader levels?

A. We thank the reviewer for this important question. Our original discussion did not clearly distinguish how the measured protective factors map onto the social–ecological model…We have revised the text to make this measurement conceptualisation more evident and note that effective prevention and response typically require multilevel approaches (Page 26-27, Lines 442-445; Page 42, Lines 723-726).

B. I thank the authors for these revisions.

Third, the treatment of barriers to disclosure could be refined. Since only those who did not disclose were asked about barriers, important nuances may be missed, as individuals who did disclose may also have faced barriers but eventually overcame them. I imagine that this is a feature of the design of the survey, and not the analytic decision made by the authors. Still, noting this limitation explicitly would improve transparency.

A. In this survey, only adolescents who did not disclose any technology-facilitated CSEA were asked about reasons for non-disclosure. As a result, our estimates capture barriers among non-disclosers and do not capture barriers that children who did disclose may also have faced but overcame. We have added this as limitation in the manuscript to guide interpretation of

these findings (Page 45, Lines 778-780).

B. Understood. I thank the authors for this clarification and consideration in the limitations section.

Finally, although the manuscript acknowledges the importance of including rural and peri-urban contexts in research, the discussion does not meaningfully engage with findings from these settings. Incorporating this dimension would further strengthen the contribution.

A. We thank you for this important observation which has allowed us to strengthen the urbanization analysis. Specifically, we now report adjusted estimates of technology-facilitated CSEA prevalence across rural, peri-urban, and urban settings using a Bayesian logistic regression that adjusts for age and gender and allows effects to vary by country.

B. I thank the authors for this additional set of analyses and agree with the approach they took in presenting the findings as suggestive/in need of future research.

8. References

The manuscript cites relevant and appropriate literature throughout. The references are up to date and sufficiently comprehensive.

A. We appreciate this positive feedback.

B. No additional discussion needed.

9. Clarity and Context

The abstract is clear, accessible, and appropriately structured according to the journal's guidelines. It provides an accurate and concise summary of the study, highlighting its scope and contributions in a way that will be understandable to a wide audience. The introduction is similarly well framed and situates the study effectively within the existing literature.

Again, I would like to highlight the quality of writing in these sections.

A. We are grateful for the reviewer's kind comments on the clarity and quality of the writing in the abstract and introduction.

B. No additional discussion needed.

(Remarks on code availability)

Referee #2

(Remarks to the Author)
Thank you for your thoughtful and thorough responses to my comments and for the revisions to the manuscript. I appreciate the care you took to address my concerns within the limits of the available datasets.

Although some issues could not be fully resolved due to the nature of the data, I think these limitations are now clearly and appropriately acknowledged in the manuscript. Overall, I am satisfied with the revisions and believe they have strengthened the paper.

(Remarks on code availability)

Referee #3

(Remarks to the Author)
As was requested by the editors, I focused my review on the Bayesian modeling performed in this manuscript, and carefully considered the authors responses to my questions and recommendations in the first round of reviews. I have reviewed the revised manuscript, supplementary files, and the revised code posted in OSF.

I appreciate the authors attentiveness to my questions, and believe the revisions resolve all of the queries that I had raised. At this point I have no further recommendations for refining the model parameterization or presentation of the results.

(Remarks on code availability)
The code and README provided in the OSF is well documented and should be a good resource for those interested in understanding how the results were produced. There are some points where odd object names are used as headers (e.g., "LOO Victim" in the CSEA modeling script). These are accompanied by well documented write ups, but they did produce a

temporary *squint* as I worked through the sections.

Referee #4

(Remarks to the Author)
The authors were very responsive to reviewers' comments. All of my comments were appropriately addressed and I have no further concerns. I also appreciated the authors' inclusion of a dual denominator analysis in the revised manuscript.

(Remarks on code availability)

Open Access This Peer Review File is licensed under a Creative Commons Attribution 4.0 International License, which permits use, sharing, adaptation, distribution and reproduction in any medium or format, as long as you give appropriate credit to the original author(s) and the source, provide a link to the Creative Commons license, and indicate if changes were made.
In cases where reviewers are anonymous, credit should be given to 'Anonymous Referee' and the source.
The images or other third party material in this Peer Review File are included in the article's Creative Commons license, unless indicated otherwise in a credit line to the material. If material is not included in the article's Creative Commons license and your intended use is not permitted by statutory regulation or exceeds the permitted use, you will need to obtain permission directly from the copyright holder.
To view a copy of this license, visit https://creativecommons.org/licenses/by/4.0/

16 November 2025

Reviewer Response Letter

Dear Dr. Villamizar Santamaría,

Thank you very much for sending us the reviews for our manuscript *Technology-facilitated child sexual exploitation and abuse in Eastern and Southern Africa and Southeast Asia*. We are grateful to you and to the reviewers for the thoughtful and constructive feedback provided. We have now revised the manuscript substantially.

Editor Response

Your manuscript, "Technology-facilitated child sexual exploitation and abuse in Eastern and Southern Africa and Southeast Asia", has now been seen by four referees. You will see from their comments below that while they find your work of interest, some important points are raised. We are interested in the possibility of publishing your study in Nature, but would like to consider your response to these concerns in the form of a revised manuscript before we make a final decision on publication. We therefore invite you to revise your manuscript taking into account the following points:

Editor, point 1: *The methodological clarifications asked by all the reviewers, especially in relation to the denominators (R2, R4) and the many questions posed by R3.*

We have carefully addressed all methodological questions raised by the reviewers, particularly those concerning denominators and model specifications. Following the recommendations of Reviewers 2 and 4, we now dual-report prevalence using (a) prevalence among internet-using children aged 12–17 from the Disrupting Harm child survey (design-weighted) and (b) overall prevalence among all children aged 12–17 derived from the Disrupting Harm household survey (detailed in R2, Point 2). We propagate uncertainty in the combined estimates, strengthen both prevalence and disclosure measurement and provide additional analytical and methodological clarification in response to Reviewer 3's modelling queries (see R2 Points 2-7; R4 Point 10-13; R3 Points 1-6).

In addition, we contextualise the location of perpetration, whether CSEA was experienced online, in person, or in some other way, as this directly affects the scale and interpretation of prevalence estimates (e.g., estimates restricted to technology-facilitated CSEA are substantially lower than those for any CSEA among internet-using children). More broadly, the Methods section, Supplementary Materials, and Extended Data Figures 1 to 4 have been revised to reflect all methodological clarifications, and detailed responses to each reviewer's queries are provided in our point-by-point replies below.

Editor, point 2*:* *A more robust Introduction and Discussion that engages with recent literature (as suggested by R4) but that also responds to the changes in the analytical strategies requested (R1, R2, R4), and also in relation to the question about prevention from criminology and from public health.*

We have substantially revised the Introduction (see R1, Point 7) and Discussion (see R1, Point 2) to engage more fully with recent public health and criminology scholarship, and situate the study within a children's rights–based prevention framework. In line with the updated analytical strategies, we now clarify how disclosure functions within secondary and tertiary prevention and link observed disclosure and non-disclosure patterns to implications for prevention and child protection responses.

Editor, point 3: *A streamlined version of the figures to avoid confusions (R1, R2, R4).*

We streamlined key figures to improve accessibility. Figure 3 and Extended Data Figure 1 (detailed in R1, Point, 4) have been reformatted to reduce visual density and guide interpretation more directly (e.g., separating pooled and stratified estimates, simplifying colour encoding). We have added new Extended Data figures (please see R2, Point 2) to complement the prevalence estimates and have standardised all Supplementary figures for consistency.

Editor, point 4: *The rest of the reviewers' comments.*

We addressed all additional suggestions and questions raised by the reviewers and provide full responses below. All page and line number references in this response letter correspond to the tracked changes version of the manuscript (with inline markup).

Reviewer 1

Your manuscript, "Technology-facilitated child sexual exploitation and abuse in Eastern and Southern Africa and Thank you for the opportunity to review, "Technology-facilitated child sexual exploitation and abuse in Eastern and Southern Africa and Southeast Asia." Below, I have detailed comments related to this review, as provided by the guideline for reviewers. I am happy to provide any clarification on points listed below.

Reviewer 1, Point 1: *Key Results: One of the most valuable contributions of this manuscript is its focus on low- and middle-income countries, an area where empirical research on technology-facilitated child sexual abuse (CSA) remains particularly limited. By situating the study in these contexts, the authors significantly expand the global scope of scholarship on CSA. Similarly, the attention to technology-facilitated CSA is important, as this remains an underdeveloped area of research. I found the authors' harm-focused framing—particularly their observation that "although not every online risk leads to harm, adopting a harm focused framing is crucial to fully recognising the severity of children's experiences, and safeguarding their rights"—to be a powerful and persuasive approach that directly addresses common criticisms of being "too sensitive" about behaviours such as online harassment. Methodologically, the approach is well described and appropriate, and the paper is written in an engaging, accessible style. Indeed, the opening sections are so well framed that I could imagine using them as an example for my graduate students.*

We are grateful to the reviewer for their generous comments on the framing, methodological clarity, and accessibility of the manuscript.

Reviewer 1, Point 2: *Validity: I did not identify any flaws that would prohibit publication of this manuscript. The methods appear sound and the analyses are appropriately applied. My main concern lies with the underdeveloped discussion section, which would benefit from further elaboration before publication. Expanding this section would allow the authors to highlight the broader implications of their findings and strengthen the overall contribution of the paper.*

We appreciate the reviewer's feedback on the methodological rigor of our study. The reviewer raised important concerns about the underdeveloped discussion section, and we agree these improvements will significantly strengthen the manuscript's impact.

To frame our revisions, we have reorganised our Introduction (see Point 7 below) around a children's rights and public health prevention framework that distinguishes primary prevention (prevention before abuse occurs), secondary prevention priorities (child protection and detection), and early tertiary priorities (response and recovery). To address the Reviewer 1's concerns, we have substantially revised the Discussion section by: (a) organising findings within this three-tier framework; (b) explicitly linking empirical patterns to actionable strategies at individual, family, cultural, and system levels; (c) proposing concrete future research directions; and (d) maintaining appropriate epistemic caution about causal inference while identifying prevention priorities. We acknowledge that our cross-sectional design cannot establish causal relationships or determine directionality of observed associations. However, our findings provide essential baseline data about factors potentially related to children's disclosure of technology-facilitated CSEA in contexts where such evidence has been largely absent. The key changes are as below:

1. Age patterns inform prevention timing: We now explicitly discuss how age-graded patterns in our data point to critical periods for prevention beginning in early childhood and sustained through late adolescence. (Page 38-39, Line 628-639).

> **Discussion**: "We find that although older children were more likely to experience technology-facilitated CSEA, they were less likely to disclose such experiences. As the survey captured only past year experiences, cumulative exposure (particularly among older adolescents) is likely underestimated. These findings highlight the need for prevention efforts that begin in early childhood and are sustained through late adolescence, adapting to children's evolving developmental contexts. Both qualitative and longitudinal data are needed to understand how developmental trajectories influence vulnerabilities, the likelihood of disclosure over time and how decisions around disclosures are shaped by children's broader life circumstances."

2. Gender patterns and gender-responsive strategies. We have expanded our treatment of gender throughout the Discussion. Although overall exposure rates did not differ by gender in our data, we discuss how similar prevalence may still mask contextual differences in risk, experience, and consequences (Page 39, Line 642-648)

"We found no gender differences in exposure rates, though this may mask important differences in context and consequences. Emerging evidence from the Disrupting Harm project[10] indicates that societal expectations shape how perpetrators target children, how children interpret their experiences, and the responses they receive. Prevention strategies must therefore account for gendered dimensions of risk and response, even when prevalence rates appear similar[66]. If internet access differs by gender (e.g., in many LMICs), absolute exposure numbers may vary substantially, thus warranting gender-disaggregated monitoring and support."

3. Barriers to disclosure and gaps in primary prevention infrastructure. We expand on implications of the findings for knowing where to go or who to tell in the most common barriers, highlighting gaps in reporting pathways, accessible services, and trust in formal systems (Page 39-40, Line 655-662).

"“Not knowing where to go or who to tell" was the most common barrier to disclosure, suggesting gaps in both awareness of formal channels and access to trusted adults. Although basic information on where to report sexual abuse should be provided to all children from an early age (e.g., schools, health systems), formal reporting channels must be made more accessible and responsive to encourage disclosure[68]. Addressing other barriers identified in this study, including worries about not being believed, self-blame, fear of repercussions for the child or perpetrator, concerns about confidentiality, and limited awareness of reporting options, will also be crucial for ensuring an effective child protection response."

4. Informal disclosure to peers and implications for system design. We discuss the high rates of informal disclosure to peers and the need for developmentally appropriate, light-touch peer-support guidance. We also stress on strengthening of formal systems, so the burden does not fall on children, families, or civil society (Page 40, Line 663-669).

"Friends were frequently the first port of call, highlighting young people's roles in supporting their peers. While children should not bear the responsibility of child protection professionals, they may benefit from guidance that helps them support peers by connecting them to appropriate services or taking actions that minimise subsequent short-term harm. However, the burden of detection and response should not rest on children, families, or civil society alone[69]."

5. Modifiable protective factors. We identify two potentially modifiable protective correlates: (a) enabling parental mediation and open communication, and (b) children's knowledge of where to seek help. We discuss these as plausible, low-risk candidates for upstream prevention, while noting that our cross-sectional design cannot establish directionality (Page 40-41, Line 675-687).

"First, enabling parental mediation was positively associated with disclosure. Safe family environments and open conversations about online safety may facilitate children's willingness to report incidents without fear of judgment or shame[70]. Such family climates may foster trust

that enables disclosure after harm occurs, or regular parent-child engagement may create opportunities for proactive communication that make children feel comfortable seeking help. However, our data cannot disentangle these pathways or establish directionality. Second, consistent with not knowing where to seek help being the most common barrier to disclosure, knowing where to seek help was associated with higher disclosure, particularly through informal channels. Developmentally appropriate help-seeking education may be a low-risk, scalable prevention target, however, this association was sensitive to model regularisation, suggesting that relationship may vary across countries."

6. Cultural-level levers: gender norms and stigma. Building on extensive violence preventon literature, we expand on how inequitable gender norms and stigma surrounding sexual abuse must also be addressed. Social-norms interventions need to be considered alongside family and system-level strategies to reduce shame and increase the likelihood of help-seeking after harm (Page 41-42, Line 706-711).

"Addressing harmful gender norms is widely recognised as critical for violence prevention and may be relevant for technology-facilitated harms, particularly as shame, or embarrassment around these conversations can prevent children from disclosing experiences of abuse altogether[60,72] . When these norms persist, perpetrators may exploit them to reduce the likelihood of being reported."

7. Synthesis of intervention priorities by prevention level. We conclude the Discussion with a reorganised, concrete synthesis of intervention implications across primary, secondary, and tertiary prevention levels (Page 42, Line 715-726).

"Our cross-sectional findings therefore identify plausible prevention and response levers[40] but remain hypothesis-generating and warrant prospective evaluation. They highlight intervention priorities across multiple prevention levels: providing age-appropriate information on where to seek help from early childhood; supporting caregivers to sustain open, enabling communication that strengthens protective relationships; addressing stigma around sexual topics; and improving accessibility and responsiveness of formal services to meet children's needs when harm occurs. As factors related to disclosure span interconnected domains (individual, family, cultural, and systemic), effective prevention and response will likely require coordinated, multi-level approaches, rather than isolated, child- or technology-level interventions[73]."

8. Future directions. We added a subsection to the Discussion that: (a) motivates targeted, context-specific studies, including participatory research with young people with lived experience across urban, peri-urban and rural settings; (b) proposes feasible designs suitable for LMICs to establish temporal ordering; and (c) prioritises investigation of platform-mediated disclosure and platform responses (Page 43, Line 729-743).

"These findings point to several important directions for future research. First, substantial heterogeneity observed between the 12 countries, combined with sensitivity to modelling techniques, highlights the need for culturally specific investigations into the mechanisms that shape disclosure. Participatory approaches that directly engage children with lived experience of technology-facilitated CSEA across rural, peri-urban and urban settings can generate region-specific insights that complement the population-level patterns identified[74]. Second, given the identification and retention challenges of traditional longitudinal cohorts in LMICs[75], prospective longitudinal or diary-based designs that follow children over time may be more feasible for establishing temporal precedence and determining whether associated factors prevent harm, facilitate disclosure, or both. Third, understanding platform-based disclosure mechanisms represents a critical priority[76]. Our analyses focus only on interpersonal disclosure pathways, but digital platforms can both facilitate and detect CSEA. Therefore, understanding how children use platform-specific reporting tools and how technology companies respond will provide a more complete account of help-seeking in digital environments."

Reviewer 1, Point 3: *Originality and Significance: While the methodological approach itself is not novel, the focus on low- and middle-income countries is both original and highly significant. Scholars have increasingly called for greater attention to the Global South in research on child sexual abuse and prevention, yet the literature remains sparse. By centering these contexts, this paper addresses a pressing gap and provides insights that are highly relevant across disciplines, including criminology, public health, sociology, and international development. Given this, I expect the article has strong potential to be widely cited and influential.*

We thank the reviewer for this generous assessment. By documenting both the prevalence and disclosure barriers of technology-facilitated CSEA, we provide foundational data that can potentially inform prevention programming, and future research agendas in LMICs.

Reviewer 1, Point 4: *Data and Methodology: The methods employed are appropriate for the research questions, and the data are presented with care. However, some of the figures are overly complex and may be difficult for the average Nature reader to interpret efficiently. For instance, I found Figure 3 somewhat challenging and required more time than expected to distill its main points. Similarly, Extended Figure 1 is dense and may limit its utility. While I do not have a ready alternative, I encourage the authors to consider whether the data could be presented in a clearer visual format that prioritizes accessibility for a broad readership.*

We thank the reviewer for this constructive feedback. We have substantially revised Figure 3 and Extended Data Figure 1 to improve interpretability for a broad readership.

<u>Figure 3:</u> The original figure presented barriers to disclosure as a heatmap displaying 13 barriers across 9 technology-facilitated CSEA types (presenting 117 data points in a single matrix). We acknowledge this format required considerable cognitive effort to extract meaningful patterns. The revised Figure

3 now adopts a two-panel hierarchical structure that guides readers through a clear interpretive pathway (Page 24-25, Lines 409-415):

- Panel (a) shows the overall frequency of each barrier among non-disclosed incidents (instance-level denominator), ordered from most to least common. Estimates are survey-weighted with 95% CIs using Wilson score intervals, which provide better coverage for proportions when sample sizes are modest or proportions are near boundaries (0%, 100%).
- Panel (b) displays barrier proportions stratified by the nine technology-facilitated CSEA experience types using faceted lollipop plots. For estimates with fewer than 10 observations or fewer than 3 positive responses, point estimates are shown but confidence intervals are suppressed due to insufficient precision. Supplementary Tables 46 to 47 provide tabular presentations of all barriers and CSEA type combinations with 95% CIs, preserving complete information about how barriers vary by CSEA type.

Brown, L. D., Cai, T. T., & DasGupta, A. (2001). Interval estimation for a binomial proportion. *Statistical Science*, 16(2), 101-133.

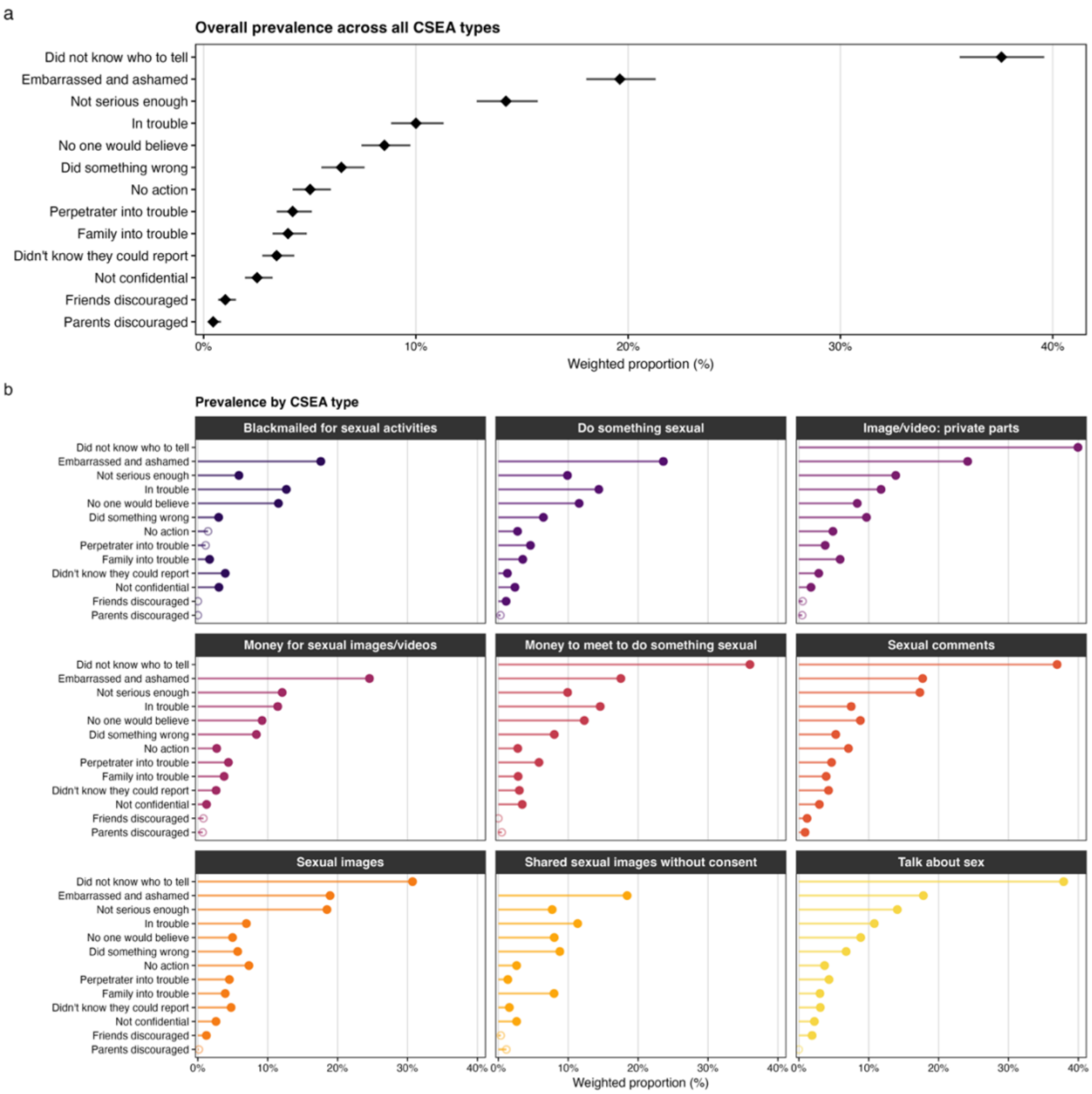

Figure 3: Barriers to disclosure of technology-facilitated CSEA (ages 12–17): Panel (a) shows overall barrier prevalence across all CSEA types among children who did not disclose their experiences. Panel (b) displays barriers stratified by each of the nine technology-facilitated CSEA types. Estimates are weighted proportions with 95% confidence intervals using Wilson score methods adjusted for unequal weighting. Wilson intervals provide better boundary behaviour than normal approximations when proportions approach 0% or 100%. In Panel (b), estimates with fewer than 10 observations or fewer than 3 positive responses are shown without confidence intervals (hollow points) due to insufficient precision; estimates with CI width exceeding 20% are marked with semi-transparent points. Full numeric results are provided in Supplementary Table 47.

<u>Old Extended figure 1:</u> To reduce density while maintaining transparency, we split the figure and adjusted the country-specific results as follows:

- *Main manuscript (new Figure 5):* The manuscript now includes a simplified Figure 5 that presents country-specific predictors of disclosure using a simplified visual grammar: points (posterior means) and 95% credible intervals from a Bayesian multilevel logistic model with random intercepts and slopes. Solid marks indicate effects where the 95% credible interval excludes zero. We have also updated text to reflect the results from this heterogeneity analysis (see Reviewer 4, Point 12 response) (Page 31, Lines 496-505; Figure 5, Page 31-33)
- *Extended Data Figure 3 (new figure):* We move channel-specific details to Extended Data Figure 3 (Page 83, Lines 1741-1750), contrasting formal (police, helpline, social worker, teacher) versus informal (family, friends, other adults) disclosure channels without overloading the main narrative.
- *Supplementary Table 31:* Full numerical estimates corresponding to both figures are reported in Supplementary Table 31 (Page 76-83), allowing interested readers to explore the channel-level results in depth.

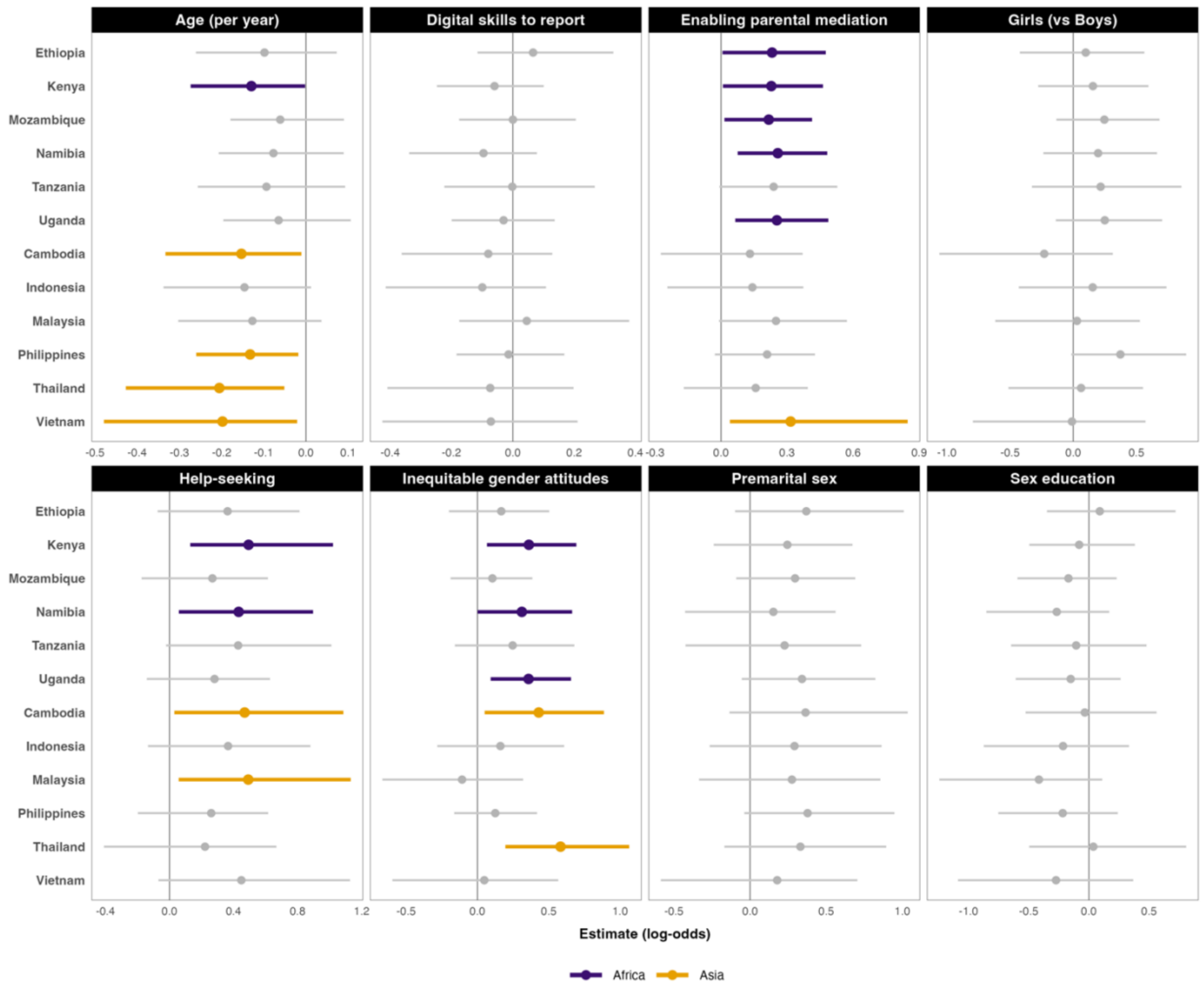

Figure 5: Country-specific parameter estimates for predictors of disclosure of technology-facilitated CSEA. Points show posterior means (log-odds of disclosure) from a

Bayesian multilevel logistic regression with random intercepts and random slopes; horizontal bars show two-sided 95% credible intervals. Purple indicates African countries (Ethiopia, Kenya, Mozambique, Namibia, Tanzania, Uganda); orange indicates Asian countries (Cambodia, Indonesia, Malaysia, Philippines, Thailand, Vietnam). Solid marks denote effects with 95% credible intervals excluding zero; faded marks denote effects with intervals including zero. The model includes all predictors simultaneously and accounts for country-level heterogeneity in baseline disclosure and predictors.

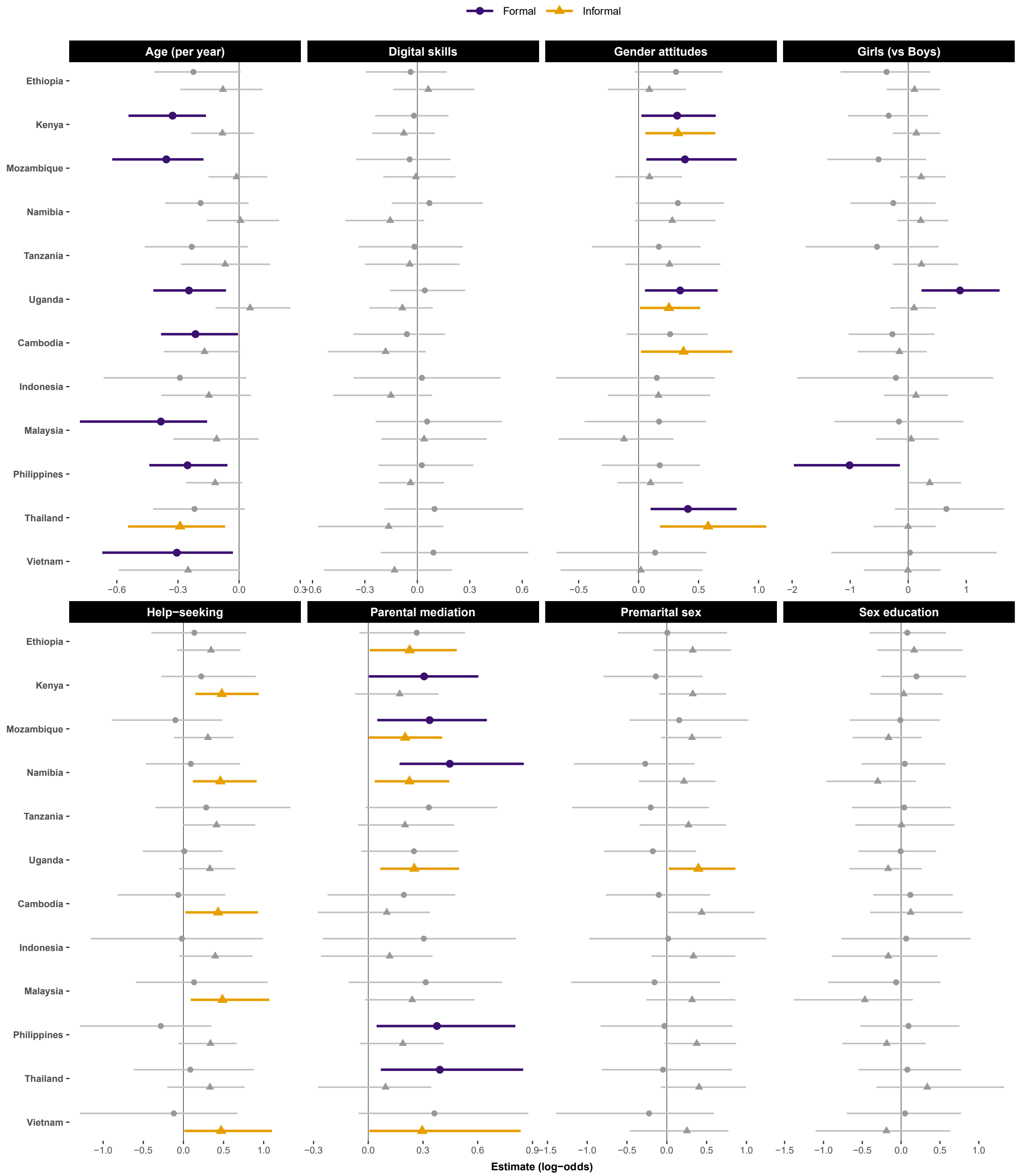

Extended Data Fig. 3: Country-specific associations between predictors and disclosure by channel type. Points show posterior means (log-odds) from Bayesian multilevel logistic models with random intercepts and slopes; horizontal bars show two-sided 95% credible intervals. Formal channels include police, helpline, social worker, and teacher; informal channels include family members, friends and other adults. Purple denotes formal

channels and orange denotes informal channels (shape and colour keys indicated in the panel legend). Faded grey points and intervals indicate effects whose 95% credible intervals include zero.). Countries are ordered by region (African countries above; Asian countries below). Each model adjusts for all predictors simultaneously and accounts for clustering at the country level.

Reviewer 1, Point 5: *Appropriate Use of Statistics and Treatment of Uncertainties: The statistical approaches appear appropriate and meets expected standards. One point that warrants clarification is the authors' use of multiple imputation by chained equations (MICE) to address missing data. While this is a reasonable choice, the manuscript does not explain why this method was selected over alternatives. A brief rationale would help strengthen confidence in the robustness of the analytic approach.*

We appreciate the reviewer's attention to the statistical rigor and have now expanded the Methods section to clarify our choice of MICE. We selected multiple imputation by chained equations (MICE) for three reasons. First, it flexibly accommodates the Disrupting Harm dataset's mix of continuous and categorical variables through sequential models. Second, it is a well-validated approach widely used in social science and public health (van Buuren, 2018). Third, it allows us to generate multiple imputed datasets (M = 30) and pool estimates using Rubin's rules, thereby propagating imputation uncertainty.

We considered alternative approaches but deemed them less appropriate. Listwise deletion would have substantially reduced sample sizes across our analyses. For example, in the demographic predictors for technology-facilitated CSEA analysis, listwise deletion would have reduced the sample from N=2,067 to N=1,149, 44% loss, or N = 2025 to 1105; 45% in weighted terms, substantially reducing statistical power. Full Bayesian imputation within brms was also considered; however, as summarised by Enders (2022), when models leverage the same assumptions and information, Bayesian estimation, and multiple imputation generally yield numerically similar results. Given this equivalence, we selected MICE for its computational efficiency with our complex multilevel models (random intercepts and slopes across 12 countries) and our team's familiarity with the approach. The key requirement was to make sure that the imputation model accurately captures the underlying distribution of the data. This rationale has been integrated into the Methods section (Page 61, Line 1160-1164), highlighting that our choice is grounded in statistical theory and appropriate for the complexity of our data.

Method: "All missing data for the independent variables (see Supplementary Table 12) were imputed using the Multiple Imputation by Chained Equations (MICE) method and the mice package in R[93]. MICE was used as it accommodates both continuous and categorical variables flexibly and is well suited to the complexity of the dataset. We ran 30 iterations of the analysis and pooled results across all datasets to ensure robust estimation."

Reference:
van Buuren, S. (2018). Flexible Imputation of Missing Data (2nd ed.). Chapman & Hall/CRC
Enders, C. K. (2022). Applied Missing Data Analysis (2nd ed.). Guilford Press.

Reviewer 1, Point 6: Conclusions: *The conclusions are well grounded in the analyses and are presented with appropriate caution. None appear overstated. The results align with prior research while also highlighting meaningful variation across countries, which adds an important dimension to the literature. Overall, the conclusions are robust, valid, and reliable.*

We thank the reviewer for their assessment of our conclusions as cautious. In revising the manuscript, we have reviewed the discussion section carefully to make sure that all conclusions remain appropriately framed within the limits of the data (as detailed above in Point 2).

Reviewer 1, Point 7: *Suggested Improvements. Several areas could be strengthened in revision. First, the introduction and discussion would benefit from a more substantive treatment of primary prevention. The current framing positions detection as the first step in addressing CSA, and it is, when viewing this problem through a criminal justice lens. However, from a public health perspective, primary prevention is the more foundational step. The data presented here suggest insights into what prevention might look like, and further exploration of this theme would add significant value.*

We agree with the reviewer that our original framing leaned too heavily on detection within a criminal-justice lens and underplayed prevention as the foundational public-health priority. We have now restructured the manuscript such that disclosure evidence is explicitly positioned within a prevention framework.

We first reorganised the Introduction to contextualise the global burden of technology-facilitated CSEA and the need for evidence-informed prevention, before turning to barriers and disclosure (Page 7, Lines 161-174).

> "Research on technology-facilitated CSEA has expanded substantially in HICs[24], yet has focused predominantly on prevalence[25–27]. Nationally representative surveys reveal wide variation in prevalence estimates due to definitional[18] and methodological choices such as whether peer or adult solicitation is counted[28], the recall period used (past year versus lifetime)[24], and sampling denominators (all children versus internet users only)[29]. For instance, 17.7% of Australian 16–24-year-olds reported adult online sexual solicitation before age 18[30] , while U.S. estimates indicates adding online abuse items can increase overall CSA prevalence estimates from 13.5% to 21.7% depending on how online harms are opertionalised[25]. A recent systematic review and meta-analysis estimates that roughly one in 12 children globally has experienced online CSEA (pooled past-year prevalence 8.1%)[24]. While such studies serve critical epidemiological functions such as mapping population burden, guiding resources, and enabling surveillance[31], prevalence data alone cannot reveal whether children seek help after

harm. Understanding disclosure pathways is therefore equally critical for effective prevention and response[32]."

We then expand on the limited evidence base on disclosure of technology-facilitated CSEA, the predominance of data from high-income and criminal-justice settings, and the lack of comparable evidence from LMICs, arguing that cross-cultural research is needed (Page 8, Lines 175-195).

"To date, research on disclosure of technology-facilitated CSEA remains limited[33,34]. Where it exists, it often draws on forensic, child protection or criminal-justice settings[35–39], which capture victims who have already navigated formal reporting systems. Recent qualitative studies have identified barriers to disclosure such as self-blame, shame, and lack of trust[33], yet empirical investigations into facilitators or enablers of disclosure in online environments has been limited[32,40]. Evidence from broader child sexual abuse research indicates that disclosure marks a pivotal step for identifying and stopping offline sexual abuse[32,41,42], facilitating recovery[43], and reducing long-term mental health impacts[42]. Children are more likely to confide in peers or siblings and rarely use formal reporting channels such as police, helplines or teachers[44,45]. Yet little is known about disclosure of technology-facilitated CSEA in LMICs[46], where formal and informal support systems may differ substantially from those in HICs. Cross-cultural research on prevalence and disclosure is therefore needed to inform timely prevention support and protection."

Together, these revisions create a clearer progression: we begin with what harm exists and at what scale (prevalence data), then move to whether and how children seek help (disclosure patterns), and finally to what these patterns reveal about gaps in prevention systems.

Next, we explicitly situate disclosure within a three-tier public health model and child-rights framework. We adopt a prevention-first framing because both prevalence and disclosure data, including barriers and predictors are crucial for timely prevention and response. Prevalence data shows the burden of harm but cannot guide prevention without understanding who can access help, what prevents help-seeking, and which factors are related to disclosure. Importantly, our research examines not only barriers, but also predictors of disclosure as potential targets for upstream prevention efforts. Such framing ensures that our estimates pinpoint priority areas for prevention rather than detection alone (Page 9-10, Lines 203-235).

"Guided by child-rights[47] and public-health frameworks[48–50], we examine disclosure patterns and associated factors through a socio-ecological lens[51–53]. The UN Committee on the Rights of the Child's General Comment No. 25 affirms that protection in digital environments depends on accessible, child-friendly pathways to seek help[47]. From a public-health perspective, primary prevention (preventing abuse before it occurs) is the foundational priority, supported by secondary (early detection and intervention) and tertiary (treatment and rehabilitation) responses[50]. These tiers are interdependent as disclosure operates primarily within secondary and tertiary prevention[48] yet patterns of disclosure and non-disclosure can highlight how systems prevent, detect, and respond to harm. When children cannot disclose, whether due to lack of awareness, inaccessible systems, or lack of trusted adults, these barriers may signal gaps in primary prevention infrastructure[50]. Conversely, when disclosure does

occur, timely intervention and trauma-informed care become possible. Understanding how, to whom, and under what conditions children disclose technology-facilitated CSEA can therefore guide more targeted interventions, strengthen support pathways, and ultimately reduce subsequent harms to children[32].

In this cross-sectional study, we address two questions: (1) What are the prevalence and disclosure rates of technology-facilitated CSEA in these contexts? and (2) Which demographic, family, cultural, and protective factors are associated with disclosure? (see Methods). This study thus provides researchers, policymakers, child-protection and education systems, and law enforcement with population-level data on children living in LMICs who both are highly vulnerable and systematically underrepresented on the global scientific stage."

Finally, we expanded the Discussion to draw out prevention and response implications from our results (as detailed in Point 2). In revising this section, we have been careful not to frame disclosure research as a replacement for primary prevention or as sufficient on its own for prevention, and we do not claim that our cross-sectional study directly tests primary prevention interventions. Discussion now offers a more clearly structured, prevention-oriented synthesis that acknowledges the limits of our design. By linking observed barriers and predictors of disclosure to potential testable targets, we aim to provide baseline evidence on which future intervention research can build. Our intention is to position the prevalence and disclosure findings as foundational inputs into prevention-oriented work, and we hope this addresses the reviewer's concern about how the implications are framed.

Reviewer 1, Point 8: *Second, the discussion of protective factors—such as sex education, digital skills, and knowledge of help-seeking resources—would benefit from greater clarity. Do the authors conceptualize these as individual-level factors within the social ecological model, or do they also operate at broader levels?*

We thank the reviewer for this important question. Our original discussion did not clearly distinguish how the measured protective factors map onto the social–ecological model.

Although we measured them at the child level (e.g., whether a given child had received sex education, possessed digital skills or knew where to seek help), we recognise that these protective factors operate across multiple ecological levels. At the individual level, such capacities may help children recognise risk and seek assistance. At the family/community level, safety education and digital literacy are typically nurtured by caregivers, peers, schools, and community programmes (e.g., comprehensive sex education). At the cultural level, safeguarding policies, service availability, legal protections, and gender norms condition whether individual capacities translate into action (consistent with ecological syntheses of correlates of victimisation, e.g., Sabri et al., 2013). In this study we, however, only examined gender norms at this latter level. Analytically, we treat these variables as observed correlates rather than interventions. We have revised the text to make this measurement conceptualisation more evident and note that effective prevention and response typically require multilevel approaches (Page 26-27, Lines 442-445; Page 42, Lines 723-726).

Disclosure section: "Operationally, we analyse these as child-level indicators of protective factors that span the three levels of our social-ecological model: acquired and enacted by children (individual), reinforced by caregivers, peers and schools (family/community), and enabled or constrained by curricula, safeguarding policies, and legal frameworks (cultural)."

Discussion: "As factors related to disclosure span interconnected domains (individual, family, cultural, and systemic), effective prevention and response will likely require coordinated, multi-level approaches, rather than isolated child- or technology-level interventions[73]."

References: What works to prevent online violence against children? executive summary. https://www.who.int/publications-detail-redirect/9789240062085.

Sabri, B., Hong, J. S., Campbell, J. C. & Cho, H. Understanding Children and Adolescents' Victimizations at Multiple Levels: An Ecological Review of the Literature. *J. Soc. Serv. Res.* **39**, 322–334 (2013).

Reviewer 1, Point 9: *Third, the treatment of barriers to disclosure could be refined. Since only those who did not disclose were asked about barriers, important nuances may be missed, as individuals who did disclose may also have faced barriers but eventually overcame them. I imagine that this is a feature of the design of the survey, and not the analytic decision made by the authors. Still, noting this limitation explicitly would improve transparency.*

In this survey, only adolescents who did not disclose any technology-facilitated CSEA were asked about reasons for non-disclosure. As a result, our estimates capture barriers among non-disclosers and do not capture barriers that children who did disclose may also have faced but overcame. We have added this as limitation in the manuscript to guide interpretation of these findings (Page 45, Lines 778-780).

Limitation: "Because questions about barriers were administered only to non-disclosers, findings may underrepresent barriers overcome by those who eventually disclosed."

Reviewer 1, Point 10: *Finally, although the manuscript acknowledges the importance of including rural and peri-urban contexts in research, the discussion does not meaningfully engage with findings from these settings. Incorporating this dimension would further strengthen the contribution.*

We thank you for this important observation which has allowed us to strengthen the urbanisation analysis. Specifically, we now report adjusted estimates of technology-facilitated CSEA prevalence across rural, peri-urban, and urban settings using a Bayesian logistic regression that adjusts for age and gender and allows effects to vary by country.

As shown in Supplementary Figure 22 (and corresponding Supplementary Table 9), adjusted prevalence is highest in peri-urban, followed by urban, then rural settings. Rural versus urban contrasts

are supported at the 95% credible intervals, whereas peri-urban versus urban is inconclusive. Conceptually, peri-urban areas often combine urban features, such as higher population density, better infrastructure, and more institutional services, with characteristics more common to rural settings, making the relationship between urbanisation and technology-facilitated CSEA complex. We speculate that factors such as internet access, digital literacy and local infrastructure intersect to shape risk, although relying solely on these cross-sectional contrasts may overemphasise these relationships.

Because these adjusted differences are modest in absolute terms, we refrain from strong interpretation in the Discussion to avoid presenting this as a definitive pattern. Instead, we frame this as a direction for future work, particularly participatory research engaging children in rural, peri-urban, and urban contexts to develop region-specific insights (see Page 18, Lines 341-348; Page 38/43, Lines 625-628/ 732-735; Page 60 Lines 1118-1124). Understanding how urbanisation intersects with technology access, social structures and help-seeking pathways will require qualitative and longitudinal approaches that our cross-sectional survey cannot provide. This revision incorporates the urbanisation dimension without overclaiming from exploratory patterns that warrant further investigation.

> **Results:** "Prevalence of technology-facilitated CSEA also varied by degree of urbanisation (urban, peri-urban, rural) (Supplementary Figure 22; Table 9 and 40). After adjusting for gender and age, children in peri-urban settings reported the highest prevalence, followed by urban and rural settings. Rural children had lower reported prevalence than peri-urban children (−4.2 percentage points, AME = -0.042, 95% CI = [−7.3, −1.7]), while the difference between urban and peri-urban settings was inconclusive (−1.9 percentage points, AME = -0.019, 95% CI = [−4.9, 0.6]). Urban children had higher reported prevalence than rural children (2.3 percentage points, AME = 0.023, 95% CI = [0.8, 4.0]).

Discussion: "By analysing data from children living in Eastern and Southern Africa and South-East Asia, including peri-urban and rural populations, often underrepresented in behavioural research… Participatory approaches that directly engage children with lived experience of technology-facilitated CSEA across rural, peri-urban and urban settings can generate region-specific insights that complement the population-level patterns identified[74]."

Methods: "We further examined whether harms differed across urban, peri-urban, and rural settings, while appropriately accounting for gender, age, and cross-country heterogeneity. This model preserves an additive fixed effect for degree of urbanisation (i.e., the urban, peri-urban and rural contrasts are adjusted for gender and age), while allowing gender and age effects (and their interaction) to vary by country as random effects. We chose not to model degree of urbanisation with random effects because each country contributes only three categories, making random-effects estimation unstable and difficult to interpret."

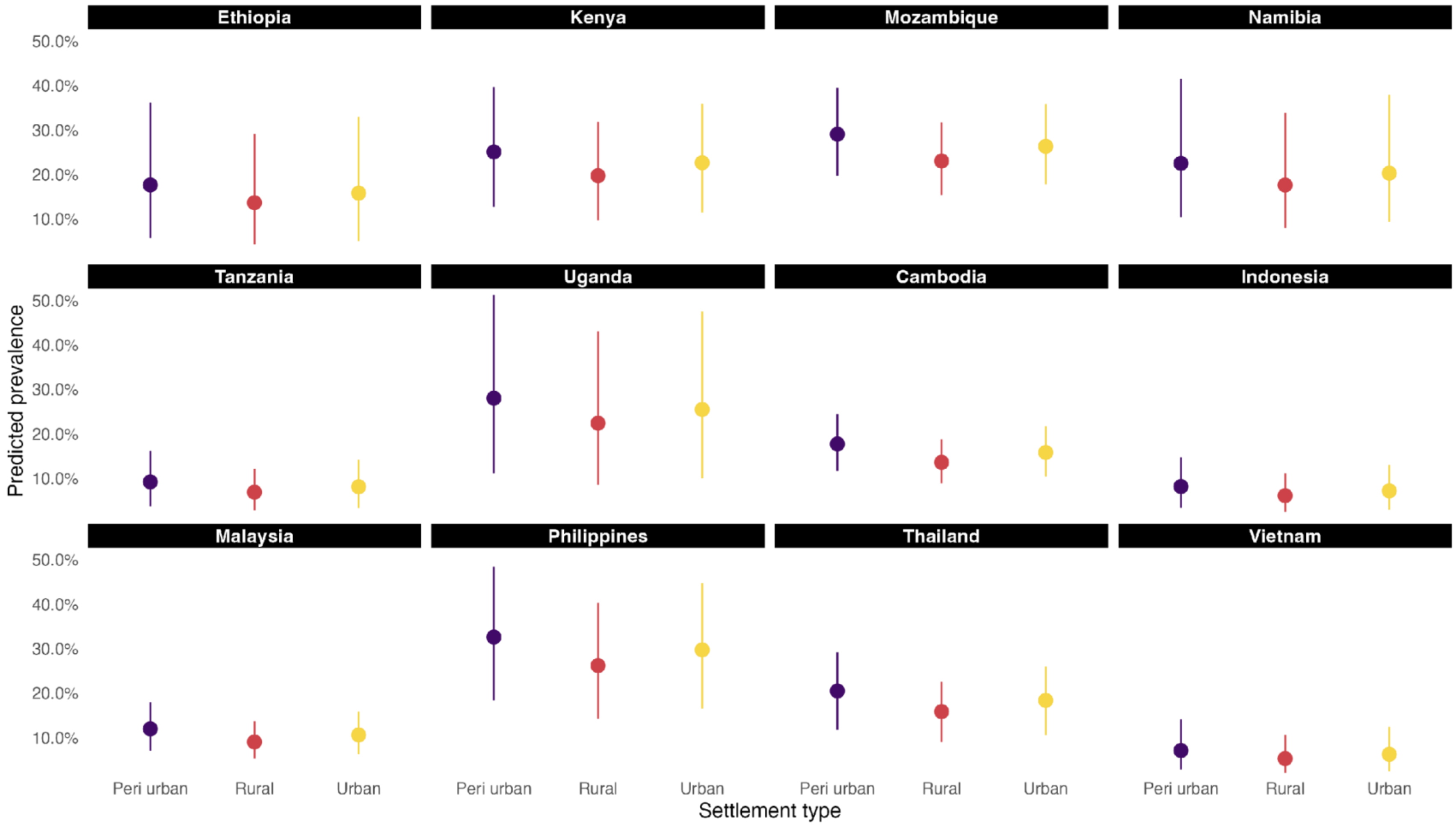

Supplementary Figure 22: Predicted prevalence of technology-facilitated CSEA by degree of urbanisation (Rural, Peri-urban, Urban), with 95% credible intervals. Values are posterior means from the survey-weighted Bayesian regression, marginalised over covariates in the fitted model (e.g. age, gender). Within each country, estimates are contrasted across rural, peri-urban and urban areas; panels correspond to countries.

Country	Degree of urbanisation	Mean %	95% CI
Cambodia	Rural	13.4%	[11.3%, 15.7%]
	Urban	15.6%	[13.2%, 18.4%]
	Peri-urban	17.5%	[14.5%, 21.1%]
Ethiopia	Rural	13.7%	[11.4%, 16.3%]
	Urban	16.0%	[13.2%, 18.9%]
	Peri-urban	17.9%	[14.2%, 21.5%]
Indonesia	Rural	6.1%	[4.6%, 7.6%]
	Urban	7.2%	[5.5%, 9.0%]
	Peri-urban	8.1%	[6.1%, 10.4%]
Kenya	Rural	20.3%	[17.8%, 22.9%]
	Urban	23.4%	[20.6%, 26.6%]
	Peri-urban	25.9%	[22.0%, 30.4%]
Malaysia	Rural	9.1%	[7.3%, 10.9%]
	Urban	10.7%	[8.8%, 12.9%]
	Peri-urban	12.1%	[9.7%, 14.7%]
Mozambique	Rural	23.4%	[20.8%, 26.2%]
	Urban	26.8%	[23.7%, 29.7%]
	Peri-urban	29.6%	[25.3%, 34.0%]
Namibia	Rural	18.0%	[15.7%, 20.6%]
	Urban	20.8%	[18.2%, 23.6%]
	Peri-urban	23.2%	[19.4%, 27.3%]
Philippines	Rural	27.0%	[24.0%, 29.9%]
	Urban	30.7%	[27.3%, 34.0%]
	Peri-urban	33.7%	[29.0%, 38.4%]
	Rural	6.9%	[5.3%, 8.5%]

**Tanzania**	Urban	8.2%	[6.4%, 10.0%]
	Peri-urban	9.2%	[7.1%, 11.9%]
**Thailand**	Rural	15.9%	[13.6%, 18.2%]
	Urban	18.5%	[15.7%, 21.2%]
	Peri-urban	20.7%	[17.0%, 24.4%]
**Uganda**	Rural	23.1%	[20.5%, 26.1%]
	Urban	26.4%	[23.1%, 29.7%]
	Peri-urban	29.1%	[24.5%, 33.7%]
**Vietnam**	Rural	5.3%	[4.1%, 6.7%]
	Urban	6.2%	[4.7%, 7.9%]
	Peri-urban	7.1%	[5.3%, 9.3%]

Supplementary Table 9: Predicted prevalence (%) of technology-facilitated CSEA by degree of urbanisation (rural, peri-urban, urban), with 95% Bayesian credible intervals. Values are posterior means from the survey-weighted Bayesian regression, marginalised over covariates in the fitted model (e.g. age, gender). Each row shows the posterior mean and 95% credible interval for a given country and degree of urbanisation.

Reviewer 1, Point 11: *References: The manuscript cites relevant and appropriate literature throughout. The references are up to date and sufficiently comprehensive.*

We appreciate this positive feedback.

Reviewer 1, Point 12: *Clarity and Context: The abstract is clear, accessible, and appropriately structured according to the journal's guidelines. It provides an accurate and concise summary of the study, highlighting its scope and contributions in a way that will be understandable to a wide audience. The introduction is similarly well framed and situates the study effectively within the existing literature. Again, I would like to highlight the quality of writing in these sections.*

We are grateful for the reviewer's kind comments on the clarity and quality of the writing in the abstract and introduction.

Reviewer 1, Point 13: *Referee #1 (Remarks on code availability): No--I am currently abroad and working on an old laptop, so I do not have the capacity to check the code. So I defer to the other reviewers and the editor here.*

No comment.

Reviewer 2

Thank you for the opportunity to review, "Technology-facilitated child sexual exploitation and abuse in Eastern and Southern Africa and Southeast Asia." Below, I have detailed comments related to this review, as provided by the guideline The study examined children's self-reported experiences of technology-facilitated CSEA across 12 countries. The topic is of great interest, and the paper is well-written, but I have several concerns and comments for the authors to consider.

Reviewer 2, Point 1: *The authors should clearly specify the reference time period used to measure the prevalence of technology-facilitated CSEA—was it the past 6 months, past year, or past two years?*

We appreciate this helpful clarification. The manuscript already specifies that prevalence estimates refer to children's experiences within the past year during the 2020-2021 survey period. To avoid any ambiguity, we have revised the description in methods to restate this point more clearly as well as highlighted this at several points in the manuscript (Page 2, Line 32-34; Page 5, Line 114-119; Page 49-50, Line 887-890).

> "Our findings show that one in six digitally connected children in these countries experienced one or more forms of technology-facilitated CSEA in 2020-2021, scaled to over 10 million children."
>
> "The Disrupting Harm project is a collaboration between UNICEF Innocenti Global Office of Research and Foresight, ECPAT International, and INTERPOL, funded by the Safe Online initiative, which collected survey data from nationally representative samples of 11,912 internet-using children aged 12 to 17 years across 12 countries (Ethiopia, Kenya, Mozambique, Namibia, Tanzania, Uganda, Cambodia, Indonesia, Malaysia, the Philippines, Thailand, and Vietnam)[17] between 2020 and 2021."
>
> "Prevalence of CSEA among internet-using was measured using a composite variable capturing whether a child had experienced any form of technology facilitated CSEA within the past year, as reported during the 2020-2021 survey period."

Reviewer 2, Point 2: *When estimating the prevalence of technology-facilitated CSEA, the authors used only internet-using children as the denominator. It would be helpful for the authors to justify this choice. In my view, both approaches—using all children (e.g., all children aged 12–17 in the population) and only internet-using children—provide valuable but different insights. Prevalence among all children reflects the overall population burden, while prevalence among internet users more accurately captures individual-level risk. The authors may wish to consider a dual reporting approach that presents prevalence estimates for both the general child population and internet users.*

This is a very important methodological suggestion. To address this comment we made a number of changes to implement the dual reporting approach throughout the manuscript.

Prevalence among internet-using children (individual-level harms): We estimate prevalence using data from the Disrupting Harm child survey (12 countries; n = 11,912), with our primary outcome defined as the proportion of internet-using children aged 12–17 who experienced technology-facilitated CSEA through social media or gaming platforms in 2020–2021 (Table 1, column 1). Estimates are design-weighted to represent the national population of internet-using children in each country and directly answer: *What proportion of internet-using children experience at least one form of technology-facilitated CSEA?*

Overall prevalence among all children (population burden): In response to the reviewer's suggestion, we now also report prevalence among all children aged 12–17 (Table 1, column 3) by combining: (a) the proportion of children who use the internet, and (b) the prevalence of technology-facilitated CSEA among internet-using children. This addresses the complementary question: *What proportion of the total child population experiences technology-facilitated CSEA?* Internet use is derived from the DH household survey (~1,500–10,000 households per country), in which caregivers report whether 12–17-year-olds in the household use the internet via any device or location. For example, in Cambodia, 81.0% of children aged 12–17 use the internet and 14.7% of internet-using children experienced technology-facilitated CSEA, yielding an overall prevalence of 11.9% among all children. We report 95% confidence intervals for all estimates.

Because technology-facilitated harms require online exposure, children who do not use the internet are treated as unexposed to these specific harms (while acknowledging they may experience other forms of abuse). Population-level estimates in the manuscript thus focus on technology-facilitated harms because in-person abuse rates cannot be validly extrapolated to non-internet users. For completeness, we also report the prevalence of any CSEA (irrespective of where it occurred), providing context for the relative contribution of technology-facilitated abuse to children's overall burden of sexual victimisation. Approximately one out of three internet-using children experienced at least one form of CSEA (online or in-person or some other way; Supplementary Table 3).

Major additions and validation: We added dual-denominator results to the main manuscript in Table 1 (Page 15–16), which now includes 95% CIs that propagate sampling error in all components via Monte Carlo simulation on the logit scale (5,000 draws per country). The Methods section now clearly delineates data sources and calculations, describes design-weighted child-survey estimates, and details the household module used to ascertain whether children aged 12–17 use digital devices or the internet (any device/location).

To validate our internet exposure estimates, we compare DH-based exposure rates with International Telecommunication Union (ITU) youth internet indicators, while noting differences in age bands and reference periods (Supplementary Section 2.1). We added Supplementary Figures 11 and 13 (DH vs ITU comparisons) and Supplementary Section 2.2 (uncertainty derivation and methodological caveats). Supplementary Figure 14 presents three panels for each country: (a) prevalence of technology-facilitated CSEA among internet users, (b) internet exposure rates, and (c) the resulting overall population prevalence. To complement these dual-reporting prevalence estimates, we added

Extended Data Figures 1 and 2 (Page 81-82), which show survey-weighted prevalence for each CSEA type and the relationship between prevalence among internet users and internet exposure.

Results overview: The dual-reporting approach suggested by the reviewer reveals important patterns. In high-penetration settings (e.g., Philippines, Malaysia, Thailand), overall prevalence closely tracks prevalence among internet users because most children are online. In lower-penetration settings (e.g., Ethiopia, Uganda, Tanzania), overall prevalence is markedly lower despite comparable user-prevalence, reflecting limited exposure. Countries with large youth populations (e.g., Indonesia, Philippines) show substantial absolute numbers affected even where percentages are moderate, underscoring the value of reporting both relative and absolute burden (Page 10-12, Line 237-287; Table 1, Page 15-16).

> **Main text:** "To address the first research question, we identify the prevalence of each of nine categories of technology-facilitated CSEA, reporting rates for internet-using 12–17-year-olds and population-adjusted estimates for all children. Pooled across 12 nationally representative samples and surveys of 11,912 internet-using children in Eastern and Southern Africa and Southeast Asia, we found that one in six (17%, 95% CI = [16.2, 17.8]; *n* = 2025 children) had experienced at least one instance of the technology-facilitated CSEA included in this study, in the year 2020-2021. Considering all instances of CSEA regardless of where it was perpetrated, one in three (31%, 95% CI [30.1, 32.1]; *n* = 3477) internet-using children experienced some form of sexual exploitation or abuse (Supplementary Table 3 and and 50-51). These included incidents occurring via digital (social media, online games), in person, some other way, or where setting was not specified (don't know/prefer not to say).
>
> Specifically, on social media or in an online game, about 10% of children received both unwanted sexual images (95% CI = [9.0, 10.2]; *n*=1143) and 8% received comments that made them feel uncomfortable (95% CI = [6.9, 8.0]; *n*=889). Additionally, 5% (95% CI = [4.4, 5.2]; *n*=571) were asked to discuss sex or sexual acts, while 4% (95% CI = [3.5-4.3]; *n*=462) were asked online to explicitly do something sexual. Further, 4% (95% CI = [3.8, 4.6], *n*=498) of children were asked for a photo or video showing their private parts, and about 3% (95% CI = [2.4, 3.1], *n*=328) of children were offered money or gifts online to meet the perpetrator in person to perform sexual acts. Finally, 3% of children reported that their sexual images were shared without their consent (95% CI = [2.5, 3.1]; *n*=332), or they were offered money or gifts to share sexual images (95% CI = [2.4, 3.1; *n*=324) and even blackmailed online to engage in sexual activity (95% CI = [2.1, 2.8]; *n*=294). Further details on the digital platforms and perpetrator's identity associated with technology-facilitated CSEA are provided in Supplementary Figures 17 to 19.

Rates of technology-facilitated CSEA varied between countries, ranging from an estimated 5.5% [3.7 - 7.3, *n* = 54] of internet-using children in Vietnam to 29% [25.3 - 31.9, *n* = 271] in Philippines. Further, there were notable differences in the types of exposures to technology-facilitated CSEA. For instance, less than 1% of children in Vietnam were asked to talk about sex or sexual acts online, while this occurred to almost 9% of children in the Philippines (Figure 1 and Supplementary Figure 2-10; Table 53). Extended Data Fig. 1 presents survey-weighted prevalence for each type of technology-facilitated CSEA across 12 countries, with 95% confidence intervals.

To calculate the proportion of all children aged 12-17 experiencing technology-facilitated CSEA, we multiplied each country's internet penetration rate (collected via Disrupting Harm household surveys) by prevalence among internet users (see Table 1 and Extended Data Fig. 2). Across 12 countries, this corresponds to approximately **10.7 million children (**95% CI = [7,931,739, 13,181,985]). In countries with widespread internet penetration, such as the Philippines (95%), Malaysia (94%), and Thailand (92%), the prevalence for the child population closely mirrors that among children with internet access. In contrast, in countries with more limited access, such as Ethiopia (25%) and Uganda (40%), the overall prevalence is attenuated. As connectivity is expected to rapidly advance in these countries, prevalence may increase significantly over the next decade. Countries with large youth populations such as Indonesia and the Philippines also face particular challenges, as even a moderate prevalence translate into substantial absolute numbers of affected children."

Table 1 Country-level prevalence and population impact of technology-facilitated CSEA among 12–17-year-olds (2020–2021). Column a: Country: Countries included in the Disrupting Harm data, ordered by (Cambodia, Ethiopia, Indonesia, Kenya, Malaysia, Mozambique, Namibia, Philippines, Tanzania, Thailand, Uganda, Vietnam). **Column b:** Prevalence of technology-facilitated CSEA among internet-using children aged 12-17: Percentage of internet-using 12–17-year-olds who experienced any form of technology-facilitated CSEA in 2020-2021, estimated from the Disrupting Harm child survey using nationally representative probability samples with design weights. **Column c:** Proportion of child internet users, aged 12-17: Percentage of all 12-17-year-olds in each country who use the internet (any use, any device), estimated from the Disrupting Harm household survey module administered at every sampled household. **Column d:** Prevalence of technology-facilitated CSEA among all children: Percentage of all 12-17-year-olds who experienced technology-facilitated CSEA, calculated by multiplying prevalence among internet users (column b) by the proportion of child internet users (column c) in each country; uncertainty propagated using Monte Carlo simulation (5,000 iterations per country). **Column e:** Estimated number of affected children: Absolute number of 12-17-year-olds estimated to have experienced technology-facilitated CSEA in each country, calculated by applying the prevalence among all children (column d) to the total population aged 12-17 (2020) (column f) (95% CIs reflect uncertainty in prevalence only (population treated as fixed). **Column f:** Child population aged 12–17: The total number of children aged 12–17 in each country, derived from United Nations World Population Prospects 2022 (reference year: 2020).

Country	Prevalence of technology-facilitated CSEA among internet using children aged 12-17	Proportion of child internet users, aged 12–17.	Prevalence of technology-facilitated CSEA among all children	Estimated number of children affected	Child population aged 12–17
**Cambodia**	14.7%	81.0%	11.9% (9.9%–14.1%)	215,962 (179,843–256,539)	1,819,679
**Ethiopia**	18.6%	25.0%	4.6% (3.5%–6.1%)	775,526 (581,437–1,018,510)	16,680,781
**Indonesia**	6.6%	92.0%	6.1% (4.6%–7.9%)	1,665,769 (1,250,027–2,150,872)	27,231,704
**Kenya**	21.7%	67.0%	14.5% (12.4%–16.8%)	1,096,163 (932,413–1,269,843)	7,539,254

**Malaysia**	10.2%	94.0%	9.6% (7.6%–12.0%)	306,234 (241,236–380,807)	3,178,610
**Mozambique**	25.9%	56.0%	14.5% (12.3%–17.0%)	634,331 (536,934–744,858)	4,381,428
**Namibia**	20.0%	81.0%	16.2% (13.6%–18.9%)	47,686 (40,172–55,612)	294,899
**Philippines**	28.6%	95.0%	27.1% (23.1%–30.6%)	3,580,485 (3,043,351–4,035,759)	13,190,360
**Tanzania**	7.7%	67.0%	5.2% (4.0%–6.7%)	456,274 (353,053–589,653)	8,813,838
**Thailand**	17.0%	92.0%	15.6% (13.0%–18.2%)	769,133 (642,336–896,437)	4,922,710
**Uganda**	27.7%	40.0%	11.1% (9.1%–13.3%)	755,632 (623,496–907,413)	6,819,466
**Vietnam**	5.5%	89.0%	4.9% (3.6%–6.5%)	410,438 (300,307–548,994)	8,421,996

Extended Data Fig. 1; While our main text focuses on prevalence of any technology-facilitated CSEA, this extended figure allows readers to examine variation in specific technology-facilitated CSEA types (e.g., sexual extortion, grooming, unwanted exposure to sexual content) (Page 81).

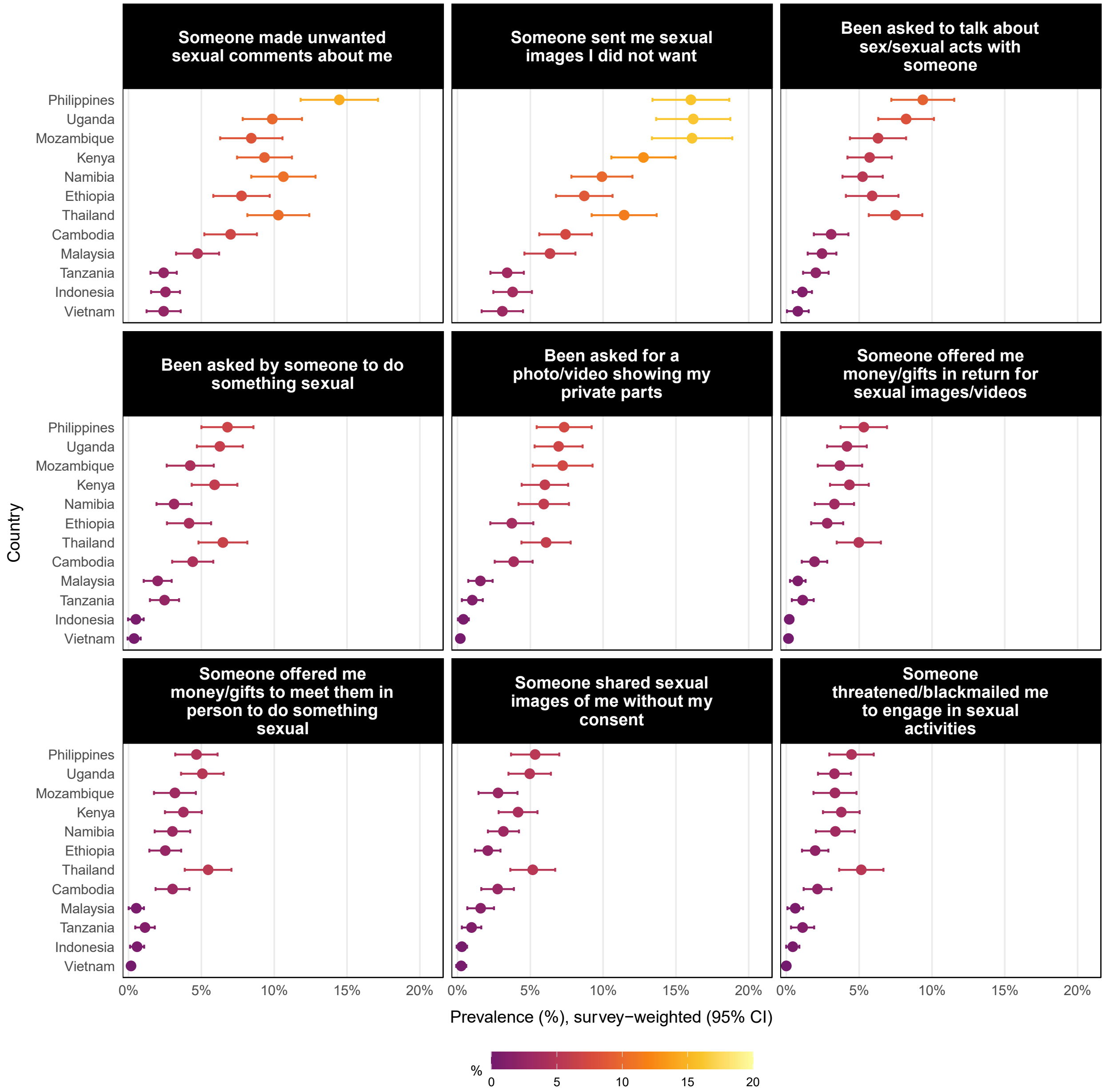

Extended Data Fig. 1: Prevalence of technology-facilitated child sexual exploitation and abuse by country (via social media or online game). Survey-weighted prevalence estimates with 95% design-based confidence intervals for nine types of technology-facilitated CSEA experienced by internet-using children aged 12–17 years. Colour indicates prevalence (lighter = higher). Error bars represent 95% confidence intervals accounting for the survey design. Countries are ordered by prevalence within each harm type: Philippines, Uganda,

Mozambique, Kenya, Ethiopia, Namibia, Thailand, Cambodia, Malaysia, Tanzania, Indonesia, Vietnam.

Extended Data Fig. 2: We also provide an intuitive visual showing the relationship among internet-using children and reach of the internet to the population-level burden (Page 82).

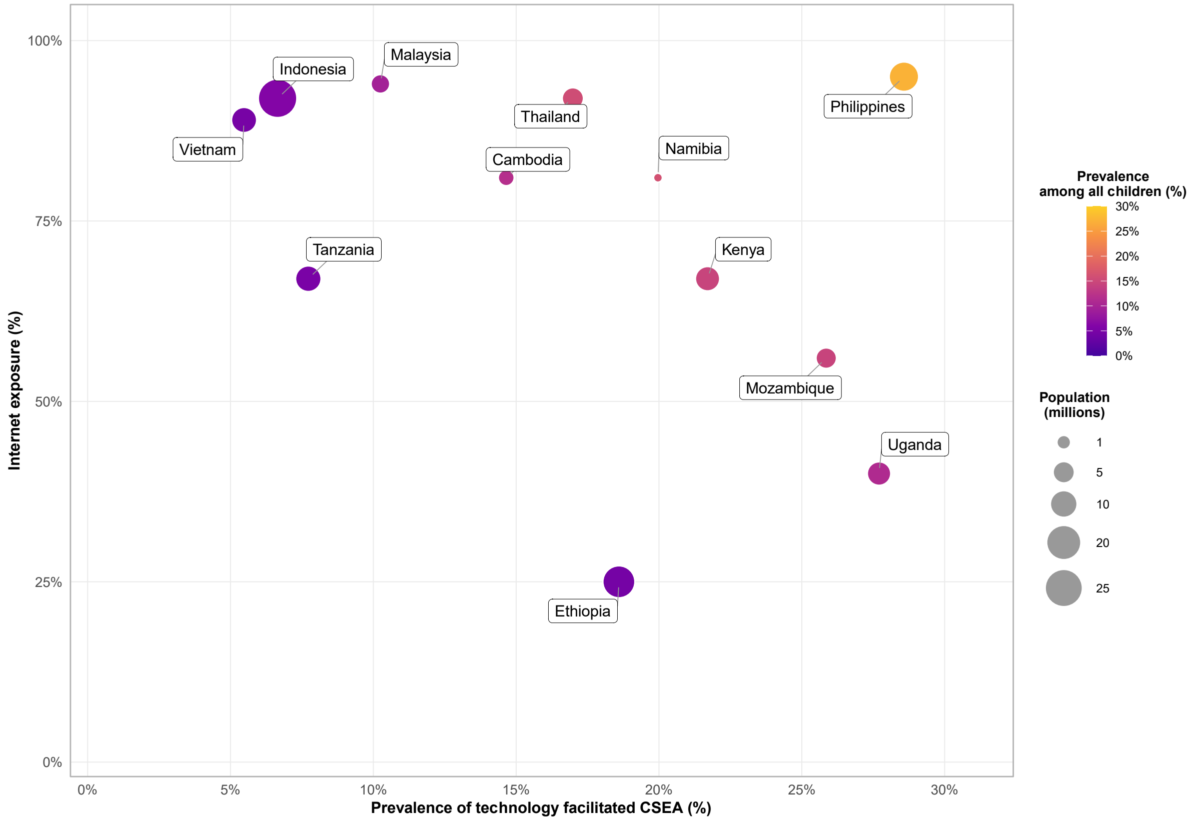

Extended Data Fig. 2. Relationship between prevalence of technology facilitated CSEA among internet users, internet exposure, and estimated overall prevalence. Scatter plot with point size proportional to population showing prevalence among internet-using 12–17-year-olds who reported one or more forms of technology-facilitated CSEA (x-axis) versus internet-exposure among all 12–17-year-olds (y-axis), across 12 countries. Colour denotes overall prevalence among all children (the product of the x- and y-axis values); bubble size denotes the 12–17 population (millions). Estimates are survey-weighted. Data sources: Disrupting Harm child and household surveys (2020–2021) and UN World Population Prospects 2022 (2020 population estimates).

Methods: Our expanded Methods section now describes these data sources and calculations (Page 49-50, Line 873-891; Page 51-53, Line 924-952).

"Disrupting Harm household survey (internet exposure): In each country, the Disrupting Harm household survey (2020–2021) visited a nationally representative sample of approximately ~1,500–10,000 households depending on connectivity. At each household,

enumerators established whether any children aged 12–17 lived in the household and, if so, whether those children used the internet (via any device and at any location). These exposure data were collected regardless of whether a child from the household completed the separate CSEA interview, thereby avoiding selection on child-interview participation. Aggregating responses across sampled households yields the national proportion of 12–17-year-olds who are internet users for each country. For validation, we compare these estimates with International Telecommunication Union (ITU) youth internet-use indicators (typically ages 15-24, nearest available year); however, because ITU data differ in age band and reference period, we retain the Disrupting Harm household data as our primary exposure source (Supplementary Figure 12).

Measures

Overall Instance of Technology-facilitated Child Sexual Exploitation and Abuse

Measuring technology-facilitated CSEA: Prevalence of CSEA among internet-using was measured using a composite variable capturing whether a child had experienced any form of technology facilitated CSEA within the past year, as reported during the 2020-2021 survey period. [rest of the CSEA variable construction section]"

"Dual reporting approach: We report technology-facilitated CSEA prevalence two ways. First, we estimate prevalence among internet-using children directly from the Disrupting Harm child survey. Second, we estimate prevalence in the total population aged 12-17 by multiplying individual-level prevalence by internet penetration rates from the Disrupting Harm household survey. This approach assumes that only children with internet access can experience technology-facilitated harms.

Uncertainty Propagation: We propagated uncertainty from both survey components using Monte Carlo simulation to estimate 95% confidence intervals for population-level prevalence and victim counts. We drew 5,000 samples from each component's sampling distribution on the logit scale, calculated the product for each iteration, and extracted the 2.5th and 97.5th percentiles of the resulting distribution (see Supplementary Figures 12-17 for details). This approach treats the child and household surveys as statistically independent, justified by their separate sampling frames, child respondents, and field operations. We calculated standard errors using effective sample sizes (Kish's method) that account for survey weights. For the internet exposure component in countries where sample sizes were unavailable, we applied conservative default standard errors (3 percentage points for internet exposure and 5 percentage points for prevalence among internet users). These defaults affect only the width of the confidence intervals, not the point estimates.

Several considerations warrant caution when interpreting population-level estimates. First, internet exposure data were collected in 2020-2021. In rapidly digitalising countries, exposure rates have likely increased substantially since then, meaning our estimates do not represent

current population burden. Second, the household measure captures regular internet use but may not capture occasional use outside the home (e.g., at schools or community centers), leaving our prevalence estimates conservative. Third, our framework assumes children without internet access cannot experience technology-facilitated harms, an assumption that cannot be empirically validated with our data. Given these constraints and propagated uncertainty, population-level estimates should be interpreted as illustrative indicators of relative burden across countries rather than precise national estimates. Full list of caveats are provided in Supplementary 2.2."

Supplementary:

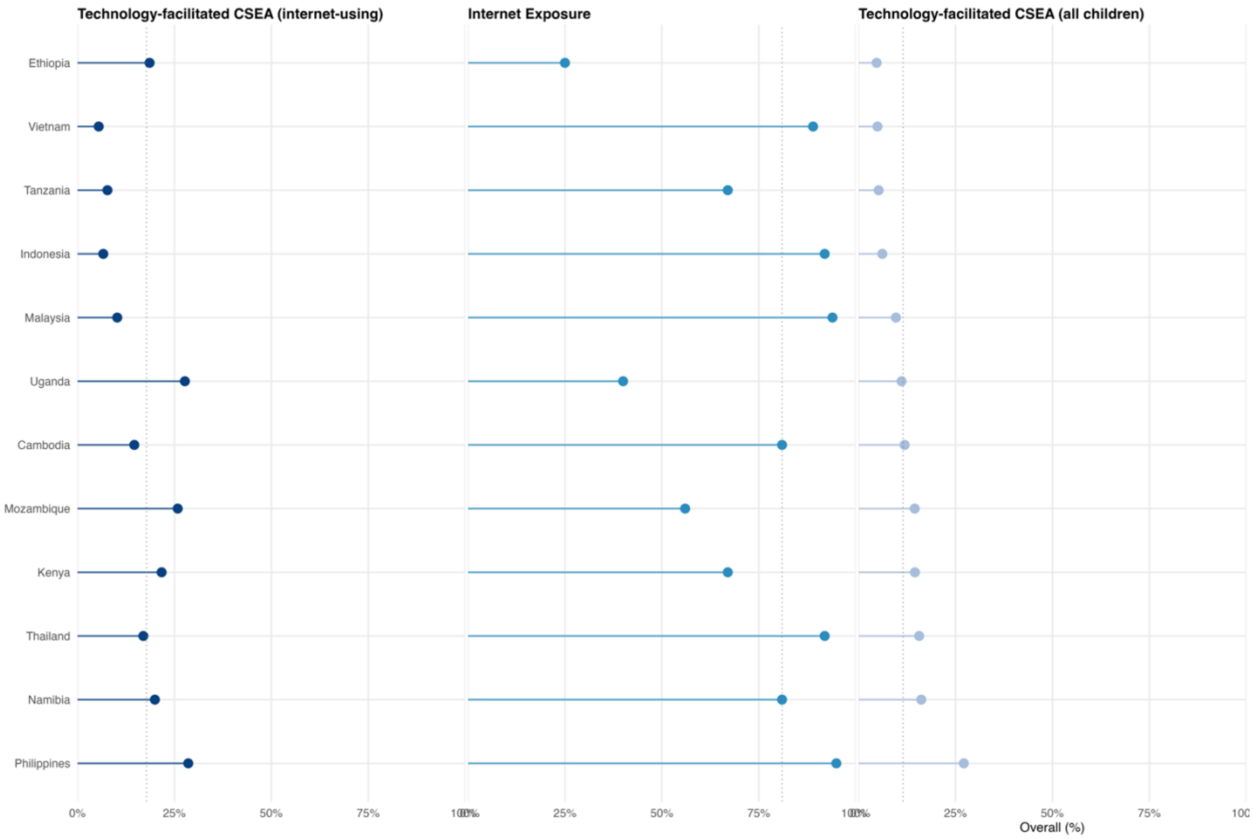

Supplementary Figure 14: Technology-facilitated CSEA, exposure, and overall population prevalence (ages 12–17). Panel (a) shows the prevalence of technology-facilitated CSEA among internet-using 12–17-year-olds (Disrupting Harm (DH) child survey; survey-weighted). Panel (b) shows internet exposure from the DH household survey: the percentage of all 12–17-year-olds who use the internet. Panel (c) shows the overall prevalence among all 12–17-year-olds, computed as Panel (a) × Panel (b) for each country and year. Countries are ordered by the overall estimate in Panel (c); dashed vertical lines mark medians.

Supplementary 2.1. Data Sources: To compute the dual reporting approach (among internet-using versus all children), we estimate the share of children who go online using the following two comparative sources.

a) Estimating exposure (primary source): We estimate the share of 12–17-year-olds who use the internet with the Disrupting Harm (DH) household module (see Figure 11). During each household visit, enumerators first confirm whether any 12–17-year-olds live in the household and then ask whether those children use the internet (any use). The module was fielded nationally with probability sampling (approximately 1,500–10,000 households per country, depending on connectivity and country size), yielding age-specific estimates aligned to our fieldwork period. The DH household item is intentionally simple, a household-level screen about internet use among resident 12–17-year-olds to ensure consistent implementation across countries and years. This preserves both age specificity (12–17) and timing (aligned with the child survey). The module captures any internet use irrespective of device, making it appropriate for defining exposure for technology-facilitated outcomes.

b) External context (not used in main estimates). For context and face validity, we next compare youth internet-use indicators from the International Telecommunication Union (ITU) (see Figure 13). ITU compiles country-reported statistics, primarily nationally representative household surveys using standard ICT questions, into a harmonised database. Where recent observations are missing, ITU may publish estimates informed by earlier surveys and regional patterns. Age breakdowns are commonly available for youth (often 15–24 years), with additional disaggregation by sex and urban/rural in some settings (Figure 12). Internet use is defined broadly (any location, any device), but reference periods and exact wording can vary by country and year. Availability is uneven and reference years may not align with our fieldwork. To preserve age specificity (12–17) and timing, we retain the DH household estimate as the primary exposure measure and present ITU series as context in the Supplement.

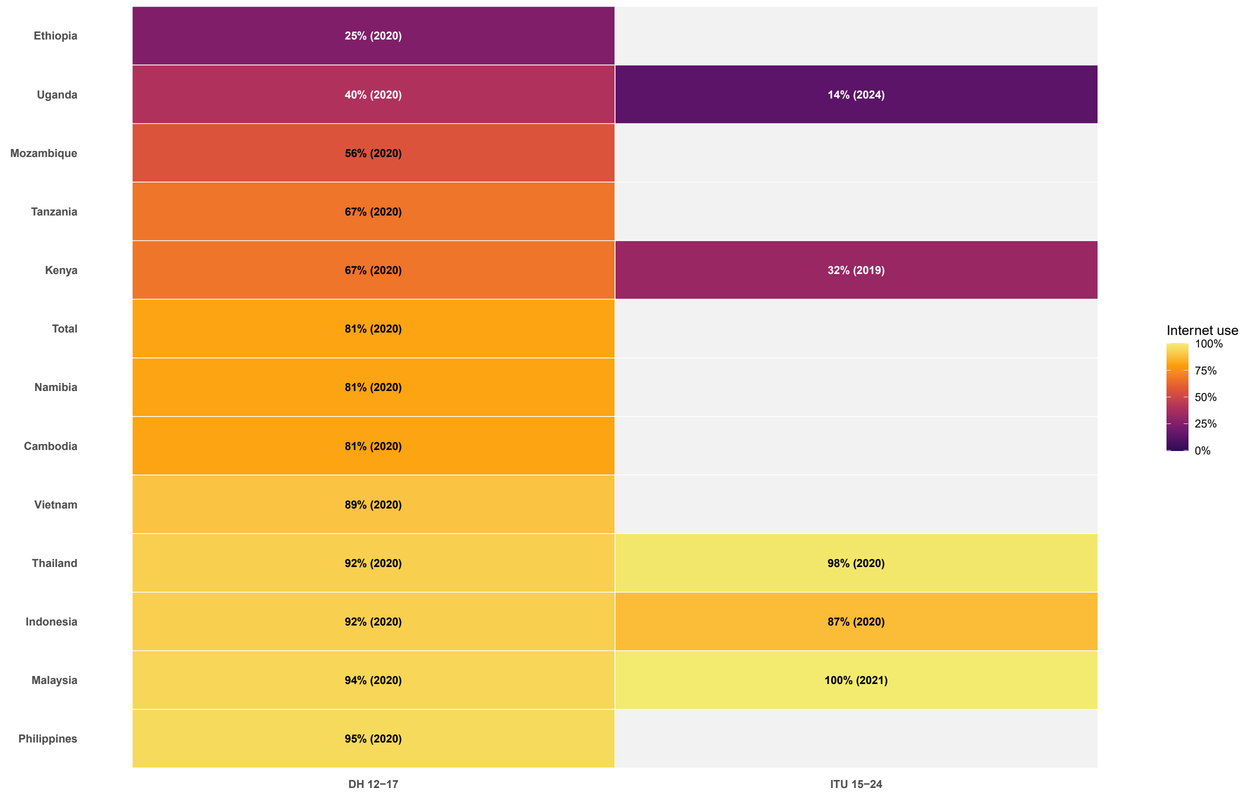

Supplementary Figure 12: Availability of internet exposure data from Disrupting Harm survey versus International Telecommunication Union (ITU) data. Heatmap showing internet use for study countries using DH (12-17) and ITU youth statistics (15–24 years) as the closest available proxy to our target group (12–17 years). Each cell reports the percent using the internet and the reference year in parentheses (latest available; or nearest to 2020 if specified), with colour mapping 0–100%.

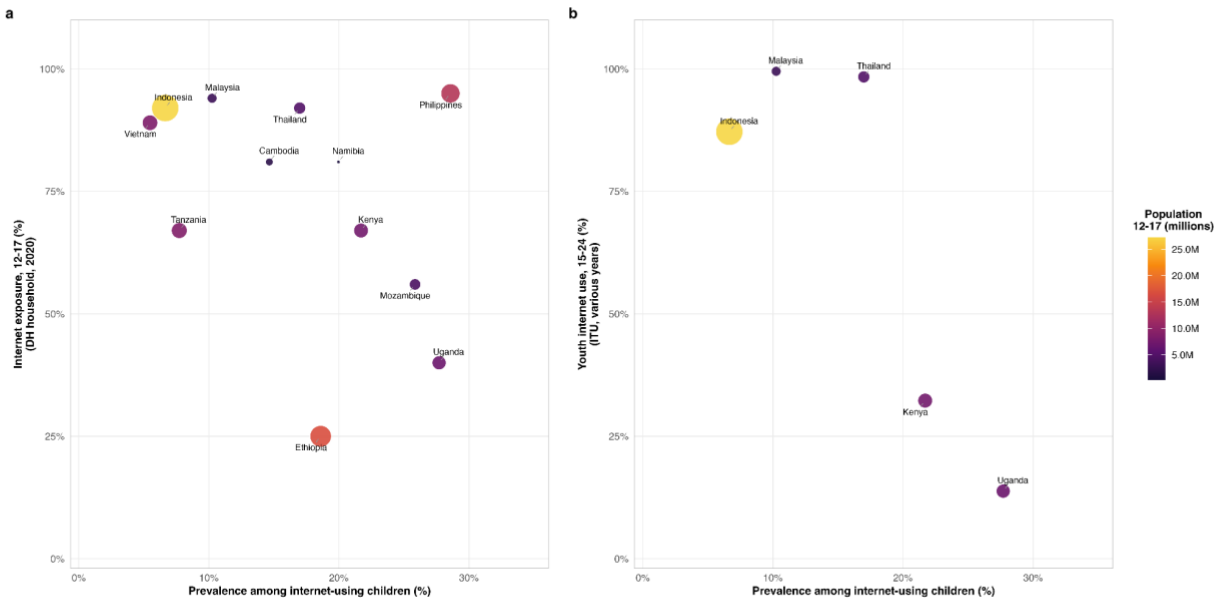

Supplementary Figure 13: Comparison of Disrupting Harm (DH) and International Telecommunication Union (ITU) internet exposure measures. Panel (a) Prevalence among internet users (x-axis) versus DH household exposure estimates (y-axis, 12–17-year-olds, 2020). Panel (b) Prevalence among internet users (x-axis) versus ITU youth internet indicators (y-axis, 15–24-year-olds, various years). Point size and colour represent the 12–17-year-old population (millions). DH estimates (a) are used as the primary exposure measure; ITU indicators (b) provide validation.

Uncertainty: We quantify uncertainty in overall prevalence by propagating sampling error from both data sources, the DH child survey (prevalence among internet-using children) and the DH household survey (proportion of 12–17-year-olds online), using a parametric Monte Carlo procedure on the logit scale. For each country, we generate 5,000 random draws from the sampling distribution of each component, assuming approximate normality on the logit scale with means equal to the point estimates and standard deviations equal to the survey-design standard errors (or conservative defaults when standard errors are unavailable). Each draw is inverse-logit transformed back to the probability scale, and overall prevalence is calculated as the product of the paired draws from the two components. We report 95% confidence intervals as the 2.5th and 97.5th percentiles of the resulting distribution.

The DH household survey module asked enumerators to record whether 12-17 year-olds in each household used the internet, yielding nationally representative proportions. However, the aggregated data available to researchers contains only the final weighted proportions without the underlying sample counts or design parameters needed to calculate standard errors. This data structure requires our conservative default SE approach for the exposure component of the uncertainty propagation. Our conservative default of 3 percentage points ensures confidence intervals remain appropriately wide, avoiding false precision in population estimates

This approach treats the child and household surveys as independent samples, which is appropriate given their separate sampling frames and data collection procedures. National population sizes for ages 12–17 are treated as fixed. The confidence intervals for estimated numbers affected reflect uncertainty in the two prevalence rates but not in population counts. Point estimates of overall prevalence are calculated as the direct product of the two component estimates and do not depend on the Monte Carlo procedure or default standard error assumptions. This approach follows standard methods for propagating uncertainty in products of independent estimates (Wolter, 2007).

Methodological Caveats: Several factors mean that our estimates should be interpreted as conservative lower bounds on the true population estimates.

- *Temporal limitations.* Internet exposure data derive from the 2020 household survey, while child victimisation data span 2020–2021. In countries experiencing rapid digitalisation, exposure rates may have changed between these measurement periods and have increased substantially

by 2025. These estimates therefore reflect 2020–2021 conditions and do not represent current population burden.

- *Core analytical assumption.* Our framework treats children without internet access as unexposed to technology-facilitated harms. While this is a reasonable assumption for outcomes that require online interaction, it cannot be empirically verified and assumes a clear online/offline boundary that may not fully capture hybrid harm pathways.
- *Internet exposure measurement.* The DH household measure captures reported internet use by resident 12–17-year-olds but may not capture all occasional or informal internet access occurring outside the household (e.g., at schools, libraries, friends' homes, or public venues). Children reported as non-users in the household module may therefore have intermittent exposure to online harms meaning the true exposed population could be larger than estimated.
- *Uncertainty propagation methods.* We use Monte Carlo simulation (5,000 draws per country) with logit-transformed proportions, a standard approach for bounded measures that can be less accurate at extreme values (very close to 0 or 1). We bound all estimates to mitigate this limitation.
- *Survey independence assumption.* The DH child and household modules use separate sampling frames and are treated as statistically independent in our uncertainty propagation. While this is appropriate for sampling error, any unmeasured correlation between the two estimates within countries (e.g., due to shared fieldwork timing or geographic clustering) could affect confidence interval widths.

Ref: Wolter, K. M. (2007). *Introduction to variance estimation* (2nd ed.). Springer Science + Business Media.

Reviewer 2, Point 3: *In lines 183–184, the sentence 'We found that 51% of boys (n=1,026) and 49% of girls (n=999) ranging from 12 to 17 years old experienced at least one of the different forms of technology-facilitated CSEA" is not particularly informative. As written, it does not clarify whether boys or girls are more likely to experience technology-facilitated CSEA, since it lacks context on the underlying gender distribution among internet-using children. Depending on the original gender ratio (e.g., 52% boys and 48% girls, or vice versa), the interpretation of these numbers could vary significantly. A more informative approach would be to present the prevalence of experiencing at least one form of technology-facilitated CSEA separately for boys and for girls. This would better highlight any differences in risk between the two groups.*

We thank the reviewer for this important clarification. We acknowledge that the original text was misleading; it presented the gender distribution of victims (51% boys, 49% girls) rather than gender-specific prevalence rates. We have revised the manuscript to report prevalence by gender (Page 16, Line 318-321).

> **Demographic Differences**: "Among internet-using children aged 12-17, technology-facilitated CSEA prevalence was nearly identical for boys and girls: 17% (95% CI = [15.9,

18.0]; n = 1026) of boys and 17% (95% CI = [15.9, 18.1]; n =999) of girls experienced at least one form of technology-facilitated CSEA through social media or gaming platforms."

Population-level considerations: We further explored whether gender-specific population-level prevalence estimates (among all children) would be feasible. To that end, we investigated whether the International Telecommunication Union (ITU) DataHub could provide gender-disaggregated internet exposure for ages 12–17 (Supplementary Figure 12).

The ITU DataHub reports indicators on individuals using the internet disaggregated by age group, gender and location (e.g. 15–24 years, by gender and by location), but it does not provide official statistics simultaneously disaggregated by both the specific age band 12–17 years and gender (Supplementary Figure 12). To the best of our knowledge, after checking all 12 study countries for 2020–2021, no official ITU statistics report internet use jointly disaggregated by age 12–17 and gender. While one could attempt to construct such estimates by merging separate age-specific and gender-specific ITU series, this would yield derived rather than official ITU statistics and could introduce bias due to differences in survey years, samples or methods. Given these data constraints, we therefore report gender-specific prevalence among internet-using children from the Disrupting Harm child survey, where we have direct measurements.

Reference: International Telecommunication Union (ITU). DataHub: Individuals using the Internet (by age, sex, and location). ITU Statistics Division, Geneva. Available at: https://www.itu.int/en/ITU-D/Statistics/Pages/stat/default.aspx

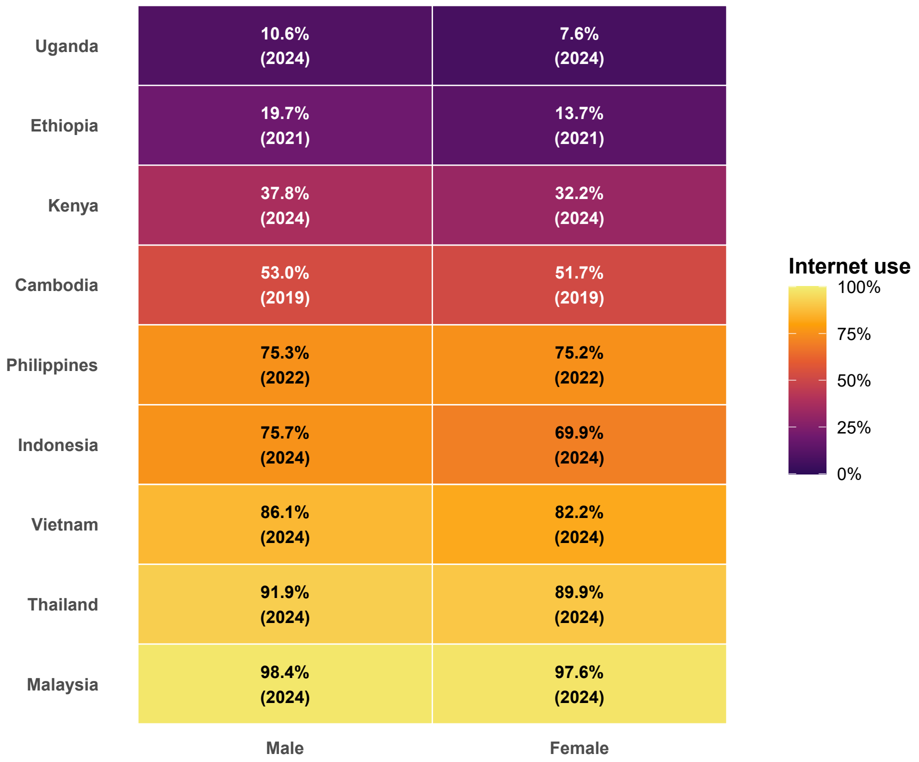

Supplementary Figure 12: Availability of gender-disaggregated internet-use data in study countries. Heatmap showing youth (15–24 years) internet use from ITU statistics, used as the closest available proxy to our target group (12–17 years). For each country and sex, cells show the percentage using the internet and the reference year (latest available, or nearest to 2020 if specified), with colours mapping 0–100%. Only nine of the twelve study countries have gender-disaggregated ITU data and are shown here, ordered by data availability.

Implications: Our finding that 17% of boys and 17% of girls experience technology-facilitated CSEA among internet users is substantively important. It demonstrates that the rate of harm in an internet-using population does not show large gender differences. This pattern aligns with evidence from a survey of 389 young people (between the ages of 18 and 34) in sub-Saharan Africa showing high exposure across genders without a statistically significant differences in any form of technology-facilitated sexual violence (Makinde et al., 2021).

These findings challenge the common assumption that girls are uniformly more affected than boys. In our pooled sample, harms among users were comparable across genders. Prevention strategies based solely on gendered risk assumptions may therefore inadvertently neglect substantial harm experienced by boys and fail to address the structural and behavioural factors that shape online vulnerability for all children. However, we also acknowledge that absolute numbers may differ from the value of knowing the conditional prevalence. Reporting gender-specific prevalence among

internet-using adolescents remains valuable because pathways, contexts, disclosure patterns, and effective safeguards for technology-facilitated CSEA differ for girls and boys (even when overall prevalence is similar). These changes are highlighted as follows (Page 48-49, Lines 860-864; Page 39, Lines 643-649).

> **Methods:** "Gender-specific estimates are calculated among internet-using adolescents from the Disrupting Harm child survey. However, we could not construct comparable gender-specific population denominators because harmonised data on internet use by age (12–17) and gender are not consistently available across all study countries, including in International Telecommunication Union statistics.
>
> **Revised text:** We found no gender differences in exposure rates, though this may mask important differences in context and consequences. Emerging evidence from the Disrupting Harm project indicates that societal expectations shape how perpetrators target children, how children interpret their experiences, and the responses they receive. Prevention strategies must therefore account for gendered dimensions of risk and response, even when prevalence rates appear similar. If internet access differs by gender (e.g., in many LMICs), absolute exposure numbers may vary substantially, thus warranting gender-disaggregated monitoring and support.
>
> **Ref:** Makinde O. A., Olamijuwon E., Ichegbo N. K., Onyemelukwe C., Ilesanmi M. G. (2021). The nature of technology-facilitated violence and abuse among young adults in sub-Saharan Africa. In Bailey J., Flynn A., Henry N. (Eds.), *The Emerald International Handbook of Technology-Facilitated Violence and Abuse* (pp. 83–101). *Emerald Publishing Limited.*

Reviewer 2, Point 4: *In the subsection "Disclosure of Technology-Facilitated CSEA," it would be helpful for the authors to clarify at the outset whether participants' responses to the disclosure questions were limited to a single choice or allowed for multiple selections. For example, if a child disclosed their experience to a parent, a friend, and a helpline, were they allowed to select all applicable options, or only the first person they disclosed to? This distinction is critical, as the interpretation of disclosure prevalence differs significantly depending on whether responses were single- or multiple-choice. The authors later mention that "children could select more than one disclosure type." If that is the case, it would be important to explain how disclosures to both formal and informal channels were handled in the subsequent analyses. Specifically, if a child disclosed to both types, were they classified as disclosing to both, or counted separately in each category? If the latter, how was this overlap accounted for, and what implications might it have for the findings and their interpretation? Children who disclose to both formal and informal channels may differ meaningfully from those who disclose only to one type. Simply including these children in both categories could blur distinctions between the groups, potentially limiting the clarity and validity of group comparisons.*

We thank the reviewer for this important comment regarding the disclosure measurement approach. We have revised the Methods and Supplementary to address interpretation concerns. Specifically, we provide three complimentary approaches to allow readers to understand: (a) overall patterns of any,

formal, and informal disclosure (non-mutually exclusive approach), (b) distinct disclosure pathways and how children differ based on whether they use single versus multiple disclosure routes (mutually exclusive approach), and (c) unique factors predicting disclosure through each specific channel (channel-specific analyses).

We appreciate the Reviewer 2's insight that children who disclose to both channel types may differ meaningfully from those who disclose exclusively to one type. The mutually exclusive categories (approach b) and channel-specific analyses (approach c) directly address this concern, while the non-mutually exclusive approach (approach a) provides behavioural insights into channel-specific predictors. Together, these three perspectives offer a more detailed understanding of disclosure.

(a) Mutually non-exclusive categories: First, our survey design allowed participants to indicate disclosure to each channel independently for each of the nine CSEA types. The instrument presented multiple potential disclosure targets (mother, father, police, helpline, teacher, social worker, friend, sibling, other trusted adults, other), and participants could select all sources without restriction. This multi-select format means channel proportions are non-exclusive and can sum to more than 100% by design. For example, a child who disclosed to both their mother and the police appears in both the informal and formal disclosure categories. This allows us to examine unique factors predicting disclosure through each specific channel, recognising that each disclosure represents a distinct behavioral decision that may be influenced by different factors (e.g., parental mediation may predict disclosure to parents while help-seeking knowledge predicts disclosure to formal channels). Further, our exploratory research question examined what predicts disclosure to each specific channel, rather than classification into mutually exclusive disclosure groups (Supplementary Table 41).

For our primary analyses, we therefore describe three composite outcomes in Methods (Page 52-53, Line 957-974).

> "To determine if children disclosed online sexual incidents, they were asked *'Who did you tell about what happened?"* and presented with a list of multiple response options such as potential individuals, educators, and law enforcement organisations. They could also respond that they hadn't disclosed to anyone, didn't know whether they had, or preferred not to say (multiple responses possible). Because each disclosure target is a distinct behavioural decision with potentially different determinants and our exploratory research question is channel-specific, we derived three child-level binary indicators:
>
> 1. Any disclosure (told all): Coded 1 if the child disclosed at least one CSEA type to any channel 0 otherwise
> 2. Informal disclosure (told informal): Coded 1 if the child disclosed any CSEA type to informal sources (parents/caregivers, siblings, friends, other children, other adults), 0 otherwise

3. Formal disclosure (told formal): Coded 1 if the child disclosed any CSEA type to formal authorities (teachers, police, helplines, social workers), 0 otherwise

    These categories are non mutually-exclusive. Children who disclosed to both formal and informal channels are counted in both categories because each disclosure represents a distinct behavioral decision potentially influenced by different factors. Children selecting only "didn't tell anyone" are counted as non-disclosures. Children could also select "prefer not to say," or "don't know" which are counted as non-responses. Because this question employed this multi-select format, channel proportions can sum to more than 100% by design, as children who disclosed to multiple sources are counted in each applicable category. See Supplementary Figure 24 for mutually-exclusive categories of disclosure."

<u>(b) Mutually exclusive categories:</u> Second, to separate patterns of single versus multi-route disclosure, we created four mutually exclusive categories: informal-only, formal-only, both informal and formal, or none. This provides a stacked 100% figure of the mutually exclusive categories by country, with a small dot overlay indicating any-disclosure percentages. Such an approach allows readers to examine distinct disclosure pathways (Supplementary Figure 24).

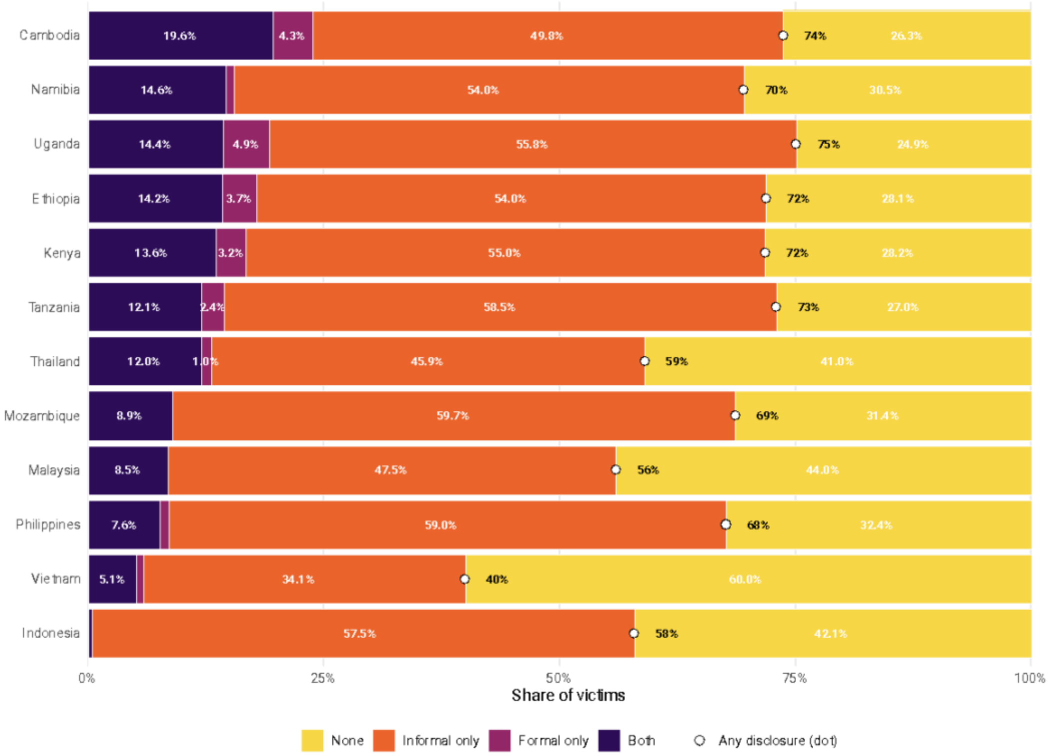

Supplementary Figure 24: Mutually exclusive disclosure categories by country. Bars show the share of victims in each group: None, Informal only, Formal only, and Both and sum to 100% within each country. The dot overlays the 'Any disclosure' proportion (logical

OR across channels); percentage labels indicate values. Countries are ordered by the share using both routes.

(c) New channel-specific analysis: Finally, we provide supplementary analyses examining each of the nine specific disclosure channels separately (mother, father, sibling, friend, teacher, police, helpline, social worker, other adults). These channel-specific models fit separate logistic regression models for each channel using identical predictor sets, allowing us to: (a) examine which factors distinguish disclosure to one particular channel, and (b) provide granular insight into the distinct pathways children use when disclosing.

To implement this, we first evaluated statistical power for each channel using events-per-variable (EPV) criteria (Supplementary Table 20). We then estimated odds ratios for all predictors separately by channel (Supplementary Tables 21–23) and visualised these as a forest plot to highlight category-specific effects (Supplementary Figure 30). These channel-specific models use random intercepts only and are based on complete-case analyses, in contrast to the primary manuscript models, which incorporate multiple imputation and random slopes. Accordingly, the supplementary channel-specific estimates are not directly comparable to the main results and should be interpreted as within-channel associations.

Supplementary 5.5: Channel-specific Analysis: "To provide granular insight into predictors of disclosure through specific pathways, we conducted supplementary analyses examining each disclosure channel separately (mother, father, sibling, friend, teacher, police, helpline, social worker, other adults). These channel-specific models used identical predictor sets to the main analyses but examined each channel independently, allowing us to identify which factors distinguish disclosure to one particular channel versus non-disclosure to that channel."

Statistical Power Assessment: "We first evaluated statistical power for each channel using events-per-variable (EPV) criteria. We excluded one channel (other: n=19, 0.9%, EPV=2.1) from regression analysis due to insufficient events. Three formal channels (police: n=58, helpline: n=63, social worker: n=60; EPV=6.4–7.0) have limited power and should be interpreted as exploratory findings. All other channels met conventional power thresholds (EPV>10)."

Channel-Specific Predictors: "We fit separate logistic regression models for each channel using composite case analysis. Channel-specific analyses revealed important distinctions in how predictors operate across pathways, showing meaningful heterogeneity that is not visible in composite analyses.

Age was the most consistent correlate, with each additional year associated with 13–28% lower odds of disclosure across 10 channels, including formal (helpline 28% lower; police 23%; social

worker 22%) and informal (father 17%; mother 24%; sibling 14%; other trusted adults 17%). These associations are consistent with lower odds of disclosure among older children across multiple channels.

More inequitable gender attitudes were associated with 30–39% higher odds of disclosure across six channels, primarily informal sources (e.g., mother 36% higher; father 33%; sibling 39%; friend 30%). The composite informal estimate was 35% higher. Associations with formal channels were not detected in channel-specific models.

Parental mediation was associated with 18–35% higher odds of disclosure across five channels, with larger associations for formal channels overall (35%) and mothers specifically (29%). Positive associations were also observed for fathers (23%) and for informal channels collectively (18%).

Gender (female vs male). Girls had higher odds of disclosure to teachers (73% higher), friends (36%), and any channel overall (40%), but lower odds to fathers (32% lower; OR 0.68). This pattern indicates channel-specific gender differences in disclosure.

Help-seeking knowledge. Knowledge was associated with higher odds of disclosure across three channels (friends 54% higher; informal channels collectively 47%; any disclosure 40%). Associations with individual formal services were not detected in channel-specific models, although the composite formal indicator was associated with knowledge in the main models.

Attitudes toward premarital sex. More permissive attitudes were associated with 33–59% higher odds across three informal channels (father 59%; sibling 47%; friend 33%), with no detected associations for formal channels.

Sex education was associated with higher odds of teacher disclosure (150% higher) but wider CI, with no detected associations for other channels. This highly specific association is consistent with the idea that school-based sex education may create conditions that help disclosure to teachers, for example, by establishing teachers as trusted sources of information about sexual matters or by normalising conversations about sexual topics in educational settings (but cannot be taken as causal evidence).

Digital skills. Higher digital skills were associated with 23% lower odds of disclosure to fathers, with no associations detected for other channels.

Taken together, the channel-specific analyses indicate several consistent patterns. First, age shows a broadly negative association with disclosure across most channels, suggesting lower odds of disclosure among older adolescents regardless of pathway. Second, gender associations are channel-dependent: girls have higher odds of disclosure for most channels but

lower odds to fathers, pointing to gendered dynamics in parent–child communication. Third, parental mediation is positively associated with disclosure across both informal and formal pathways, whereas more permissive attitudes toward premarital sex are associated with disclosure to family and friends. Fourth, help-seeking knowledge is associated with higher odds of disclosure overall without strong differentiation by specific formal services. Finally, sex education shows a specific association with disclosure to teachers (although wide CIs), suggesting that the school context may be particularly relevant for that pathway.

Channel-specific models used complete case analysis (no multiple imputation) and random intercepts only (no random slopes), reflecting the simpler models needed given reduced sample sizes when disaggregating by channel. These methodological differences mean that estimates from channel-specific models are not directly comparable to the primary models reported in the main manuscript, which used multiple imputation and random slopes. Channel-specific results should be read as supplementary, within-channel associations that highlight heterogeneity in predictors across disclosure pathways rather than as direct comparisons to composite models."

Channel	N disclosed	N total	Prevalence (%)	EPV	Analysis
**Other**	19	2067	0.9	2.1	Descriptive only
**Social Worker**	58	2067	2.8	6.4	Regression (exploratory)
**Police**	59	2067	2.9	6.6	Regression (exploratory)
**Helpline**	63	2067	3	7	Regression (exploratory)
**Other adult**	130	2067	6.3	14.4	Regression
**Teacher**	179	2067	8.7	19.9	Regression
**Father**	384	2067	18.6	42.7	Regression
**Mother**	420	2067	20.3	46.7	Regression
**Sibling**	516	2067	25	57.3	Regression
**Friend**	956	2067	46.3	106	Regression

Supplementary Table 20: Channel-specific power (events-per-variable, EPV) and inclusion notes. Counts are complete-case, unweighted.

	Any channels	Formal channels	Informal channels	Police	Helpline	Social worker	Teacher	Mother	Father	Sibling	Friend	Other Adult
Girls (vs Boys)	****1.40 (1.07–1.83)****	1.34 (0.90–1.99)	1.27 (0.98–1.64)	1.12 (0.57–2.17)	0.84 (0.41–1.70)	0.95 (0.45–2.04)	****1.73 (1.06–2.85)†****	1.16 (0.85–1.60)	****0.68 (0.50–0.93)****	1.27 (0.96–1.71)	**1.36 (1.06–1.76)**	1.02 (0.59–1.78)
Age (per year)	****0.87 (0.79–0.95)****	****0.79 (0.69–0.89)****	****0.87 (0.80–0.96)****	****0.77 (0.63–0.94)****	****0.72 (0.56–0.90)****	****0.78 (0.61–1.00)****	0.88 (0.75–1.04)	****0.76 (0.69–0.84)****	****0.83 (0.75–0.92)****	****0.86 (0.78–0.95)****	0.97 (0.89–1.06)	****0.83 (0.71–0.99)****
Premarital sex	1.26 (0.92–1.73)	0.79 (0.50–1.25)	1.32 (0.97–1.83)	0.47 (0.19–1.07)	0.89 (0.37–2.05)	1.37 (0.58–3.18)	0.69 (0.37–1.24)	1.05 (0.72–1.48)	****1.59 (1.09–2.28)†****	****1.47 (1.08–2.03)****	****1.33 (1.01–1.76)****	1.38 (0.75–2.49)
Inequitable attitudes towards sex	****1.35 (1.16–1.56)****	1.19 (0.97–1.45)	****1.35 (1.17–1.57)****	1.38 (0.99–1.92)	1.18 (0.83–1.69)	1.00 (0.65–1.49)	1.24 (0.97–1.60)	****1.36 (1.16–1.59)****	****1.33 (1.14–1.57)****	****1.39 (1.20–1.60)****	****1.30 (1.14–1.49)****	1.19 (0.91–1.54)
Parental mediation	****1.21 (1.03–1.43)****	****1.35 (1.08–1.68)****	****1.18 (1.00–1.39)****	1.39 (0.96–1.99)	1.18 (0.80–1.78)	1.25 (0.84–1.89)	1.22 (0.93–1.61)	****1.29 (1.08–1.54)****	****1.23 (1.03–1.47)****	1.15 (0.98–1.34)	0.98 (0.85–1.13)	1.20 (0.90–1.59)

Sex education	0.86 (0.64–1.16)	1.20 (0.80–1.82)	0.90 (0.68–1.21)	0.70 (0.34–1.40)	1.61 (0.72–3.60)	0.47 (0.20–1.12)	****2.50 (1.41–4.58)†****	1.32 (0.93–1.85)	0.96 (0.68–1.35)	1.25 (0.92–1.72)	1.19 (0.91–1.55)	1.06 (0.59–1.86)
Help-seeking	**1.40 (1.06–1.86)**	0.88 (0.59–1.31)	**1.47 (1.13–1.93)**	0.79 (0.38–1.56)	0.52 (0.24–1.10)	0.83 (0.36–1.85)	0.99 (0.61–1.64)	1.14 (0.83–1.57)	1.20 (0.87–1.70)	1.18 (0.89–1.57)	****1.54 (1.20–2.00)† ****	0.68 (0.39–1.22)
Digital skills to report	0.99 (0.87–1.12)	0.98 (0.82–1.16)	0.95 (0.84–1.07)	0.81 (0.60–1.07)	0.86 (0.62–1.19)	1.18 (0.85–1.63)	0.95 (0.75–1.18)	1.05 (0.91–1.21)	****0.77 (0.66–0.88)****	1.10 (0.97–1.24)	1.06 (0.95–1.19)	0.96 (0.76–1.21)

Supplementary Table 21-23: Channel-specific predictors of disclosure following technology-facilitated CSEA. Separate logistic regression models for each disclosure channel showing adjusted odds ratios (OR) and 95% confidence intervals. Models include identical predictor sets examining demographic and sociocultural factors. Estimates are not directly comparable to main manuscript models due to methodological differences (complete cases vs. multiple imputation; random intercepts only vs. random intercepts and slopes).

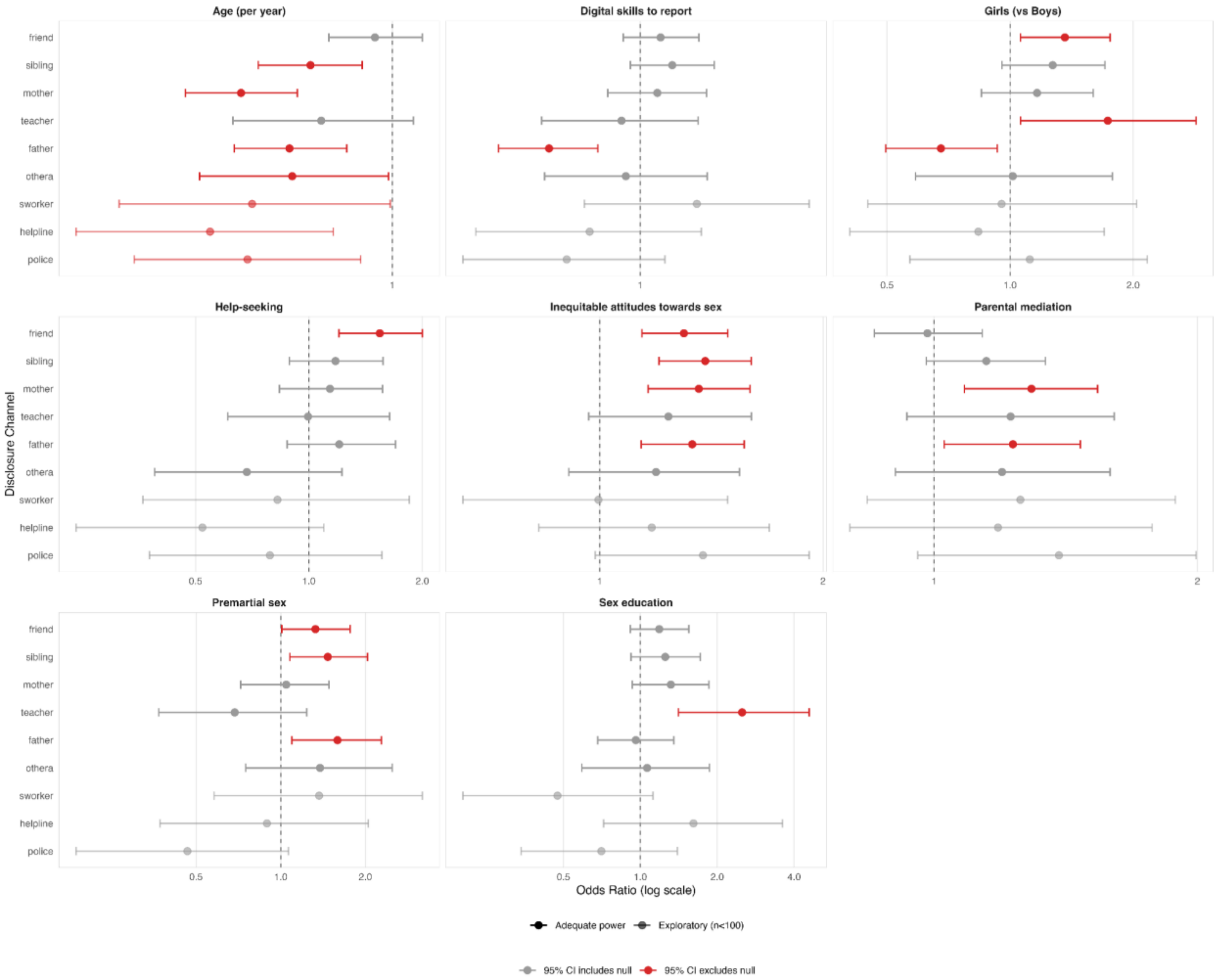

Supplementary Figure 30: Forest plot of channel-specific odds ratios (ORs) for disclosure. Odds ratios with 95% CIs for all predictors across each individual channel (separate logistic regressions; complete-case analytic sample).

Reviewer 2, Point 5: *I found Figure 3 somewhat difficult to interpret. For example, in the statement, "The most common barriers include children not knowing to whom they should disclose (e.g., 48% of instances involving sharing sexual images of the child without their consent)," does this mean that among all non-disclosure cases citing this specific barrier, 48% involved the sharing of sexual images without the child's consent? It would also be helpful for the authors to clarify whether the barrier question was asked only of children who did not disclose their CSEA experience, and whether respondents were allowed to select multiple barriers or only one. If multiple responses were permitted, how were cases reporting several barriers handled in the analysis? Were such cases counted multiple times across categories? The interpretation of the findings could vary considerably depending on the degree of overlap among reported barriers, so further clarification would strengthen readers' understanding. The authors may also wish to consider whether the overlap among barriers should be analyzed, in addition to treating them separately.*

Thank you for flagging the interpretability issues in Figure 3. We agree that Figure 3 required clearer signposting of the relevant denominator for all percentages shown, whether multiple barriers could be selected, and how overlap across barriers was handled.

Barriers to disclosure clarification: The barrier questions were administered only to non-disclosed incidents of technology-facilitated CSEA. Children at the CSEA-type level who did not disclose at least one barrier item were multi-select; children could select any of 13 barriers (Methods now includes full list of barriers). For each non-disclosed incident of technology-facilitated CSEA, the unit of analysis is the non-disclosed incident at the CSEA-type level (i.e., for each CSEA type where the child did not disclose). All reported percentages are survey-weighted shares of non-disclosed incidents citing each barrier; because the item is multi-select, within-type percentages do not sum to 100%.

To improve clarity, we revised Figure 3 into two panels and rephrased results to avoid ambiguity. Panel (a) pools all non-disclosed incidents across CSEA types to show overall barrier prevalences. Panel (b) presents the same ordered barriers within each CSEA type to aid comparison across types (see Reviewer 1, Point 5 for revised figure).

Barrier Overlap: The reviewer specifically suggested that any potential overlaps be examined. We therefore added an overlap analysis, including the co-occurrence of barriers. Among non-disclosed incidents, 74.6% cited a single barrier, while 13.3% cited 2-3 barriers and 2.5% cited 4 or more. This pattern indicates that barriers to disclosure do not collapse into a single, uniform profile, but instead reflect different types of challenges.

Some barriers appear to reflect informational gaps ("Did not know where to go/who to tell": 37.6%), others reflect emotional barriers ("Embarrassed/ashamed/too difficult": 19.6%), and still others involve anticipatory concerns about consequences ("Worried I would get in trouble": 14.0%).
We now acknowledge this heterogeneity in the Supplementary, noting that disclosure support may require tailored strategies to the specific combinations of barriers relevant to children's circumstances. These changes appear in the Results (Page 22, Lines 394-405); Supplementary Tables 45-46 and Methods (Page 54-55, Lines 983-1006).

> **Results:** "For non-disclosed incidents of technology-facilitated CSEA, children completed a multi-select barrier item asking why they did not tell anyone about the experience (see Supplementary Tables 31-32; Figure 25). Because respondents could select multiple barriers for each non-disclosed incident, percentages do not sum to 100% within any CSEA type. Pooled across CSEA types, (Figure 3a), the most common barrier to disclosure was "Did not know where to go or who to tell" (37.6%, 95% CI = [36.0, 39.6]), followed by "feeling embarrassed, ashamed or that it would be too emotionally difficult to tell": (19.6%, 95% CI = [18.0, 21.3]) and "did not think it was serious enough to report": (14.2%, 95% CI = [12.9, 15.7]). Figure 3b shows stratified barriers by each CSEA type. For example, among non-disclosed instances of sharing sexual images without consent, 48% (95% CI = [39.8, 56.5])

cited "did not know who to tell" as a barrier, while 18% (95% CI = [12.8, 25.9]) reported feeling embarrassed or ashamed."

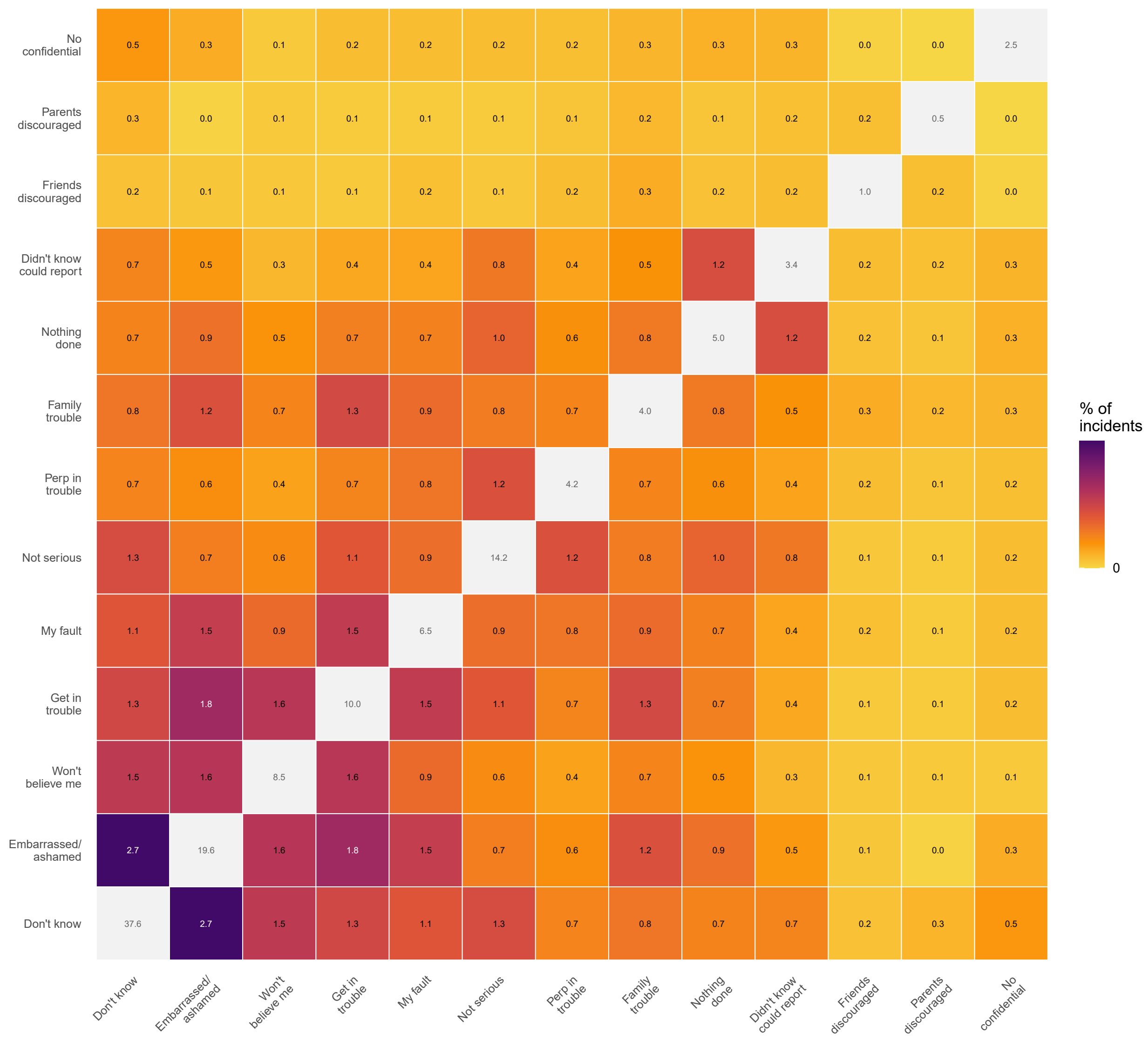

Supplementary Figure 25. Co-occurrence of barriers to disclosure among non-disclosed incidents (N=2,751). Heat map showing the weighted percentage of non-disclosed incidents in which each barrier combination was cited. Diagonal cells show individual barrier prevalence. Diagonal cells show individual barrier prevalence, while off-diagonal cells show co-occurrence rates between barrier pairs. Survey weights were applied to provide population-representative estimates.

"Among non-disclosed incidents, 74.6% cited a single barrier, while 13.3% cited 2–3 barriers and 2.5% cited 4 or more. The most common two-barrier combination was "Embarrassed/ashamed/too difficult" + "Did not know where to go/who to tell" (2.7% of non-disclosed incidents), followed by "Embarrassed/ashamed/too difficult" + "Worried I

would get in trouble" (1.8%). The most prevalent individual barriers were: (1) "Did not know where to go/who to tell" (37.6%), (2) "Embarrassed/ashamed/too difficult" (19.6%), and (3) "Did not think it was serious enough" (14.2%).

Importantly, barrier co-occurrence patterns revealed substantial heterogeneity in children's experiences. While some barrier pairs co-occurred, for example, 2.7% of non-disclosed incidents involved both "Embarrassed/ashamed/too difficult" and "Did not know where to go/who to tell", most barriers were cited relatively independently. Among children who cited "Embarrassed/ashamed/too difficult," 14.0% also cited "Did not know where to go/who to tell"; conversely, among those who cited "Did not know where to go/who to tell," 7.3% also cited "Embarrassed/ashamed/too difficult."

These findings suggest that barriers to disclosure do not collapse into a single, uniform profile but instead reflect several different obstacles. Some children report primarily informational barriers (e.g. "did not know where to go/who to tell"), others emphasise emotional barriers (e.g. "embarrassed/ashamed/too difficult"), while others highlight anticipatory concerns about consequences (e.g. "worried I would get in trouble", "wouldn't be believed"). This heterogeneity has potential implications for intervention design: rather than a one-size-fits-all approach, disclosure support may require strategies tailored to the specific combinations of barriers relevant to children's circumstances."

Methods: "Following the reporting item, adolescents who indicated "I did not tell anyone about it" for a given technology-facilitated CSEA harm received a multi-select barrier question ("Were any of the following reasons why you did not tell anyone about what happened?"). The unit of analysis is the non-disclosed incident at the CSEA-type level (i.e., for each CSEA type where the child did not disclose).

For each barrier, we estimated the survey-weighted proportion of all non-disclosed incidents that cited that barrier. Because the item is multi-select, an instance can contribute to multiple barrier categories, and percentages within a harm type need not sum to 100%. To assess whether barriers cluster together, we constructed a co-occurrence matrix of weighted joint percentages among all non-disclosed incidents (see Supplementary Figure 25; Table 56-57). Children could select any of the following 13 multi-select barriers:

- I did not know where to go or who to tell
- I felt embarrassed, ashamed or that it would be too emotionally difficult to tell
- I did not think anyone would believe me or understand my situation
- I was worried I would get in trouble if I told someone
- I felt that I did something wrong and did not want to tell
- I did not think it was serious enough to report
- I did not want the person who did this to get into trouble

- I feared it would cause trouble for me or my family
- I did not think anything would be done
- I did not know you could report these things
- My friends discouraged me from reporting
- My parents discouraged me from reporting
- I feared it would not be kept confidential"

Reviewer 2, Point 6: *I have some concerns about the use of a set of strongly interrelated predictors in modeling disclosure outcomes. When predictors are highly correlated, there is a risk of multicollinearity, which can distort the estimation of coefficients in a regression model. Specifically, multicollinearity can make it difficult to disentangle the unique effect of each predictor, potentially leading to misleading conclusions. A variable that is not causally related to the outcome might appear significant simply because it is correlated with a true causal factor whose effect is masked by multicollinearity. This can also result in apparent inconsistencies—where some correlated variables are significant predictors for one disclosure outcome but not another—due to statistical noise rather than meaningful differences. Such issues may partly explain findings that seem random or that contradict prior literature. The authors should consider testing for multicollinearity (e.g., using variance inflation factors) and may want to explore dimension reduction techniques or alternative model specifications to address this concern.*

We appreciate the concern raised that correlations among predictors can complicate coefficient interpretation and have taken several steps to address potential multicollinearity. Specifically, we tested multicollinearity using variance inflation factors (VIFs), visualised correlational structure among predictors, and reported model fit (R^2). These diagnostics are now presented in Supplementary Table 15 and 16 (VIF and R^2) and Supplementary Figure 26 (predictor correlation heatmap) (Supplementary Page 49-51).

Pairwise correlations among predictors were modest: the largest positive was 0.28 (premarital sex with inequitable gender attitudes), and the largest negative was −0.15 (age with parental mediation); no pair exceeded 0.30. All VIFs were well below 2, falling well below common concern thresholds and indicating no variance inflation that would undermine coefficient interpretation. As expected for behavioural disclosure outcomes, overall explanatory power (R^2) is modest, and the marginal-conditional gap indicates meaningful between-country heterogeneity captured by random intercepts.

All diagnostics were computed on the complete-case analytic sample (no imputation) to reflect the empirical covariance structure of the observed design matrix. Because multiple imputation can alter this structure in model-dependent ways, we judged complete-case diagnostics to be most interpretable for this purpose. As noted by Vanhove (2019), collinearity is most problematic when the goal is causal identification rather than characterising associations and generating hypotheses. Our models are strictly associational, and we do not draw causal inferences from individual coefficients. The above diagnostics are therefore used to check that multicollinearity is not likely to distort the reported associations.

Supplementary:

Correlations: "To assess potential multicollinearity among predictors, we examined pairwise correlations between all variables included in the disclosure models (Supplementary Figure 26). Correlations were computed on standardised predictors using survey weights to account for the complex sampling design, and results are visualised as a heatmap showing weighted Pearson correlations. Pairwise associations were modest: the largest positive correlation was 0.28 (premarital sex attitudes with inequitable gender attitudes), and the largest negative correlation was −0.15 (age with parental mediation). No pair of predictors exceeded r = 0.30, indicating that multicollinearity among model covariates was minimal."

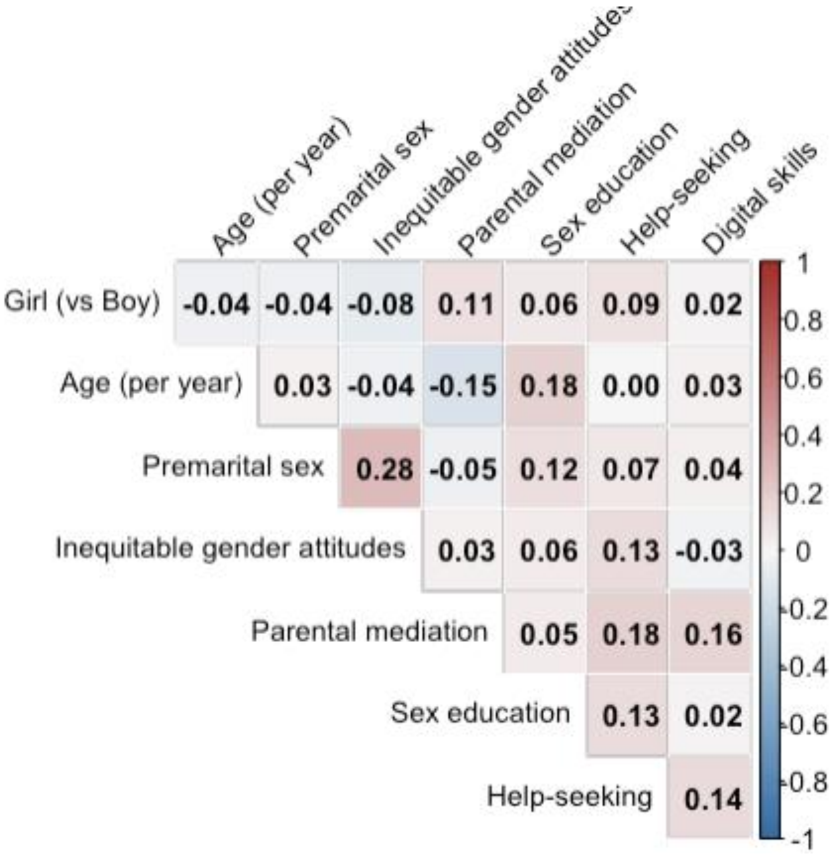

Supplementary Figure 26: Weighted correlations among predictors used in disclosure models. Cells show Pearson correlations computed on standardised predictors with survey weights applied.

Variance Inflation Factor: "We further computed variance inflation factors (VIFs) for all predictors in each disclosure model, using the complete-case dataset. All predictors enter with one degree of freedom, so classic VIF applies. To provide a scale-free summary we also report Inflation which is the square of VIF. Across models, VIFs were 1-1.2 (Inflation =1.0-1.1), well below common concern thresholds (~2–5) (Table 15). This suggests that the predictors are not strongly intercorrelated and that our estimates are not unduly distorted by collinearity."

Outcome	Term	VIF	Inflation
Any disclosure	Premarital sex	1.1	1.05
	Inequitable gender attitudes	1.08	1.04
	Positive parental mediation	1.07	1.04
	Sex education	1.07	1.04
	Help seeking	1.07	1.03
	Digital skills to report	1.05	1.03
	Girls (vs Boys)	1.03	1.02
	Age (per year)	1.05	1.02
Formal disclosure	Premarital sex	1.16	1.08
	Inequitable gender attitudes	1.15	1.07
	Positive parental mediation	1.09	1.05
	Sex education	1.1	1.05
	Help seeking	1.09	1.04
	Digital skills to report	1.08	1.04
	Girls (vs Boys)	1.06	1.03
	Age (per year)	1.04	1.02
Informal disclosure	Premarital sex	1.1	1.05
	Inequitable gender attitudes	1.07	1.04
	Positive parental mediation	1.07	1.04
	Sex education	1.07	1.04
	Help seeking	1.07	1.03
	Digital skills to report	1.06	1.03
	Girls (vs Boys)	1.03	1.02
	Age (per year)	1.05	1.02

Supplementary Table 15 Variance Inflation Factors (VIF) for predictors in each disclosure model. Values < 5 indicate acceptable levels of multicollinearity.

R2: "To contextualise the overall explanatory power of our models, we report Bayesian R^2 (posterior mean with 95% credible interval) for each disclosure outcome (Supplementary Table 16). For mixed models, we also report Nakagawa's marginal R^2 (variance explained by fixed effects) and conditional R^2 (variance explained by both fixed and random effects). The gap between marginal and conditional R^2 indicates the proportion of variance captured by country-level heterogeneity. As we would expect for behavioural disclosure outcomes, overall explanatory power is modest, and the gap between marginal and conditional R2 indicates meaningful between-country heterogeneity captured by the random intercepts."

Models	Bayes R^2 (mean)	Bayes R^2 (2.5%	Bayes R^2 (97.5%)	Nakagawa R^2 (marginal)	Nakagawa R^2 (conditional)
Any disclosure	0.056	0.016	0.095	0.119	0.23
Formal disclosure	0.047	0.016	0.086	0.087	0.19
Informal disclosure	0.053	0.015	0.091	0.119	0.18

Supplementary Table 16: Model-level fit indices. Bayesian R^2 (posterior mean, 95% CI) and Nakagawa's marginal and conditional R^2 are reported for each disclosure model.

Vanhove, J. (2019). Collinearity isn't a disease that needs curing. *Meta-Psychology*, 2021, vol 5, MP.2020.2548. https://doi.org/10.15626/MP.2021.2548

Reviewer 2, Point 7: *In addition, the possibility of reverse causality is a critical concern, especially given the cross-sectional nature of the data. Since all variables are measured at the same point in time, it is difficult to determine whether certain factors preceded the disclosure or occurred as a result of it. For example, the finding that "children who knew where to seek help following an assault were more likely to disclose" (lines 262–263) could reflect the reverse: children who disclosed may have subsequently received guidance about where to seek help. Similarly, the finding that "positive parental mediation was positively associated with a higher likelihood of disclosing through informal channels" (lines 271-273) may also be subject to reverse causality—children who disclosed to their parents might have prompted greater parental involvement in their media use as a response, rather than a cause. Regardless of direction, the associations may still appear statistically significant in regression models. However, the policy implications would differ drastically depending on whether the factor is a cause or a consequence. It is important that the authors avoid interpreting such associations as causal effects without stronger methodological support. If the intent is to inform policy or intervention design, the authors should consider methods that better address causality, such as instrumental variable techniques or longitudinal designs, where feasible.*

Thank you for this critical observation. High-quality data in the field of online violence against children remains rare, and this cross-sectional data provide nationally representative, population-based evidence from African and Asian contexts.

However, our study was not designed to identify causal effects, and we have clarified this limitation throughout the manuscript. We re-read all sections to remove phrasing that could imply causation from associations (e.g., "effects") and to frame results strictly as associations. We follow Kruschke et al (2021) Bayesian Analysis Reporting Guidelines throughout, reporting model specification (Bayesian multilevel logistic regression in brms with weakly informative priors), posterior summaries (means), 95% credible intervals, and posterior probabilities (e.g., PD). We have double-checked our language to distinguish associations and highlight uncertainty (in highlighted text) (Page 27-28, Lines 447-481).

> **Results:** "Exploratory Bayesian multilevel model examined factors related to children's disclosure of their exposure to technology-facilitated CSEA through any channel (i.e., formal or informal). Older children were less likely to disclose these experiences (*posterior mean* = -0.12, *SD* = 0.05, 95% CI = [-0.22, -0.03], PD = 99.4%) but this did not differ by gender (*posterior mean* = 0.13, *SD* = 0.16, 95% CI = [-0.19, 0.42], PD = 81.9%). In contrast, more parental involvement in children's digital lives (i.e., enabling parental mediation) related to higher overall disclosure rates (*posterior mean* = = 0.22, *SD* = 0.08, 95% CI = [0.06, 0.38], PD = 99.4%) Similarly, children who knew where to seek help following an assault were more likely to disclose (*posterior mean* = 0.38, *SD* = 0.13, 95% CI = [0.11, 0.67], PD = 99.5%). No relationships to disclosure were found for sex education, attitudes towards premarital sex, inequitable gender attitudes, and digital skills to report (95% CI crossed zero) (see Figure 4).
>
> Two supplementary exploratory analyses compared children's disclosure via informal and formal channels, revealing distinct factors associated with each. Children who knew where to seek help following sexual assault (posterior mean = 0.40, SD = 0.13, 95% CI = [0.15, 0.66], PD = 99.8%) and who received enabling parental mediation (posterior mean = 0.19, SD = 0.08, 95% CI = [0.04, 0.35], PD = 98.9%), were more likely to disclose through informal channels, potentially reflecting that the former addresses a key barrier to disclosure (Figure 3A). Children who believed in the acceptability of premarital sex (posterior mean = 0.33, SD = 0.16, 95% CI = [0.02, 0.64], PD = 98%) were also more likely to disclose to friends, family and other adults. Only two predictors were related to disclosure via formal channels: older children were less likely to disclose (posterior mean = -0.27, SD = 0.07, 95% CI = [-0.41, -0.14], PD = 99.9%), while children who received enabling parental mediation were more likely to do so (posterior mean = 0.32, SD = 0.11, 95% CI = [0.11, 0.54], PD = 99.7%)."

Reference: Kruschke, J.K. Bayesian Analysis Reporting Guidelines. *Nat Hum Behav* 5, 1282–1291 (2021).

Indeed, reverse causality remains a substantive concern, and we cannot establish directionality in our study. We have therefore added clarifying clauses in the Discussion (detailed in Reviewer 1, Point 2, highlighted in bold) to acknowledge specific instances where reverse causality is plausible (Page 40-41, Lines 679-688/ 701-707).

> **Discussion:** "Safe family environments and open conversations about online safety may facilitate children's willingness to report incidents without fear of judgment or shame[70]. Such family climates may foster trust that enables disclosure after harm occurs, or regular parent-child engagement may create opportunities for proactive communication that make children feel comfortable seeking help. **However, our data cannot disentangle these pathways or establish directionality.** Second, consistent with not knowing where to seek help being the most common barrier to disclosure, knowing where to seek help was associated with higher disclosure, particularly through informal channels. Developmentally appropriate help-seeking education may be a low-risk, scalable prevention target, **however, this association was sensitive to model regularisation, suggesting that relationship may vary across countries."**
>
> "Although reduced stigma around sex and sexuality is theoretically associated with lower barriers to disclosure of sexual abuse, **this predictor was not robust across all modelling specifications.** Unexpectedly, inequitable gender attitudes were associated with higher disclosure rates in a subset of models. The relationship between beliefs about gender roles (e.g., preservation of male honour and acceptance of violence against women) and children's willingness to disclose **remains complex and difficult to disentangle[60,71]."**

With respect to methods that better address causality, we strongly agree with the reviewer here that instrumental-variable and longitudinal designs are ideal approaches. However, this study was not designed as longitudinal or to include instrumental variables. At present, such strategies are also constrained by incomplete and non-comparable subnational administrative indicators (e.g., internet penetration), and suitable panel data do not exist across these settings (see Response to Reviewer 2, Points 2-3).

To move beyond associations, any pursuit of causal identification in this sensitive area of research must rely on ethically appropriate and feasible designs. As a research team, we do not advocate experimental approaches that would increase risk for children or restrict support to existing victims (e.g., withholding help-seeking programs to create control groups) without robust safeguarding. Participatory and qualitative approaches are essential complements to causal identification. These methods can help clarify mechanisms (e.g., norms, power dynamics, barriers), capture contextual heterogeneity across settings, assess the feasibility of interventions, identify potential unintended effects and improve measurement validity with children and caregivers. However, they complement rather than replace causal identification efforts when causal inference is the primary goal. We have

updated the Discussion (Page 43, Lines 733-736) and Limitations (Page 43-44, Lines 751-756) to address reviewer's concerns.

> **Discussion:** "Participatory approaches that directly engage children with lived experience of technology-facilitated CSEA across rural, peri-urban and urban settings can generate region-specific insights that complement the population-level patterns identified[74]. Second, given the identification and retention challenges of traditional longitudinal cohorts in LMICs[75], prospective longitudinal or diary-based designs that follow children over time may be more feasible for establishing temporal precedence and determining whether associated factors prevent harm, facilitate disclosure, or both."

> **Limitations**: "Cross-sectional data precludes causal inference[77]; we cannot determine whether knowing where to seek help facilitated disclosure, or whether disclosed experiences increased awareness of support channels. Binary measurement (e.g., help seeking, premarital sex) of key constructs provides only preliminary estimates. As such, all findings should be interpreted as identifying plausible associations that warrant future testing through ethically appropriate and feasible methodological approaches."

Reviewer 3

I am focusing my review on data and methodology, and appropriate use of statistics and treatment of uncertainties.

Reviewer 3, Point 1: *Clarity for in-text descriptions of parameter estimates: On page 10 there are some in-text descriptions of parameter estimates from the models that require some additional explication to be meaningful. For instance, "Older children are more likely to experience technology-facilitated CSEA (posterior mean = 0.18, SD = 0.04, 95% Bayesian Credible Interval (CI) = [0.10 to 0.26], and posterior probability of direction (PD) = 99.9%)". In this instance, how age was parameterized in the models is unclear, so it's unclear whether this posterior reflects a year-over-year difference, or a contrast between some specific coding of "older" vs "younger". Similarly, continuing in the same sentence, "whereas gender was not credibly associated with it (posterior mean = -0.01, SD = 0.11, 95% CI = [-0.22 to 0.20], PD = 46%)" the paramererization of gender is unclear, so whether this posterior is describing the difference from girls to boys or boys to girls is not apparent. I recommend the authors carefully consider whether they are supplying sufficient information to map their conclusions to the posterior distributions for parameters of interest.*

Thank you. We have clarified the parameterisation in the text and report effects on both the log-odds and probability scales. Age is modelled as a continuous covariate in years (12–17), mean-centred at 15, so coefficients represent the per-year change in log-odds. Gender is coded with Boy as the reference; the contrast reported is Girl − Boy. Alongside posterior summaries, we now report average marginal effects (AMEs) on the probability scale, averaged over the observed covariate distribution (Page 17, Line 333-337).

"Average marginal effects showed that girls have, on average, a slightly higher predicted probability of CSEA than boys (~0.8 percentage points, AME = 0.008, 95% CI = [−0.6, 2.0]). However, this difference was small in magnitude and credibly indistinguishable from zero. By contrast, each additional year of age is associated with about 2.7 percentage points higher probability of experiencing CSEA (AME = 0.027; 95% CI = [2.2, 3.1])."

Reviewer 3, Point 2: *Clarity on priors: I believe the author's use of the horseshoe prior is a reasonable choice given the lack of LASSO capabilities in brms. However, the authors are not clear on their prior strategy across all other models, and for the other parameters in the horseshoe model. From reviewing the code, it is apparent that the authors use the brms defaults in all models, save for the horseshoe regression, in which they apply the horseshoe prior to the beta parameters, and leave all other parameters at the default. This means that there are flat, uninformative priors set on all beta parameters, and half student-t distributions set on the random effects. The authors should be clear about this, and justify this decision. Otherwise, just as with the horseshoe prior employed to achieve a specific purpose, the authors can be more deliberate in their choice of prior distributions to achieve valid inference.*

We apologise for the lack of clarity regarding our priors. We used two types of Bayesian models in our analysis. For standard multilevel models, we used brms default priors (Bürkner, 2017), flat priors for fixed effect coefficients, a Student-t (3, 0, 2.5) prior for the intercept, half-Student-t (3, 0, 2.5) priors for random effect standard deviations, and an LKJ (1) prior for the random effects correlation matrix. These defaults provide weakly informative regularisation that facilitates MCMC convergence without unduly constraining inference (Gelman, 2006).

For the horseshoe regression models, we specified a regularised horseshoe prior (Piironen & Vehtari, 2017) on the regression coefficients with 3 degrees of freedom and a global shrinkage scale of 0.5. This prior induces adaptive shrinkage and serves as a Bayesian alternative to LASSO for variable selection. All other parameters in the shrinkage models (intercept, random effects, correlation matrices) retained the brms defaults described above.

We selected the brms defaults to strike a balance between noninformative priors on fixed effects and weakly informative priors on hierarchical parameters. For variable selection, we chose the horseshoe prior because it is specifically designed for sparse estimation problems where many coefficients may be near zero (Piironen & Vehtari, 2017). These specifications are now detailed in the Methods section with references (Page 60, Lines 1134-1138).

Methods: "To reduce the risk of overfitting, we applied a regularised horseshoe prior on the fixed effect coefficients, specifying three degrees of freedom and a global scale parameter of 0.5. The horseshoe prior induces adaptive shrinkage and is a recommended Bayesian alternative to LASSO for variable selection[89]. All other parameters retained brms default priors: a Student-t(3, 0, 2.5) prior for the intercept, half-Student-t(3, 0, 2.5) priors for random effect standard deviations, and an LKJ(1) prior for the random effects correlation matrix[90,91].

References

Bürkner, P. C. (2017). brms: An R package for Bayesian multilevel models using Stan. Journal of Statistical Software, 80(1), 1–28.
Gelman, A. (2006). Prior distributions for variance parameters in hierarchical models. Bayesian Analysis, 1(3), 515–534.
Piironen, J., & Vehtari, A. (2017). Sparsity information and regularization in the horseshoe and other shrinkage priors. Electronic Journal of Statistics, 11(2), 5018–5051.

Reviewer 3, Point 3: *Appropriate parameter labeling in supplement tables: Similar to the latter point in the comment above, in Table 3 in the supplemental file, the parameter "Sex" should be labeled as whichever category is being contrasted to the intercept. Again, is this the difference from girls compared to boys, or vice-versa. This same point applies to all tables in the supplement file, as well as Figure 4 and extended Figures 1 and 2 in the main text*

We appreciate the reviewer's attention to this detail, as proper labelling reduces potential confusion about coefficient interpretation. We have revised all tables and figures in the manuscript and Supplement to indicate the contrast direction for selected predictors. Binary predictors are coded as 0 (reference) and 1 (comparison group) using brms default effects coding (−0.5, +0.5). Under this coding, the intercept represents the grand mean, and coefficients show the log-odds difference for the comparison category relative to the reference category.

We have updated the following with explicit contrast labels:

1. Supplementary Tables: 6-7; 9; 12-15; 17-18, and 20-31
2. Supplementary Figures 28-31
3. Main figures: Figure 4 and Extended Data Figures 3-4 have updated legends/axis labels indicating Girls (vs Boys) and Age (per year). We have also updated Figure 3 and Figure 5 (please see Reviewer 1, Point 4)
4. Model diagnostics: Supplementary Figures 34-38

Reviewer 3, Point 4: *Professional labeling for graphics: Similarly, Figure 16 in the supplement should alter the parameter labeling for "Sex1" to something more human-readable*

We replaced all non-descriptive labels (e.g., "Sex1") with Age (per year) and Gender [Girls vs Boys] across all figures and tables. Supplementary Figure 21 (formerly 16) has been updated accordingly, and the caption now states the reference group (Boys) explicitly. A thumbnail of the revised figure is included here for the reviewer's convenience.

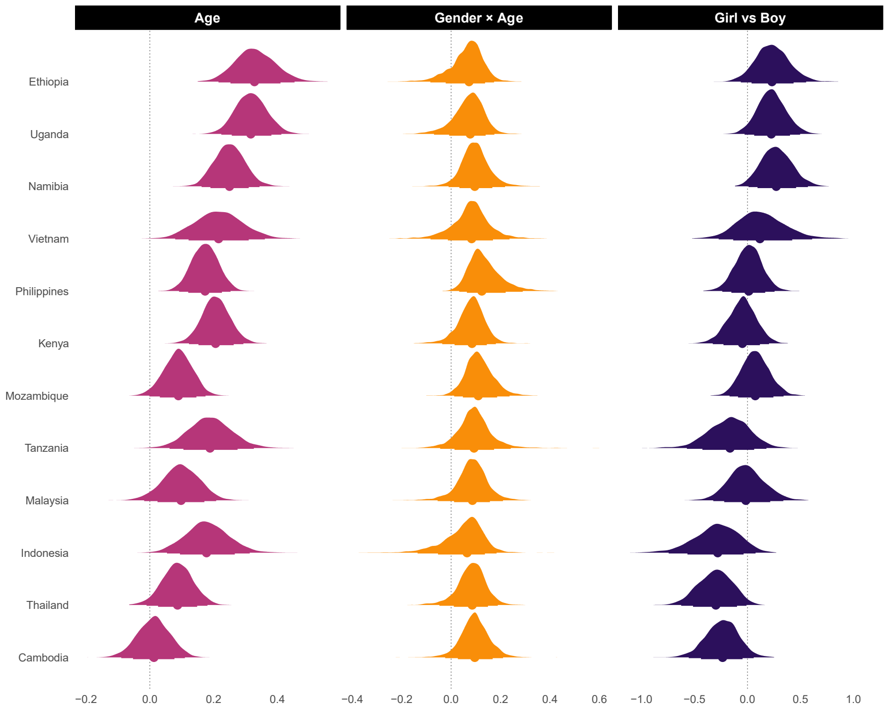

Supplementary Figure 21: Posterior densities of country-specific coefficients for Age (+1 year), Gender (Girls vs Boys) and Gender and Age interaction. Distributions show uncertainty in associations with experiencing technology facilitated CSEA across countries; vertical dotted lines mark zero.

Reviewer 3, Point 5: *Determination age x sex interaction and differences across countries: In the "Demographic Differences" section, the authors describe the positive association between age and CSEA, and the uncertain gender differences. However, then they note "a slight increase in the prevalence of … CSEA for older girls compared to boys…" presumably coming from the interaction effect in Table 3 in the supplementary materials. However, the use of the non-linear link function in the logit regression makes the interpretation of interactions from product terms somewhat unreliable (see Long & Mustillo, 2021). Similar to another analysis the authors employed, I would recommend using LOO cross validation here to compare a specification in which age and sex are additive, to this alternate specification in which they interact. This should be the basis of determining whether the difference the authors describe in the demographics section is systematic.*

Long, J. S., & Mustillo, S. A. (2021). Using predictions and marginal effects to compare groups in regression models for binary outcomes. Sociological Methods & Research, 50(3), 1284-1320.

Thank you for highlighting the interpretational pitfalls of product terms in logit models and for pointing us to Long & Mustillo (2021). In line with this guidance, we revised our analysis to: (a) compare additive versus interaction specifications using Pareto-Smoothed Importance Sampling Leave-One-Out (PSIS-LOO) cross-validation, which evaluates expected log predictive density (ELPD) as a measure of out-of-sample fit, and (b) report effects on the probability scale (both predicted probabilities and average marginal effects).

We have revised our interpretation to avoid overstating these patterns, presenting marginal effects with confidence intervals. For instance, when discussing the gender difference, we explicitly state: "this difference was small in magnitude and credibly indistinguishable from zero" (Page 17, Line 335) and use cautious language when discussing age-by-gender differences throughout. Similarly, in the discussion, we clearly state: "We found no gender differences in exposure rates" while explaining why gender-disaggregated monitoring remains important for other reasons (context, consequences, differential internet access) (Page 39, Line 640).

Supplementary: We contrasted an additive model (age and gender without interaction) with a model including age by gender. Both models showed satisfactory PSIS-LOO diagnostics (all Pareto-k < 0.7; max k = 0.33 for the additive model and 0.39 for the interaction; 0 observations > 0.7) and small Monte Carlo SEs. Predictive performance did not differ meaningfully (elpd_diff = −0.9, SE = 1.9), indicating no systematic out-of-sample improvement from adding the interaction. Given the theoretical relevance of potential age-by-gender differences and because prediction is not worse with the interaction, we retain the interaction as our main specification, while interpreting it via marginal effects and predicted probabilities.

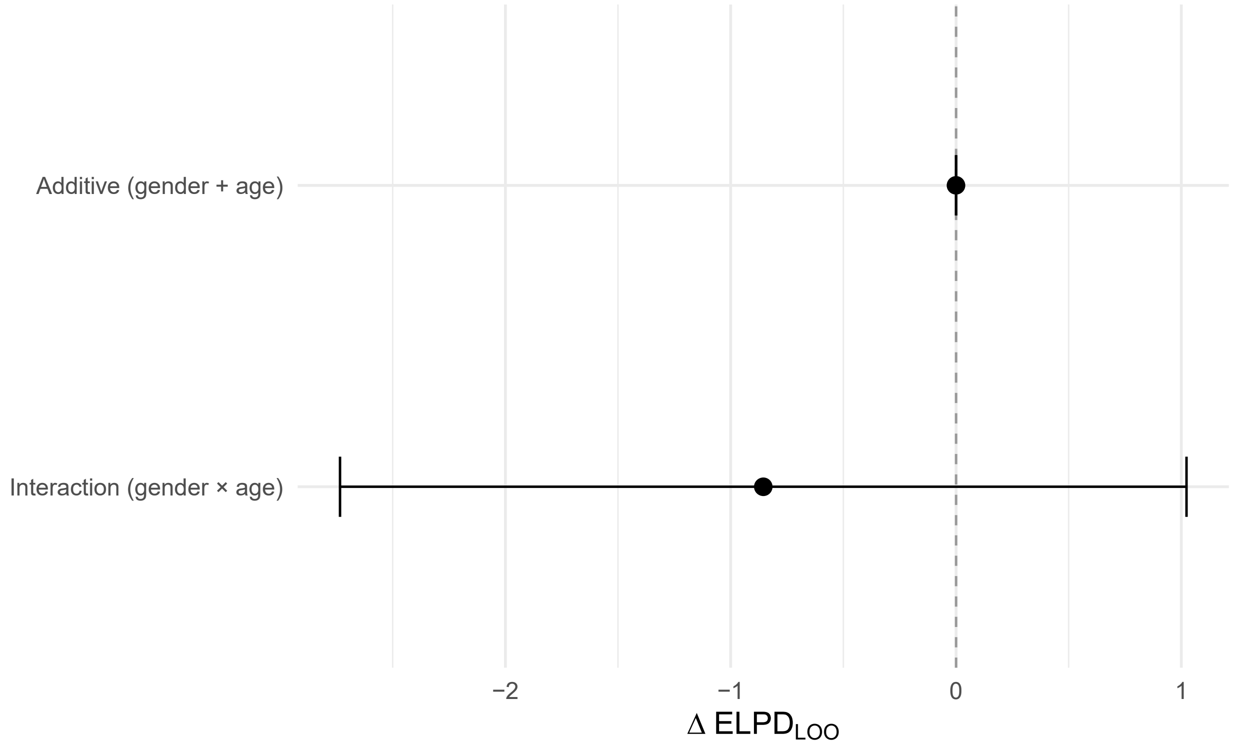

Supplementary Figure 31: Leave-one-out cross-validation (LOO) comparison of multilevel models by country: a random-intercepts-only model versus a model with both random intercepts and random slopes of victimisation. Points show the expected log predictive density (ELPD) difference (ΔELPD) relative to the best model; error bars are

±1 SE from LOO. Positive ΔELPD indicates better out-of-sample predictive fit. Models: Additive (gender + age) and Interaction (gender x age)

To present results on the probability scale, we report both a predicted probabilities in Supplementary Table 7 and average marginal effects to summarise group differences in directly interpretable units in the main results (as detailed in the Point 1 response) (Page 59, Line 1111-1118).

Methods: Because logit-scale interaction coefficients can be hard to interpret[88], we report predicted probabilities and average marginal effects (AMEs) for gender, age, and their interaction. Uncertainty reflects posterior draws and is summarised with 95% credible intervals. We also compared additive and gender and age interaction specifications using Pareto-smoothed importance-sampling leave-one-out cross-validation (PSIS-LOO) (Supplementary Figure 31). The difference in expected log predictive density (ΔELPD) was −0.9 (SE = 1.9), indicating no meaningful improvement in out-of-sample predictive performance from including the interaction.

Supplementary

1) Predicted Probabilities: We calculated the predicted probabilities of technology-facilitated CSEA at ages 12 and 17 for boys and girls in each country, estimated from the Bayesian multilevel model. Values are posterior means with 95% credible intervals, averaged over the observed distribution of covariates. These estimates are provided to aid interpretation on the probability scale.

Country	Age	Sex	Estimate	Lower Bound	Upper Bound
**Cambodia**	12	Boy	0.176	0.127	0.235
**Cambodia**	12	Girl	0.113	0.077	0.159
**Cambodia**	17	Boy	0.151	0.11	0.194
**Cambodia**	17	Girl	0.146	0.104	0.195
**Ethiopia**	12	Boy	0.063	0.032	0.102
**Ethiopia**	12	Girl	0.065	0.036	0.103
**Ethiopia**	17	Boy	0.223	0.184	0.264
**Ethiopia**	17	Girl	0.292	0.235	0.354
**Indonesia**	12	Boy	0.052	0.028	0.084
**Indonesia**	12	Girl	0.034	0.018	0.055
**Indonesia**	17	Boy	0.104	0.07	0.15
**Indonesia**	17	Girl	0.088	0.057	0.124
**Kenya**	12	Boy	0.148	0.102	0.198

**Kenya**	12	Girl	0.114	0.081	0.154
**Kenya**	17	Boy	0.283	0.231	0.342
**Kenya**	17	Girl	0.306	0.249	0.365
**Malaysia**	12	Boy	0.092	0.059	0.135
**Malaysia**	12	Girl	0.072	0.048	0.107
**Malaysia**	17	Boy	0.117	0.082	0.156
**Malaysia**	17	Girl	0.135	0.093	0.183
**Mozambique**	12	Boy	0.231	0.169	0.305
**Mozambique**	12	Girl	0.187	0.135	0.252
**Mozambique**	17	Boy	0.259	0.21	0.308
**Mozambique**	17	Girl	0.322	0.267	0.38
**Namibia**	12	Boy	0.107	0.07	0.151
**Namibia**	12	Girl	0.104	0.071	0.143
**Namibia**	17	Boy	0.241	0.19	0.296
**Namibia**	17	Girl	0.337	0.276	0.403
**Philippines**	12	Boy	0.232	0.171	0.302
**Philippines**	12	Girl	0.169	0.124	0.217
**Philippines**	17	Boy	0.335	0.265	0.403
**Philippines**	17	Girl	0.402	0.337	0.469
**Tanzania**	12	Boy	0.056	0.031	0.09
**Tanzania**	12	Girl	0.036	0.019	0.059
**Tanzania**	17	Boy	0.106	0.076	0.14
**Tanzania**	17	Girl	0.109	0.071	0.154
**Thailand**	12	Boy	0.176	0.128	0.234
**Thailand**	12	Girl	0.111	0.077	0.152
**Thailand**	17	Boy	0.211	0.161	0.269
**Thailand**	17	Girl	0.189	0.142	0.24
**Uganda**	12	Boy	0.112	0.068	0.165
**Uganda**	12	Girl	0.113	0.073	0.162
**Uganda**	17	Boy	0.335	0.289	0.386
**Uganda**	17	Girl	0.422	0.361	0.483

**Vietnam**	12	Boy	0.034	0.017	0.06
**Vietnam**	12	Girl	0.03	0.016	0.05
**Vietnam**	17	Boy	0.076	0.048	0.109
**Vietnam**	17	Girl	0.099	0.064	0.143

Supplementary Table 7: Predicted probabilities of technology-facilitated CSEA at ages 12 and 17 for boys and girls in each country, based on posterior estimates from the Bayesian multilevel model. The table shows point estimates and 95% credible intervals

Reference: Arel-Bundock V, Greifer N, Heiss A (2024). "How to Interpret Statistical Models Using marginaleffects for R and Python." Journal of Statistical Software, 111(9), 1-32. doi:10.18637/jss.v111.i09 https://doi.org/10.18637/jss.v111.i09.

Reviewer 3, Point 6: *Determination of country differences in associations via random slopes and multimodel comparison: In Table 13, the author(s) present the results of their LOO cross validation to determine whether the association between predictors of CSEA disclosure vary across countries. This seems to contrast two possibilities – a model in which the associations between each predictor and the outcome are consistent across all countries, and then a second model in which the association between *every* predictor and the outcome varies across countries. It is not surprising that the LOO reveals the latter model to not be a systematic improvement over the baseline. I believe the authors are at risk of throwing the baby out with the bathwater here. What I would recommend is that the authors need to consider each predictor individually here. You have a baseline additive model, and then estimate a series of supplemental models in which each predictor is allowed to have a random slope, one at a time (while still including all other predicitors as fixed effects). The LOO is then compared for each model back to the baseline to determine whether *that specific predictor* has an association that varies across the countries.*

This is an important point. We thank the reviewer for this suggestion to probe country-level heterogeneity in a more targeted way. Following this recommendation, we revised our analytic strategy as detailed below.

Specifically, we compared a baseline additive model (all predictors fixed, random intercepts for country) to a series of supplementary models in which each predictor, one at a time, was allowed to have a random slope by country (e.g., the effect of inequitable gender attitudes varying across countries), with all other predictors remaining fixed. We compared each random-slope model to the baseline using approximate leave-one-out cross-validation (PSIS-LOO; Vehtari et al., 2017), reporting ΔELPD and standard errors. We examined Pareto-k diagnostics to ensure reliability; PSIS diagnostics indicated reliable LOO estimates for the baseline (max Pareto-k = 0.28–0.53; 0 observations with k > 0.7). Across all predictors, ΔELPD values ranged from -3.1 to 0.0 (SE: 3.4–4.3). In all cases, ΔELPD was smaller than its standard error, indicating that allowing predictor slopes to vary by country did not meaningfully improve predictive performance. The model with a random slope for inequitable gender attitudes yielded ΔELPD = 0.0 (SE = 0.0) relative to the baseline, likewise indicating no supported slope variation across countries.

Taken together, these results suggest that, in our 12-country sample, country differences in overall disclosure likelihood are well captured by random intercepts, and that heterogeneity in predictor–disclosure associations is small in statistical terms. However, this does not rule out contextual differences in lived experiences or in the interpretation of constructs across settings; rather, it suggests that any such variation did not translate into systematically different statistical associations in this sample. To minimise the risk that multiple imputation might induce shrinkage and blur evidence of slope heterogeneity, we conducted these random-slope sensitivity analyses on the complete-case dataset. This ensures that any observed slope variation reflects empirical cross-national differences rather than artefacts of the imputation model. We have revised the methods (Page 60-61, Lines 1139-1144) and supplementary accordingly (see Supplementary 33).

> **Methods:** "To assess whether predictor–disclosure associations varied by country, we compared a baseline additive model (all predictors fixed, random intercepts for country) to a series of models in which one predictor at a time had a random slope by country (e.g., inequitable gender attitudes allowed to vary by country). We used PSIS-LOO to compute ELPD and ΔELPD (SE) relative to the baseline model. ΔELPD values ranged from -3.1 to 0.0 (SE: 3.4–4.3), indicating no supported improvement from adding any single random slope (Supplementary Figure 32)."
>
> **Supplementary: Country-level heterogeneity in predictor associations (LOO)**
> Using leave-one-out cross-validation (LOO), we assessed whether allowing any single predictor's slope to vary by country improves predictive performance over a random-intercepts-only specification. All models showed acceptable diagnostics (max k = 0.28–0.53; no k > 0.7). ΔELPD versus the random-intercepts baseline ranged −3.1 to 0.0 with SE 3.4–4.3, placing all differences within one SE and indicating no supported predictive gain from allowing any single slope to vary by country. The model with a random slope for gender attitudes (inequitable attitudes towards sex) attained the highest ELPD, but its advantage over the fixed-slope baseline was small (ΔELPD = +1.6, SE = 3.4) and therefore not supported. Here we find that allowing any single predictor's association with disclosure to vary across countries does not yield a meaningful gain in predictive performance. Taken together, these additional LOO comparisons suggest that country differences are well captured by random intercepts, while slopes are broadly similar across settings in out-of-sample terms.
>
> We conducted this analysis on the complete case dataset, rather than the imputed dataset, to ensure that between-country variation in slopes was not confounded by imputation uncertainty. Since imputation involves partially pooling across countries, it could artificially reduce or inflate evidence for cross-national heterogeneity in predictor effects. The complete-case approach avoids this potential confound and ensures that observed patterns of between-country variation reflect true heterogeneity rather than artifacts of the imputation model."

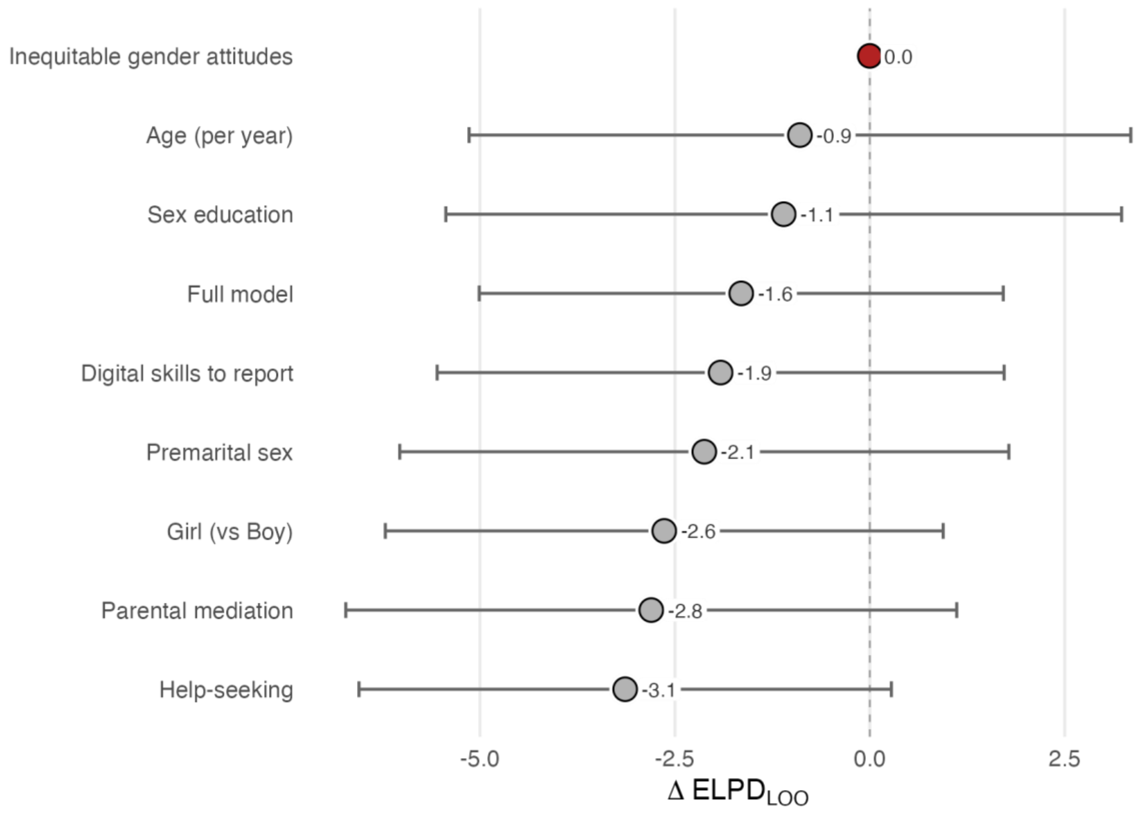

Supplementary Figure 32. Model comparison using PSIS-LOO. Points show the difference in expected log predictive density (ΔELPD) relative to the best-fitting model (marked in red; higher = better; 0 = best). Negative values indicate worse predictive performance. Error bars show ±1 SE of the difference. Across the baseline and predictor-specific random-slope models, ΔELPD values are small (−3.1 to 0, SE 3.4–4.3), placing all models within 1 SE of the best and therefore LOO-comparable. The random-slope model for inequitable gender attitudes is closest to the best (ΔELPD = −0.9, SE = 3.4), providing no supported improvement over the fixed-slope baseline.

Reviewer 3, Point 7: *The results from this analysis will make a more compelling and informative case regarding whether specific predictors indeed have associations that span multiple countries, or are specific to certain countries. Signed: Jason Rydberg, University of Massachusetts Lowell*

We are grateful to the reviewer for encouraging us to make this analysis more informative. In response, we examined predictor-specific heterogeneity across countries. We have also restructured all analysis code to make our modelling decisions more transparent. The updated code, including a detailed README and rendered HTML files, is available on Open Science Framework repository (in the *code/revised_code_v2*), and the Supplementary Materials have been revised accordingly.

Reviewer 4

Reviewer 4, Point 1: *Summary of the key results: Thank you for the opportunity to review the manuscript titled "Technology-facilitated child sexual exploitation and abuse in Eastern and Southern Africa and Southeast Asia". The paper uses nationally representative household data from 12 countries in Eastern and Southern Africa and Southeast Asia that were part of the Disrupting Harm surveys to examine technology-facilitated child sexual exploitation and abuse (CSEA).*
We thank the reviewer for their time on this manuscript.

Reviewer 4, Point 2: *Originality and significance: if not novel, please include reference*
The manuscript addresses a significant gap in the technology facilitated CSEA literature by investigating the prevalence of online abuse disclosure across countries. This is a highly significant, well-written manuscript.
We are grateful to reviewer for this positive assessment.

Reviewer 4, Point 3: *Data & methodology: validity of approach, quality of data, quality of presentation Appropriate.* No comment.

Reviewer 4, Point 4: *Appropriate use of statistics and treatment of uncertainties Appropriate.*

We appreciate the reviewer's recognition that the data and methodological approach are appropriate.

Reviewer 4, Point 5: *Conclusions: robustness, validity, reliability. See suggestions in section H.*

We have added supporting explanations (see detailed responses below), and revised the text accordingly.

Reviewer 4, Point 6: F. *Suggested improvements: experiments, data for possible revision. No data revisions suggested.* No comment.

Reviewer 4, Point 7: *G. References: appropriate credit to previous work?*
Suggest updated search for prevalence of technology-facilitated abuse:
See: Ben Mathews recent work in Australia; David Finkelhor in the USA; also:
Fry, Deborah, et al. "Prevalence estimates and nature of online child sexual exploitation and abuse: a systematic review and meta-analysis." The Lancet Child & Adolescent Health 9.3 (2025): 184-193.

We appreciate these suggestions, which have helped us to align the manuscript more closely with the current literature on technology-facilitated CSEA prevalence. Several of the recommended works were already cited in the original submission, and we have now broadened and sharpened our discussion of them.

Specifically, Fry et al.'s (2025) Lancet Child & Adolescent Health systematic review and meta-analysis (now reference 24) was already included and provides the global pooled prevalence estimate that underpins our epidemiological framing. We also cited multiple studies by Finkelhor and colleagues and have expanded our discussion of definitional and methodological issues in prevalence research (Finkelhor & Tucker, 2015; Finkelhor et al., 2024; Turner et al., 2023). In line with the reviewer's suggestion, we now additionally reference the Australian Child Maltreatment Study analysis led by

Walsh and Mathews (2025; now reference 30), which reports nationally representative estimates of online child sexual victimisation. We also refine our discussion of other heterogeneity drivers: definitional scope (e.g., peer versus adult solicitation) and recall period, building on Fry et al.'s article. We also add methodological clarification on sampling denominators, distinguishing prevalence among internet-using children (individual-level harms) from prevalence among all children (population burden), which sets up our later distinction between prevalence estimates in the Results.

These revisions situate our work within the existing epidemiological evidence and more fully acknowledge key contributions in this area (Page 7-8, Lines 161-174).

> **Introduction:** "Research on technology-facilitated CSEA has expanded substantially in HICs[24], yet has focused predominantly on prevalence[25–27]. Nationally representative surveys reveal wide variation in prevalence estimates due to definitional[18] and methodological choices such as whether peer or adult solicitation is counted[28], the recall period used (past year versus lifetime)[24], and sampling denominators (all children versus internet users only)[29]. For instance, 17.7% of Australian 16–24-year-olds reported adult online sexual solicitation before age 18[30] , while U.S. estimates indicates adding online abuse items can increase overall CSA prevalence estimates from 13.5% to 21.7% depending on how online harms are opertionalised[25]. A recent systematic review and meta-analysis estimates that roughly one in 12 children globally has experienced online CSEA (pooled past-year prevalence 8.1%)[24]. While such studies serve critical epidemiological functions such as mapping population burden, guiding resources, and enabling surveillance[31], prevalence data alone cannot reveal whether children seek help after harm. Understanding disclosure pathways is therefore equally critical for effective prevention and response[32]."

> Fry D et al. Prevalence estimates and nature of online child sexual exploitation and abuse: a systematic review and meta-analysis. Lancet Child Adolesc. Health 9, 184–193 (2025).

> Finkelhor D & Tucker CJ. A holistic approach to child maltreatment. Lancet Psychiatry 2, 480–481 (2015).

> Finkelhor D et al. When is online sexual solicitation of a minor considered sexual abuse? Recommendations for victim prevalence surveys. Trauma Violence Abuse 25, 4117–4129 (2024)

> Turner, H. A., Finkelhor, D. & Colburn, D. Predictors of Online Child Sexual Abuse in a U.S. National Sample. J. Interpers. Violence 38, 7780–7803 (2023).

> Finkelhor, D., Turner, H., & Colburn, D. (2024). The prevalence of child sexual abuse with online sexual abuse added. Child abuse & neglect, 149, 106634. https://doi.org/10.1016/j.chiabu.2024.106634

> Walsh K., Mathews B., Parvin K., Smith R., Burton M., Nicholas M., Napier S., Cubitt T., Erskine H., Thomas H. J., Finkelhor D., Higgins D. J., Scott J. G., Flynn A., Noll J., Malacova E., Le H., Tran N. Prevalence and characteristics of online child sexual

victimization: Findings from the Australian Child Maltreatment Study. Child Abuse & Neglect. 2025 Feb:160:107186.

Reviewer 4, Point 8: (minor) Lines 225 – 227 *"Addressing other barriers such as feelings of shame, fears that the child, the perpetrator, or their family might get in trouble and underestimations of the severity of technology-facilitated CSEA will also be crucial for ensuring effective future interventions." Suggest adding "Addressing other barriers identified in this study such as (…)*

We incorporated the suggested wording, expanded the barriers identified in this study, and moved the sentence to the Discussion section (Page 39-40, Lines 660-663).

> "Addressing other barriers identified in this study, including worries about not being believed, self-blame, fear of repercussions for the child or perpetrator, concerns about confidentiality, and limited awareness of reporting options, will also be crucial for ensuring an effective child protection response."

Reviewer 4, Point 9: Lines 382 - 384 "*These estimates, however, provide likely conservative approximations of the true scale of technology-facilitated CSEA, as one would expect severe underreporting of this sensitive issue." There seems to be some conflation between disclosure and reporting in this sentence. The severe underreporting is often associated with reports made to formal channels, such as law enforcement and child protective services. Given that this is a victim survey at the population level, would you still expect severe underreporting? Perhaps the numbers in the present study don't reflect the true prevalence, given social desirability bias in the data collected from face-to-face interviews, or because some participants may still experience victimization until they turn 18. However, the underrepresentation in a victim survey differs from that in crime or social services data.*

We agree that our original wording conflated underreporting to formal authorities with non-disclosure in victim surveys. In this revision, we use *disclosure* only for self-reported experiences within this survey, not for formal reporting. We further clarify that, although population-based victim surveys are less affected by the selection biases inherent in administrative or service data, they may still underestimate prevalence due to social desirability bias, stigma and shame in face-to-face interviews, recall limitations of a one-year window (which misses cumulative and ongoing victimisation), and non-disclosure even under conditions of anonymity. In light of this, we replace underreported with underestimated to highlight that our figures should be interpreted as lower-bound estimates (Page 37, Lines 607–610).

> "While population-based surveys capture prevalence more accurately than administrative data or case reports, they likely still underestimate the true scale of sexual violence due to social desirability bias, stigma, shame, or fear of social repercussions, and ongoing victimisation[65]. Even under these conservative assumptions, this is a growing problem affecting millions of children globally."

Stoltenborgh, M., van Ijzendoorn, M. H., Euser, E. M., & Bakermans-Kranenburg, M. J. (2011). A global perspective on child sexual abuse: meta-analysis of prevalence around the world. *Child maltreatment,* 16(2), 79–101. https://doi.org/10.1177/1077559511403920

Reviewer 4, Point 10: *Lines 379 – 384 "Approximately one in six of the 11,912 internet-using children surveyed reported experiencing at least one instance of technology-facilitated CSEA during 2020-2021. When extrapolated to national population estimates, this translates to millions of children exposed to potentially harmful experiences online in these countries alone." It would be helpful to the reader if early on the authors included the range of internet using children across the 12 studied countries. Because the statistics "one in six" may be compared with other child sexual abuse related statistics that account for the entire population of children, I would suggest the authors add one sentence further highlighting that these numbers relate to internet using children and cannot be directly extrapolated to the general population of children.*

We agree with the reviewer that providing the size of the internet-using child population improves interpretability. This point also echoes Reviewer 2's recommendations.

For related analytical details underlying the denominators, please see Reviewer 2, Point 2. In brief, we: (a) report country-specific internet-use rates for 12–17-year-olds and summarise the country-level range at the start of the Results (Table 1); (b) present a dual-denominator perspective, reporting prevalence among internet users (individual-level harms) alongside prevalence scaled to all children (population burden conditional on internet exposure), with visualisations in Supplementary Figures 14–15; and (c) add a cautionary sentence in the Discussion noting that the one in six figure refers to internet-using children (Page 37, Lines 603–605).

> "It is important to note that this figure cannot be directly extrapolated to the general population of children."

Reviewer 4, Point 11: *In the sentence, "When extrapolated to national population estimates, this translates to millions of children exposed to potentially harmful experiences online in these countries alone." Is this accounting only the proportion of children who use technology? I recommend that the authors clarify.*

Thank you for raising this important distinction. Our population estimates are scaled to the number of internet-using children, not to the total child population. We have revised the text to make this clear in two instances (Page 12, Lines 276-280; Page 37, Lines 605-606).

> **Technology-facilitated CSEA:** "To calculate the proportion of all children aged 12-17 experiencing technology-facilitated CSEA, we multiplied each country's internet penetration rate (collected via Disrupting Harm household surveys) by prevalence among internet users (see Table 1 and Extended Data Fig. 2). Across 12 countries, this corresponds to approximately 10.7 million children (95% CI = [7,931,739, 13,181,985]) (Supplementary Figure 15)."

Discussion: "Scaled by the share of child internet users in each country, we estimate at least 10 million children were exposed to technology-facilitated CSEA over a single year alone."

We have also added clarification in the Methods section (Page 51, Lines 930-952) and Supplementary Note 2.2 Uncertainty Propagation, where we describe both the denominators and construction of population-level estimates. Specifically, Supplementary Figure 15 now visualises the components of the population estimates, showing how absolute estimated children affected are calculated based on overall prevalence. Estimated counts are obtained by multiplying overall prevalence of technology-facilitated CSEA by the national 12–17 population (UN Population Division estimates derived from the year 2020).

Uncertainty Propagation: "We propagated uncertainty from both survey components using Monte Carlo simulation to estimate 95% confidence intervals for population-level prevalence and victim counts. We drew 5,000 samples from each component's sampling distribution on the logit scale, calculated the product for each iteration, and extracted the 2.5th and 97.5th percentiles of the resulting distribution (see Supplementary Figures 12-17 for details). This approach treats the child and household surveys as statistically independent, justified by their separate sampling frames, child respondents, and field operations. We calculated standard errors using effective sample sizes (Kish's method) that account for survey weights. For the internet exposure component in countries where sample sizes were unavailable, we applied conservative default standard errors (3 percentage points for internet use and 5 percentage points for prevalence). These defaults affect only the width of the confidence intervals, not the point estimates.

Several considerations warrant caution when interpreting population-level estimates. First, internet exposure data were collected in 2020-2021. In rapidly digitalising countries, exposure rates have likely increased substantially since then, meaning our estimates do not represent current population burden. Second, the household measure captures regular internet use but may not capture occasional use outside the home (e.g., at schools or community centers), leaving our prevalence estimates conservative. Third, our framework assumes children without internet access cannot experience technology-facilitated harms, an assumption that cannot be empirically validated with our data. Given these constraints and propagated uncertainty, population-level estimates should be interpreted as illustrative indicators of relative burden across countries rather than precise national estimates. Full list of caveats are provided in Supplementary 2.2."

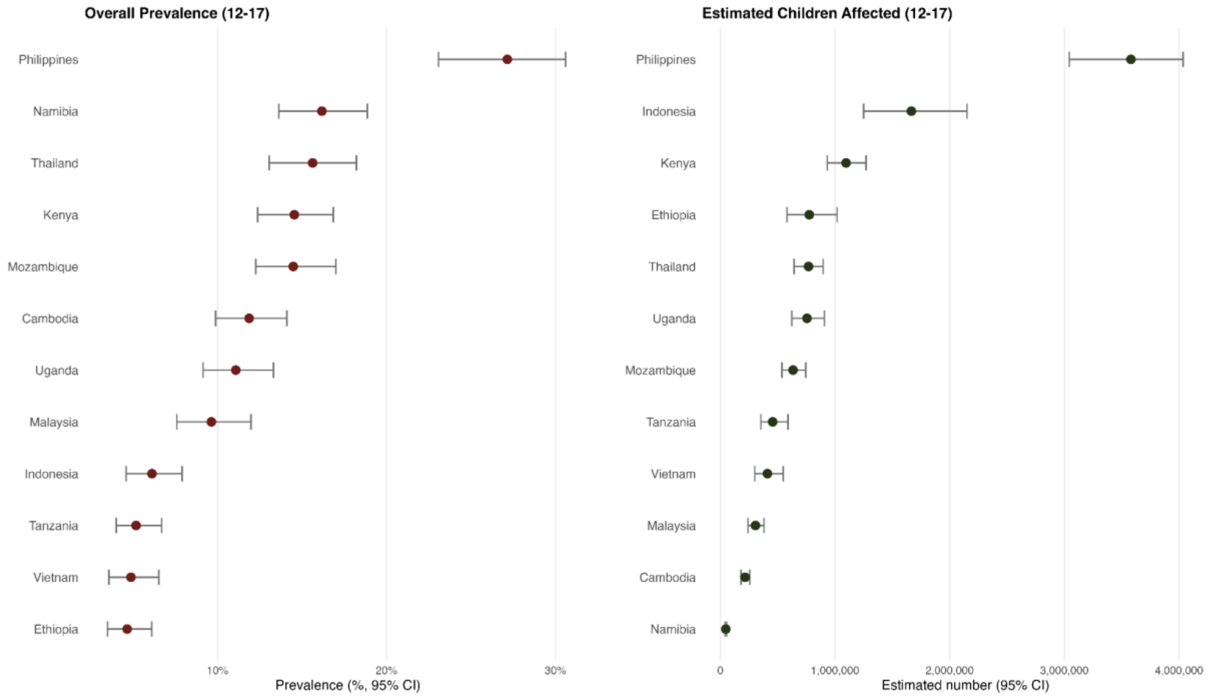

Supplementary Figure 15: Country-level prevalence and burden of technology-facilitated CSEA (ages 12–17). Left: estimated overall prevalence among all 12–17-year-olds (% with 95% CIs). Right: estimated number of affected children (95% CIs). For each country, overall prevalence is computed as the product of (a) prevalence among internet-using children (DH child survey) and (b) the proportion of all 12–17-year-olds who use the internet (DH household survey). Counts equal overall prevalence x national 12–17 population (UN WPP 2022, year 2020). Uncertainty is propagated via parametric Monte Carlo on the logit scale (5,000 draws per country); population counts are treated as fixed. Countries are ordered separately within each panel by the point estimate.

Reviewer 4, Point 12: *Line 406 – Heterogeneity refers to heterogeneity between countries?*

The reviewer is correct. Heterogeneity refers to between-country variation in the estimated associations, rather than within-country variation. We have revised the text to make the between-country interpretation clear (Page 31, Lines 497-506; Page 43, Lines 730-733).

Results: "The above results reflected average trends across 12 countries, examining country-specific estimates reveals substantial between-country heterogeneity in factors associated with disclosure of technology-facilitated CSEA (see Figure 5 and Supplementary Table 31). For disclosure to any channel, enabling parental mediation was associated with higher disclosure in 6 of 12 countries (95% CI excludes 0). Knowing where to seek help was positively associated with disclosure in 4 of 12 countries; older age was associated with lower disclosure in 5 of 12 countries; and more inequitable gender attitudes were associated with

higher disclosure in 5 of 12 countries. Country-level estimates are further disaggregated by formal versus informal channels in Extended Data Fig. 3, underscoring the complexity of cross-cultural comparisons."

Discussion: "First, substantial heterogeneity observed between the 12 countries, combined with sensitivity to modelling techniques, highlights the need for culturally specific investigations into the mechanisms that shape disclosure."

Reviewer 4, Point 13: *Lines 557 to 568 – I suggest the authors include a rationale for why they chose to classify disclosure between formal vs. informal channels. Teachers and helplines could also be classified as informal channels in the sense that they don't trigger an investigation, as with a report to the police or social service agencies.*

We thank the reviewer for this helpful suggestion and have revised the manuscript to clarify our rationale. Specifically, we explain that we use a role-based heuristic that groups disclosure recipients according to their typical role, rather than whether a specific disclosure leads to an investigation. As outlined in Supplementary Table 35, we classify as formal those channels positioned within or closely linked to statutory child-protection systems (e.g., police, social services, teachers, helplines), while informal channels provide primarily personal or familial support (e.g., parents, siblings, peers). We also explicitly acknowledge that the boundary between formal and informal channels can be fluid, particularly in Global South contexts where, for example, helplines may operate mainly in sometimes advisory roles. We therefore present this classification as an analytic heuristic designed to preserve conceptual clarity and comparability across diverse country contexts (Page 21, Lines 361–367).

Disclosure of Technology-facilitated CSEA: "We next identified to whom children disclosed CSEA, distinguishing between formal and informal channels. Formal channels are typically embedded within statutory child protection systems (e.g., police, teachers, or social workers), while informal channels offer more personal and familial support (e.g., friends, family, or peers). This distinction serves as an analytic heuristic to organise disclosure pathways based on the role of the recipient rather than the outcome of the disclosure (i.e., whether it triggers an investigation), consistent with past research (Supplementary Table 34)[54,55]."

Paine, M. L., & Hansen, D. J. (2002). Factors influencing children to self-disclose sexual abuse. *Clinical psychology review,* 22(2), 271–295. https://doi.org/10.1016/s0272-7358(01)00091-5

Gilbert, N., Parton, N., & Skivenes, M. (Eds.). (2011). *Child protection systems: International trends and orientations. Oxford University Press.* https://doi.org/10.1093/acprof:oso/9780199793358.001.0001

Channel type	Role-based definition	Typical recipients	Link to statutory system	Boundary/fluidity	Key references
**Formal**	Positioned within or directly linked to child-protection systems; recipients have statutory duties or formal referral pathways	Police; social services; teachers; helplines	Typically embedded in, or mandated to interface with, statutory protection	Not all disclosures to "formal" recipients trigger an investigation (e.g., helplines); roles vary across LMIC contexts	Paine & Hansen (2002); Gilbert et al. (2011); Larner (2022)
**Informal**	Provide personal, familial, or peer support without statutory mandate	Parents/caregivers; siblings; friends; other trusted adults	Not typically linked to statutory systems	Often the first line of disclosure; may facilitate (or impede) movement to formal channels	Paine & Hansen (2002); McElvaney (2015)

Supplementary Table 35: Role-based classification of disclosure channels (analytic heuristic; role-based, not outcome-based)

Supplementary references:
McElvaney, R. (2015). Disclosure of child sexual abuse: Delays, non-disclosure and partial disclosure. What the research tells us and implications for practice. *Child Abuse Review*, 24(3), 159–169. https://doi.org/10.1002/car.2280
Price, H. L., & Kehn, A. (2023). Potential reporters of suspected child maltreatment are sensitive to the amount of evidence and the potential consequences of reporting. Journal of Interpersonal Violence, 38(1–2), 391–417. https://doi.org/10.1177/08862605221081934
Larner, S. (2022). Facilitating children's informal disclosures of sexual abuse: The role of online counsellors at a national children's helpline. Journal of Child Sexual Abuse, 31(3), 276–296. https://doi.org/10.1080/10538712.2022.2047854

Reviewer 4, Point 14: *Line 682 – What is the range of percent missingness in the imputed variables?*

Prior to multiple imputation, item-level missingness ranged from 0.2% (enabling parental mediation) to 33.8% (inequitable gender attitudes). Supplementary Table 11 now presents the percent missing for each variable (Page 61, Lines 1160-1163):

Method: "Missing data ranging from 0.2% to 33.8% across variables. All missing data for the independent variables (see Supplementary Table 11) were imputed using the Multiple Imputation by Chained Equations (MICE) method and the mice package in R[93]."

Supplementary 4.1 Investigate Missingness: "We initially estimated the disclosure model using a single, complete-case dataset. To more robustly address missing data, we now re-estimate the models using multiple imputation. Specifically, we generated M = 30 imputed datasets, fitted the model separately to each, and pooled the results to obtain final estimates, thereby propagating imputation uncertainty into the inference. This step was motivated by the extent of missingness in key variables, summarised in the table below, and provides a more reliable basis for the main analyses."

Variable	Missing Count	Missing %
Inequitable gender attitudes	698	33.8
Premarital sex	301	14.6

Variable	Missing Count	Missing %
Digital skills to report	118	5.71
Help-seeking	108	5.22
Sex education	98	4.74
Positive parental mediation	5	0.242

Supplementary Table 12: Summary of missingness for key variables (only variables with missing values are shown)

Summary

We thank the reviewers for their thoughtful feedback on our work, which has substantially improved the manuscript. We sincerely hope that the revised version will meet the criteria for further consideration and contribute to advancing the research agenda on LMICs.

Yours sincerely,

Dr. Sakshi Ghai
Assistant Professor | Department of Psychological and Behavioural Science
The London School of Economics and Political Science
Houghton Street, London WC2A 2AE
lse.ac.uk/pbs

LSE THE LONDON SCHOOL OF ECONOMICS AND POLITICAL SCIENCE

09 March 2026

Reviewer Response Letter

Dear Dr Villamizar Santamaría,

We sincerely thank the reviewers and the editor for their re-evaluation of our revised manuscript. We are pleased that all four reviewers are satisfied with the revisions and have no outstanding concerns requiring major changes. Below, we respond to each editorial and reviewer comment in turn.

Editorial Comments

Editorial Point 1

Please use exact quantification whenever possible (e.g., in the Introduction, please put the exact number instead of '…Children are more likely to confide in peers…' [line 132]). Please make sure you do this throughout the manuscript.

We have reviewed the manuscript and replaced vague comparative language throughout. Where the cited literature reports qualitative findings without exact percentages, we revised the language to remove implied comparisons:

> "Children are more likely to confide in peers" to "Children tend to first disclose to peers rather than formal channels such as police, helplines, or teachers."
>
> "In high-connectivity countries (e.g., Malaysia, Thailand) this approximates most children" to "In high-connectivity countries (e.g., Malaysia, Thailand) this covers the majority of children, but substantial populations remain excluded in low-connectivity settings (e.g., Ethiopia)."

Editorial Point 2

Statistics: Please add direct comparisons to your quantitative descriptive results, i.e. where you say the amounts and how they are different, please add statistics to show whether these differences are statistically significant or not. When you do this, please be sure that all statistical details are reported in full (name of the test, test statistic, sample size/degrees of freedom, p value, effect size, when appropriate, etc., eg lines 85-86, etc.). Please also be sure to check the test assumptions and to correct for multiple comparisons, when necessary. If you do get non-significant results, please do not interpret them unless you add statistical support for the null (e.g. equivalence test or Bayes factors) and throughout the manuscript. If you have this information in a figure legend, please add it in the Main text and vice versa.

Response: We thank the editor for this guidance, which has strengthened the statistical rigour of the manuscript. We reviewed all quantitative statements and identified three categories: (1) sections reporting purely descriptive estimates (overall prevalence, types of abuse, disclosure) where no comparisons were made and thus no inferential tests were needed; (2) two sections describing country-level differences where we revised the language to remove implied comparisons; and (3) one section containing demographic comparisons (sex, age, urbanisation) that required formal statistical testing or equivalence testing. We address (2) and (3) below:

1. Country-level differences: We revised the language to be purely descriptive, reporting prevalence estimates with 95% confidence intervals and directing the reader to Extended Data Fig. 1. We removed language that implied formal comparisons and instead present the range of estimates without inferential claims. Between-country variation is formally addressed in the multilevel model via country-level random effects.

> Country-level prevalence estimates of technology-facilitated CSEA ranged from an estimated 5.5% [95% CI = 3.7 - 7.3, *n* = 54] of internet-using children in Vietnam to 29% [95% CI = 25.3 - 31.9, *n* = 271] in the Philippines. Extended Data Fig. 1 presents survey-weighted prevalence for each specific type of technology-facilitated CSEA across 12 countries, with 95% confidence intervals. For instance, less than 1% of children in Vietnam [95% CI = 0.0–1.5, *n* = 8] were asked to talk about sex or sexual acts online; in the Philippines, this figure was almost 9% [95% CI = 7.2–11.5, *n* = 89] (Figure 1 and Supplementary Figure 2-10; Table 52).

Country-level prevalence and population impact of technology-facilitated CSEA: We revised this section to remove comparative language (e.g., moderate, attenuated) and present estimates descriptively. We also corrected an error in one of the reported confidence intervals.

> To calculate the proportion of all children aged 12-17 experiencing technology-facilitated CSEA, we multiplied each country's internet penetration rate (collected via Disrupting Harm household surveys) by prevalence among internet users (see Table 1 and Extended Data Fig. 2). Across 12 countries, this corresponds to approximately 10.7 million children (95% CI = 9.9, 11.5 million; Supplementary Information Section 2.2; Figure 15). In countries with widespread internet penetration, such as the Philippines (95%), Malaysia (94%), and Thailand (92%), the prevalence for the child population closely mirrors that among children with internet access. In countries with more limited access, such as Ethiopia (25%) and Uganda (40%), the overall prevalence among all children was 5% and 11% respectively, reflecting differences in both internet exposure and prevalence among internet-using children. Countries with large youth populations such as Indonesia (6% among all children) and the Philippines (27% among all children) also face particular challenges as these rates translate into substantial absolute numbers of affected children. As internet connectivity continues to expand across LMICs, the population-level burden of technology-facilitated CSEA may grow substantially over the next decade.

2. Demographic Differences: For non-significant results (e.g., sex differences in CSEA prevalence), we conducted a Region of Practical Equivalence (ROPE) test on the relevant posterior distribution, following the editor's recommendation to provide statistical support for the null. Equivalence bounds were set at log(0.67) to log(1.50) on the log-odds scale. For sex differences, 99.7% of the posterior fell within the ROPE, providing strong evidence for practical equivalence. We have updated both methods and results accordingly.

> **Sex:** A Bayesian multilevel model confirmed no meaningful gender difference in the probability of experiencing technology-facilitated CSEA (posterior mean = -0.01, SD = 0.11, 95% Bayesian Credible Interval (CI) = [-0.22, 0.20], posterior probability of direction (PD) = 54%) with 99.7% of the posterior distribution falling within the region of practical equivalence (ROPE; odds ratios 0.67–1.50).

Methods: To evaluate evidence for null, we conducted Bayesian equivalence tests by calculating the proportion of posterior distribution falling within a region of practical equivalence (ROPE) as defined as odds ratios between 0.67 and 1.50, a range conventionally considered negligible.

Age: For significant results (e.g., age differences), the posterior mean and 95% credible interval are reported directly, as these clearly demonstrate a credible association.

Urban-rural: We have removed the inconclusive claim and changed to highlight that the credible interval included zero. We have also replaced Supplementary Figure 23 which previously contained an error.

Prevalence of technology-facilitated CSEA also varied by degree of urbanisation after adjusting for gender and age (Supplementary Figure 23; Table 39). Children in peri-urban settings reported the highest prevalence. Rural children had lower prevalence than peri-urban children (AME = −4.4 percentage points, 95% CI [−7.1, −1.9]), and urban children had higher prevalence than rural children (AME = 2.4 percentage points, 95% CI [1.0, 3.9]). The difference between urban and peri-urban settings was small and uncertain (AME = −2.0 percentage points, 95% CI [−4.8, 0.7]). Full model results reported in Supplementary Tables 8 and 9.

Figure legends and main text: We have added the statistical details from the Figure 2 legend in the main text and also updated the figure legend to guide the reader.

Main text: Predicted probabilities of technology-facilitated CSEA varied substantially across countries (see Figure 2 and Supplementary Table 7 and Figure 21). Among girls aged 17, predicted probabilities were highest in Uganda (42%, 95% CI = [0.36-0.49]) and Philippines (40%, 95% CI = [0.34-0.47]). Among boys aged 17, predicted probabilities were highest in Uganda (34%, 95% CI = [0.29-0.39]) and Philippines (34%, 95% CI = [0.27-0.40]). In Vietnam, predicted probabilities were among the lowest, ranging from 3% for boys aged 12 (95% CI = [0.02-0.06]) to 8% (95% CI = [0.05-0.11]) for boys aged 17 and 3% for girls aged 12 (95% CI = [0.02-0.05]) to 10% (95% CI = [0.06-0.14]) for girls aged 17. These differences could reflect variability in likelihood of reporting across countries rather than true prevalence differences alone.

Figure Legend: Figure 2. *Predicted probability of experiencing one or more forms of technology-facilitated CSEA by age and gender across 12 countries (N =11,912 participants). The x-axis shows age in years (12–17) and the y-axis shows predicted probability (0–0.50). Predictions are derived from a Bayesian multilevel logistic regression (Bernoulli family) with gender, age, and their interaction as fixed effects, and country-varying intercepts and slopes for gender, age, and their interaction. Lines show posterior mean predicted probabilities for girls (red) and boys (blue), with shaded bands indicating 95% credible intervals. Panels are ordered by country-level mean predicted probability.*

Editorial Point 3: *Please reduce the length of the title to 75 characters (with spaces) or less. Suggested: "Technology mediation in child sexual exploitation and abuse in Africa and Asia."*

Response: We have adopted the suggested title: "Technology mediation in child sexual exploitation and abuse in Africa and Asia."

Editorial Point 4: *Please add references to the abstract (if applicable).*

Response: We have added two citations to the abstract: ref 1 (Fry et al.) to support the claim that evidence on technology-facilitated CSEA remains limited, and ref 2 (Ghai et al.) to support the claim that evidence is lacking particularly across Africa and Asia.

1. Fry, D. *et al*. Prevalence estimates and nature of online child sexual exploitation and abuse: a systematic review and meta-analysis. *Lancet Child Adolesc. Health* **9**, 184–193 (2025).
2. Ghai, S., Magis-Weinberg, L., Stoilova, M., Livingstone, S. & Orben, A. Social media and adolescent well-being in the Global South. *Curr. Opin. Psychol.* **46**, 101318 (2022).

Editorial Point 5: *Please reduce the Abstract to 230 words or less. Currently there are 250 words.*

Response: We have edited it to 230 words now.

As digital access expands rapidly among children worldwide, technology-facilitated child sexual exploitation and abuse (CSEA), including online grooming, sexual solicitation, non-consensual image sharing, and sexual extortion, have emerged as an urgent yet underexamined digital harm[1]. Despite growing policy attention to online safety, evidence remains limited, particularly in low- and middle-income countries (LMICs), where most of the world's children live[2]. Here we analyse nationally representative survey data from 11,912 children aged 12–17 across 12 countries in Eastern and Southern Africa and South-East Asia, collected through the Disrupting Harm project in 2020–2021. We find that one in six internet-using children experienced at least one form of technology-facilitated CSEA, equivalent to over 10 million children. Despite this scale, most experiences went undisclosed, pointing to disclosure as a critical pathway for protection in the digital age. When children did disclose, they relied primarily on informal channels, especially friends, rather than formal reporting mechanisms such as police or helplines. Using Bayesian hierarchical models accounting for cross-country heterogeneity, we find that older children were less likely to disclose, whereas enabling parental mediation of online activities and children's knowledge of where to seek help following sexual harassment or assault were associated with higher rates of disclosure. These findings provide population-level evidence to inform prevention and response across LMICs, where coordinated action by policymakers, law enforcement, and technology companies is urgently needed to protect all children.

Editorial Point 6: *Please create a separate reference list for any methods references, making sure that the numbering continues from the main text references.*

Response: We have created a separate Methods reference list (refs 80–97), with numbering continuing from the main text references (1–79).

Editorial Point 7: *Please remove the main figures from the article file and re-supply them individually in an acceptable format (EPS, AI, PS, PDF, PPT, PSD, or XLS) with editable vector files.*

Response: All main text figures have been removed from the article file and re-supplied individually as separate editable vector files in **PDF** format.

Editorial Point 8: *You have more than one account on EJP. Please contact Nature Manuscripts to confirm all accounts and your primary email address.*

Response: I have contacted naturemanuscripts@nature.com to confirm the primary email address.

Editorial Point 9: *Please provide a supplementary information guide as a separate Word document.*

Response: A Supplementary Information guide has been prepared as a separate Word document including a cover page, author names and a brief summary (~50 words) for all supplementary items.

Editorial Point 10: *There are potential third party rights issues in the figures. Please check the sources of all illustrations and provide a completed third party rights table. In particular, please check Figure 1a–d, Supplementary Figures 1–10, 16.*

Response: Thank you for raising the query regarding potential third-party rights issues in the figures. We have carefully reviewed all figures in the manuscript, particularly Figure 1a–d and Supplementary Figures 1–10 and 16.

- **Figure 1a–d and Supplementary Figures 1–10** are original visualisations created by the authors using R (ggplot2) and, in the case of Supplementary Figure 1 using Tableau. Country boundary data were sourced from Natural Earth via the R package rnaturalearth. Natural Earth data are in the public domain and require no permission for reuse. We have added the following credit line to the relevant figure legends: Basemap boundaries: Natural Earth (public domain).
- **Supplementary Figure 16** is an original diagram created by the authors in Microsoft PowerPoint. All icons were sourced from the built-in Microsoft PowerPoint icon library, which are licensed for use in publications under Microsoft's standard terms and require no additional permission.

A completed third-party rights table is provided separately. Please let us know if any additional clarification or documentation is required.

Editorial Point 11: *Please remove the Extended Data figures and tables from the article file and re-supply them individually in EPS, JPEG, or TIF format.*

Response: All Extended Data figures have been removed from the article file and re-supplied individually as TIF files.

Editorial Point 12: *Please ensure that the text size in all figures is at least 5 pt Arial.*

Response: We have reviewed all figures and confirmed that text size is at least 5 pt (sans) throughout.

Editorial Point 13: *Please provide more details about IRB in the Main text (you can bring some of what's in the SI about this).*

Response: We have added full ethical approval details to the Methods section in prose, including the global IRB (HML IRB Research and Ethics) and national/institutional ethics approvals obtained in each of the 12 participating countries. We have also replaced Supplementary Table 1 with data collection field timelines.

> The Disrupting Harm Survey was reviewed and approved by a global institutional review board (HML IRB Research and Ethics). In addition, ethics approval was obtained from national or institutional ethics review bodies in each of the 12 participating countries. These included: the National Commission for Science, Technology and Innovation (Kenya); Makerere University School of Public Health and the Uganda National Council of Science and Technology (Uganda); the Cambodia National Council for Children and the Ministry of Interior (Cambodia); the Ministry of Health, National Committee on Bioethics for Health (Mozambique); the Medical Research and Ethics Committee (Malaysia); the Health Research Ethics Committee, National Institute of Health Research and Development (Indonesia); the Ministry of Health and Social Services Ethical Review Board (Namibia); the Ministry of Labour, Invalids and Social Affairs (Vietnam); the Philippine Social Science Council Ethical Review Board (Philippines); the Ethiopian Society of Sociologists, Social Workers and Anthropologists (Ethiopia); and multiple bodies in Tanzania: the Medical Research, National Bureau of Statistics, and the President's Office–Regional Administration and Local Government, with permits from the Tanzania Commission for Science and Technology and additional approvals from the Zanzibar Health Research Institute. In Thailand, the study was reviewed by a special panel at Mahidol University's Institute of Human Rights and Peace Studies, as no formal government ethics review process exists for social research.

Reporting Summary Checklists

Editor Point 1: Please ensure all data collection/data analysis software/tools/algorithms/packages mentioned in the manuscript are also listed in the reporting summary (with version numbers). Eg: ggplot2

Response: We have updated the reporting summary to include all software and packages used in data analysis, with version numbers.

> All analyses were conducted in R (version 4.4.1). Key R packages used include: brms, cmdstanr, posterior, loo, bayestestR, marginaleffects, emmeans, tidyverse, survey, srvyr, mice, ggplot2, and ggdist. A full list of packages and version numbers is provided in the GitHub repository README (https://github.com/sghai9/technology-mediation-csea)

Editor Point 2: Please elaborate on the study design indicating whether the data are quantitative, qualitative or mixed-methods.

Response: We have added this to the reporting checklist.

This study uses a quantitative, cross-sectional survey design. Data are drawn from the nationally representative household survey component of the Disrupting Harm project – a multi-sectoral research initiative led by UNICEF Office of Research – Innocenti, ECPAT International, and INTERPOL, with funding from Safe Online. While the broader Disrupting Harm project employed a mixed-methods design across its three partner organisations, this paper analyses only the quantitative survey data collected by UNICEF. Approximately 1,000 internet-using children aged 12–17 were surveyed in each of 12 countries across Eastern and Southern Africa (Ethiopia, Kenya, Mozambique, Namibia, Tanzania, Uganda) and Southeast Asia (Cambodia, Indonesia, Malaysia, Philippines, Thailand, Vietnam).

Editor Point 3: Please state whether the sample is representative. Also, please provide a rationale for the chosen study sample.
Response: We have added this to the reporting checklist.

The sample is nationally representative of internet-using children aged 12–17 in each country. A stratified random cluster sample with random walk within clusters was used. Children were randomly selected at household level if they were aged 12–17 and had used the internet at least once in the past three months. Survey weights were applied in three stages: design weight adjustments to reflect probabilities of selection (inverse probability weights), non-response weights to reduce non-response bias, and post-stratification weights to adjust for differences between the sample and population distributions. The sampling design aimed at achieving full or near-full national coverage, making findings representative of internet-using children aged 12–17 in each participating country, rather than the general child population.

Editor Point 4: *Please describe the statistical methods that were used to predetermine sample size OR if no sample size calculation was performed, describe how sample sizes were chosen and provide a rationale for why these sample sizes are sufficient. For qualitative data, please indicate whether data saturation was considered and what criteria were used to decide that no further sampling was needed.*

Response: We have added this to the reporting checklist.
The total analytic sample comprises N = 11,912 internet-using children across 12 countries. No formal a priori power calculation was conducted. Sample sizes of approximately 1,000 children per country were determined by UNICEF Office of Research: Innocenti and Ipsos as a standard survey design target for nationally representative household surveys, sufficient to provide adequate precision for country-level prevalence estimates of technology-facilitated CSEA.

Editor Point 5: *Please elaborate on the data collection procedure, specifying the instruments used to record it. Also, please indicate if anyone was present besides the participants(s) and the researcher, and whether the researcher was blinded to experimental conditions and/or the study hypothesis.*

Response: We have added this to the reporting checklist.

> Data was collected by Ipsos MORI (and local Ipsos affiliates) on behalf of UNICEF Office of Research – Innocenti between 2020 and 2021. The survey instrument was adapted from the Global Kids Online questionnaire (originally developed by UNICEF Innocenti and the London School of Economics), with roots in the EU Kids Online survey (2012) with additional modules developed specifically for the Disrupting Harm project. The survey used a mixed-mode design combining computer-assisted personal interviewing and computer-assisted self-interviewing. Questions relating to children's life context and general internet use were administered face-to-face by trained enumerators to build rapport. Sensitive sections covering sex and sexual violence including the nine items assessing technology-facilitated CSEA were completed by children themselves in self-complete mode on a tablet or phone, primarily to protect privacy and to allow children to feel more comfortable responding. Enumerators remained available throughout to answer questions, address concerns, and activate the safeguarding and referral protocol if needed. Regardless of mode, children retained the right to skip any question or section or to stop the interview at any time. As this is an observational survey study with no experimental conditions, blinding of interviewers to the study topic was not applicable. While other household members were sometimes present during face-to-face portions, the self-complete mode for sensitive questions provided additional privacy protection.

Editor Point 6: *Please indicate the start and stop dates of data collection in the format month/year.*

Response: We have added Supplementary Table 1, which reports country-specific fieldwork start and end dates in month/year format together with target and achieved sample sizes. Data collection took place between January 2020 and November 2021 across 12 countries.

> Data collection took place between January 2020 and November 2021 across 12 countries. In Eastern and Southern Africa, fieldwork began earliest in Uganda (January 2020). Fieldwork in Ethiopia, Kenya and Tanzania was conducted between December 2020 and January 2021, whereas Namibia ran from December 2020 to February 2021. Mozambique had a staggered fieldwork schedule, beginning in the south in February 2021 and in the central and northern regions in May 2021, with all fieldwork completed in July 2021. In South-East Asia, fieldwork began in Cambodia (November 2020), followed by Thailand and Vietnam (November 2020 to February 2021) and Indonesia (November 2020 to February 2021). Fieldwork in the Philippines ran from January to April 2021, and Malaysia had the latest collection window, from April to November 2021. Each country targeted a sample of 1,000 internet-using children aged 12–17 years. Achieved unweighted sample sizes ranged

from 975 (Philippines) to 1,111 (Mozambique), and weighted sample sizes ranged from 950 (Philippines) to 1,016 (Uganda).

Editor Point 7: *Please provide a rationale for why randomization was not applicable to the study.*

Response: We have added this to the reporting checklist.

Randomization was applicable and was employed at multiple stages of the sampling procedure. A stratified random cluster sampling approach was used, with primary sampling units (clusters) selected with probability proportional to size. Within selected clusters, households were identified through a random walk procedure. Within eligible households, if more than one child aged 12–17 met the eligibility criteria (internet use in the past three months), a single respondent was selected. This multi-stage random sampling procedure ensured that every eligible child in each country had a non-zero probability of selection, and survey weights were applied to account for unequal probabilities of selection, non-response, and post-stratification.

Reviewer Comments

Reviewer 1

Thank you for the opportunity to review a revised version of , "Technology-facilitated child sexual exploitation and abuse in Eastern and Southern Africa and Southeast Asia." Below, I have included my original comments (#), the authors' response, often abbreviated for space (A), and any new response from me (B). I am happy to discuss any of the points below in more detail if needed.

1. Key Results

One of the most valuable contributions of this manuscript is its focus on low- and middle-income countries, an area where empirical research on technology-facilitated child sexual abuse (CSA) remains particularly limited. By situating the study in these contexts, the authors significantly expand the global scope of scholarship on CSA. Similarly, the attention to technology-facilitated CSA is important, as this remains an underdeveloped area of research. I found the authors' harm-focused framing—particularly their observation that "although not every online risk leads to harm, adopting a harm focused framing is crucial to fully recognising the severity of children's experiences, and safeguarding their rights"—to be a powerful and persuasive approach that directly addresses common criticisms of being "too sensitive" about behaviors such as online harassment. Methodologically, the approach is well described and appropriate, and the paper is written in an engaging, accessible style. Indeed, the opening sections are so well framed that I could imagine using them as an example for my graduate students.

A. We are grateful to the reviewer for their generous comments on the framing, methodological clarity, and accessibility of the manuscript.

B. No further discussion.

We thank Reviewer 1 for their generous and thorough engagement with our manuscript across both rounds of review. Their feedback has been instrumental in refining the paper.

2. Validity: I did not identify any flaws that would prohibit publication of this manuscript. The methods appear sound and the analyses are appropriately applied. My main concern lies with the underdeveloped discussion section, which would benefit from further elaboration before publication. Expanding this section would allow the authors to highlight the broader implications of their findings and strengthen the overall contribution of the paper.

A. To frame our revisions, we have reorganised our Introduction (see Point 7 below) around a children's rights and public health prevention framework that distinguishes primary prevention (prevention before abuse occurs), secondary prevention priorities (child protection and detection), and early tertiary priorities (response and recovery). To address the Reviewer 1's concerns, we have substantially revised the Discussion section by: (a) organising findings within this three-tier framework; (b) explicitly linking empirical patterns to actionable strategies at individual, family, cultural, and system levels; (c) proposing concrete future research directions; and (d) maintaining appropriate epistemic caution about causal inference while identifying prevention priorities. We acknowledge that our cross-sectional design cannot establish causal relationships or determine directionality of observed associations. However, our findings provide essential baseline data about factors potentially related to children's disclosure of technology-facilitated CSEA in contexts where such evidence has been largely absent.

B. I thank the authors for the effort put towards this suggestion. In my view, the discussion section feels more comprehensive and richer.

Response: We thank the reviewer for acknowledging the improvements to the discussion. The reorganisation around the three-tier prevention framework and the addition of concrete future research directions were directly motivated by the reviewer's insightful suggestion.

3. Originality and Significance

While the methodological approach itself is not novel, the focus on low- and middle-income countries is both original and highly significant. Scholars have increasingly called for greater attention to the Global South in research on child sexual abuse and prevention, yet the literature remains sparse. By centering these contexts, this paper addresses a pressing gap and provides insights that are highly relevant across disciplines, including criminology, public health, sociology, and international development. Given this, I expect the article has strong potential to be widely cited and influential.

A. We thank the reviewer for this generous assessment. By documenting both the prevalence and disclosure barriers of technology-facilitated CSEA, we provide foundational data that can potentially inform prevention programming, and future research agendas in LMICs.

B. No further discussion needed.

Response: We appreciate the reviewer's positive assessment.

4. Data and Methodology: The *methods employed are appropriate for the research questions, and the data are presented with care. However, some of the figures are overly complex and may be difficult for the average Nature reader to interpret efficiently. For instance, I found Figure 3 somewhat challenging and required more time than expected to distill its main points. Similarly, Extended Figure 1 is dense and may limit its utility. While I do not have a ready alternative, I encourage the authors to consider whether the data could be presented in a clearer visual format that prioritizes accessibility for a broad readership.*

A. We thank the reviewer for this constructive feedback. We have substantially revised Figure 3 and Extended Data Figure 1 to improve interpretability for a broad readership.

B. I went back to the original manuscript to remind myself what Figure 3 looked like. The revised Figure 3 is much better. By leaps and bounds. Nice work.

Response: We are pleased that the revised figures meet the reviewer's expectations for accessibility and clarity.

5. Appropriate Use of Statistics and Treatment of Uncertainties: *The statistical approaches appear appropriate and meets expected standards. One point that warrants clarification is the authors' use of multiple imputation by chained equations (MICE) to address missing data. While this is a reasonable choice, the manuscript does not explain why this method was selected over alternatives. A brief rationale would help strengthen confidence in the robustness of the analytic approach.*

A. We appreciate the reviewer's attention to the statistical rigor and have now expanded the Methods section to clarify our choice of MICE. We selected multiple imputation by chained equations (MICE) for three reasons. First, it flexibly accommodates the Disrupting Harm dataset's mix of continuous and categorical variables through sequential models. Second, it is a well-validated approach widely used in social science and public health (van Buuren, 2018). Third, it allows us to generate multiple imputed datasets (M = 30) and pool estimates using Rubin's rules, thereby propagating imputation uncertainty.

B. I thank the authors for this explanation. That approach seems justified to me.

Response: We thank Reviewer 1 for their careful consideration of our MICE rationale and for confirming that the approach is justified.

6. Conclusions: The conclusions are well grounded in the analyses and are presented with appropriate caution. None appear overstated. The results align with prior research while also highlighting meaningful variation across countries, which adds an important dimension to the literature. Overall, the conclusions are robust, valid, and reliable.

A. We thank the reviewer for their assessment of our conclusions as cautious. In revising the manuscript, we have reviewed the discussion section carefully to make sure that all conclusions remain appropriately framed within the limits of the data (as detailed above in Point 2).

B. I thank the authors for this effort.

Response: We appreciate the reviewer's positive assessment.

7. Suggested Improvements: Several areas could be strengthened in revision. First, the introduction and discussion would benefit from a more substantive treatment of primary prevention. The current framing positions detection as the first step in addressing CSA, and it is, when viewing this problem through a criminal justice lens. However, from a public health perspective, primary prevention is the more foundational step. The data presented here suggest insights into what prevention might look like, and further exploration of this theme would add significant value.

A. We agree with the reviewer that our original framing leaned too heavily on detection within a criminal justice lens and underplayed prevention as the foundational public-health priority. We have now restructured the manuscript such that disclosure evidence is explicitly positioned within a prevention framework.

B. I think the revisions made are effective. A prevention lens (as well as the children's rights perspective) to me offer a richer framework to understand response to sexual harm.

Response: We are grateful that the reviewer considers the prevention and children's rights framework an effective lens for the manuscript.

Second, the discussion of protective factors—such as sex education, digital skills, and knowledge of help-seeking resources—would benefit from greater clarity. Do the authors conceptualize these as individual-level factors within the social ecological model, or do they also operate at broader levels?

A. We thank the reviewer for this important question. Our original discussion did not clearly distinguish how the measured protective factors map onto the social–ecological model…We have revised the text to make this measurement conceptualisation more evident and note that effective prevention and response typically require multilevel approaches (Page 26-27, Lines 442-445; Page 42, Lines 723-726).

B. I thank the authors for these revisions.

Response: We thank the reviewer for this positive feedback.

Third, the treatment of barriers to disclosure could be refined. Since only those who did not disclose were asked about barriers, important nuances may be missed, as individuals who did disclose may also have faced barriers but eventually overcame them. I imagine that this is a feature of the design of the survey, and not the analytic decision made by the authors. Still, noting this limitation explicitly would improve transparency.

A. In this survey, only adolescents who did not disclose any technology-facilitated CSEA were asked about reasons for non-disclosure. As a result, our estimates capture barriers among non-disclosers and do not capture barriers that

children who did disclose may also have faced but overcame. We have added this as limitation in the manuscript to guide interpretation of these findings (Page 45, Lines 778-780).

B. Understood. I thank the authors for this clarification and consideration in the limitations section.

Finally, although the manuscript acknowledges the importance of including rural and peri-urban contexts in research, the discussion does not meaningfully engage with findings from these settings. Incorporating this dimension would further strengthen the contribution.

A. We thank you for this important observation which has allowed us to strengthen the urbanization analysis. Specifically, we now report adjusted estimates of technology-facilitated CSEA prevalence across rural, peri-urban, and urban settings using a Bayesian logistic regression that adjusts for age and gender and allows effects to vary by country.

B. I thank the authors for this additional set of analyses and agree with the approach they took in presenting the findings as suggestive/in need of future research.

8. References: The manuscript cites relevant and appropriate literature throughout. The references are up to date and sufficiently comprehensive.

A. We appreciate this positive feedback.

B. No additional discussion needed.

9. Clarity and Context

The abstract is clear, accessible, and appropriately structured according to the journal's guidelines. It provides an accurate and concise summary of the study, highlighting its scope and contributions in a way that will be understandable to a wide audience. The introduction is similarly well framed and situates the study effectively within the existing literature.

Again, I would like to highlight the quality of writing in these sections.

A. We are grateful for the reviewer's kind comments on the clarity and quality of the writing in the abstract and introduction.

B. No additional discussion needed.

Response: We appreciate the reviewer's positive feedback.

Referee #2: *Thank you for your thoughtful and thorough responses to my comments and for the revisions to the manuscript. I appreciate the care you took to address my concerns within the limits of the available datasets. Although some issues could not be fully resolved due to the nature of the data, I think these limitations are now clearly and*

appropriately acknowledged in the manuscript. Overall, I am satisfied with the revisions and believe they have strengthened the paper.

Response: We thank Reviewer 2 for their thoughtful and constructive engagement across the two rounds of review. Their feedback has been very helpful in strengthening the manuscript, and we appreciate their positive assessment of the revised version.

<u>Referee #3</u>

Reviewer 3, Point 1: *As was requested by the editors, I focused my review on the Bayesian modelling performed in this manuscript, and carefully considered the authors responses to my questions and recommendations in the first round of reviews. I have reviewed the revised manuscript, supplementary files, and the revised code posted in OSF. I appreciate the authors attentiveness to my questions, and believe the revisions resolve all of the queries that I had raised. At this point I have no further recommendations for refining the model parameterization or presentation of the results.*

Response: We thank Reviewer 3 for their careful and expert evaluation of the Bayesian modelling. We appreciate their confirmation that the revisions have addressed all outstanding concerns regarding model parameterisation and presentation of the results.

Reviewer 3, Point 2: (Remarks on code availability): *The code and README provided in the OSF is well documented and should be a good resource for those interested in understanding how the results were produced. There are some points where odd object names are used as headers (e.g., "LOO Victim" in the CSEA modeling script). These are accompanied by well documented write ups, but they did produce a temporary *squint* as I worked through the sections.*

Response: We thank Reviewer 3 for their positive assessment of the code and README posted on OSF. We also appreciate their noting that some section headers used idiosyncratic object names. We have revised these headers to use clearer descriptive labels (e.g., changing LOO Victim to Leave-one-out cross-validation).

<u>Referee #4</u>:

The authors were very responsive to reviewers' comments. All of my comments were appropriately addressed and I have no further concerns. I also appreciated the authors' inclusion of a dual denominator analysis in the revised manuscript.

We thank Reviewer 4 for their constructive feedback throughout the review process and are grateful for their positive assessment of the revised manuscript.

We hope that the revised version will meet the criteria for formal acceptance. We remain available to address any remaining queries promptly and would be honoured to see this work published in *Nature*.

Yours sincerely,

Sakshi Ghai (on behalf of all authors)

Assistant Professor | Department of Psychological and Behavioural Science
The London School of Economics and Political Science
Houghton Street, London WC2A 2AE
lse.ac.uk/pbs
